# A donor-supported silavinylidene and silylium ylides: boroles as a flexible platform for versatile Si(ii) chemistry†

**DOI:** 10.1039/d3sc00808h

**Published:** 2023-03-31

**Authors:** Julijan Sarcevic, Tobias Heitkemper, Paul Niklas Ruth, Leonard Naß, Maximilian Kubis, Dietmar Stalke, Christian P. Sindlinger

**Affiliations:** a Institut für Anorganische Chemie, Universität Stuttgart Pfaffenwaldring 55 70169 Stuttgart Germany sindlinger@iac.uni-stuttgart.de; b Institut für Anorganische Chemie, Georg-August-Universität Göttingen Tammannstr. 4 37077 Göttingen Germany; c Institut für Anorganische Chemie, RWTH Aachen University Landoltweg 1a 52074 Aachen Germany

## Abstract

Electron-deficient, *anti*-aromatic 2,5-disilyl boroles are shown to be a flexibly adaptive molecular platform with regards to SiMe_3_ mobility in their reaction with the nucleophilic donor-stabilised precursor dichloro silylene SiCl_2_(IDipp). Depending on the substitution pattern, selective formation of two fundamentally different products of rivalling formation pathways is achieved. Formal addition of the dichlorosilylene gives the 5,5-dichloro-5-sila-6-borabicyclo[2.1.1]hex-2-ene derivatives. Under kinetically controlled conditions, SiCl_2_(IDipp) induces 1,3-trimethylsilyl migration and adds exocyclically to the generated carbene fragment giving an NHC-supported silylium ylide. In some cases interconversion between these compound classes was triggered by temperature or NHC-addition. Reduction of silaborabicyclo[2.1.1]hex-2-ene derivatives under forcing conditions gave clean access to recently described *nido*-type cluster Si(ii) half-sandwich complexes of boroles. Reduction of a NHC-supported silylium ylide gave an unprecedented NHC-supported silavinylidene which rearranges to the *nido*-type cluster at elevated temperatures.

## Abstract 2

Boroles react with a nucleophilic silylene to give silylium ylides or bicyclic sila-borabicyclo[2.1.1]hex-2-ene. Reduction either affords clusters of Si(ii) or a donor-supported silavinylidene, which can be subsequently converted into the former.

## Graphical abstract

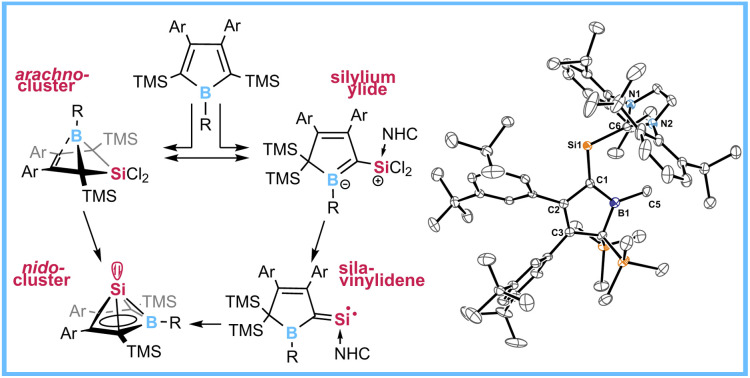

## Introduction

Free 1*H*-boroles (boracyclopentadienes) fulfill the Breslow-requirements for *anti*-aromaticity.^1^ The extent of *anti*-aromatic destabilization is distinct, yet much weaker compared to its iso-electronic and utmost reactive carbon analog, the cyclopentadienyl cation.^2^ Therefore, along with cyclobutadienes free boroles allow preparative synthetic chemistry and experimental exploration of, albeit weak, *anti*-aromaticity which is a very rare feature for small localized π-systems within the chemical space.^3^ The thermodynamic driving force of the exceptional electronic situation associated with a relieve from *anti*-aromatic destabilization has manifested in a series of examples for the past five decades since the first experimental descriptions of an authentic sample of pentaphenyl borole.^4–8^ As such, boroles are easily reduced to form respective non-aromatic (single electron transfer) or aromatic (two-electron transfer) species,^6,9–13^ feature remarkable Lewis-acidity,^5,14–17^ but foremost reveal numerous rearrangements and ring-expansion reactions that ultimately result in removal of the cyclic conjugate 4 π-electron system.^18–32^ Most recently, Erker and coworkers elucidated an intriguing reversible isomerism between 2,5-disilyl-1*H*-boroles and respective Wade–Mingos-type clusters of borapyramidanes, which may also account for an ultimately apparent scrambling of the substitution pattern.^33,34^

Migration of substituents around the central borole moiety in the course of chemical reactions has been previously noted in a few cases.^35–37^ We would now wish to report on our findings on a system, where depending on subtle electronic and steric changes in the substitution patterns, a borole proves to be a flexible platform allowing dynamic trimethylsilyl-migrations initially induced by a Si(ii) donor-addition to generate in principle two products including an unexpected NHC-supported silylium ylide. Almost coinciding with this study, the Scheschkewitz and Nakamoto groups reported the highly related reaction involving trimethylsilyl migrations of a cyclobutadiene with Roesky's PhC(N*t*Bu)_2_SiCl^38^ to give a donor-stabilized silene which can be reduced to give access to silapyramidane.^39^

**Chart 1:**
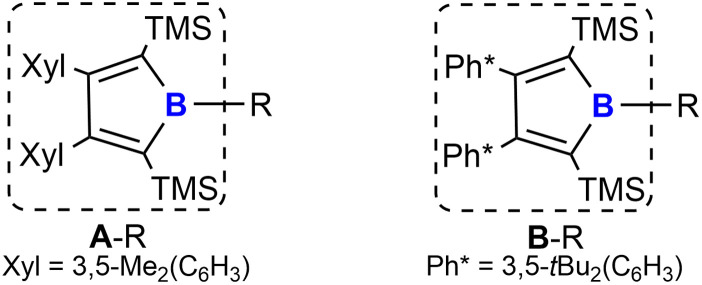
Borole substitution patterns under investigation.

We recently reported a synthetic route to a series of 2,5-disilyl-3,4-diaryl boroles [(Ar_2_TMS_2_C_4_)B–R, A/B–R] (backbone system A: Ar = Xyl = 3,5-Me_2_(C_6_H_3_); B: Ar = Ph* = 3,5-^*t*^Bu_2_(C_6_H_3_), Chart 1) and successfully applied boroles as a dianionic, [Cp]^−^-mimicking ligand for E(+II) coordination chemistry.^17,40–43^

**Scheme 1:**
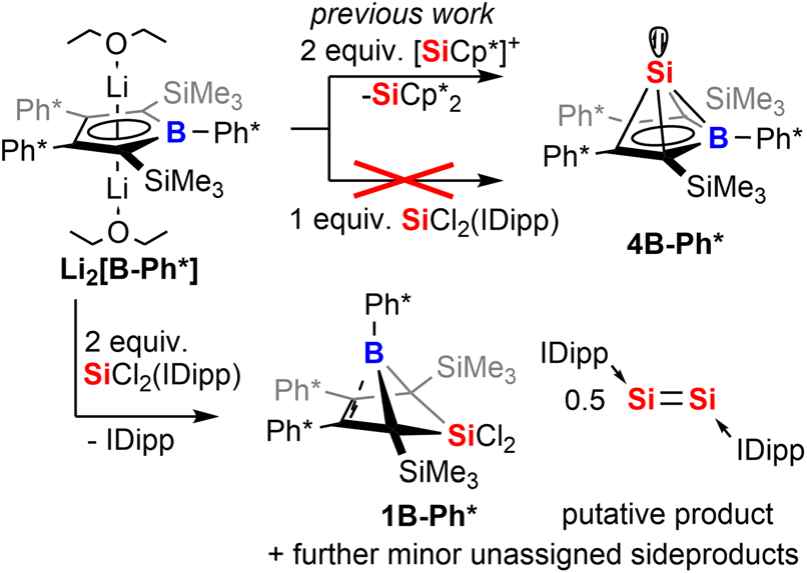
Reaction of borole dianion with SiCl_2_(IDipp).

In the following discussion of compounds, all product numbers will be accompanied by the borole identifier from which they derive. The Si(ii) half-sandwich *nido*-type cluster compound 4B-Ph* has been reported to be formed by the reaction of the borolediide [B-Ph*]^2−^ with two equivalents of Jutzi's [Cp*Si]^+^ as effective source of Si(ii) in a salt metathesis reaction also affording silicocene Cp*_2_Si as a side product (Scheme 1).^44–48^ However, the necessity to provide [Cp*Si]^+^ with weakly coordinating anions rendered this approach rather costly and very atom inefficient and we were interested in studying alternative approaches. However, meaningful reagents that function as suitable sources of Si(ii) for metathetic approaches are very scarce and mostly limited to said [Cp*Si]^+^, Cp*_2_Si,^49^ and Roesky's and Filippou's SiX_2_(IDipp) (X = Cl, Br, IDipp = 1,3-bis-2′,6′-^i^Pr_2_(C_6_H_3_)-imidazole-2-ylidene).^50–53^

## Results and discussion

### Initial findings

When [B-Ph*]^2−^ was respectively reacted with 1 equiv. of SiCl_2_(IDipp), the resulting mixture did not contain 4B-Ph* and revealed only *ca.* 50% conversion of [B-Ph*]^2−^ along with full consumption of SiCl_2_(IDipp). Accordingly, with two equivalents SiCl_2_(IDipp), starting materials were fully converted to form a complex mixture of compounds of burgundy colour that reveal the NMR-spectroscopic fingerprints of free IDipp and 5,5-di-chloro-5-sila-6-borabicyclo[2.1.1]hex-2-ene 1B-Ph*, a formal oxidation (*i.e. via* chlorination) product of 4B-Ph* (Scheme 1).

We reason that this product formation follows initial reduction of one equivalent of SiCl_2_(IDipp), putatively to Robinson's burgundy red Si^0^-species [Si_2_(IDipp)_2_]^54^ with the borolediide acting as a reducing agent (similar to the COT dianion (COT = 1,3,5,7-cyclooctatetraene)) rather than a salt metathesis precursor, regenerating the free borole B-Ph*.^55,56^ In a subsequent redox-process the free borole B-Ph* then reacts with SiCl_2_(IDipp) serving as a SiCl_2_ transfer reagent to form the 5-sila-6-borabicyclo [2.1.1]hex-2-ene 1B-Ph*. Formally, this formation can also be seen as a formal [4 + 1] cycloaddition reaction between the borole and dichloro silylene.

**Scheme 2:**
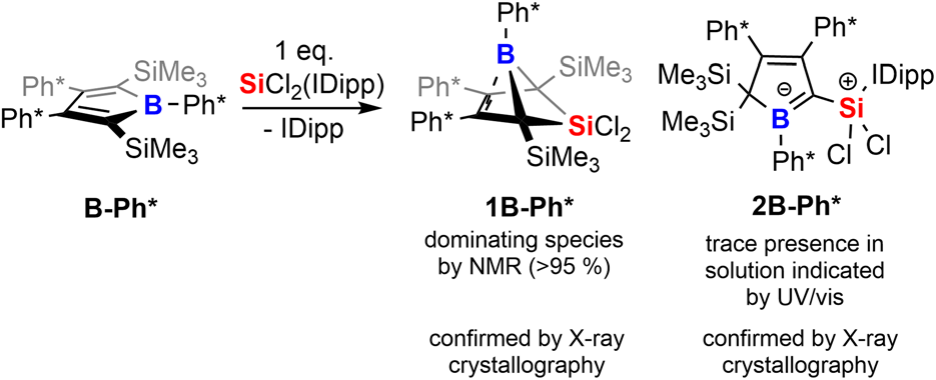
Reaction of free borole with SiCl_2_(IDipp).

To corroborate this reasoning, we directly reacted the free borole B-Ph* with SiCl_2_(IDipp). NMR spectroscopic monitoring of the reaction mixture indeed revealed dominating (>95%) conversion to the anticipated 5,5-dichloro-5-sila-6-borabicyclo [2.1.1]hex-2-ene 1B-Ph* along with free IDipp and again a red-orange colour. This distinct colour could however not be routed back to [Si_2_(IDipp)_2_]. Upon crystallization from the crude mixture, we were able to identify this “coloured impurity” as a minor share of orange-red crystals aside to colourless specimen of 1B-Ph* (of which the structure is deposited and documented in the ESI†). X-ray diffractometric examination of these mediocrely diffracting orange-red crystals clearly revealed the unexpected connectivity pattern and molecular structure of an SiCl_2_(IDipp) adduct to a rearranged borole-scaffold which results from the migration of a SiMe_3_-group (2B-Ph*, Scheme 2). The determined molecular structure of 2B-Ph*(see ESI†) indicated a unique bonding situation. Unfortunately, further corroboration of the structure of 2B-Ph* by NMR remained impaired by low-concentrations in crude mixtures and broad, poorly defined signals sets resulting from steric congestion (*vide infra*).

### Substitution-dependent selectivity and mechanistic proposal

**Scheme 3:**
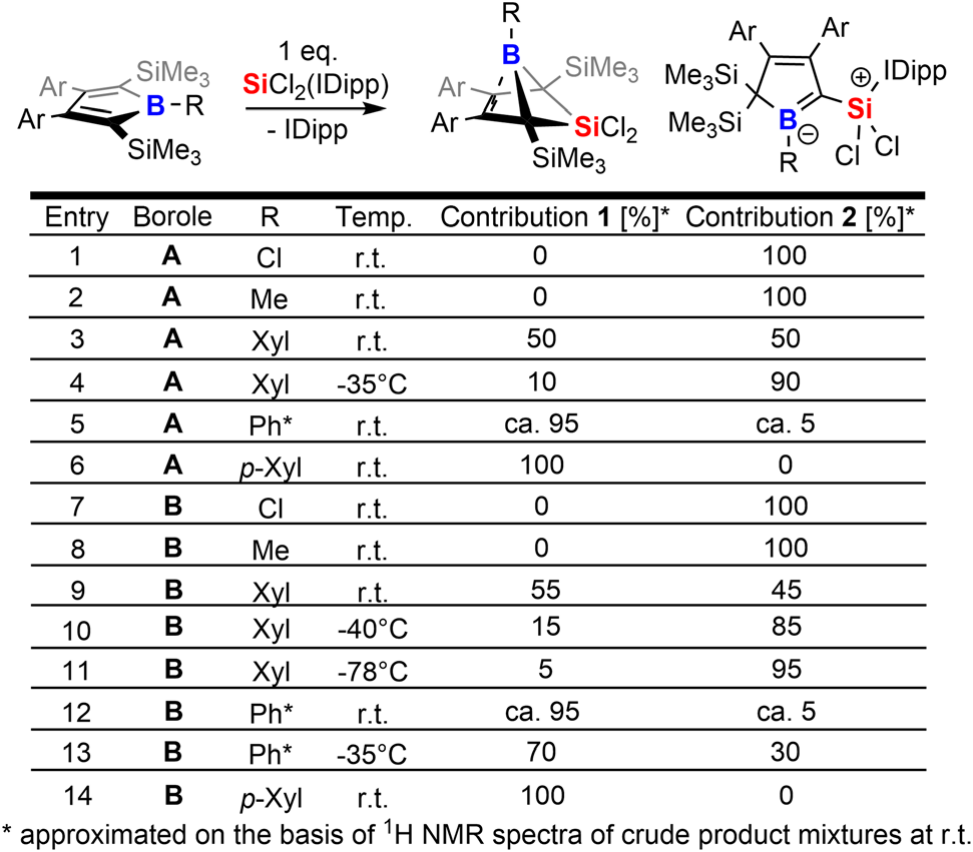
Tabulated summary for the product ratios between 1 and 2 at r.t. (20 °C).

The intriguing electronic bonding situation of the NHC-supported silylium ylide 2B-Ph* prompted us to investigate synthetic strategies for the selective formation of such species. We therefore probed the outcome of the reaction of SiCl_2_(IDipp) with a series of differently substituted free 2,5-disilylboroles iteratively tuning both the B-bound residue R (R = Cl, Me, Xyl [3,5-Me_2_(C_6_H_3_)], *p*-Xyl [2,5-Me_2_(C_6_H_3_)], Ph*) and the 3,4-bound aryls (Xyl, *vs.* Ph*) and thus changing electronic and steric profiles of the respective free boroles. The results of this screening are tabulated in Scheme 3.

From this experimental insight a few observations can be summarized: (1) the steric profile of the borole backbone, i. e. system A or B, has no major influence on the selectivity; (2) for small substituents at the boron-atom (Cl, Me), the NHC-supported silylium ylide 2 are obtained selectively (entries 1, 2 and 7, 8); (3) only very bulky substituents such as Ph* result in clear favourisation of 1 (entries 5, 12) making our initially probed system a particularly unfortunate case; (4) boron-bound aryl-substituents with one *ortho*-methyl group (such as *p*Xyl) partially blocking one hemisphere of the borole plane completely circumvent silyl migration and formation of the silylium ylide 2 in favor of 1 (entries 6, 14); (5) systems with a similar steric profile but non-π-plane interfering B-bound aryl group (such as *m*-Xyl *vs. p*-Xyl) are suitably balanced to give rise to both products 1 and 2 in almost equal shares at room temperature (20 °C, entries 3, 9); (6) conducting these unselective reactions at low temperatures notably increases the formation of NHC supported silylium ylide 2 in the obtained reaction mixtures indicating 2 to be a kinetically favored product (entries 9–11); (7) potential π-donation interactions from Cl atoms in chloroboroles do not influence the selectivity.

**Scheme 4:**
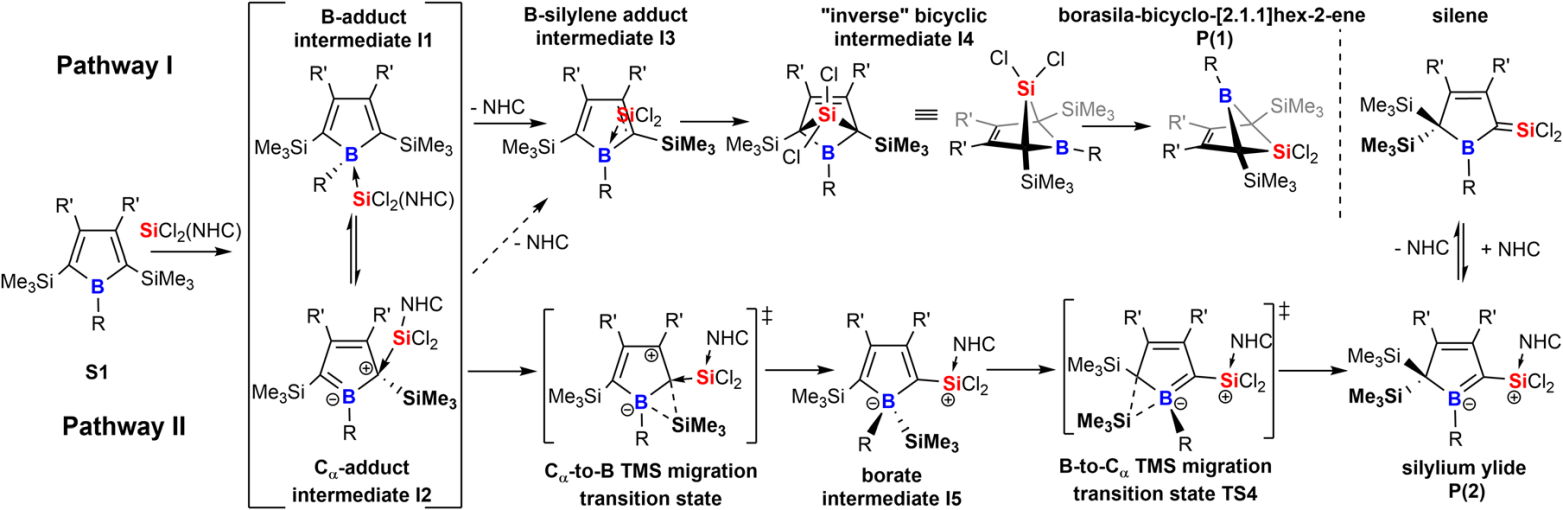
Proposed mechanistic steps for the formation of products 1 and 2.

Taking these observations into account we propose the following rivalling reaction pathways for the formation of 1 and 2, summarized in Scheme 4.

A more detailed account on the computational assessment of this mechanistic proposal including transition states for a sterically reduced model system (S1: R,R′ = Me; NHC: 1,3-Me_2_-imidazol-2-ylidene, Scheme 4) and a graphical depiction of relative energies of proposed species in the course of the reaction is documented in the ESI.† However, as sterics are most likely to play an important role, only relative free energies (BP86/def2-TZVPP and benzene solvation model) of intermediates with experimentally probed substitution patterns are given here. The anticipated initial step is an adduct formation of the Lewis-base SiCl_2_(IDipp) with free borole S1. This would intuitively add to the Lewis-acidic boron atom to give I1. So reported an example of a silylene-donor adduct to pentaphenyl borole.^57^ An alternative target for a nucleophilic attack at boroles is the C_α_ atom which reveals a vinylogous connection to the boron-site to result in a zwitter-ionic boratabutadiene-type adduct I2.^36^ For example, Braunschweig previously reported on a reversible B-/C_α_-atom adduct formation of 2,6-lutidine to boroles.^58,59^ Formation of both I1 (*ca.* −17 kcal mol^−1^) and I2 (*ca.* −2 kcal mol^−1^) would be exergonic. However, these putative intermediates I1 and I2 have not been experimentally observed in the course of the reaction, suggesting that subsequent processes must have low barriers. We propose the decisive forking of the pathway to occur at this stage.

Pathway I (Scheme 4) assumes an energetically uphill dissociation of the NHC. Computational NHC removal from both adducts I1 and I2 affords the identical structure I3 after optimization. I3 can be described as an adduct of an ambiphilic dichlorosilylene to the equally ambiphilic borole. The dissociation free energy at 298 K of the NHC from I1 is high for A-Xyl (18 kcal mol^−1^) and almost prohibitively high for A/B-Me (*ca.* 24 kcal mol^−1^). For our model system low barriers (*ca.* 1 kcal mol^−1^) for the formation of the bicyclic I4 from I3 and conversion/isomerization of I4 to the bicyclic product P1 are suggested. The overall free energies of the formation of P1 are exergonic by *ca.* −10 kcal mol^−1^.

We propose the pathway II to originate from the C_α_-adduct I2 from which a 1,2-SiMe_3_ migration from C_α_ to the B-atom occurs forming the silyl borate intermediate I5. Experimentally, no direct evidence for the involvement of a species of the I5-type were observed. However, the complete suppression of trimethylsilyl migration in systems with *ortho*-methyl substituted aryls at boron (A/B-*p*Xyl) is considered as a strong indirect proof for a migration *via* the boron atom. Subsequent 1,2-SiMe_3_-migration provides silylium ylide product class P2 and again small barriers (*ca.* 1 kcal mol^−1^) are suggested for the reduced model system for this step.

An example for comparable sigmatropic 1,2- and 1,3-shifts of hydrogen in borole-NHC adducts have been described by Braunschweig and coworkers.^35^ For all substitution variants probed, despite considerable steric bulk and thus repulsive interactions involved, the NHC-supported silylium ylides P2 were the global energy minimum (*ca.* −30 kcal mol^−1^*vs.*S1) of all species investigated and much more favoured than the bicyclic species P1 (−10 kcal mol^−1^*vs.*S1). For further details, see the ESI.† A key experimental finding is that mixing the starting materials in the cold favors the formation of the NHC-supported silylium ylides, suggesting that a decisive energy barrier at an early stage after bifurcation must be lower on the path towards P2. It seems reasonable that the NHC dissociation to form the I3 is the key barrier process.

The seemingly ideal test system, A-Xyl allowed us to develop protocols toward separation of either product from the respective mixtures. However, achieving rigorous separation to yield pure isolated products 1/2 from mixtures often remained a tedious task affecting the yields. The NHC supported silylium ylides 2 reveal considerable dipolar moment and are less soluble in hexane. Careful extraction of crude product mixtures with hexane thus resulted in removal of the more soluble 1. Removal of remaining free IDipp can be achieved by addition of ZnCl_2_ to solutions of 1 in hexane and precipitation of IDipp(ZnCl_2_). This protocol can also be applied for further purification and isolation of the other product.

**Scheme 5:**
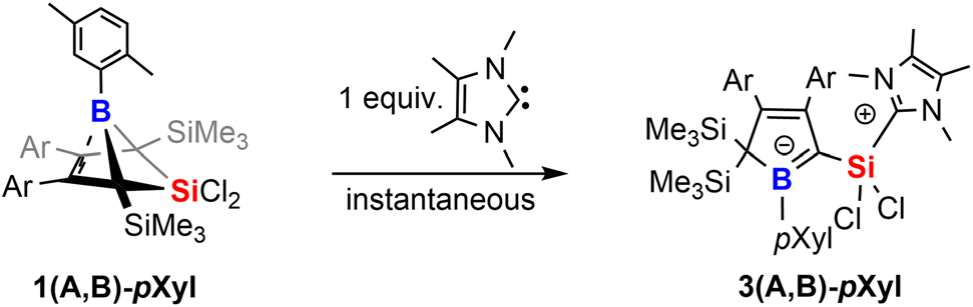
NHC-induced silyl-migration of 1.

However, we note the clean and irreversible thermal conversion of the supposedly thermodynamically favoured NHC-supported silylium ylides 2A-Xyl or 2B-Xyl into the respective bicyclic species 1A-Xyl or 1B-Xyl under NHC elimination at *ca.* 50–80 °C. However, when we treated the readily isolable bicyclic species 1A-*p*Xyl and 1B-*p*Xyl with the much smaller 2,3,4,5-tetramethylimidazol-2-ylidene (^Me^NHC), immediate colorization to yellow and essentially quantitative formation of the ^Me^NHC-supported silylium ylides 3(A,B)-*p*Xyl are formed (Scheme 5).

**Fig. 1:**
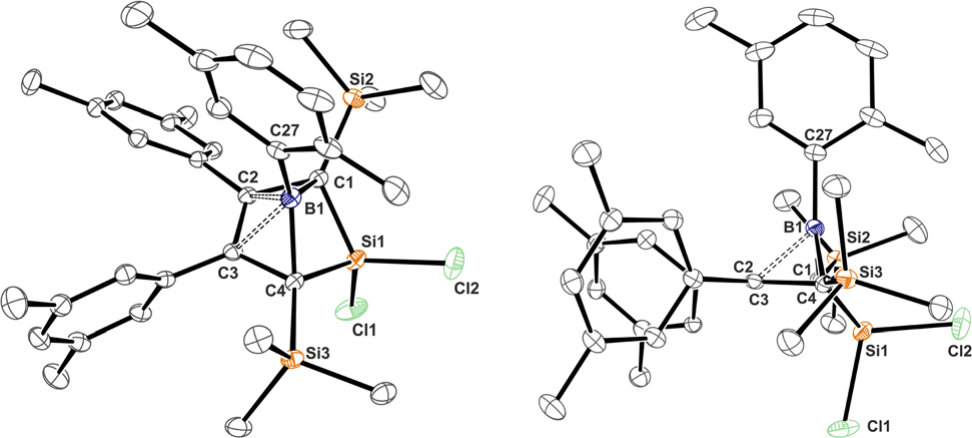
ORTEP of the solid state molecular structure of 5-sila-6-borabicyclo[2.1.1]hex-2-ene 1A-*p*Xyl. A second molecule in the asymmetric unit and H-atoms are omitted for the sake of clarity. Anisotropic displacement parameters are drawn at 50% probability. Selected interatomic distances [Å] and angles [°]: B1–(C2/C3)_centr_ 1.673; (C2/C3)_centr_–(C1/C4)_centr_–B1 82.7°, (C2/C3)_centr_–(C1/C4)_centr_–Si1 130.8°. Further details are listed in Table 1.

We assume that this product formation is enabled by the much less bulky NHC that can attack the SiCl_2_ moiety of the bicyclic compound 1 to proceed to a I2-type C_α_-adduct intermediate *via* cleavage of a Si–C_bridgehead_ bond. The X-ray structure of 1A-*p*Xyl reveals the *ortho*-methyl group of the boron-bound *p*Xyl-group to point toward the SiCl_2_ bridge (Fig. 1). We assume that a SiMe_3_ migration from this so generated I2-type intermediate is now possible, because the SiCl_2_(NHC) donor and the respective *ortho*-methyl group of the boron-bound aryl are located on the same half sphere of the borole plane allowing the SiMe_3_ group to migrate on the opposite, unblocked side. In the reactions of free *p*Xyl-bearing boroles with the SiCl_2_(NHC) nucleophile, the SiCl_2_(NHC) adduct would, for steric reasons, always attack from the unprotected side resulting in an *anti*-periplanar type arrangement of the *ortho*-methyl group and the SiCl_2_(NHC) fragment.

### Structural and spectroscopic properties of 1 and 2

**Fig. 2:**
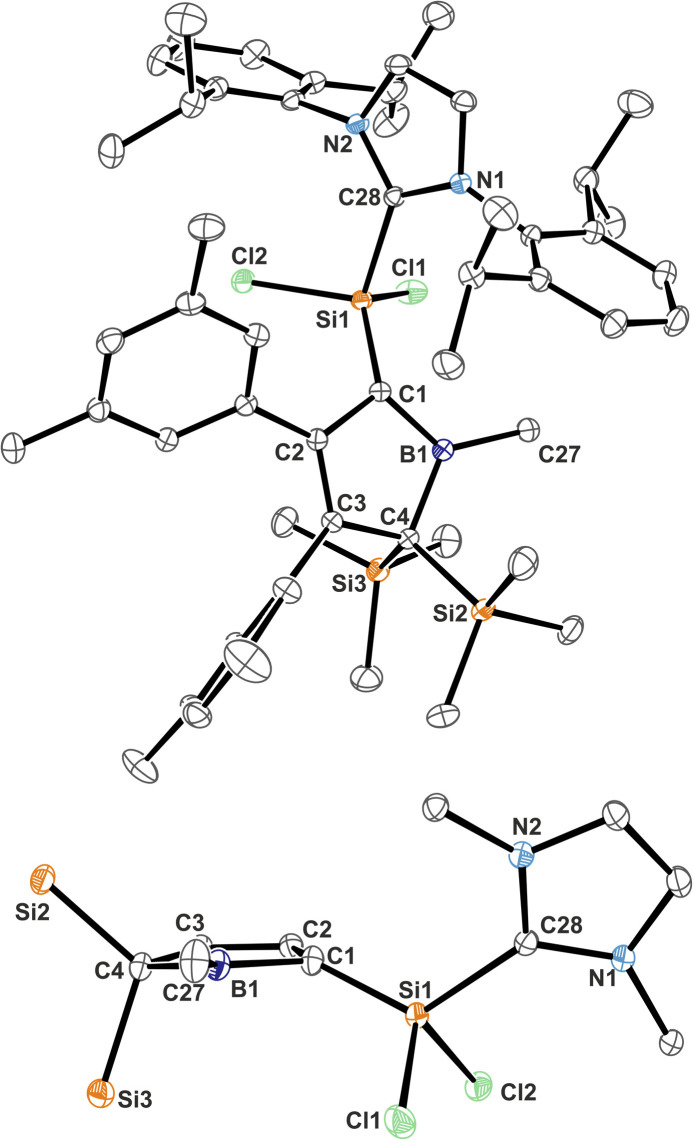
ORTEP of the solid state molecular structure of NHC-supported silylium ylide 2A-Me. H-atoms are omitted for the sake of clarity. Anisotropic displacement parameters are drawn at 50% probability. Selected interatomic distances are listed in Table 1.

Almost all derivatives based on the 3,5-^*t*^Bu_2_(C_6_H_3_)-substituted borole system B revealed very poor crystallinity and severe disorder and thus only allowed for collection of poor or mediocre data from X-ray diffraction. Derivatives of borole A however crystallized reliably. Structural features of substitution derivatives of 1 and 2, respectively, did not differ much within the series of compounds. Exemplarily representing their substitution derivatives, the solid state molecular structures of 1A-*p*Xyl (Fig. 1) and 2A-Me (Fig. 2) are depicted.

Compared to the starting material of a free borole with a localised 1,3-butadiene backbone of short C=C bonds (*ca.* 1.36 Å) and a long C–C bond (1.54 Å), the 5-sila-6-borabicyclo[2.1.1]hex-2-ene derivatives 1 all feature a short C2–C3 distance (*ca.* 1.4 Å) and elongated C1–C2/C3–C4 contacts (*ca.* 1.50 Å) indicative of a 2-butene moiety. This is in line with a description of 1 as the product of a formal [4 + 1] cycloaddition of the butadiene moiety of the borole with a [SiCl_2_] fragment.

The borane moiety stands almost perpendicular on the C_4_-plane [(C2/C3)_centr_–(C1/C4)_centr_–B1 82.7°] indicating clear interaction of the Lewis-acidic borane with the π-bond of the 2-butene moiety, corroborated by short C2/3–B1 contacts (B1–(C2/C3)_centr_ 1.673 Å). Such an arrangement has been observed previously in related products of pericyclic Diels–Alder reactions of free boroles with alkynes and alkenes and a cationic bicyclic hydrosilylium cation.^18,41,61^ Jutzi described a related η^4^-(Cp*R′)BR′ (R′ = Cp*(Br)_2_Si–) as an *arachno*-tetracarbaborane cluster.^62^

NHC-supported silylium ylide derivatives 2 reveal a rather unusual structural motif. Structural features are summarized in Table 1. The silyl group at C1 migrated to C4 and was replaced by the SiCl_2_(IDipp) moiety – an electronic equivalent of a formally neutral silyl group. This rearrangement goes along with a shortening of the C2–C3 contact to 1.371(1) Å indicative of a double-bond. The short C1–B1 contact (1.513(1) Å) lies between typical boron–carbon bonds (*ca.* 1.55–1.60 Å) and authentic boron–carbon double bonds (*ca.* 1.44–1.46 Å).^63,64^ Most notably, the Si1–C1 contact is rather short (1.7590(9) Å). Si–C single bonds usually amount to 1.85 Å while selected examples for Si=C have been reported at 1.702(5) Å (in silenes)^65^ or 1.774(2) Å (in NHC@Si-adducts to R_2_CSi=Si=CR_2_ heterocumulenes).^66^ The Si1–C1 bond vector is significantly deviating by 21° from the (B1–C1–C2)-plane in line with a deviation from (trigonal) planarity at C1 (sum of basal angles *ca.* 354°). A related motif of a slightly *trans*-bent geometry at the carbon atom was described by Roesky and coworkers for a cyclic amidinate-stabilised silene.^67^ This hints at a rather unusual electronic situation at carbon-atom C1 as, to the best of our knowledge, known NHC supported silylium ylides (or silenes) only reveal planar C-atoms.

**Table 1:** Selected structural and spectroscopic details of 5-sila-6-borabicyclo[2.1.1]hexenes 1 and NHC supported silylium ylides 2/3 (ref. 60)

Entry	B1–C1, C1–C2, C2–C3, C3–C4, C4–B1^a^	C1/(C4)–Si1^a^	Si1–Cl1/2^a^	Si1–C_NHC_^a^	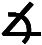 (B1–C1–C2)–(C2–Si1)	*λ*_max_ b exp/comp	^29^Si^c^(Si1)	^11^B
1A-Xyl	No X-ray structure						1.8	−26.7
1A-*p*Xyl	1.693(2), 1.494(3), 1.398(2), 1.502(3), 1.674(2)	1.830(2), 1.837(2)	2.0633(5), 2.050(1)				3.0	−22.0
1B-Xyl	No X-ray structure						1.0	−26.7
1B-Ph*	1.694(4), 1.496(3), 1.411(3), 1.493(3), 1.702(3)	1.842(2), 1.836(2)	2.072(1), 2.0485(8)				1.5	−25.3
1B-*p*Xyl	No X-ray structure						3.0	−22.4
2A-Me	1.513(1), 1.477(1), 1.371(1), 1.530(1), 1.610(1)	1.7590(9)	2.0772(6), 2.0691(5)	1.947(1)	21°	548/535	−26.2	61.8
2A-Cl	1.491(2), 1.483(2), 1.369(2), 1.536(2), 1.591(2)	1.765(1)	2.0673(6), 2.0667(5)	1.939(1)	21.6°	500/498	−24.8	53.8
2A-Xyl	1.513(3), 1.476(3), 1.368(3), 1.525(3), 1.619(4)	1.773(2)	2.069(1), 2.059(1)	1.957(3)	13.8°	514/497	−23.4	54.7
2B-Me	1.50(1), 1.480(8), 1.37(1), 1.536(9), 1.618(9)^d^	1.753(8)^d^	2.070(2), 2.074(3)^d^	1.931(7)^d^	28.2°^d^	560/511	−28.0	62.9
2B-Cl	1.49(2), 1.48(1), 1.37(1), 1.54(1), 1.63(1) d	1.77(1)^d^	2.072(3)^d^ 2.073(5)^d^	1.926(9)^d^	32.2°/16.4°^d^^f^	500/496	−24.1	52.4
2B-Xyl	No X-ray structure					487	−25.2	57.4
2B-Ph*	1.515(4), 1.481(4), 1.365(4), 1.531(4), 1.616(4)	1.776(3)	2.055(1), 2.072(1)	1.963(3)	9.2°	493/444	—e	—e
3A-*p*Xyl	No X-ray structure					410^g^	−19.9	54.8
3B-*p*Xyl	1.499(3), 1.466(3), 1.355(3), 1.538(3), 1.633(3)	1.765(2)	2.0772(8), 2.0851(9)	1.908(2)	0.2°	415^g^/398	−20.0	54.7
5B-Me	1.537(3), 1.475(4), 1.360(3), 1.539(3), 1.603(4)	1.784(2)		1.975(3)	0°	496/456	226.3	61.3

a In Å, following the numbering scheme shown in Fig. 1 and 2.

b In nm (toluene solutions) – TDDFT calculations using RI-CAM-B3LYP/def2SVP/J.

c In ppm.

d Crystals diffracted poorly and structure could not be determined to allow extensive discussion.

e The species is only present in solution in very small quantities with broad ill-defined spectra potentially due to dynamic equilibria which do not allow unambiguous identification by NMR spectroscopy.

f Two molecules in the asymmetric unit.

g Shoulder.

**Fig. 3:**
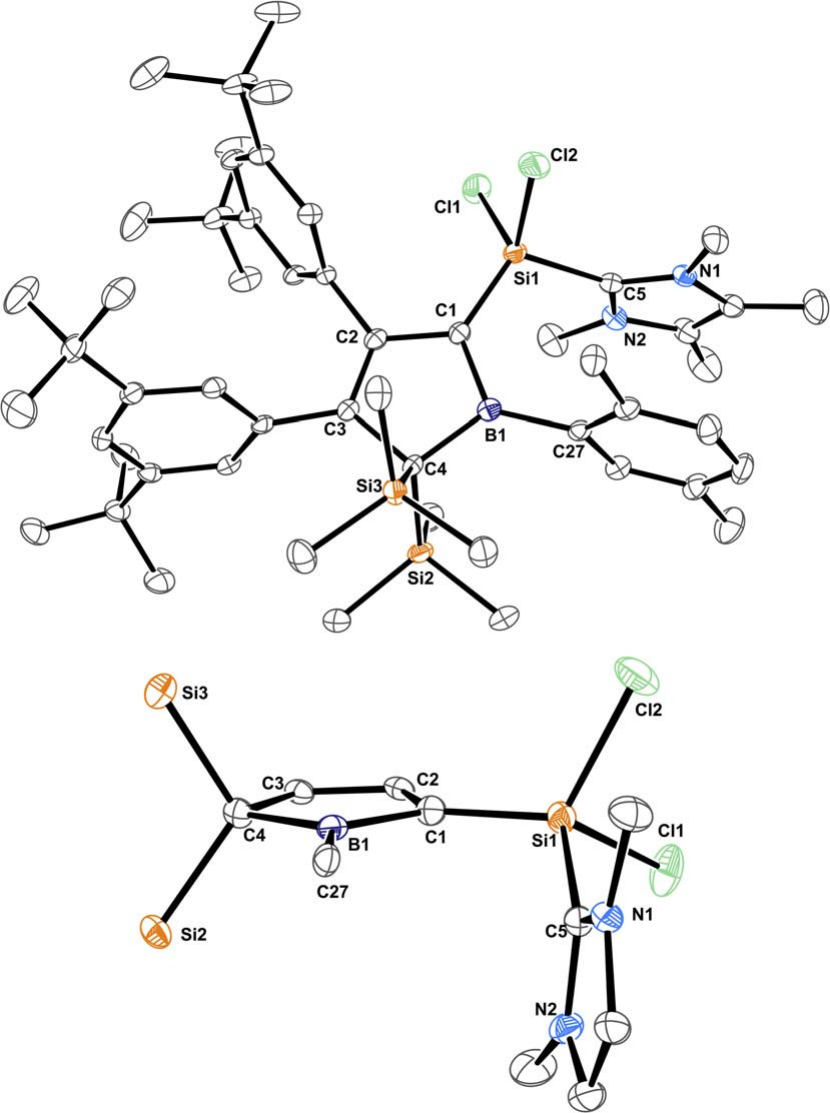
ORTEP of the solid state molecular structure of NHC-supported silylium ylide 3B-*p*Xyl. H-atoms are omitted for the sake of clarity. Anisotropic displacement parameters are drawn at 50% probability. Interatomic distances are listed in Table 1.

The molecular structure of 3B-*p*Xyl is shown in Fig. 3 and details are listed in Table 1. Key difference to IDipp adducts 2 is the lack of distortion of the [SiCl_2_(NHC)]-fragment from the borole plane and a planar C1-atom suggesting that the IDipp sterics impose this pyramidalisation of C1 in derivatives of 2. It is to note that the orientation of the Si–C_NHC_ vector towards the borole-plane differs. In 2 the NHC stands *syn*-periplanar to a lone pair at C1 while in 3B-*p*Xyl a chlorine atom adopts this position.

Trends for NMR spectroscopic observables will be discussed as summary for the two compound classes 1 and 2. Individual details for each compound are listed in Table 1. The most notable NMR spectroscopic property of compound class 1 is the considerably highfield shifted, narrow ^11^B NMR signal (FWHM between *ca.* 350–450 Hz), which is observed at *δ*(^11^B) around −22 ppm. ^11^B NMR signals of 2 are much broader (FWHM *ca.* 1400–1600 Hz). This is a characteristic region (*ca.* −5–−50 ppm) for boron atoms intramolecularly stabilised by π-donor interactions.^36,61,62,68–72^ The ^29^Si-resonance of the bridging SiCl_2_ lies in the regular region around 0 ppm. The bridgehead ^13^C signals are found around 54 ppm and the π-bonded carbon atoms in the backbone of the [2.1.1]-cyclohexene framework are found around 127 ppm.

A very notable feature of the ^1^H-NMR spectra of the B–Cl/Me derivatives of compound class 2 is the time-averaged presence of a mirror-plane symmetry (*e.g.* only one resonance for both SiMe_3_-groups) through the borole-plane, despite the solid-state structure suggests otherwise (Fig. 2). This could be rationalised by either a putative free rotation around the C1–Si1 bond or a dynamic NHC-coordination and dissociation fast enough within the timescale of the NMR experiment. The more bulky B–Ar derivatives (Ar = Xyl, Ph*) of compound class 2 show significantly broadened and seemingly ill-defined signal patterns particularly for the most bulky aryl group Ph*, which indicates presence of reduced symmetry due to a putative sterics-impaired rotation around the C1–Si1 bond. Heating these samples to *ca.* 60–75 °C causes sharpening of these signals.

Compounds 2 reveal a low-field shifted ^11^B-NMR signal around *δ*(^11^B) = 60 ppm. The B-chloro derivatives reveal resonances shifted to lower frequencies (*ca.* 53 ppm) in line with an increased occupation of the empty p-orbital at the boron atom due to π-donation from the chlorine atom or increased C=B π-interaction resulting from higher Lewis-acidity caused by the electron withdrawing chlorine atom. The observed ^11^B resonances lie in between the shift observed for the respective free boroles (*δ*(^11^B) = *ca.* 80 ppm, tricoordinate B-atom) and a more highfield shifted boratafulvene derivative that features authentic B=C double bonds (*δ*(^11^B) = *ca.* 40 ppm). This indicates the B-atom in 2 to be involved in π-bonding to C1 with a partially populated p-orbital, however not to the extent of an authentic double bond.

Spectroscopic characteristics of 3 except for the perceived colour and the respective absorption in the UV-vis spectra barely deviate.

We reason that the mild pyramidalization at C1 stems in part from the localisation of a lone pair of electrons, which is only partially delocalised into the accepting p-orbital at the boron atom to form a B1 = C1 double bond. This is corroborated by the respective notably shortened B–C contact. The lone pair of electrons at C1 is attracted by both π-accepting moieties: the borane as well as the adjacent vinyl moiety. (Allylic) delocalisation into the vinyl moiety explains the rather short C1–C2 contact. Feasible deviation from planarity to account for steric pressure of the C–Si vector is only reasonable if no Si=C double-bond character of the ylide *vs.* ylene is assumed, which is in line with the coordination number at Si. A similar mildly pyramidalised carbon atom with a lone pair of electrons delocalised between two accepting boron-atoms was recently described by Erker.^73^

**Scheme 6:**
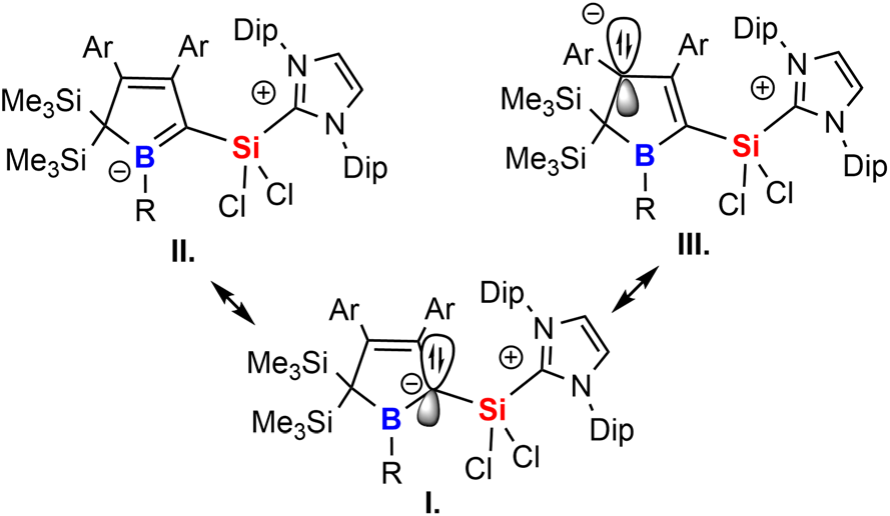
Mesomeric descriptions of NHC-supported silylium ylides.

The experimental properties advocate for a description of 2 as NHC-supported silylium ylide in which the ylide is further internally stabilised by adjacent π-accepting moieties. The cationic charge is mostly localised on the imidazolium moiety. Suitable mesomeric descriptions (I.–III.) for the Lewis-structure of 2 are summarised in Scheme 6. The short Si–C contact is thus a consequence of coulombic attraction rather than π-bonding.

A to some extent comparable electronic situation has been previously described by Berndt and coworkers for their true (*i.e.* unsupported) germene/stannene derivatives of cyclic diboryl-substituted methylene fragments or respective phosphine adducts.^74–78^

A somewhat related 1,3-silyl migration was described by Erker and coworkers who noted the formation of an unusual ketene derivative from reaction of 2,5-disilylboroles with carbon monoxide.^36^ However, compared to the essentially purely σ-donating SiCl_2_(IDipp), CO is a potent π-acceptor readily forming an electronically trivial, authentic C=C double-bond.

### Electronic structure of silylium ylides 2

To further elucidate the electronic structure of the silylium-ylides 2, these structures were probed computationally using DFT.^79,80^ Structure optimisation with RI-BP86-D3(BJ)/def2-TZVP model chemistry reproduced the experimentally found features very well, including the deviation from planarity at C1.^81–85^

**Fig. 4:**
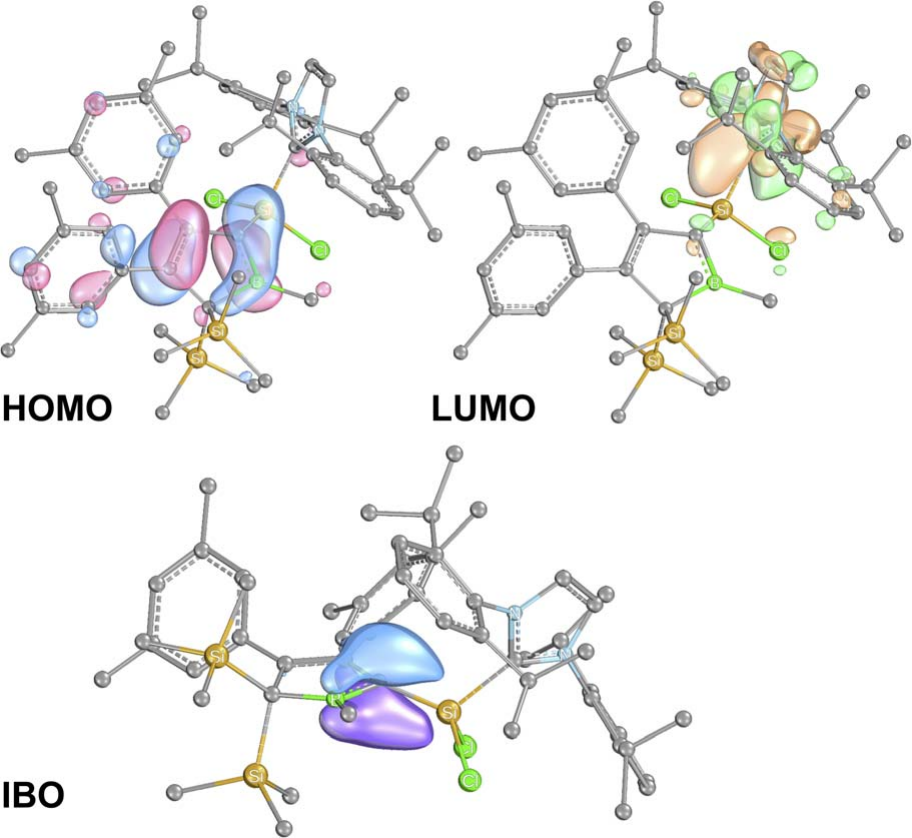
Frontier molecular orbitals of 2A-Me. (BP86/def2-TZVP). Iso-surfaces at 60% probability.

The results for 2A-Me are presented and discussed exemplarily for all experimentally accessed derivatives as deviations are only marginal. NBO analyses^86,87^ on 2A-Me corroborated electronic structure II. (Scheme 6) with a cyclic borata-butadiene moiety as the leading Lewis-structure. However, the C=B double bond is strongly polarised towards the C-atom, both in its σ- (72%) and even more in its π-contribution (82%), the latter NBO being only occupied by 1.6 electrons. Second order perturbation theory finds no significant delocalisation toward the Si-atom (a potential silene resonance structure) but a strong (28 kcal mol^−1^) delocalisation of the C=B π-electron density into the π*-orbital of the C=C doublebond in the boratabutadiene moiety. This is in line with an allyl-type delocalisation of a C1-centered lone pair of electrons (as in resonance structure III., Scheme 6) in line with structural parameters. The respective intrinsic bonding orbital (IBO)^88^ essentially represents a lone pair of electrons in a C1-centered p-orbital mildly polarised into the three adjacent atoms (Fig. 4).

The canonical frontier molecular orbitals reveal the boratabutadiene π-system to be the HOMO with the LUMO located on the imidazolium NCN π*-orbital with a fairly small energy gap (0.86 eV for 2A-Me, Fig. 4). The intense red to purple colour observed for these NHC-supported silylium ylides 2 goes back to respective π/π* HOMO to LUMO transitions as corroborated by TD-DFT calculations that reproduce these electronic spectra in the Vis range. An alternative description of compounds 2 other than NHC-supported silylium ylides would be an internally π-accepting boryl-substituted carbene fragment that is stabilized by a nucleophilic silylene donor.

Contrary to conventional widely applied nucleophilic carbenes such as NHC, this putative intermediate carbene fragment would be electrophilic an thus readily stabilised by the nucleophilic [:SiCl_2_(IDipp)] donor. Similar descriptions were previously suggested by Berndt.^78^*In situ* generation of a σ-donating cAAC by 1,2-*H* migration in a cyclic alkene induced by a borane was observed previously by Kinjo and coworkers for a 2,3-dihydro-1*H*-1,2-azaborole derivative to finally form an adduct of the carbene to the borane.^89^

### Reduction of compounds 1 and 2

Our initial investigations aimed at more efficient approaches toward the synthesis of neutral Si(ii) half-sandwich compounds (such as 4B-Ph*, Scheme 1) circumventing the necessity of two equivalents of [Cp*Si]^+^ reagents.^41^ We therefore attempted the reduction of the now straightforwardly available dichlorosilylene addition derivatives 1 and 2.

While our attempts to achieve successful reductions of 1 with common reducing agents (Li(naph), 5% Na on NaCl, KC_8_) revealed only unsatisfyingly poor selectivity, a rather clean (as monitored by NMR spectroscopy) reliable conversion was achieved by 1 equiv. of Jones' and Stasch's [^Mes^Nacnac(Mg^I^)]_2_ reagent.^90,91^ Starting from 1B-Ph* we were able to selectively form the previously described Si(ii) borole compound 4B-Ph*. The reverse reaction, an oxidative conversion of the half-sandwich to a bicyclic 5-sila-6-borabicyclo[2.1.1]hex-2-ene was previously observed by formal protonation of the Si-cluster 4B-Ph*.^41^

**Scheme 7:**
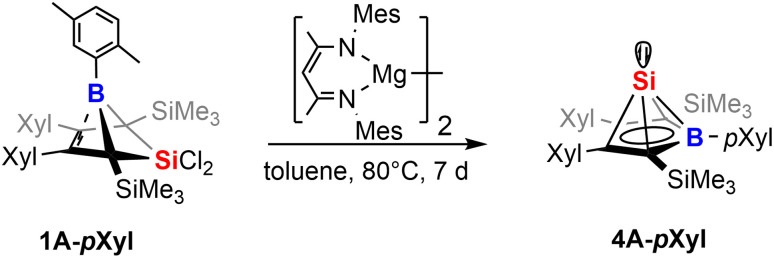
Reduction of 1 to give Si(ii) half-sandwich compounds.

**Fig. 5:**
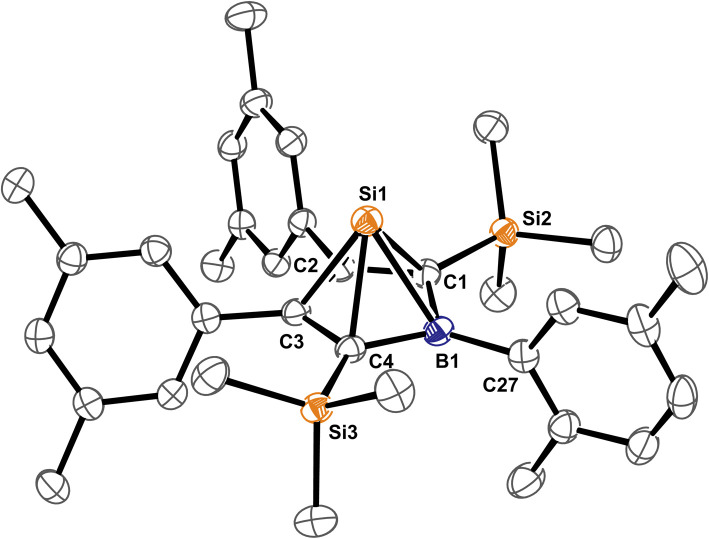
ORTEP of the solid state molecular structure of Si(ii) borole half-sandwich compound 4A-*p*Xyl. H-atoms are omitted for the sake of clarity. Anisotropic displacement parameters are drawn at 50% probability. Selected interatomic distances [Å]: B1–C1 1.549(3), C1–C2 1.462(3), C2–C3 1.429(3), C3–C4 1.460(3), C4–B1 1.547(3), B1–Si1 2.190(2), C1–Si1 2.100(2), C2–Si1 2.094(2), C3–Si1 2.109(2), C4–Si1 2.102(2). (BC_4_-borole)_centr_–Si1 1.700.

Accordingly, we converted 1A-*p*Xyl into the respective new Si(ii) cluster 4A-*p*Xyl by reduction with 1 equiv. of [^Mes^Nacnac(Mg^I^)]_2_ reagent (Scheme 7). NMR monitoring suggested clean conversions, however due to the high solubility of the resulting clusters, poor isolated yields of crystallized compounds 4 (*ca.* 20%) were obtained. The Si(ii) cluster 4A-*p*Xyl was obtained as a colorless compound revealing a mixture of two conformers (with regards to rotation around the B-*p*Xyl bond) in a ratio of *ca.* 15 : 85 as observed by NMR spectroscopy. The X-ray structure of 4A-*p*Xyl is shown in Fig. 5 and only reveals the *trans*-type conformer where the apical Si-atom and the *ortho*-methyl group occupy different half-spheres of the borole plane. The key bond lengths of the *nido*-clusters [C_4_B]Si moiety are, within the error, identical to those previously described for 4B-Ph* and feature the essential bond length homologisation that is to be expected when a free borole (with localized single- and double bonds) is reduced to a borole dianion, isoelectronic to Cp^−^.

The ^11^B resonance is found at *δ*(^11^B) = 30.6 ppm (31.5 ppm, in 4B-Ph*) and the characteristically high field shifted ^29^Si resonances are found at *δ*(^29^Si) = −354.8 ppm (minor conformer) and −355.7 ppm (major conformer). The latter also clearly reveals the lowfield-shifted shoulder at −355.6 ppm assigned to the ^10^B–^29^Si isotopologue.

**Scheme 8:**
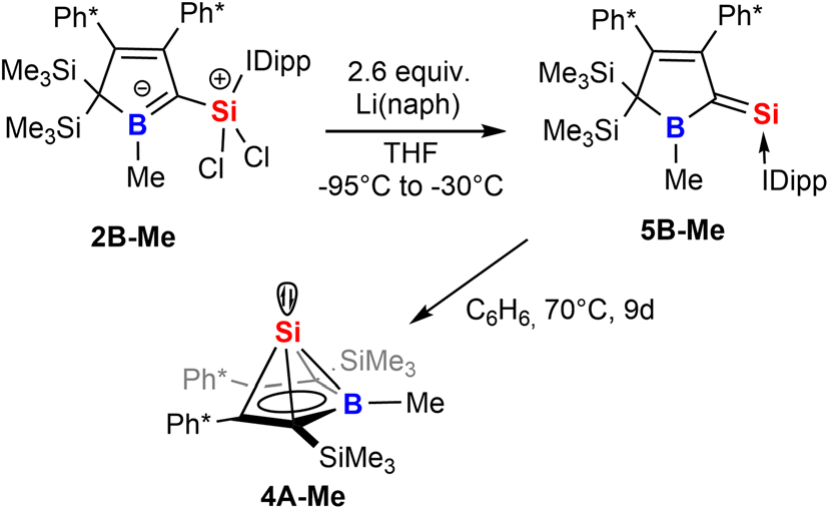
Reduction of 2 to give an NHC-supported silavinylidene 5 that can be thermally converted to a stable Si(ii) half-sandwich compound.

Strikingly, when we attempted the reduction of derivatives of 2 we found rather unselective formations of intractable mixtures however, for 2B-Me a rather clean reduction was observed with lithium naphthalenide (Scheme 8).

**Fig. 6:**
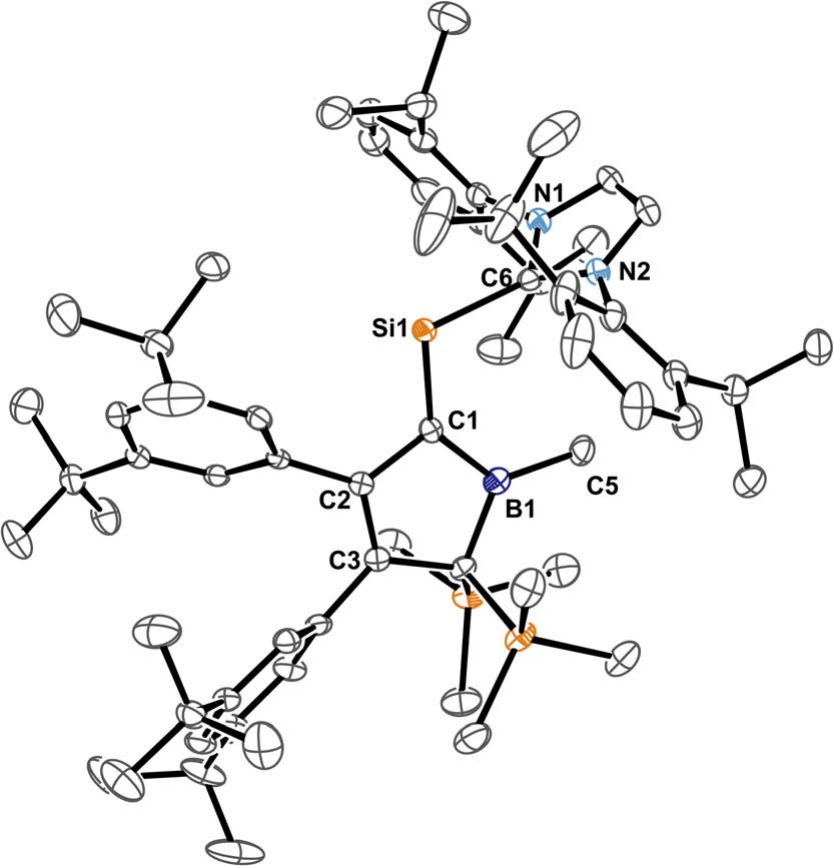
ORTEP of the solid state molecular structure of NHC-supported silavinylidene 5B-Me. H-atoms and partially occupied ether molecule are omitted for the sake of clarity. Anisotropic displacement parameters are drawn at 50% probability. Details on interatomic distances are summarized in Table 1.

Carefully allowing to warm the coloured reaction mixture from −95 °C to *ca.* −30 °C led to an intensely blood red colourisation and we were able to isolate the unprecedented NHC-supported silavinylidene 5B-Me featuring an authentic C=Si: double bond. A number of donor-stabilised heavier homologs of vinylidenes have been reported in the last decade (R_2_Ge = Ge*,^92,93^ R_2_Si = Ge*,^94,95^ R_2_Ge = Si*,^96^ R_2_Si = Si*;^97,98^ asterisks denote further donor-stabilisation of the atom). To the best of our knowledge, silavinylidene 5B-Me is the first example for the lightest heavier congener of (donor-stabilised) vinylidenes. Filippou recently reported a NHC- and isonitrile donor-stabilised Si^0^ atom which reveals the structural motif of a NHC-supported :Si=CNR fragment.^99^ The molecular structure of 5B-Me is shown in Fig. 6.

**Fig. 7:**
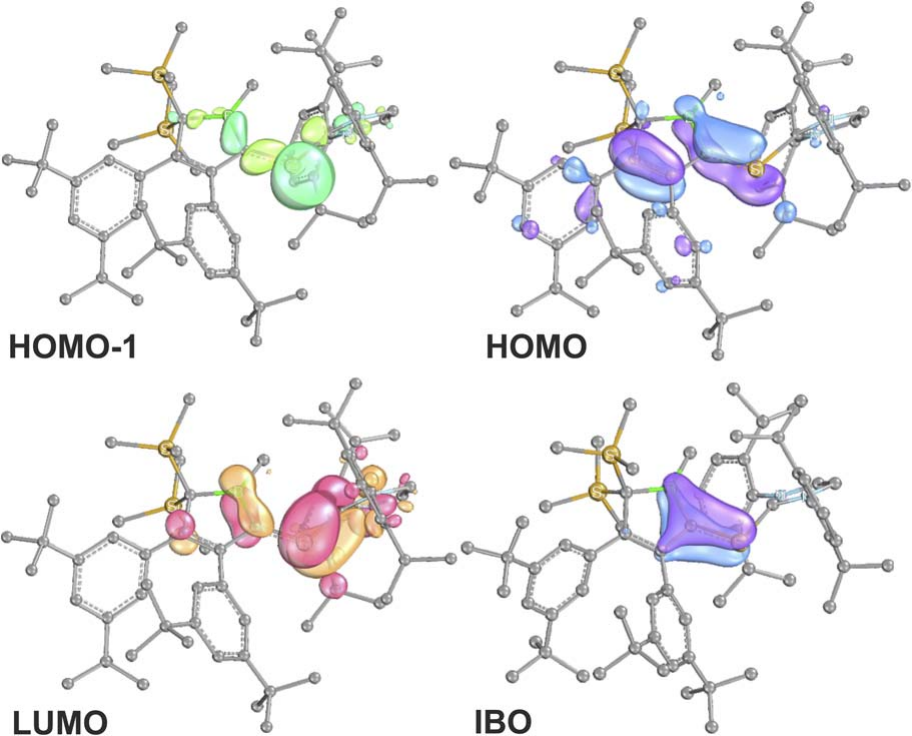
Frontier molecular orbitals of 5B-Me (BP86/def2-TZVP). Iso-surfaces at 60% probability.

Direct comparison of bond length in 5B-Me with the set of structural features of the silylium ylides reveals only minor changes with a slight elongation of the C1–B1, C1–Si1 and Si1–C_NHC_ distances being most notable. The elongation of the Si1 distances may be a result of the larger covalent radius of the reduced Si-atom. The Si1=C1 distance found is identical to what Filippou reported.^99^ The C1–B1 elongation may hint at a reduced π-interaction of the formerly ylidic lone-pair with the boron atom as π-bonding to the Si1 atom can now be established. The ^11^B-NMR signal of 5B-Me is found at *δ*(^11^B) = 61.3 ppm, barely shifted from 2B-Me. The structural data are in line with a Lewis-structure as depicted in Scheme 8 which is also corroborated by NBO analysis. The comparably short C1–C2 and C1–B1 can be rationalised with some delocalisation of the C1=Si1 π-density into the adjacent accepting vinyl and boryl moieties. This is corroborated by second order perturbation theory in NBO that finds donation from the C1=Si1 NBO (populated by 1.58*e*) into the boron p-orbital (11 kcal mol^−1^) and the C3=C4 π*-NBO (29 kcal mol^−1^). The lone-pair at Si is predicted to be high in s-orbital character (72%). The canonical HOMO-1 represents the Si-based lone-pair, while the HOMO is located on the silabutadiene moiety. The LUMO is a π-type orbital on the Si–NHC-vector (Fig. 7).

The most obvious change is observed for the ^29^Si-resonance that shifts drastically to higher frequencies and is found at *δ*(^29^Si) = 226.3 ppm. The value is reproduced by computational predictions (GIAO:PBE0/def2-TZVP *δ*(^29^Si)_calc_ = 228 ppm). This value deviates drastically from other silavinylidene derivatives such as Filippou's reported value for (NHC): Si = CNR (*δ*(^29^Si) = −142 ppm)^99^ or Wesemann's germasila vinylidene (*δ*(^29^Si) = −48 ppm)^96^ which have been discussed as masked Si^0^ compounds and is closer to Robinson's (IDipp)Si=Si(IDipp) (*δ*(^29^Si) = 225 ppm)^54^ or Filippou's (IDipp)Si = PMes* (*δ*(^29^Si) = 267 ppm).^100^ The intensely blood-red 5B-Me reveals a respective absorption band at 496 nm with a shoulder at 436 nm.

When solutions of 5B-Me were heated at *ca.* 70 °C for several days, we observed a very clean and complete conversion of the deep red silavinylidene to the colourless Si(ii) half-sandwich compound 4B-Me and free IDipp which becomes unequivocally clear from the respective characteristic heteronuclear NMR pattern with *δ*(^29^Si) = −347.6 ppm and *δ*(^11^B) = 31.4 ppm. The isomerisation of NHC-supported silavinylidene 5B-Me to 4B-Me and IDipp was computationally approximated to be endergonic at 298 K (4.3 kcal mol^−1^). The conversion of the silavinylidene 5 into the half-sandwich compounds including a remigration of the silyl groups further highlights the mobility of silicon atoms on the borole-platform. Further inspection revealed that the half-sandwich compound was also present as a minor side product in the crude reduction reaction mixture of 5.

Therefore, reductions of both products of the reaction of free boroles with SiCl_2_(IDipp) ultimately allow access to the Si(ii)-centered, nucleophilic *nido*-cluster 4 allowing for the development of more straightforward synthetic procedures than introducing Si(ii) *via* [Cp*Si]^+^.

## Conclusions

This study gives a detailed account on the reactivity of *anti*aromatic free boroles with SiCl_2_(IDipp) as a low-valent silicon precursor. They either react in a formal [4 + 1] cycloaddition reaction to give sila-borabicyclo[2.1.1]hex-2-ene derivatives or induce silyl migration to generate NHC-supported silylium ylides. Mechanistic proposals corroborated by computational assessment and experimental derivatizations have been presented for the diverging product formation pathways. We have been able to show, that the borole platform allows for facile and reversible migration of silyl groups. Depending on the substitution pattern and reaction conditions selective formation of either of these products was achieved. It was shown that the NHC-supported silylium ylides can be thermally converted into the bicyclic products but addition of small NHC to the bicycles can induce trimethyl silyl migration leading to new NHC-supported silylium ylides. Finally examples for both species, sila-borabicyclo[2.1.1]hex-2-ene and NHC-supported silylium ylides were successfully reduced to borole-based half-sandwich compounds of Si(ii), our initial target structure. In the case of the reduction of silylium ylides, an unprecedented silavinylidene was isolated and identified as a key intermediate in the formation of the Si(ii) half-sandwich cluster. This study perfectly complements to most recent findings by the groups of Nakamoto and Scheschkewitz that found virtually identical silyl migration in *anti*-aromatic cyclobutadienes by addition of nucleophilic silylenes to give donor-supported silenes.^39^ Reduction of such silenes gave Si(ii) *nido*-clusters (silapyramidanes). Our work suggests that silavinylidenes could also be relevant intermediates in the formation of silapyramidanes.

## Data availability

Synthetic details and analytical data, including depictions of all spectra and detailed accounts on the methods applied are documented in the ESI.† Crystallographic data is made available *via* the CCDC. Coordinate data of all computationally optimised species is provided as a separate xyz-file as accompanying ESI.†

## Author contributions

JS, TH, LN and MK conducted and validated the experiments. TH, JS and CPS conceptualised the project. PNR and CPS acquired and analysed X-ray data. DS and CPS acquired funding and provided resources and supervision. CPS managed the project, performed computations, wrote the original draft. The manuscript was further reviewed and edited from contributions of JS, TH and PNR.

## Conflicts of interest

There are no conflicts to declare.

## Supplementary Material

SC-014-D3SC00808H-s001

SC-014-D3SC00808H-s002
