# Supplementary material for: A donor-supported silavinylidene and silylium ylides: boroles as a flexible platform for versatile Si(ii) chemistry
Source: Chem Sci. 2023 Mar 31;14(19):5148–59. doi: 10.1039/d3sc00808h (PMC10189903; doi:10.1039/d3sc00808h)
Supplement: SC-014-D3SC00808H-s001 [file SC-014-D3SC00808H-s001.pdf]

Electronic Supplementary Material (ESI) for Chemical Science.
This journal is © The Royal Society of Chemistry 2023

Supporting Information

A Donor-Supported Silavinylidene and Silylium Ylides: Boroles as a Flexible Platform for Versatile Si(II) Chemistry

Julijan Sarcevic†, Tobias Heitkemper†, Paul Niklas Ruth, Leonard Naß, Maximilian Kubis, Dietmar Stalke and Christian P. Sindlinger*

Institut für Anorganische Chemie, Universität Stuttgart, Pfaffenwaldring 55, 70569 Stuttgart.

Institut für Anorganische Chemie, Georg-August Universität Göttingen, Tammannstr. 4, 37077 Göttingen.

Institut für Anorganische Chemie, RWTH Aachen University, Landoltweg 1a, 52074 Aachen.

Table of Contents

Experimental Details 4
- Nomenclature 4
- General Information 4
- UVVis Spectroscopy 4
- Mass spectrometry 4
- NMR spectroscopy 4
- Starting materials and reagents 5

Synthesis and Analytical Data 6
- Compound A-Me 6
- Analytical Data for Compound A-Me 6
- Spectra Plots for Compound A-Me 7
- Crystal structure of Compound A-Me 8
- Compound A-Xyl 10
- Analytical Data for Compound A-Xyl 10
- Crystal structure of Compound A-Xyl 11
- Spectra Plots for Compound A-Xyl 12
- Compound A-*p*Xyl 15
- Analytical Data for Compound A- *p*Xyl 15
- Spectra Plots for Compound A-*p*Xyl 16
- Compound B-Xyl 19
- Analytical Data for Compound B-Xyl 19
- Spectra Plots for Compound B-Xyl 20
- Compound B-*p*Xyl 22
- Analytical Data for Compound B-*p*Xyl 22
- Crystal structure of Compound B-*p*Xyl 23
- Spectra Plots for Compound B-*p*Xyl 24
- Compound 1a-Xyl 26
- Analytical Data for Compound 1a-Xyl 26
- Spectra Plots for Compound 1a-Xyl 27
- Compound 1a-*p*Xyl 29
- Analytical Data for Compound 1a-*p*Xyl 29
- Crystal structure of Compound 1a-*p*Xyl 30
- Spectra Plots for Compound 1a-*p*Xyl 31

Compound 1b-Xyl 34
Analytical Data for Compound 1b-Xyl 34
Spectra Plots for Compound 1b-Xyl 35
Compound 1b-pXyl 37
Analytical Data for Compound 1b-*p*Xyl 37
Spectra Plots for Compound 1b-*p*Xyl 38
Compounds 1b-Ph* and 2b-Ph* 40
Analytical Data for Compound 1b-Ph* 40
Crystal structure of Compound 1b-Ph* and 2b-Ph* 41
Spectra Plots for Compound 1b-Ph* 42
Compound 2a-Me 44
Analytical Data for Compound 2a-Me 44
Crystal structure of Compound 2a-Me 45
Spectra Plots for Compound 2a-Me 46
Compound 2a-Cl 49
Analytical Data for Compound 2a-Cl 49
Crystal structure of Compound 2a-Cl 50
Spectra Plots for Compound 2a-Cl 51
Compound 2a-Xyl 53
Analytical Data for Compound 2a-Xyl 53
Crystal structure of Compound 2a-Xyl 54
Spectra Plots for Compound 2a-Xyl 55
Compound 2b-Me 58
Analytical Data for Compound 2b-Me 58
Crystal structure of Compound 2b-Me 59
Spectra Plots for Compound 2b-Me 60
Compound 2b-Cl 62
Analytical Data for Compound 2b-Cl 62
Crystal structure of Compound 2b-Cl 63
Spectra Plots for Compound 2b-Cl 64
Compound 2b-Xyl 66
Analytical Data for Compound 2b-Xyl 66
Spectra Plots for Compound 2b-Xyl (RT) and mixtures of 2b-Xyl and 1b-Xyl (70°C) 67
Compound 3a-*p*Xyl 71
Analytical Data for Compound 3a-*p*Xyl 71
Spectra Plots for Compound 3a-*p*Xyl 72
Compound 3b-*p*Xyl 74
Analytical Data for Compound 3b-*p*Xyl 74
Crystal structure of Compound 3b-*p*Xyl 75
Spectra Plots for Compound 3b-*p*Xyl 76
Compound 4a-*p*Xyl 79
Analytical Data for Compound 4a-*p*Xyl 79
Crystal structure of Compound 4a-*p*Xyl 80
Spectra Plots for Compound 4a-*p*Xyl 81

Compound 5b-Me and 4b-Me 84
Analytical Data for Silavinylidene 5b-Me 84
Analytical Data for half-sandwich cluster 4b-Me 85
Crystal structure of Compound 5b-Me 85
Spectra Plots for Compound 5b-Me 86
Spectra Plots for Compound 4b-Me 88
Compound 4b-Ph* 90
UV*vis* Spectra Plots 91
Free Boroles A-Me, A-Xyl, A-*p*Xyl, B-Xyl, B-*p*Xyl 91
NHC-supported Silylium Ylides Compounds 2 91
NHC-supported Silavinylidene 5b-Me 91
Crystallographic Details 92
General Data Acquisition and Processing 92
Tabulated Crystallographic Details 93
Computational Details 94
Structure Optimisation, Frequency Calculation and Thermochemical Approximations 94
NBO Analyses 94
TD-DFT 94
Comment on the Computationally Assessed Mechanistic Proposal 97
Literature 99

Experimental Details

Internal Nomenclature

Please note that in this text, that due to the nature of possible resonance structure descriptions of compound class 2 these compounds will be either referred to as (NHC)-adducts to silenes (assuming a C=Si double bond) or NHC-supported silylium ylides (C^{-}-$Si^{+}Cl_2$(NHC)). Compound descriptors with capitalized or lowercase letter are equivalent e.g. **1A-Cl ≡ 1a-Cl.**

General Information

All manipulations requiring handling under inert conditions were carried out under argon atmosphere using standard Schlenk techniques or an MBraun Glovebox with an Ar atmosphere. Benzene was obtained from an MBraun SPS or distilled from sodium and stored over molecular sieves, toluene and ether were distilled from sodium. Fluorobenzene was dried over P_2O_5, and stored over molecular sieves (4 Å). Dichloromethane was distilled from CaH_2. Hexane and pentane were distilled from Na/K alloy. THF was distilled from potassium. Dichloromethane-*d₂* was distilled from CaH_2, THF-d_8 was dried over $LiAlD_4$ and vacuum transferred, benzene-d_6 was distilled from potassium, and solvents were degassed and stored in a glove box. All solvents were routinely degassed three times using freeze-pump-thaw cycles.

Elemental analyses were performed by the Analytical Facilities at the Institute of Inorganic Chemistry, University of Göttingen and Institute of Organic Chemistry, RWTH Aachen University.

UVVis Spectroscopy

UVVis spectra were measured on a Perkin Elmer Lambda 25 nm spectrometer in Quartz cuvettes fitted with a Yound-type teflon valve. Normalized plots of the s UV*vis* spectra are given below.

Mass spectrometry

Mass spectra were recorded by the Zentrale Analytik within the Faculty of Chemistry, Göttingen applying a Liquid Injection Field Desorption Ionisation-technique on a JEOL accuTOF instrument with an inert-sample application setup under argon atmosphere. The injection capillary was washed several times with dry, distilled and inertly injected toluene before the samples were injected. Samples usually had a concentration of 1 – 2 mmol/L in toluene with a minimum amount of fluorobenzene added to guarantee solubility and were prepared in a glovebox.

NMR spectroscopy

NMR spectra were recorded with either a Bruker Avance III 400 NMR spectrometer equipped with a 5 mm BBFO ATM probe head and operating at 400.13 (^{1}H), 100.61 (^{13}C), 128.38 (^{11}B) and 79.46 MHz (^{29}Si) along with a variable temperature set-up or a Bruker Avance Neo 400 NMR spectrometer with a CryoProbeProdigy BB ATM probe head operating at 400.25 MHz (^{1}H) and 100.65 MHz (^{13}C) or a Bruker AVIII HD 500 NMR spectrometer with a CryoProbeProdigy ATM probe head and operating at 500.25 (^{1}H), 125.80 (^{13}C), 160.50 MHz (^{11}B) and 99.37 MHz (^{29}Si) or a Bruker Avance III HD spectrometer operating at 116.64 MHz (^{7}Li) or a Bruker Avance II NMR spectrometer operating at 400.13 (^{1}H), 100.62 (^{13}C), 128.38 (^{11}B), 79.46 MHz (^{29}Si) or a Bruker Avance III NMR spectrometer operating at 400.13 (^{1}H), 100.62 (^{13}C), 128.38 (^{11}B), 79.46 MHz (^{29}Si) Chemical shifts are reported in δ values in ppm relative to external Me_4Si and, if not otherwise stated, referenced using the chemical shift of the solvent ^{2}H lock resonance frequency and Ξ = 19.867187% for ^{29}Si, Ξ = 38.863797 % for ^{7}Li, Ξ = 32.083974% for ^{11}B, and Ξ = 94.094011 % for ^{19}F.[1] ^{1}H and ^{13}C spectra have been referenced on specific values for the respective solvent signal. The proton and carbon signals were assigned where possible via a detailed analysis of ^{1}H, ^{13}C, ^{1}H-^{1}H COSY, ^{1}H-^{1}H NOESY, ^{1}H-^{13}C HSQC, ^{1}H-^{13}C HMBC NMR spectra.

Young-type teflon-valve borosilicate NMR tubes have been used throughout the study.

Starting materials and reagents

3,4-(2',5'-$Me_2(C_6H_3)$)-2,5-($SiMe_3$)-borole derivatives **A-Cl**, **A-Me**, **A-Ar** (Ar =*p*-Xyl, *m*-Xyl, Ph*; *p*-Xyl = 2,5-$Me_2(C_6H_3)$, *m*-Xyl = 3,5-$Me_2(C_6H_3)$, Ph* = 3,5-*t*$Bu_2(C_6H_3)$) were prepared as recently reported in the respective references or analogous modifications of the reported protocols.[2, 3]

3,4-(3',5'-*t*$Bu_2(C_6H_3)$)-2,5-($SiMe_3$)-borole derivatives **B-Cl**,[2] **B-Me**,[4] **B-Ar**[2] (Ar =*p*-Xyl, *m*-Xyl, Ph*; *p*-Xyl = 2,5-$Me_2(C_6H_3)$, *m*-Xyl = 3,5-$Me_2(C_6H_3)$, Ph* = 3,5-*t*$Bu_2(C_6H_3)$) and $[Li(OEt_2)]_2$[**B-Ph***][5] were prepared as recently reported in the respective references or analogous modifications thereof.

IDipp,[6] IDipp($SiCl_2$),[7] 1,3,4,5-Tetramethylimidazol-2-ylidene[8], $[^{Mes}(nacnac)Mg^{I}]_2$[9] were prepared as previously described in the literature. $ArBCl_2$ (Ar = Xyl, *p*Xyl) were prepared analogously to our previously reported procedure for Ph*BCl_2.[2]

Synthesis and Analytical Data

Compound A-Me

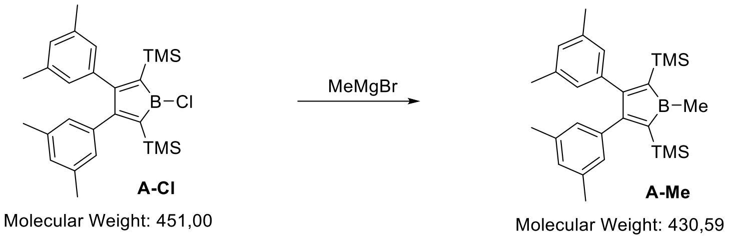

In a glove box, a solution of MeMgBr in diethylether (0.62 M, 0.19 mL, 0.12 mmol, 1 eq.) was added to a solution of chloroborole **A-Cl** (53.7 mg, 0.119 mmol, 1 eq.) in diethylether (5 mL). The resulting suspension was stirred at room temperature for 1 h and the solvent was then removed under reduced pressure. The orange solid was suspended in hexane (2 ml), filtered through a syringe filter equipped with a thin plug of glass fiber and the residue was subsequently washed with hexane (1 mL). The hexane was thereafter removed from the orange filtrate, to yield **A-Me** as an orange, crystalline solid (50.4 mg,l 0.117 mmol, 98 %).

Note: For clean synthesis it is important to accurately determine the concentration of the Grignard-solution and careful measure the added amount of the reagent as even a slight excess will lead to unwanted byproducts. For convenience, we diluted commercially available stock solutions and titrated each batch of the Grignard-reagent intended prior to its use.

Analytical Data for Compound A-Me

NMR:

^{1}H (300.13 MHz, 298 K, C_6D_6, C_6D_5H at 7.15 ppm): 6.63 (m, 4H, *o-H*), 6.57 (m, 2H, *p-H*), 1.98 (m, 12H, *m*-C*H*$_3$), 1.30 (s, 3H, B-C*H*$_3$) 0.09 (s, 18H, TMS). (multiplets could not be resolved).

^{13}C{^{1}H} (100.64 MHz, 298 K, C_6D_6 solvent signal at 128.0 ppm): 178.7 (C_β), 140.2 (*ipso*-C_{ar}), 139.9 (C_α), 136.5 (*m*-C_{ar}), 129.0 (*p*-C_{ar}), 126.3 (*o*-C_{ar}), 21.1 (*m*-*C*H$_3$), 11.7 (B-*C*H$_3$) 1.3 (TMS).

^{11}B (128.37 MHz, 298 K, C_6D_6): 81.2 ($\omega_{1/2}$ = 1255 Hz).

^{29}Si-INEPT (79.49 MHz, 298 K, C_6D_6): –9.7.

Elemental Analysis: ($C_{27}H_{39}BSi_2$) calcd C 75.31, H 9.13, B 2.51, Si 13.04, observed C 72.72, H 8.83. (best analysis obtained)

LIFDI-MS: calcd exact mass: 430.27 m/z, observed m/z: 431.4 $[M+H]^+$.

UV-Vis: λ_{max} 451 nm ($\varepsilon_{451} \approx 400$ L mol $^{-1}$ cm^{-1})

Spectra Plots for Compound A-Me

1H-NMR-spectrum of methylborole **A-Me** in C6D6
C6D5H at 7.15 ppm

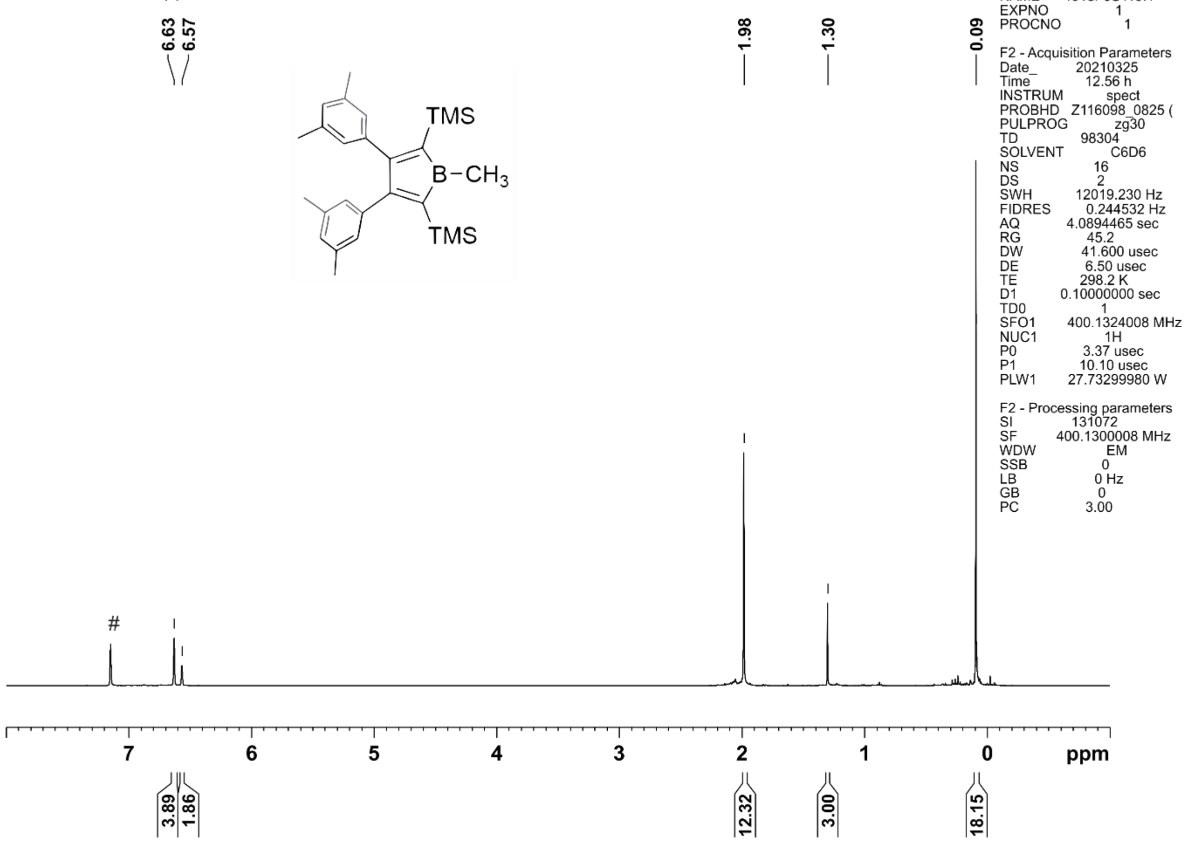

13C{1H}-NMR-spectrum of methylborole **A-Cl** in C6D6
C6D6 at 128.0 ppm

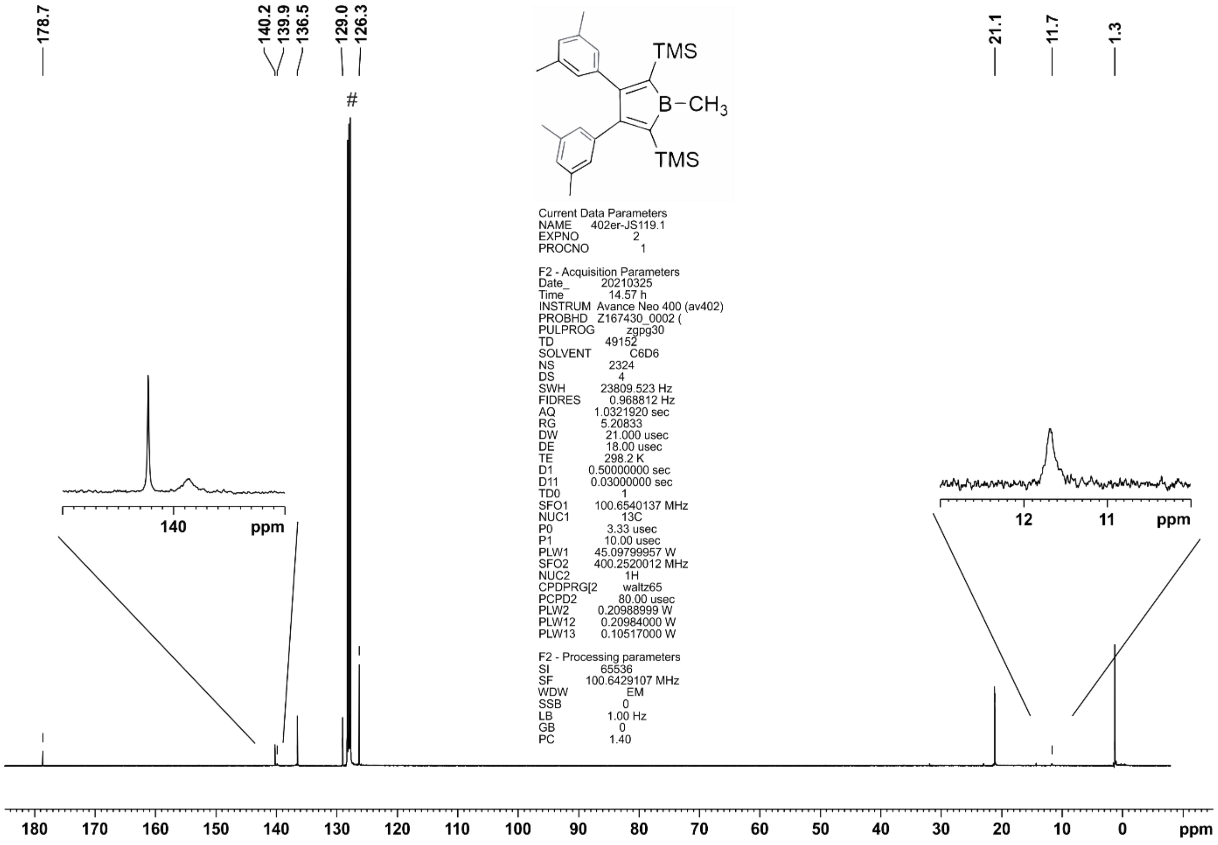

11B-NMR spectrum (background suppressed) of methylborole **A-Me** in C6D6

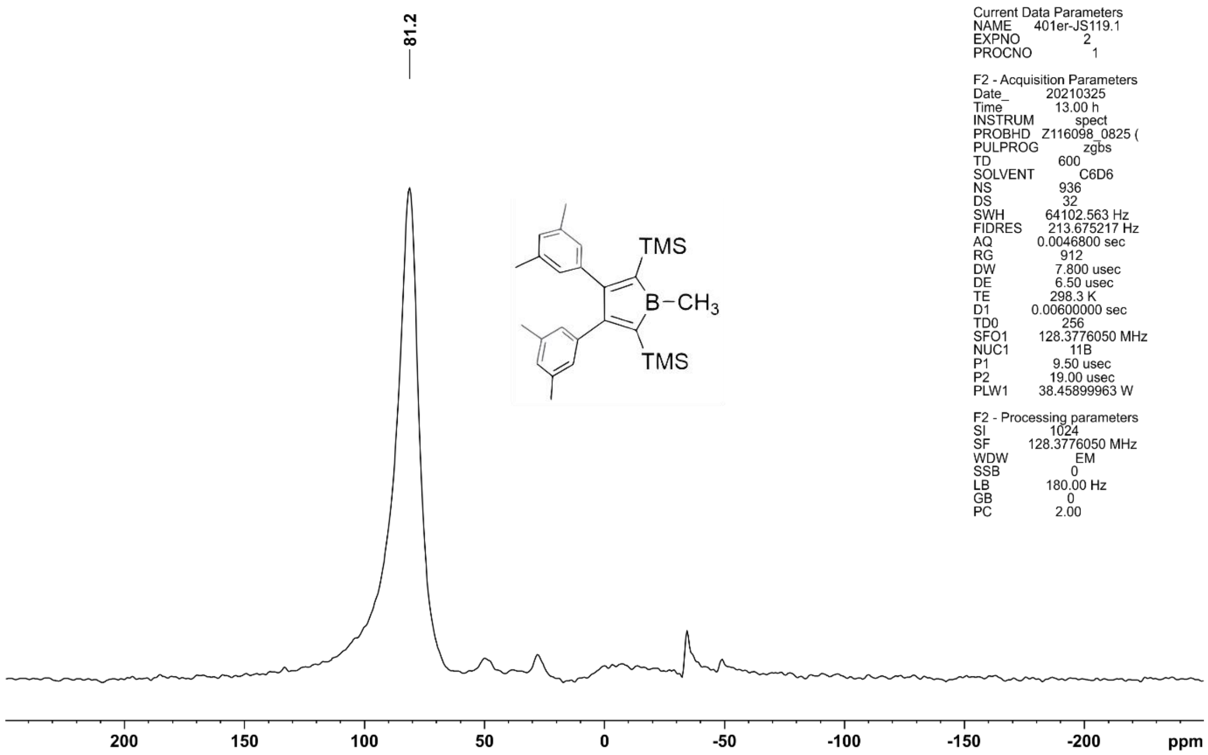

29Si-INEPT-NMR spectrum of methylborole **A-Me** in C6D6

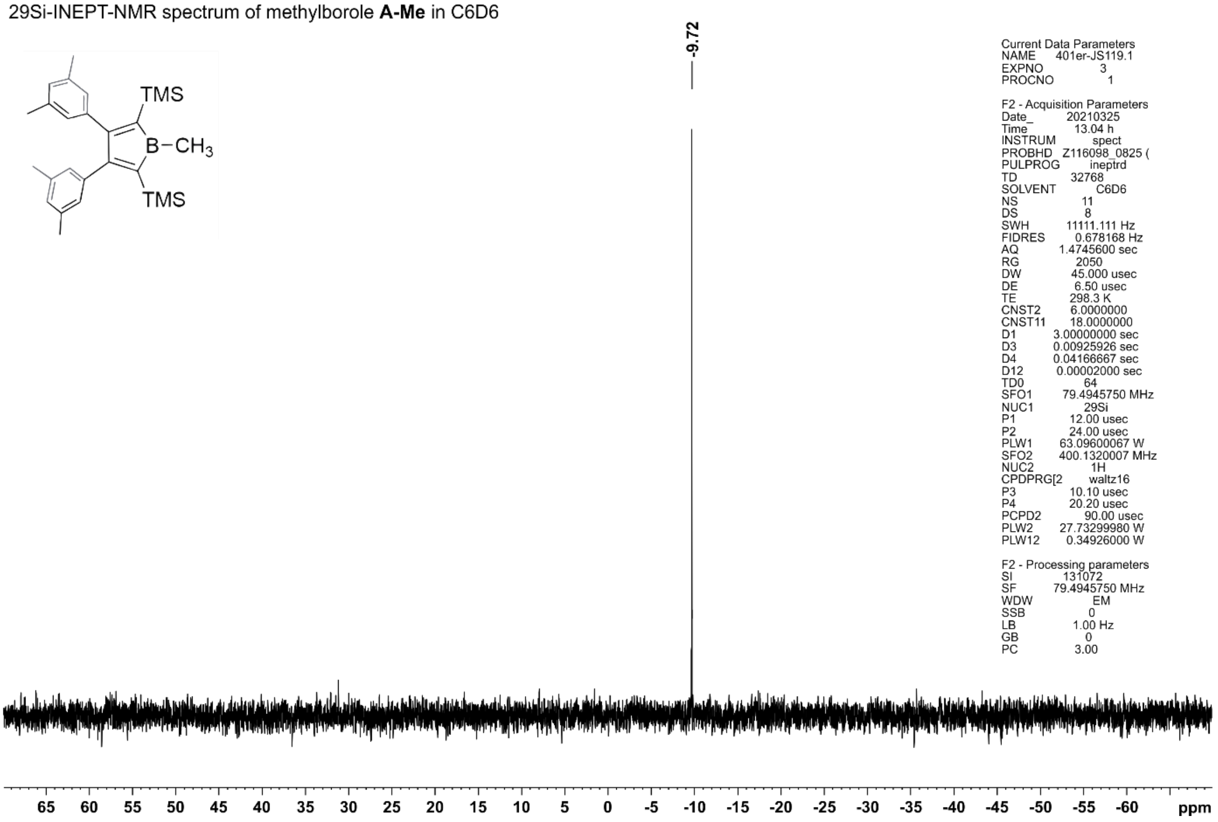

Crystal structure of Compound A-Me

For further details on the diffraction measurement please see the respective section.

A-Me crystallised from solutions in hexane in a freezer (-40°C).

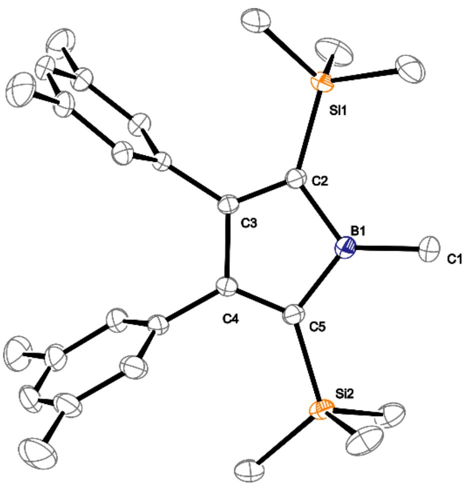

ORTEP plot of the molecular structure of **A-Me**. Atomic displacement parameters are drawn at 50% probability level. Hydrogen atoms are omitted for the sake of clarity. Selected bond length in Å: B1-C1 1.594(2), B-C2 1.594(2), C2-C3 1.357(2), C3-C4 1.536(1), C4-C5 1.358(2), C5-B1 1.593(2), The structure was deposited with the CCSD.

Compound A-Xyl

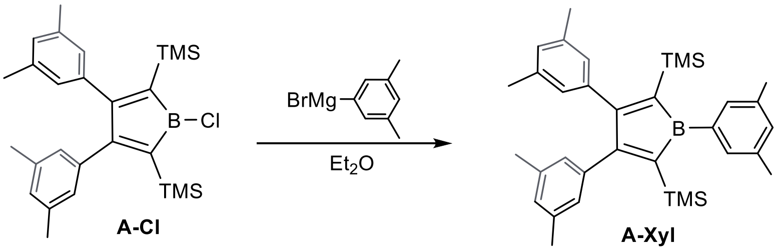

Molecular Weight: 451,00 Molecular Weight: 520,71

In a glovebox, a solution of *m*-xylenemagnesium bromide (11.5 mL, 0.247 M, 1 eq.) in diethylether was added to a cool (ca. –30°C) solution of the chloroborole **A-Cl** (1.2755 g, 2.8282 mmol, 1 eq.) in diethylether. After stirring for 2 h at room temperature, the solvent was removed in vacuo. The dark red and slightly greenish residue was suspended in hexane (2×10 mL) in hexane which was then again removed in vacuo to co-evaporate residual ether. The residue was then repeatedly extracted into hexane (2×5 mL, 4×3 mL) and the extracts were filtered through a syringe filter equipped with a thin pad of glass fiber to give a dark green filtrate. The hexane was then removed under reduced pressure. The dark green residue was thoroughly dried in vacuo and was cooled (ca. –40°C) and carefully washed with small portions (0.5 mL) of cold (ca. –40°C) hexane to remove the soluble green side-product from a red solid. After drying under reduced pressure, the product **A-Xyl** was obtained as a red, crystalline solid (0.64 g, 1.27 mmol, 45 %).

Note: To reduce the amount of the intensely green side-product, it is important to accurately determine the concentration of the Grignard-solution and careful measure the added amount of the reagent as even a slight excess will lead to unwanted byproducts. We diluted previously prepared Grignard solutions to stock solutions of lower concentration and titrated each batch of the Grignard-reagent prior to its use.

Analytical Data for Compound A-Xyl

NMR:

^{1}H (400.13 MHz, 296 K, C_6D_6, C_6D_5H at 7.15 ppm): 7.48 (m, 2H, B-Xy: *o-H*), 6.90 (m, 1H, B-Xy: *p-H*), 6.73 (m, 4H, *o-H*), 6.64 (m, 2H, *p-H*), 2.26 (m, 6H, B-Xy: Ar-C*H*$_3$), 2.02 (m, 12H, Ar-C*H*$_3$), -0.01 (s, 18H, TMS) (multiplets could not be resolved).

^{13}C{^{1}H} (100.61 MHz, 298 K, C_6D_6 solvent signal at 128.0 ppm): 181.3 (C_β), 145.0 (B-Xy: *ipso*-C_{ar}), 140.7 (C_α), 140.6 (*ipso*-C_{ar}), 136.8 (B-Xy: *m*-C_{ar}), 136.6 (*m*-C_{ar}), 131.7 (B-Xy: *p*-C_{ar}), 129.5 (*p*-C_{ar}), 126.7 (*o*-C_{ar}), 21.5 (B-Xy: *m*-CH_3), 21.2 (*m*-CH_3), 1.7 (TMS). Superimposed by solvent: (B-Xy: *o*-C_{ar}).

^{11}B (128.37 MHz, 296 K, C_6D_6): 75.2 ($\omega_{1/2}$ = 710 Hz).

^{29}Si-DEPT20 (79.49 MHz, 297 K, C_6D_6): –9.3.

Elemental Analysis: ($C_{34}H_{45}BSi_2$) calcd C 78.43, H 8.71, B 2.01, Si 10.79, observed C 78.53, H 8.92.

LIFDI-MS: calcd exact mass: 520.32 m/z, observed m/z: 538.7 $[M+H_2O]^+$.

UV-*vis*: λ_{max} 477 nm ($\varepsilon_{477} \approx$ 340 L mol^{-1}cm^{-1}) in *n*-hexane.

Crystal structure of Compound A-Xyl

For further details on the diffraction measurement please see the respective section.

A-Xyl crystallised from solutions in hexane in a freezer (-40°C).

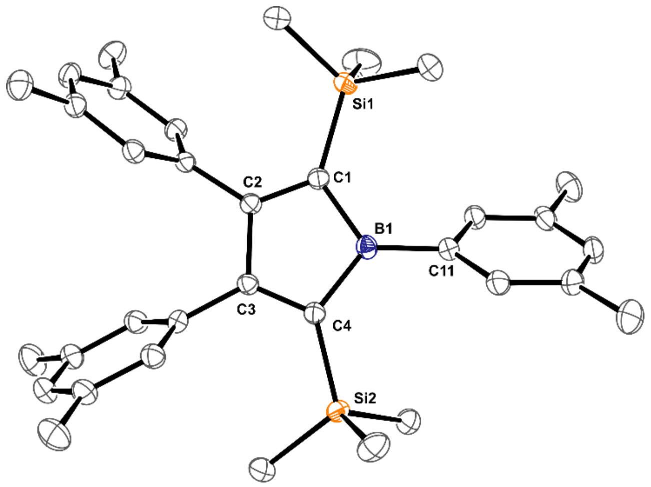

ORTEP plot of the molecular structure of **A-Xyl**. Atomic displacement parameters are drawn at 50% probability level. Hydrogen atoms are omitted for the sake of clarity. Selected bond length in Å: B1-C1 1.588(2), B1-C11 1.555(2), C1-C2 1.361(2), C2-C3 1.530(2), C3-C4 1.361(1), C4-B1 1.593(2), C1-Si1 1.871(2), C4-Si2 1.875(1). The structure was deposited with the CCSD.

Spectra Plots for Compound A-Xyl

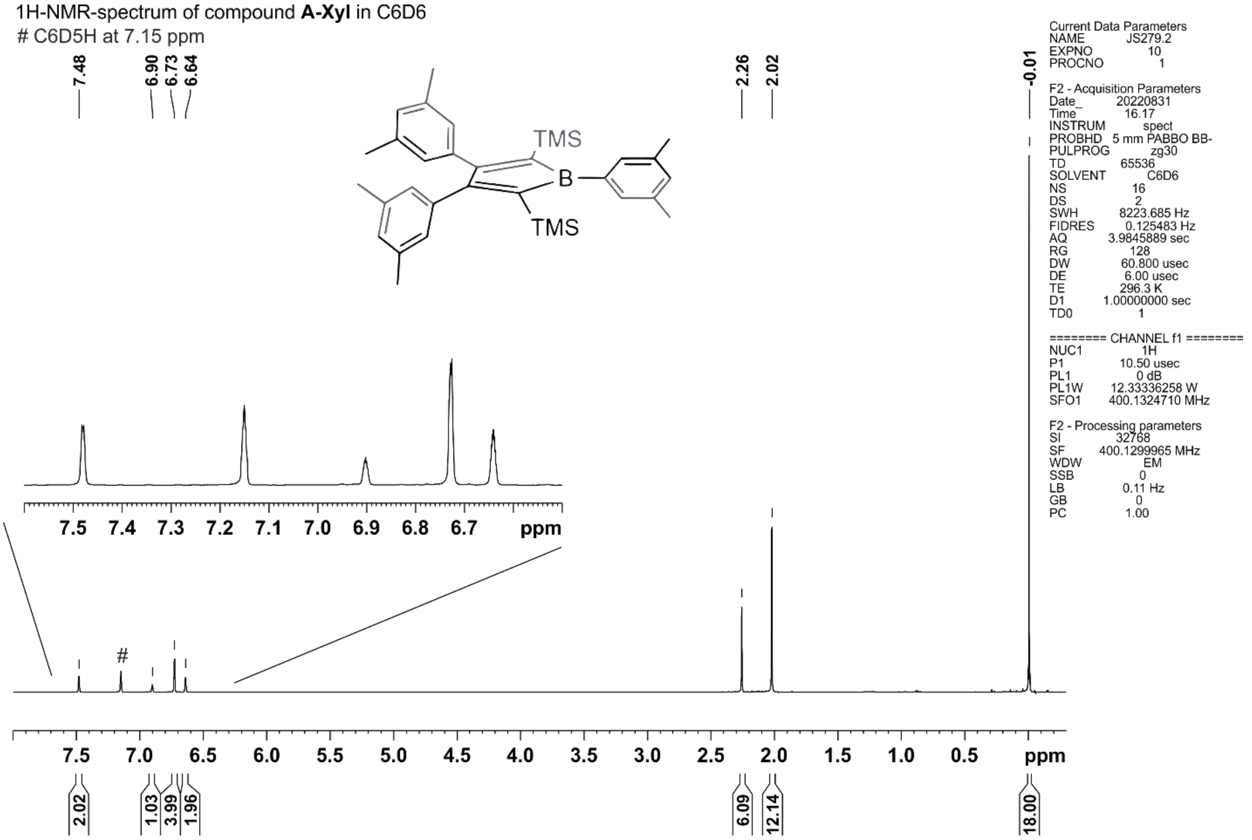

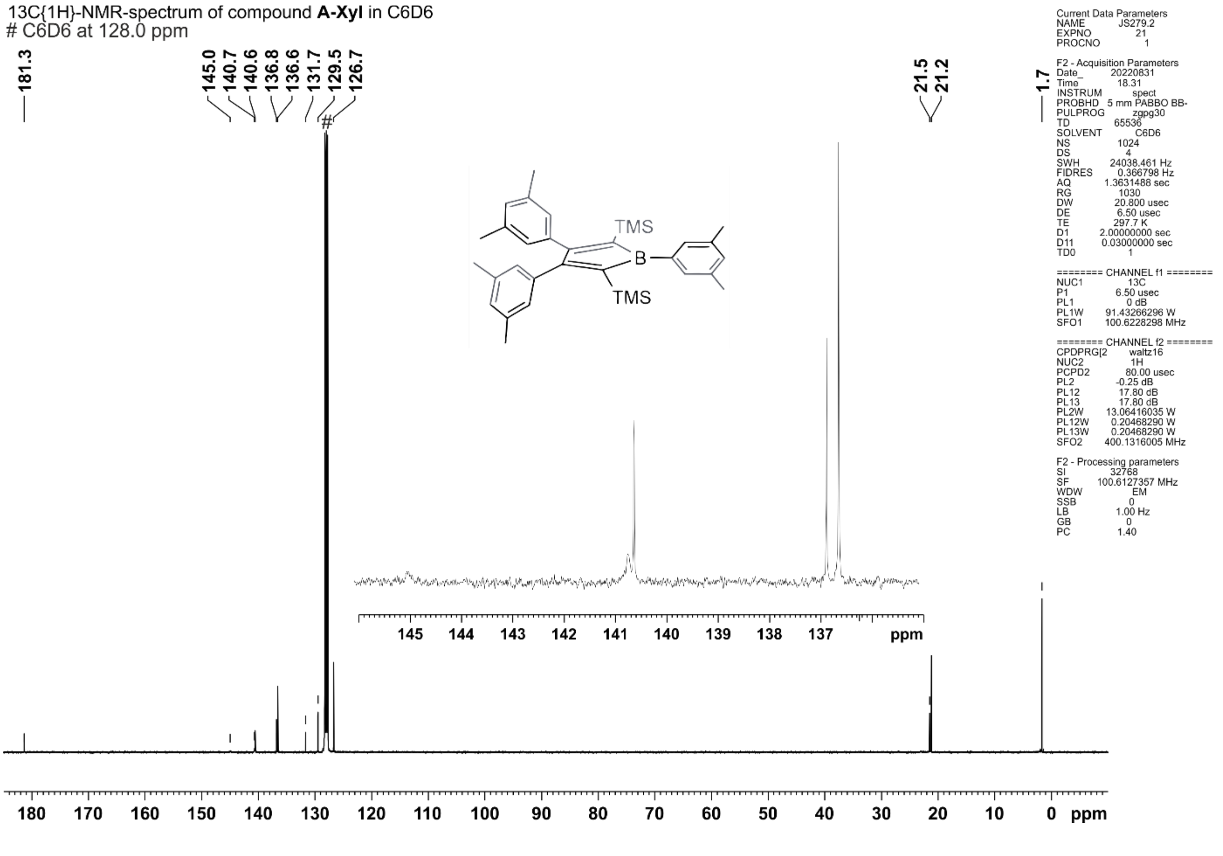

11B-NMR spectrum (background suppressed) of compound **A-Xyl** in C6D6

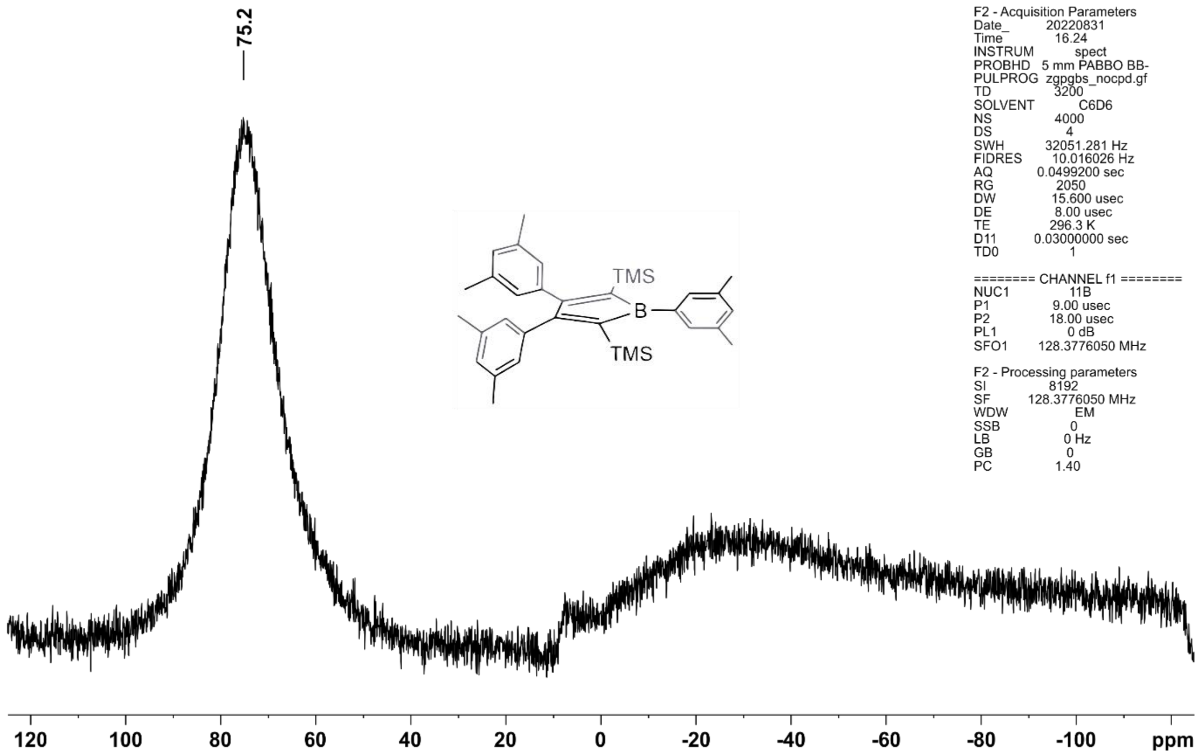

Current Data Parameters
NAME JS279.2
EXPNO 11
PROCNO 1

F2 - Acquisition Parameters
Date_ 20220831
Time 16.24
INSTRUM spect
PROBHD 5 mm PABBO BB-
PULPROG zgpgbs_nocpd.gf
TD 3200
SOLVENT C6D6
NS 4000
DS 4
SWH 32051.281 Hz
FIDRES 10.016026 Hz
AQ 0.0499200 sec
RG 2050
DW 15.600 usec
DE 8.00 usec
TE 296.3 K
D11 0.03000000 sec
TD0 1

======== CHANNEL f1 ========
NUC1 11B
P1 9.00 usec
P2 18.00 usec
PL1 0 dB
SFO1 128.3776050 MHz

F2 - Processing parameters
SI 8192
SF 128.3776050 MHz
WDW EM
SSB 0
LB 0 Hz
GB 0
PC 1.40

Current Data Parameters
NAME JS279.2
EXPNO 27
PROCNO 1

29Si-DEPT-NMR spectrum of compound **A-Xyl** in C6D6

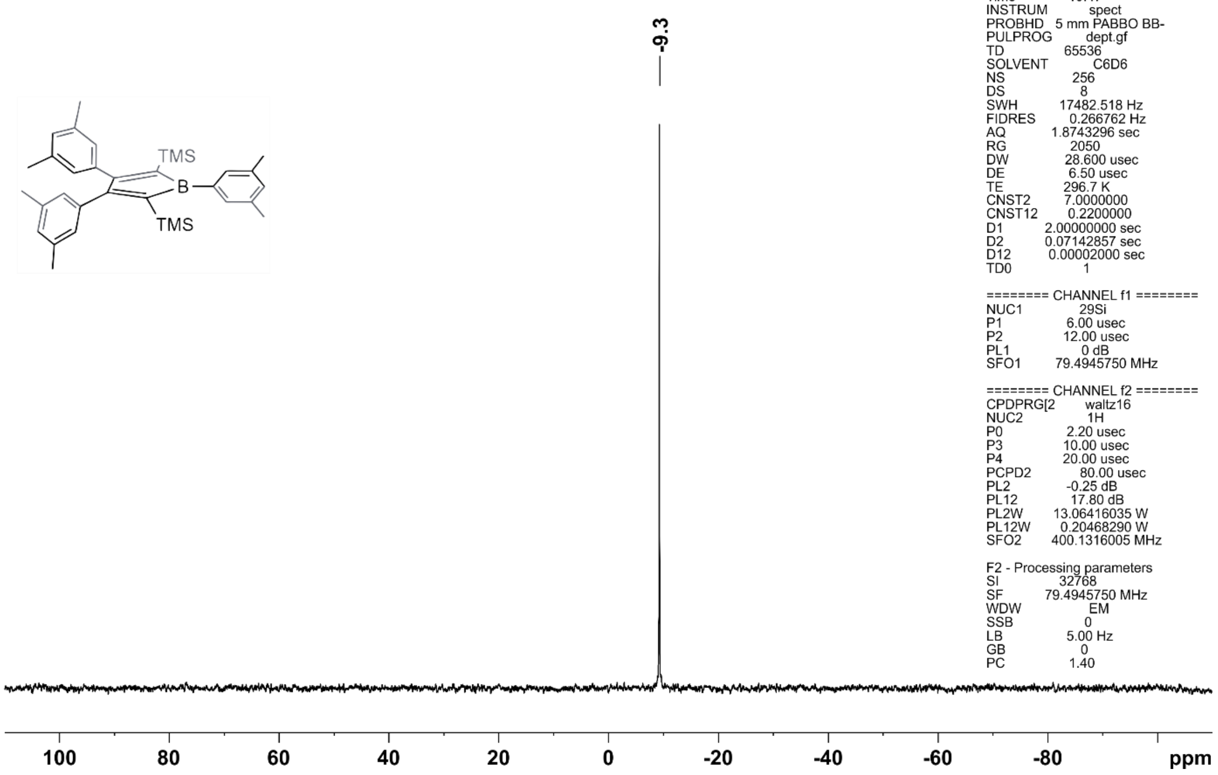

F2 - Acquisition Parameters
Date_ 20220831
Time 19.47
INSTRUM spect
PROBHD 5 mm PABBO BB-
PULPROG dept.gf
TD 65536
SOLVENT C6D6
NS 256
DS 8
SWH 17482.518 Hz
FIDRES 0.266762 Hz
AQ 1.8743296 sec
RG 2050
DW 28.600 usec
DE 6.50 usec
TE 296.7 K
CNST2 7.0000000
CNST12 0.2200000
D1 2.00000000 sec
D2 0.07142857 sec
D12 0.00002000 sec
TD0 1

======== CHANNEL f1 ========
NUC1 29Si
P1 6.00 usec
P2 12.00 usec
PL1 0 dB
SFO1 79.4945750 MHz

======== CHANNEL f2 ========
CPDPRG[2 waltz16
NUC2 1H
P0 2.20 usec
P3 10.00 usec
P4 20.00 usec
PCPD2 80.00 usec
PL2 -0.25 dB
PL12 17.80 dB
PL2W 13.06416035 W
PL12W 0.20468290 W
SFO2 400.1316005 MHz

F2 - Processing parameters
SI 32768
SF 79.4945750 MHz
WDW EM
SSB 0
LB 5.00 Hz
GB 0
PC 1.40

Acq. Data Name: jsarcev00016-1
Creation Parameters: Average(MS[1] Time:0.43..0.44)
External Sample Id: JS 167

Experiment Date/Time: 8/11/2021 10:33:57 AM
Ionization Mode: FD+

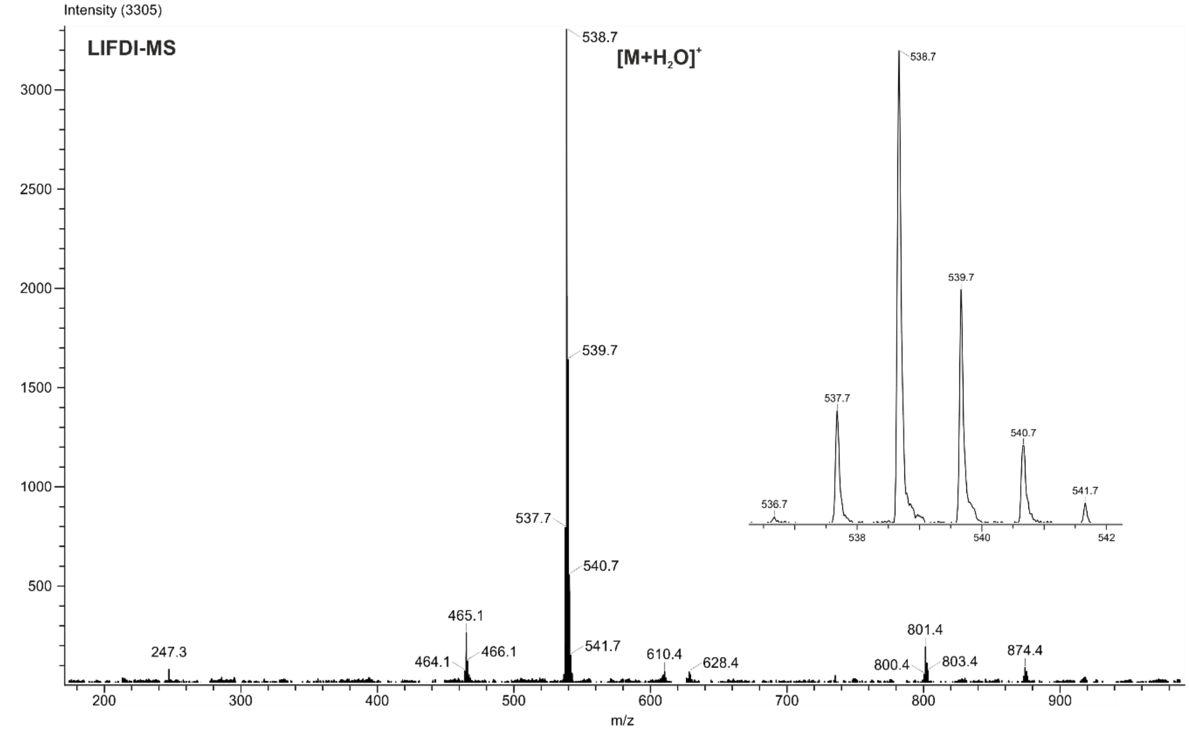

Compound A-*p*Xyl

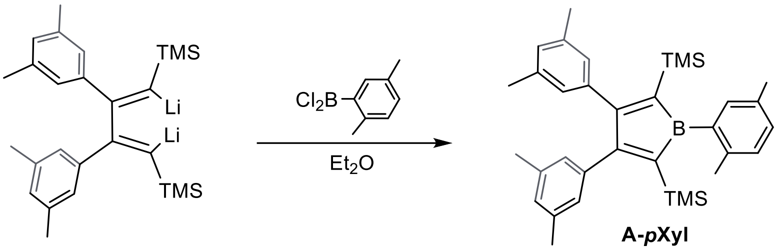

Molecular Weight: 418,62 Molecular Weight: 520,71

In a glovebox, a solution of 2,5-dimethylphenyl borondichloride in hexane (0.7 M, 2.266 mL, 1.573 mmol. 1 eq.) was added to a dark red solution of the 1,4-dilithio-1,3-butadiene (658.3 mg, 1.573 mmol, 1 eq.) in diethylether (7 mL) and the resulting suspension was stirred at room temperature for 25 min. The ether was removed under reduced pressure and resulting residue was suspended in hexane which was then subsequently removed to co-evaporate residual ether. The residue was extracted into in pentane (3 mL, 2 mL) and the extracts were filtered through a syringe filter equipped with a thin pad of glass fiber. The pentane was then removed under reduced pressure from the filtrate to give a red sticky substance. The substance was scraped of the walls of the vial and dried thoroughly in vacuo. The product was obtained as a red powder (616.0 mg, 1.183 mmol, 75 %).

Note: With minimal amounts of residual solvent (such as pentane) the crude product tends to be unpleasantly sticky and prolonged drying in vacuo and mechanical treatments are necessary to obtain a powder.

Analytical Data for Compound A- *p*Xyl

NMR:

^{1}H (400.30 MHz, 298 K, C_6D_6, C_6D_5H at 7.15 ppm): 7.18 (s, 1H, B-Ar: *o-H*), 7.04 (d, 1H, , ^{3}J = 7.80 Hz, B-Ar: *m-H*), 6.90 (dd, 1H, , ^{3}J = 7.84 Hz, ^{4}J = 1.20 Hz, B-Ar: *p-H*), 6.74 (s, 4H, *o-H*), 6.62 (s, 2H, *p-H*), 2.49 (s, 3H, *o-CH*$_3$), 2.23 (s, 3H, B-Ar: *m-CH*$_3$), 2.00 (s, 12H, *m-CH*$_3$), -0.06 (s, 18H,TMS).

^{13}C{^{1}H} (100.67 MHz, 298 K, C_6D_6 solvent signal at 128.0 ppm): 181.2 (C_β), 146.2 (B-Ar: *ipso-C*), 140.4 (C_α), 140.2 (*ipso-C*), 136.6 (*m-C*-CH$_3$), 133.9 (B-Ar: *m-C*-CH$_3$), 133.6 (B-Ar: *o-C*-CH$_3$), 129.5 (*p-C*), 129.3 (B-Ar: *m-C*H), 128.9 (B-Ar: *p-C*), 126.7 (B-Ar: *o-C*H), 126.5 (*o-C*), 22.5 (B-Ar: *o-C*H$_3$), 21.3 (B-Ar: *m-C*H$_3$), 21.1 (*m-C*H$_3$), 1.1 (TMS).

^{11}B (128.43 MHz, 298 K, C_6D_6): 78.8 ($\omega_{1/2}$ = 1645 Hz).

^{29}Si-INEPT (79.52 MHz, 298 K, C_6D_6): -9.2 (TMS).

Elemental Analysis: ($C_{34}H_{49}BSi_2$) calcd C 78.43, H 8.71, B 2.08, Si 10.79, observed C 77.72, H 8.89.

LIFDI-MS: calcd exact mass: 520.32 m/z, observed m/z: 538.7 $[M-H_2O]^+$, 520.8 $[M]^+$.

UVvis: shoulder at ca. 470 nm (ε_{470} ca. 240 L mol^{-1}cm^{-1}) and shoulder at ca. 420 nm.

Spectra Plots for Compound A-*p*Xyl

1H-NMR-spectrum of compound **A-*p*Xyl** in C6D6
\# C6D5H at 7.15 ppm

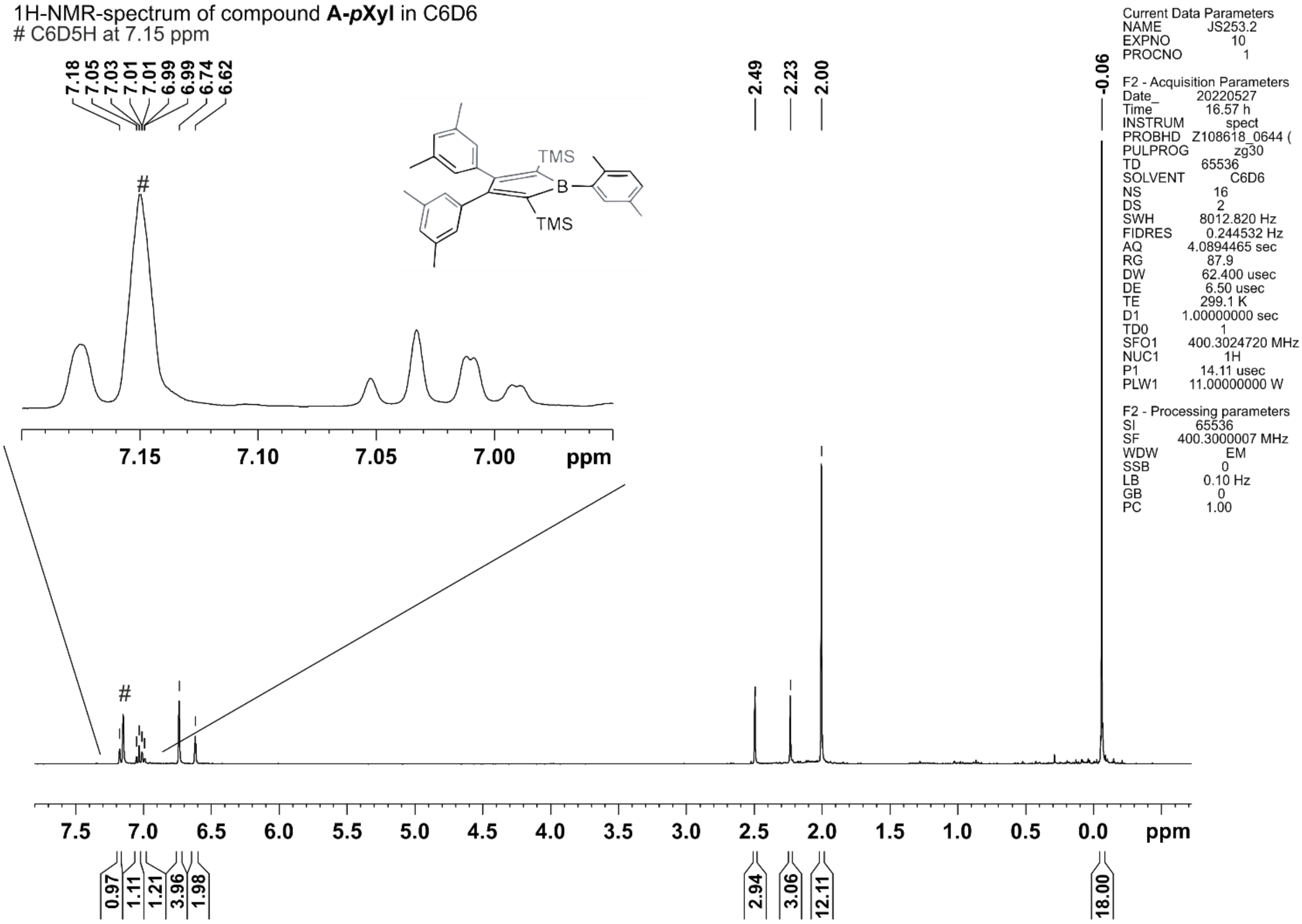

13C{1H}-NMR-spectrum of compound **A-*p*Xyl** in C6D6
\# C6D6 at 128.0 ppm

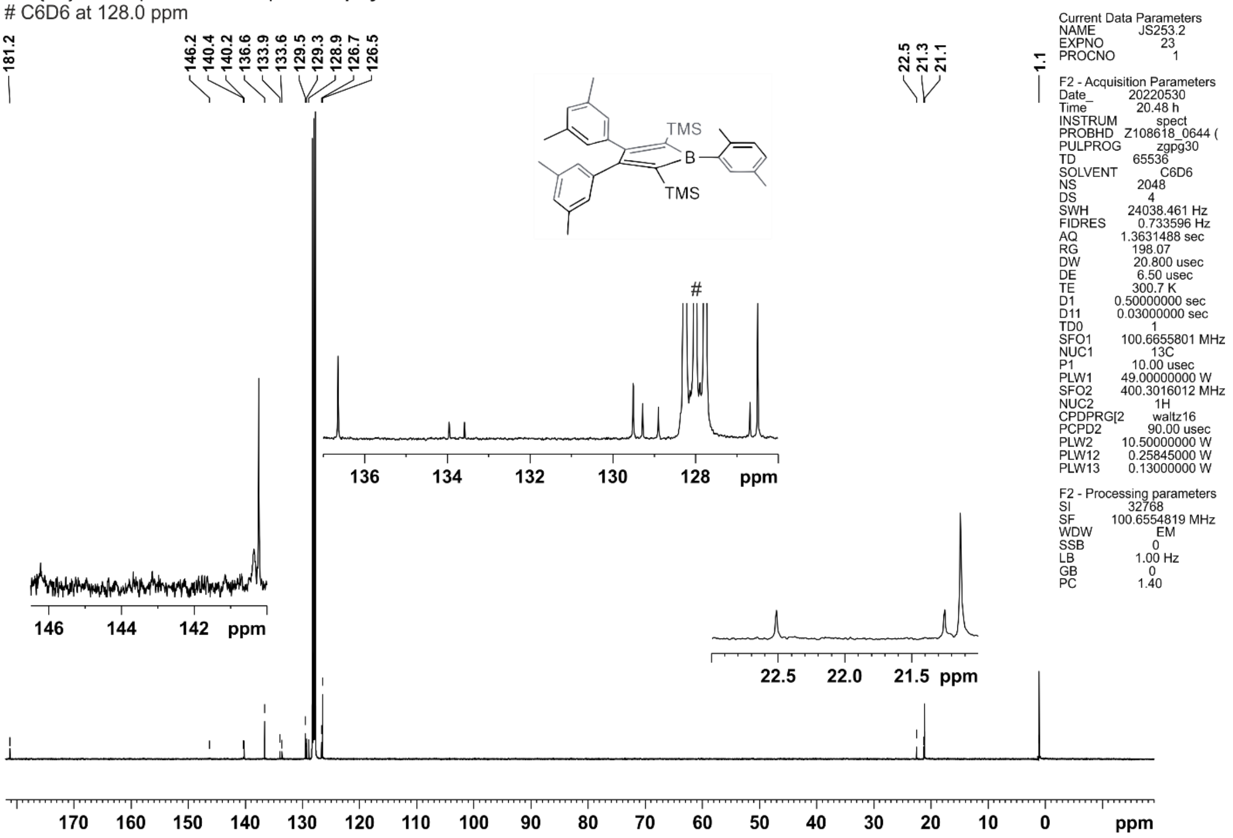

11B-NMR spectrum (background suppressed) of compound **A-*p*Xyl** in C6D6

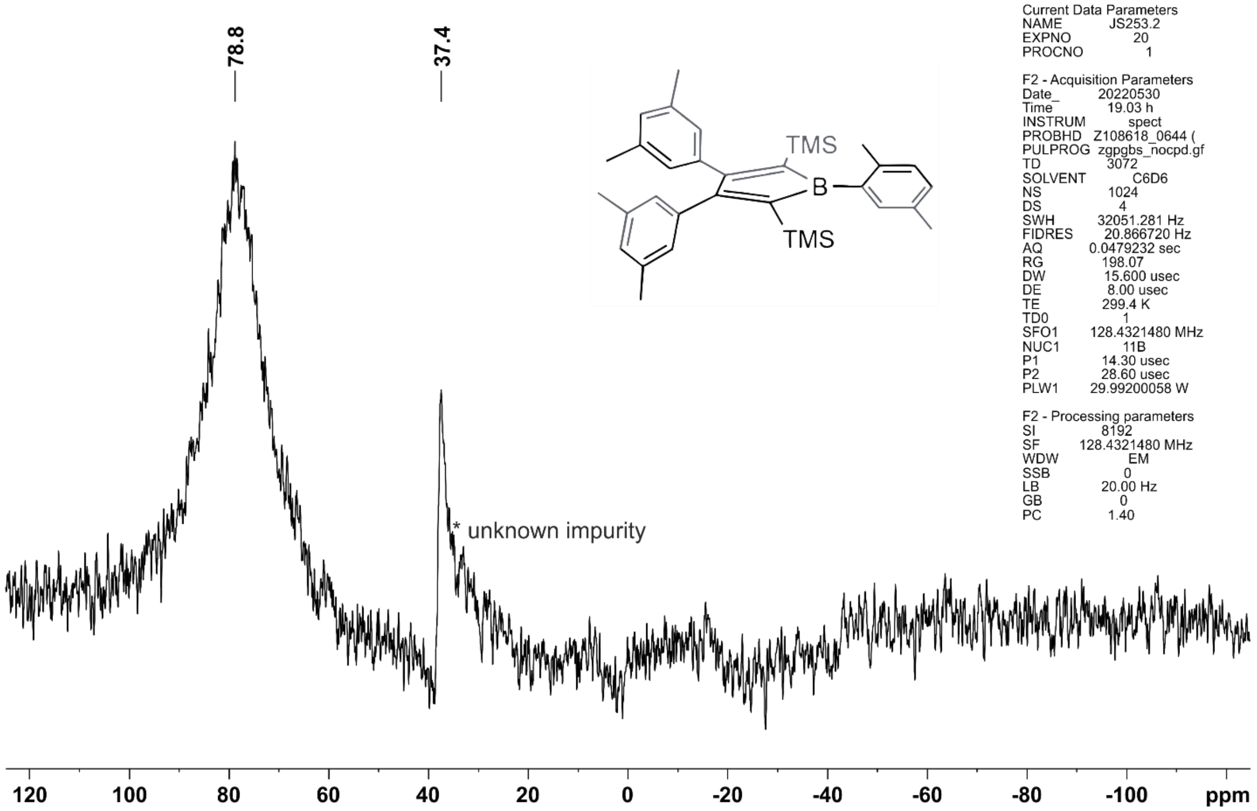

29Si-DEPT20-NMR spectrum of compound **A-*p*Xyl** in C6D6

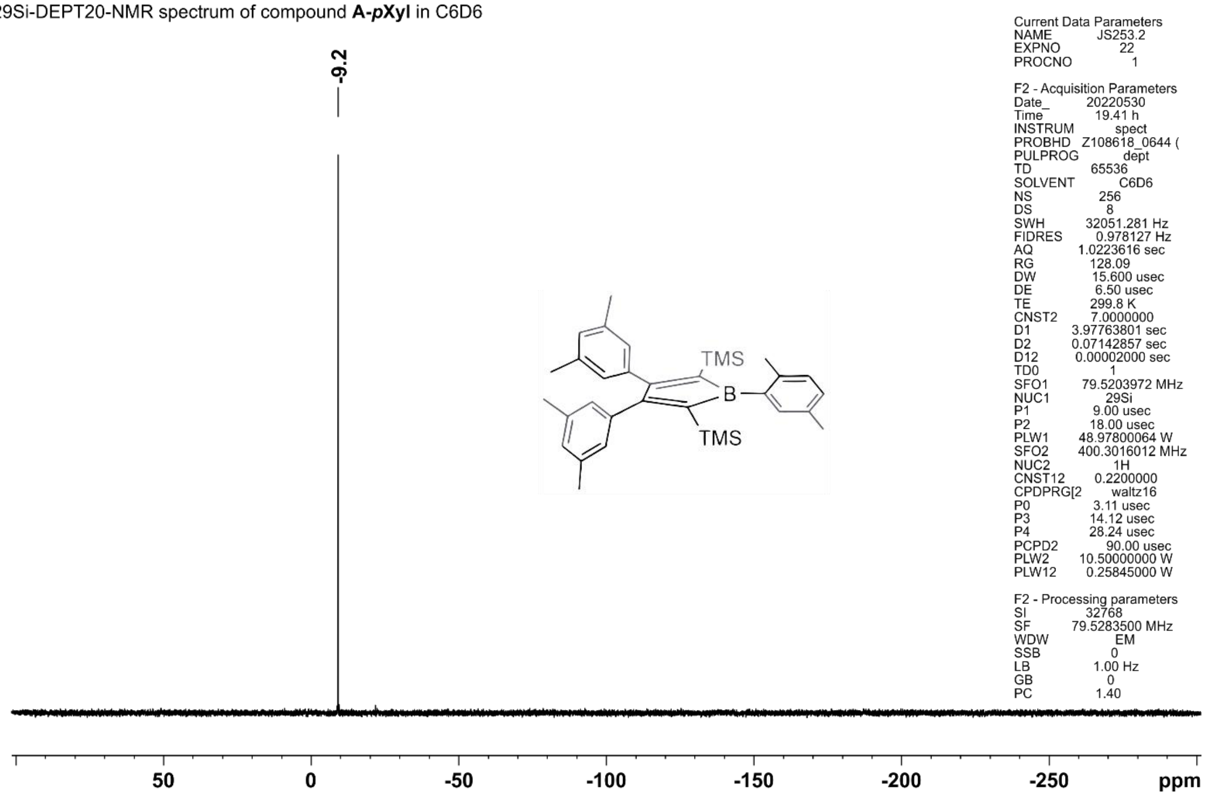

Acq. Data Name: jsarcev00012-1
Creation Parameters: Average(MS[1] Time:0.33..0.41)
External Sample Id: JS 134

Experiment Date/Time: 5/26/2021 10:21:10 AM
Ionization Mode: FD+

LIFDI-MS

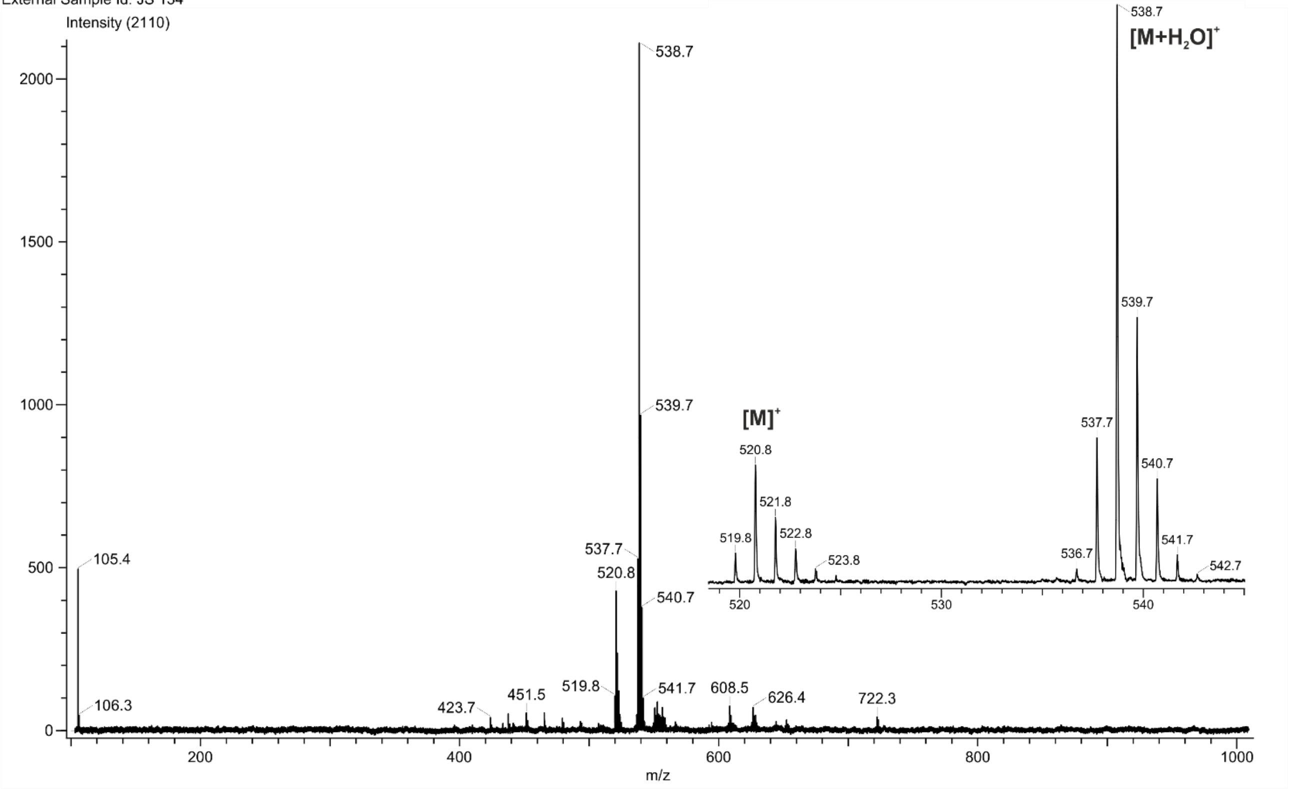

Compound B-Xyl

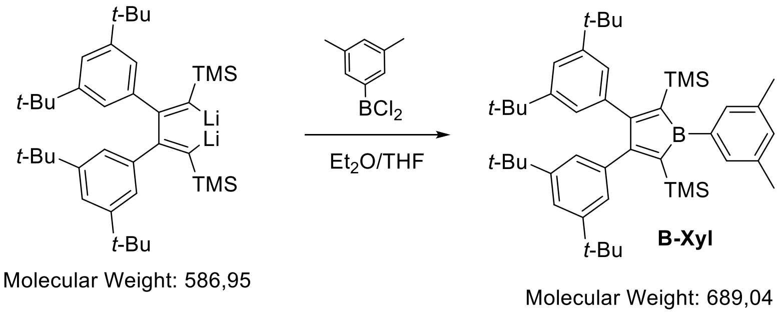

In a glovebox, dichloro-(3,5-dimethylphenyl)-borane (191.2 mg, 1.023 mmol, 1 eq) was cooled to –40 °C and dissolved in a solvent mixture of diethyl ether/THF (volume ratio: 5.5/1, 1.2 mL). The solution was allowed to warm to ambient temperature and was added dropwise to a dark red solution of 1,4-dilithio-1,3-butadiene (600.5 mg, 1.023 mmol, 1 eq) in diethyl ether/THF (volume ratio: 5.5/1, 6.5 mL). A white solid immediately started to precipitate and the colour of the reaction mixture turned from dark red to orange over the course of two hours. After stirring the reaction at ambient temperature for 3 hours, the solvent was removed under reduced pressure and the remaining red solid was extracted with *n*-hexane (17 mL) and the extracts were filtered over a syringe filter equipped with a thin pad of glass fiber. The solvent of the red hexane extract was removed under reduced pressure and the so obtained red solid was successively dissolved and dried under reduced pressure with 2-MeTHF (2 x 4 mL) and *n*-hexane (1 x 3 mL) to co-evaporate residual donor solvents. The solid was once again dissolved in *n*-hexane (4.5 mL) and stored at –40 °C overnight to yield red crystals of the desired borole **B-Xyl**. The mother liquor was removed with a syringe and the crystals were washed with cold *n*-hexane (–40 °C, 3 x 0.3 mL). After a second crystallization step borole **B-Xyl** was obtained in a combined yield as a red, crystalline solid (399.7 mg, 0.580 mmol, 57 %).

Analytical Data for Compound B-Xyl

NMR:

^{1}H (400.13 MHz, 296 K, C_6D_6, CD_5H at 7.15 ppm): 7.54 (m, 2H, *o-H_{Xyl}*), 7.29 (t, $^{4}J_{HH}$ = 1.8 Hz, 2H, *p-$H_{ar3/4}$*), 6.92 (1H, *p-H_{Xyl}*, perfectly overlapping with the signal for the o-$H_{ar3/4}$-protons), 6.92 (d, $^{4}J_{HH}$ = 1.8 Hz, 4H, *o-$H_{ar3/4}$*), 2.26 (s, 6H, C*H_3*), 1.20 (s, 36H, Ar-C(*Me*)$_3$), 0.00 (s, 18H, Si(*Me*)$_3$).

^{13}C{^{1}H} (100.62 MHz, 298 K, C_6D_6, solvent signal at 128.0 ppm): 182.8 (*C_β*), 149.6 (*m-$C_{Ar3/4}$*), 145.2 (*ipso-C_{Xyl}*), 140.1 (*ipso-$C_{Ar3/4}$*), 139.4 (*C_α*), 136.9 (*m-C_{Xyl}*), 132.0 (*p-C_{Xyl}*), 128.7 (*o-C_{Xyl}*), 123.3 (*o-$C_{ar3/4}$*), 121.2 (*p-$C_{ar3/4}$*), 34.8 ($Ar_{3/4}$-*C*(CH_3)$_3$), 31.6 ($Ar_{3/4}$-(C(*C*H_3)$_3$), 21.6 (*C*H_3), 1.6 (Si(*C*H_3)$_3$).

^{11}B (128.38 MHz, 296 K, C_6D_6): 74.5 ($\nu_{1/2}$ ≈ 2300 Hz).

^{29}Si (DEPT-20, 79.49 MHz, 297 K, C_6D_6): –9.2 (TMS).

Elemental Analysis: $C_{46}H_{69}BSi_2$ calcd C 80.19, H 10.09; observed C 80.47, H 10.45.

UV/VIS (*n*-hexane): λ_{max} = 480 nm. (ε_{480} ≈ 220 L mol^{-1}cm^{-1})

Spectra Plots for Compound B-Xyl

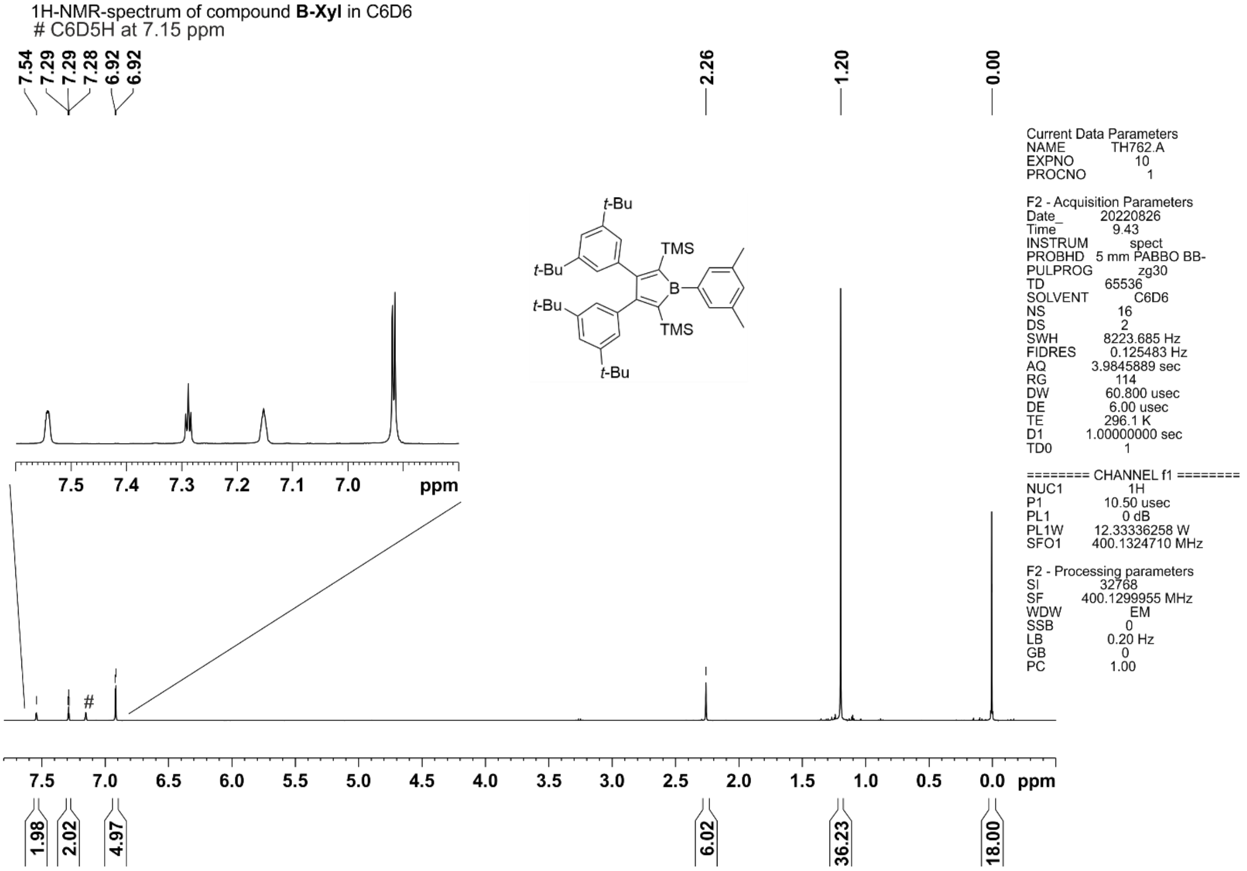

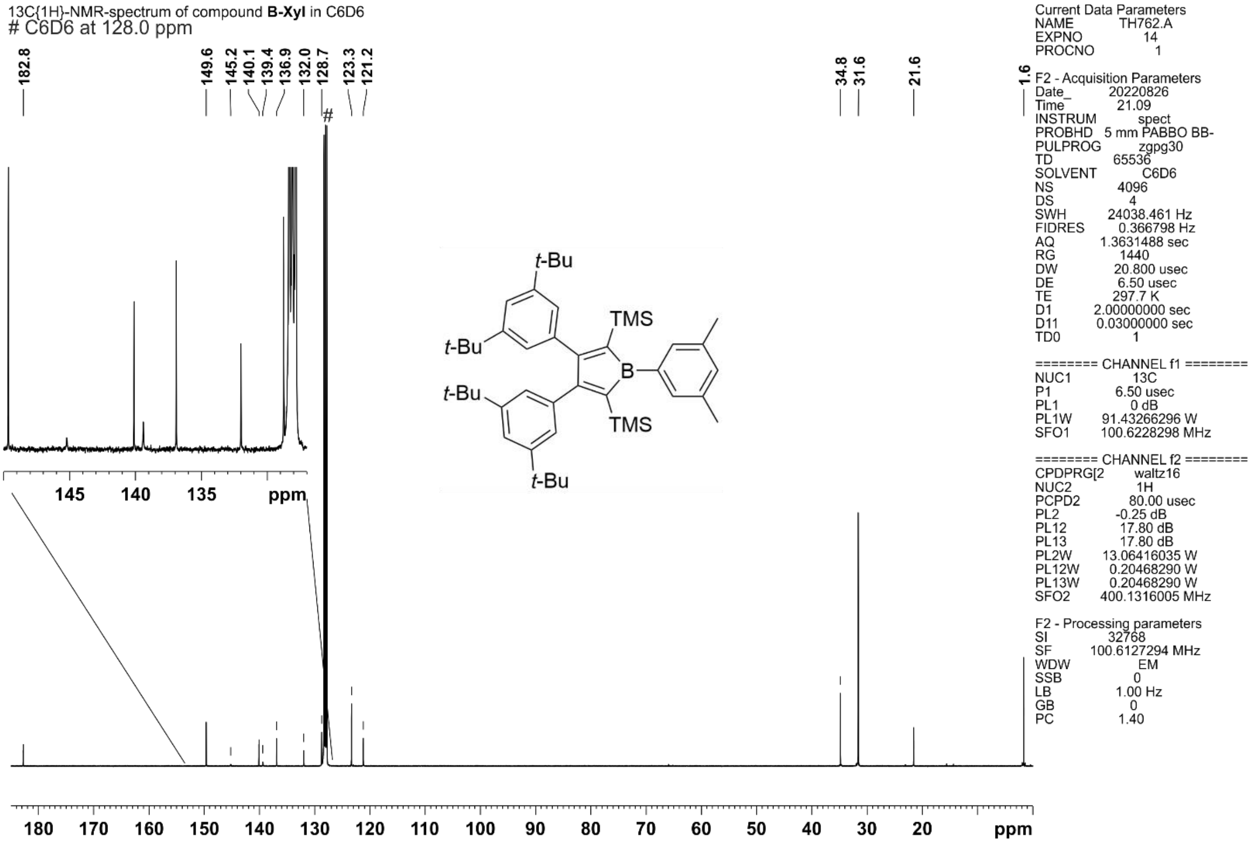

11B-NMR spectrum (background suppressed) of compound **B-Xyl** in C6D6

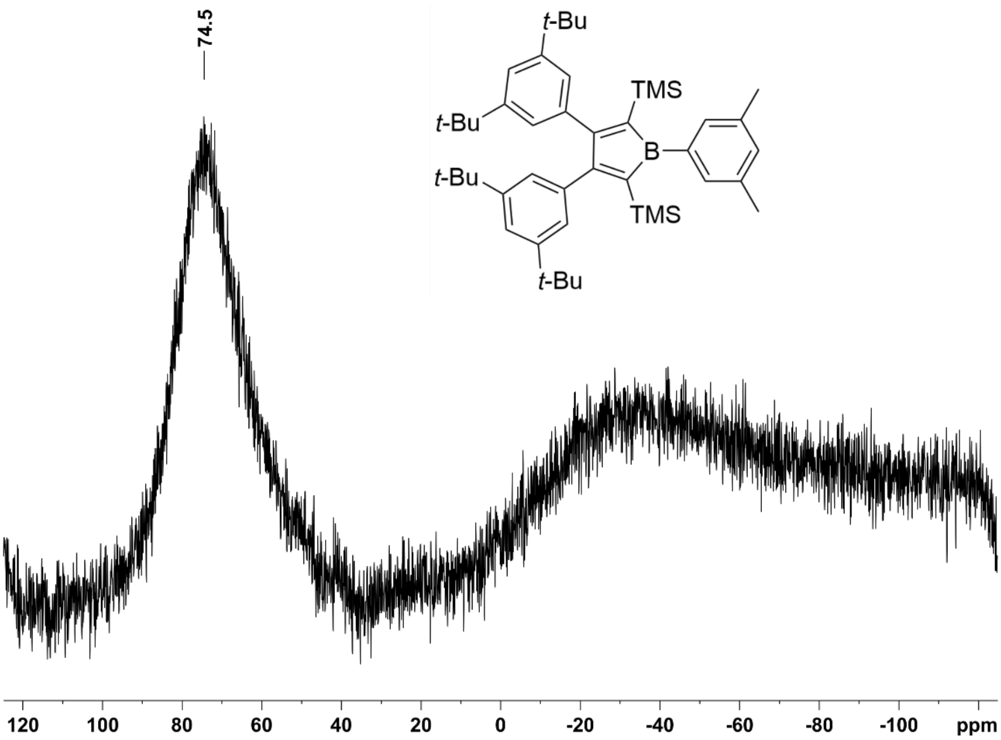

Current Data Parameters
NAME TH762.A
EXPNO 11
PROCNO 1

F2 - Acquisition Parameters
Date_ 20220826
Time 9.56
INSTRUM spect
PROBHD 5 mm PABBO BB-
PULPROG zgpgbs_nocpd.gf
TD 3200
SOLVENT C6D6
NS 8192
DS 4
SWH 32051.281 Hz
FIDRES 10.016026 Hz
AQ 0.0499200 sec
RG 2050
DW 15.600 usec
DE 8.00 usec
TE 296.2 K
D11 0.03000000 sec
TD0 1

======== CHANNEL f1 ========
NUC1 11B
P1 9.00 usec
P2 18.00 usec
PL1 0 dB
SFO1 128.3776050 MHz

F2 - Processing parameters
SI 8192
SF 128.3776050 MHz
WDW EM
SSB 0
LB 0 Hz
GB 0
PC 1.40

29Si-NMR (DEPT-20)spectrum of compound **B-Xyl** in C6D6

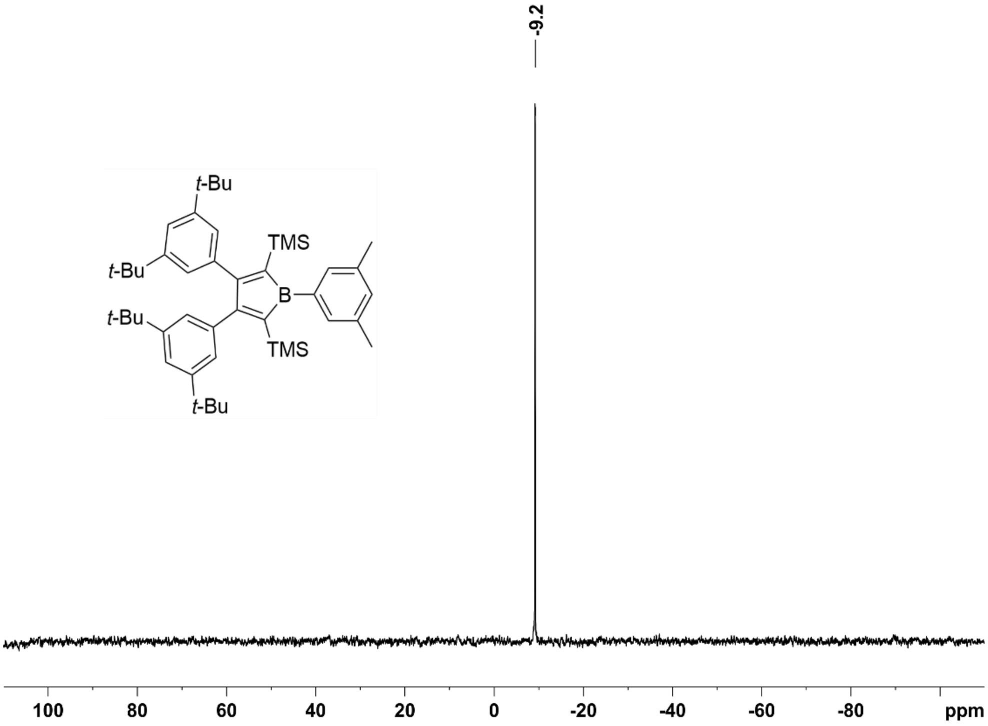

Current Data Parameters
NAME TH762.A
EXPNO 12
PROCNO 1

F2 - Acquisition Parameters
Date_ 20220826
Time 10.17
INSTRUM spect
PROBHD 5 mm PABBO BB-
PULPROG dept.gf
TD 65536
SOLVENT C6D6
NS 256
DS 8
SWH 17482.518 Hz
FIDRES 0.266762 Hz
AQ 1.8743296 sec
RG 2050
DW 28.600 usec
DE 6.50 usec
TE 296.7 K
CNST2 7.0000000
CNST12 0.2200000
D1 2.00000000 sec
D2 0.07142857 sec
D12 0.00002000 sec
TD0 1

======== CHANNEL f1 ========
NUC1 29Si
P1 6.00 usec
P2 12.00 usec
PL1 0 dB
SFO1 79.4945750 MHz

======== CHANNEL f2 ========
CPDPRG[2 waltz16
NUC2 1H
P0 2.20 usec
P3 10.00 usec
P4 20.00 usec
PCPD2 80.00 usec
PL2 -0.25 dB
PL12 17.80 dB
PL2W 13.06416035 W
PL12W 0.20468290 W
SFO2 400.1316005 MHz

F2 - Processing parameters
SI 32768
SF 79.4945750 MHz
WDW EM
SSB 0
LB 5.00 Hz
GB 0
PC 1.40

Compound B-*p*Xyl

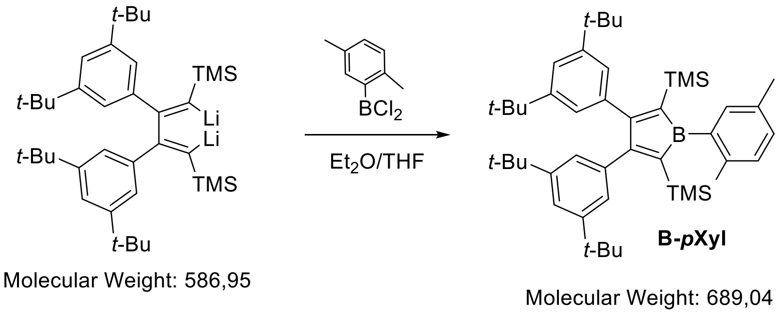

In a glovebox, dichloro-(2,5-dimethylphenyl)-borane (77.0 mg, 0.412 mmol, 1 eq) was cooled to –40 °C and dissolved in a solvent mixture of diethyl ether/THF (ratio: 3/1, 0.6 mL). The solution was allowed to warm to ambient temperature and was added dropwise to a dark red solution of 1,4-dilithio-1,3-butadiene (241.8 mg, 0.412 mmol, 1 eq) in diethyl ether/THF (ratio: 3/1, 2 mL). A white solid immediately started to precipitate. After stirring the reaction at ambient temperature for ca. 3 hours, the solvent of the dark red suspension was removed under reduced pressure and the remaining red solid was extracted into *n*-hexane (3 x 1 mL) and the extracts were filtered through a syringe filter equipped with a thin pad of glass fiber. The solvent of the red hexane extract was once again removed under reduced pressure and the obtained red solid was dissolved in *n*-hexane (1.1 mL) and stored at –40 °C overnight to yield red crystals of the desired borole **B-*p*Xyl**. The mother liquor was removed with a syringe and the crystals were washed with cold *n*-hexane (–40 °C, 3 x 0.15 mL). After a second crystallization step borole **B-*p*Xyl** was obtained in a combined yield as a red, crystalline solid (192.6 mg, 0.279 mmol, 68 %).

Analytical Data for Compound B-*p*Xyl

NMR:

^{1}H (400.13 MHz, 299 K, C_6D_6, CD_5H at 7.15 ppm): 7.29 (t, $^4J_{HH}$ = 1.8 Hz, 2H, *p-H*$_{ar3/4}$), 7.18 (br s, 1H, *o-H*$_{Xyl}$), 7.00–7.03 (m, 2H, *m-H*$_{Xyl}$ + *p-H*$_{Xyl}$), 6.97 (d, $^4J_{HH}$ = 1.8 Hz, 4H, *o-H*$_{ar3/4}$), 2.45 (s, 3H, o-C*H*$_3$), 2.21 (s, 3H, m-C*H*$_3$), 1.18 (s, 36H, Ar-C(*Me*)$_3$), –0.04 (s, 18H, Si(*Me*)$_3$).

^{13}C{^{1}H} (100.65 MHz, 298 K, C_6D_6, solvent signal at 128.0 ppm): 182.4 (C_β), 149.7 (*m-C*$_{Ar3/4}$), 146.5 (*ipso-C*$_{Xyl}$), 139.8 (*ipso-C*$_{Ar3/4}$), 139.5 (C_α), 134.2 (*o-C*$_{Xyl}$-CH$_3$), 134.0 (*m-C*$_{Xyl}$-CH$_3$), 129.4 (*m-C*H$_{Xyl}$), 129.1 (*p-C*H$_{Xyl}$), 126.9 (*o-C*H$_{Xyl}$), 123.2 (o-*C*$_{ar3/4}$), 121.4 (*p-C*$_{ar3/4}$), 34.8 (Ar$_{3/4}$-*C*(CH$_3$)$_3$), 31.6 (Ar$_{3/4}$-(C(*C*H$_3$)$_3$), 22.8 (*o-C*H$_3$), 21.3 (*m-C*H$_3$), 1.1 (Si(*C*H$_3$)$_3$).

^{11}B (128.43 MHz, 299 K, C_6D_6): 79.0 ($\nu_{1/2} \approx$ 2500 Hz).

^{29}Si (DEPT-20, 79.49 MHz, 299 K, C_6D_6): –9.1 (TMS).

Elemental Analysis: $C_{46}H_{69}BSi_2$ calcd C 80.19, H 10.09; observed C 80.04, H 10.18.

UV/VIS (*n*-hexane): $\lambda_{max} \approx$ 480 nm (shoulder). ($\varepsilon_{480} \approx$ 248 L mol^{-1}cm^{-1})

Crystal structure of Compound B-*p*Xyl

For further details on the diffraction measurement please see the respective section.

B-*p*Xyl crystallised from solutions in hexane in a freezer (-40°C).

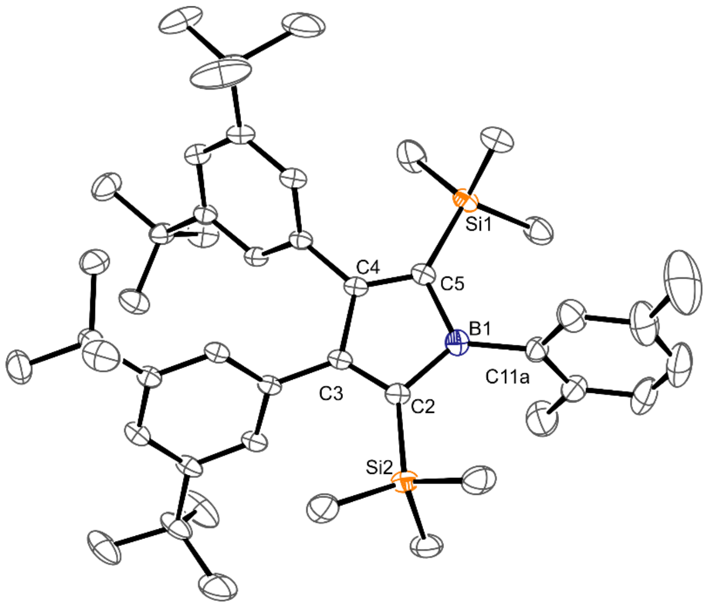

ORTEP plot of the molecular structure of **B-*p*Xyl**. Atomic displacement parameters are drawn at 50% probability level. A disordered lattice hexane, disorder of one *t*Bu group, a disordered B-bound *p*Xyl group and hydrogen atoms are omitted for the sake of clarity. Selected bond length in Å: B1-C2 1.585(2), C2-C3 1.358(2), C3-C4 1.543(2), C4-C5 1.358(2), C5-B1 1.584(2), B1-C11a 1.577(5), C2-Si2 1.871(2), C5-Si1 1.871(2). The structure was deposited with the CCSD.

Spectra Plots for Compound B-*p*Xyl

1H-NMR-spectrum of compound **B-pXyl** in C6D6
C6D5H at 7.15 ppm

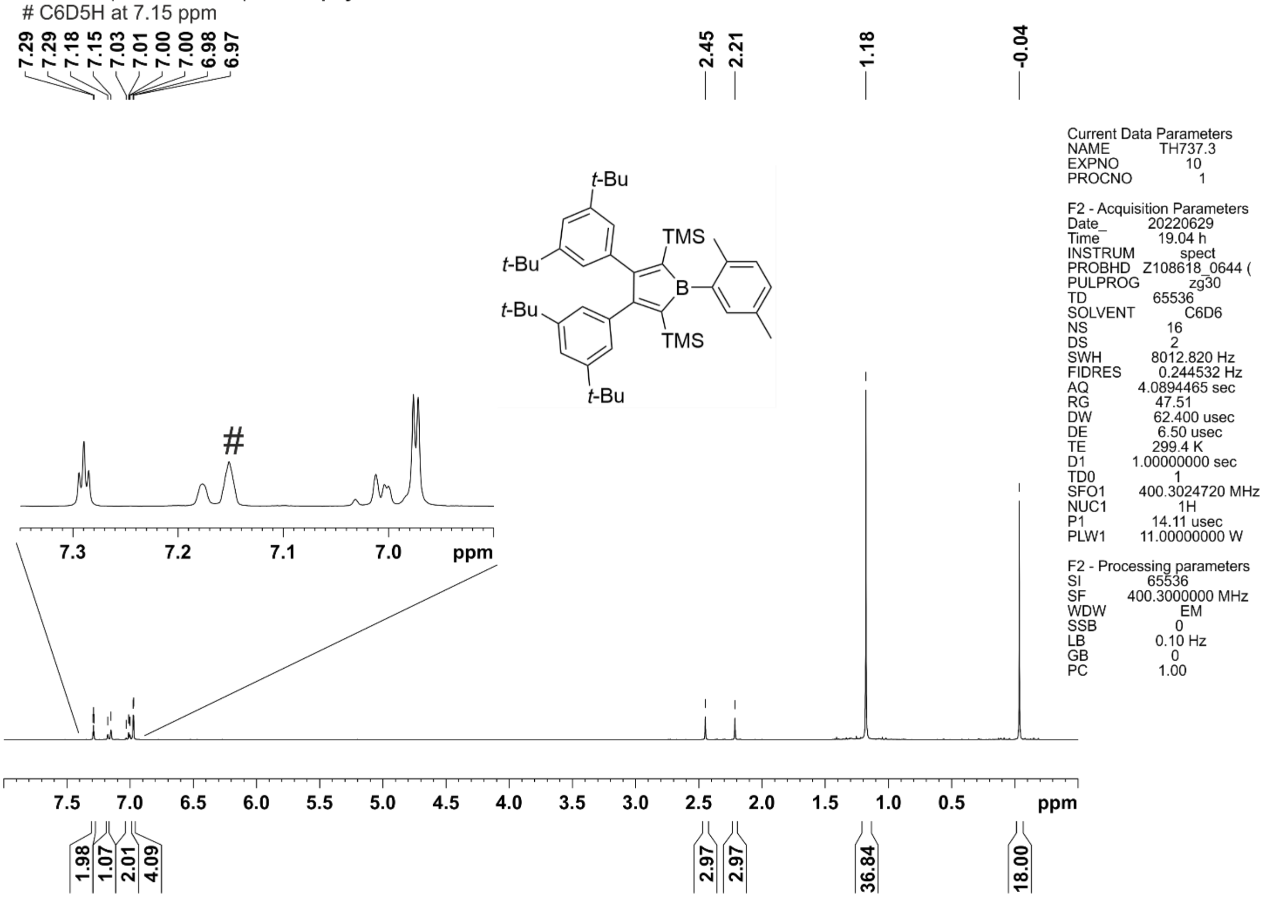

13C{1H}-NMR-spectrum of compound **B-pXyl** in C6D6
C6D6 at 128.0 ppm

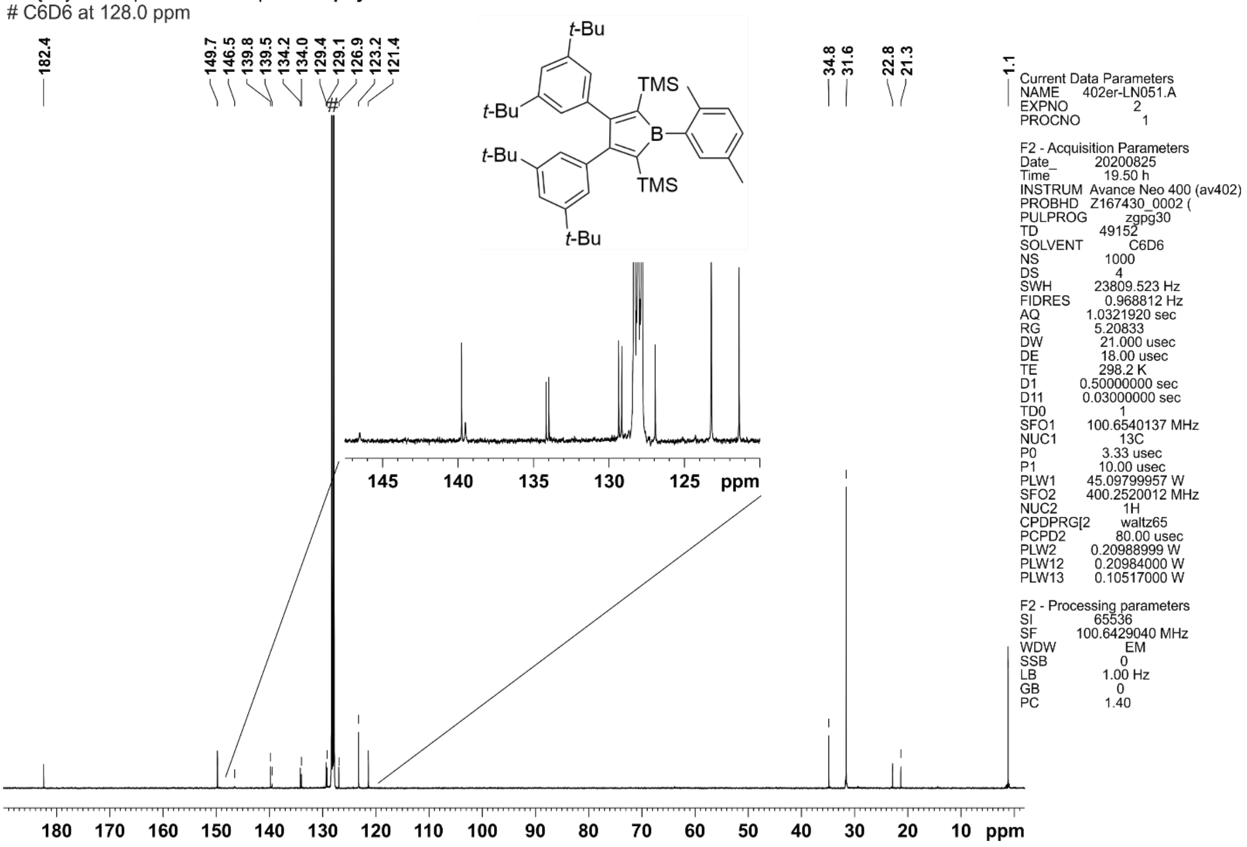

11B-NMR spectrum (background suppressed) of compound **B-pXyl** in C6D6

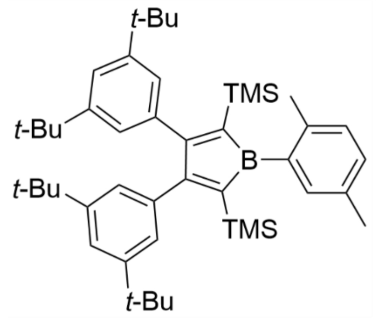

Current Data Parameters
NAME TH737.3
EXPNO 11
PROCNO 1

F2 - Acquisition Parameters
Date_ 20220629
Time 19.10 h
INSTRUM spect
PROBHD Z108618_0644 (
PULPROG zgpgbs_nocpd.gf
TD 3072
SOLVENT C6D6
NS 4096
DS 4
SWH 32051.281 Hz
FIDRES 20.866720 Hz
AQ 0.0479232 sec
RG 198.07
DW 15.600 usec
DE 8.00 usec
TE 299.5 K
TD0 1
SFO1 128.4321480 MHz
NUC1 11B
P1 14.30 usec
P2 28.60 usec
PLW1 29.99200058 W

F2 - Processing parameters
SI 8192
SF 128.4321480 MHz
WDW EM
SSB 0
LB 20.00 Hz
GB 0
PC 1.40

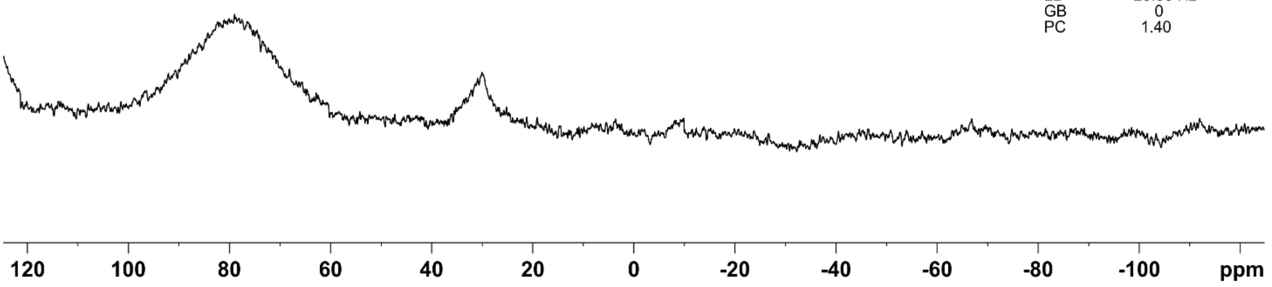

Current Data Parameters
NAME TH737.3
EXPNO 12
PROCNO 1

29Si-NMR (DEPT-20) spectrum of compound **B-pXyl** in C6D6

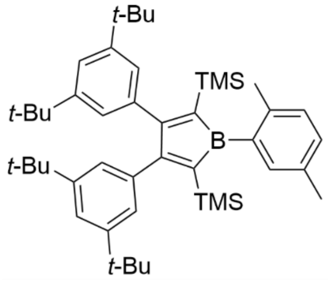

F2 - Acquisition Parameters
Date_ 20220629
Time 19.35 h
INSTRUM spect
PROBHD Z108618_0644 (
PULPROG dept
TD 65536
SOLVENT C6D6
NS 256
DS 8
SWH 32051.281 Hz
FIDRES 0.978127 Hz
AQ 1.0223616 sec
RG 128.09
DW 15.600 usec
DE 6.50 usec
TE 299.8 K
CNST2 7.0000000
D1 3.97763801 sec
D2 0.07142857 sec
D12 0.00002000 sec
TD0 1
SFO1 79.5203972 MHz
NUC1 29Si
P1 9.00 usec
P2 18.00 usec
PLW1 48.97800064 W
SFO2 400.3016012 MHz
NUC2 1H
CNST12 0.2200000
CPDPRG[2 waltz16
P0 3.11 usec
P3 14.12 usec
P4 28.24 usec
PCPD2 90.00 usec
PLW2 10.50000000 W
PLW12 0.25845000 W

F2 - Processing parameters
SI 32768
SF 79.5283500 MHz
WDW EM
SSB 0
LB 1.00 Hz
GB 0
PC 1.40

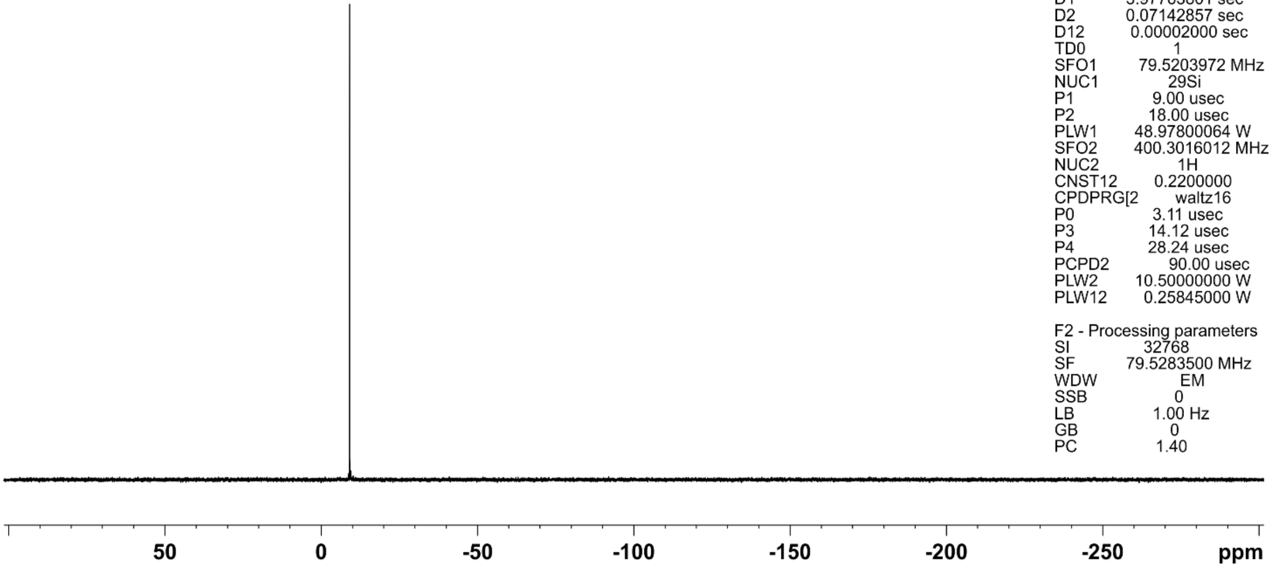

Compound 1a-Xyl

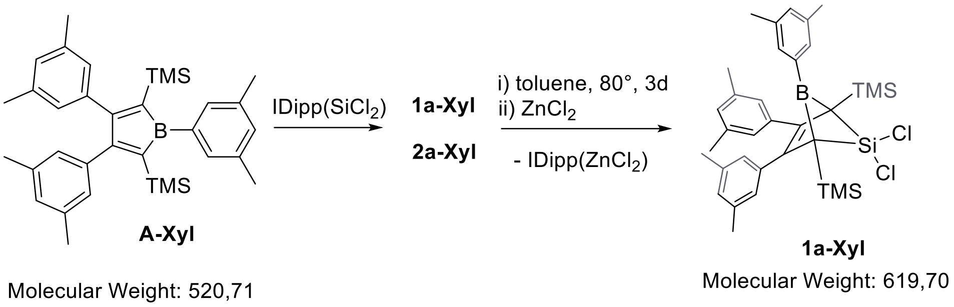

In a glovebox, to a mixture of borole **A-Xyl** (196.1 mg, 0.3766 mmol, 1 eq.) and IDipp-$SiCl_2$ (183.8 mg, 0.3770 mmol, 1 eq.) toluene (2 mL) was added. The immediately formed dark red-to-violet solution of the product mixture that contained **1a-Xyl** and silene-adduct **2a-Xyl** was transferred into a Schlenk-tube and was then left to stir at 80 °C for three days. The toluene was removed from the orange brown mixture under reduced pressure and the tube was transferred into a glovebox. $ZnCl_2$ (140.1 eq.) and toluene (2 mL) were added and the suspension was left to stir over night at ambient temperature. The solvent was removed under reduced pressure and the residue subsequently suspended in hexane (3 mL). The hexane was removed in vacuo to remove toluene leftovers to force precipitation of IDipp($ZnCl_2$) and the residue was extracted into hexane (2 mL). The mixture was filtered through a syringe filter equipped with a thin pad of glass fiber and the insoluble remains in the filter were further extracted with hexane (2 mL, 1 mL). The solvent of the combined hexane extracts was removed in vacuo from the yellow filtrate to give a foamy solid. The foam was dissolved in pentane (3 mL) which was subsequently removed in vacuo to remove solvent traces as good as possible. The product **1a-Xyl** was obtained as a beige, solid powder after thorough drying in vacuo (170.3 mg, 0.2747 mml, 73 %).

Note: The product is very soluble and all our attempts to obtain crystals (from pentane, hexane, benzene, dichloromethane) remained unsuccessful.

Analytical Data for Compound 1a-Xyl

NMR:

^{1}H (400.13 MHz, 297 K, C_6D_6, C_6D_5H at 7.15 ppm): 7.53 (m, 2H, B-Ar: *o-H*), 7.00 (m, 4H, *o-H*), 6.85 (m, 1H, B-Ar: *p-H*), 6.51 (s, 2H, *p-H*), 2.21 (m, 6H, B-Ar: C*H*$_3$), 1.89 (m, 12H, *m*-C*H*$_3$), 0.19 (s, 18H, TMS).

^{13}C{^{1}H} (100.64 MHz, 298 K, C_6D_6 solvent signal at 128.0 ppm): 137.3 (*m-C*-CH$_3$), 136.9 (B-Ar: *m-C*-CH$_3$), 134.8 (*ipso-C*), 134.1 (B-Ar: *o-C*H), 130.3 (B-Ar: *p-C*), 129.9 *(p-C)*, 128.6 (B-Ar: *ipso-C*), 125.9 (C_β), 54.9 (C_α), 21.3 (B-Ar: *m*-CH$_3$), 20.9 (*m*-CH$_3$), 1.3 (TMS), superimposed by solvent signal (*o-C*).

^{11}B (128.37 MHz, 297 K, C_6D_6): -26.7 ($\omega_{1/2}$ = 150.0 Hz).

^{29}Si-INEPT (79.49 MHz, 296 K, C_6D_6): 1.8 ($SiCl_2$), -5.0 (TMS).

Elemental Analysis: ($C_{34}H_{45}BCl_2Si_3$) calcd C 65.90, H 7.32, B 1.74, Cl 11.44, Si 13.6, observed C 68.61, H 7.881. Very poor analysis but best result obtained.

Spectra Plots for Compound 1a-Xyl

1H-NMR-spectrum of compound **1a-Xyl** in C6D6
C6D5H at 7.15 ppm

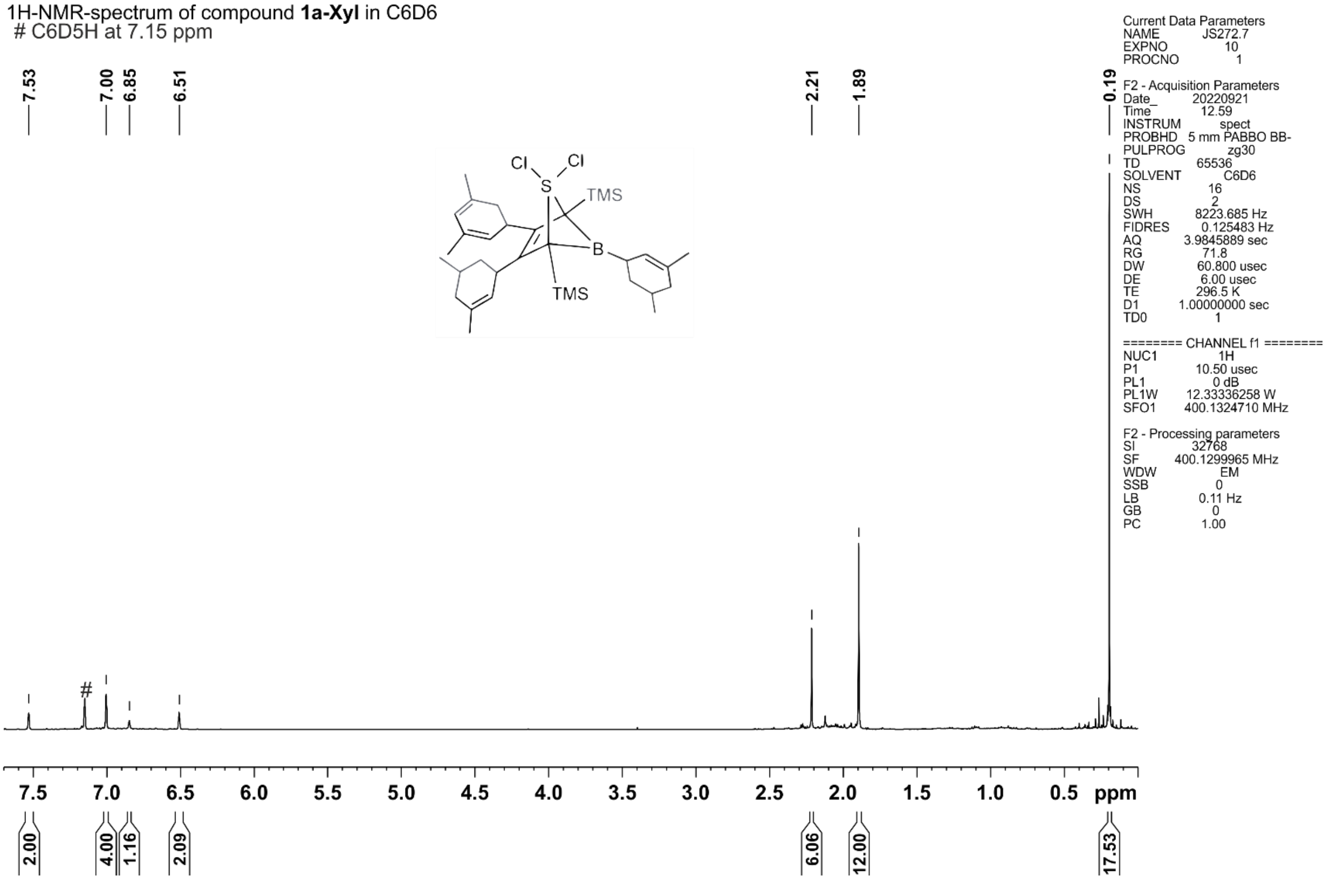

13C{1H}-NMR-spectrum of compound **1a-Xyl** in C6D6
C6D6 at 128.0 ppm

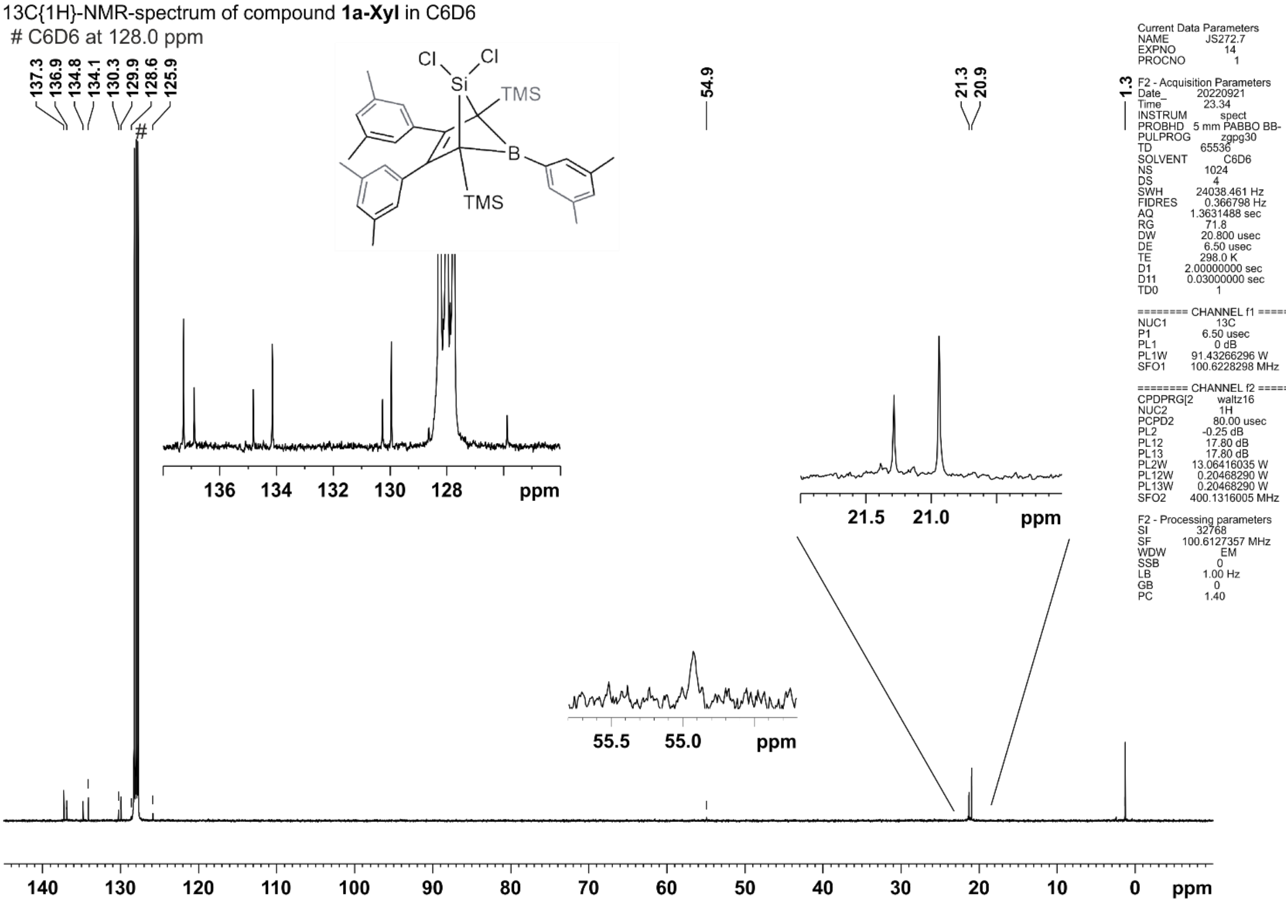

11B-NMR spectrum (background suppressed) of compound **1a-Xyl** in C6D6

Current Data Parameters
NAME JS272.7
EXPNO 11
PROCNO 1

F2 - Acquisition Parameters
Date_ 20220921
Time 13.07
INSTRUM spect
PROBHD 5 mm PABBO BB-
PULPROG zgpgbs_nocpd.gf
TD 3200
SOLVENT C6D6
NS 4000
DS 4
SWH 32051.281 Hz
FIDRES 10.016026 Hz
AQ 0.0499200 sec
RG 912
DW 15.600 usec
DE 8.00 usec
TE 296.6 K
D11 0.03000000 sec
TD0 1

======== CHANNEL f1 ========
NUC1 11B
P1 9.00 usec
P2 18.00 usec
PL1 0 dB
SFO1 128.3776050 MHz

F2 - Processing parameters
SI 8192
SF 128.3776050 MHz
WDW EM
SSB 0
LB 4.00 Hz
GB 0
PC 1.40

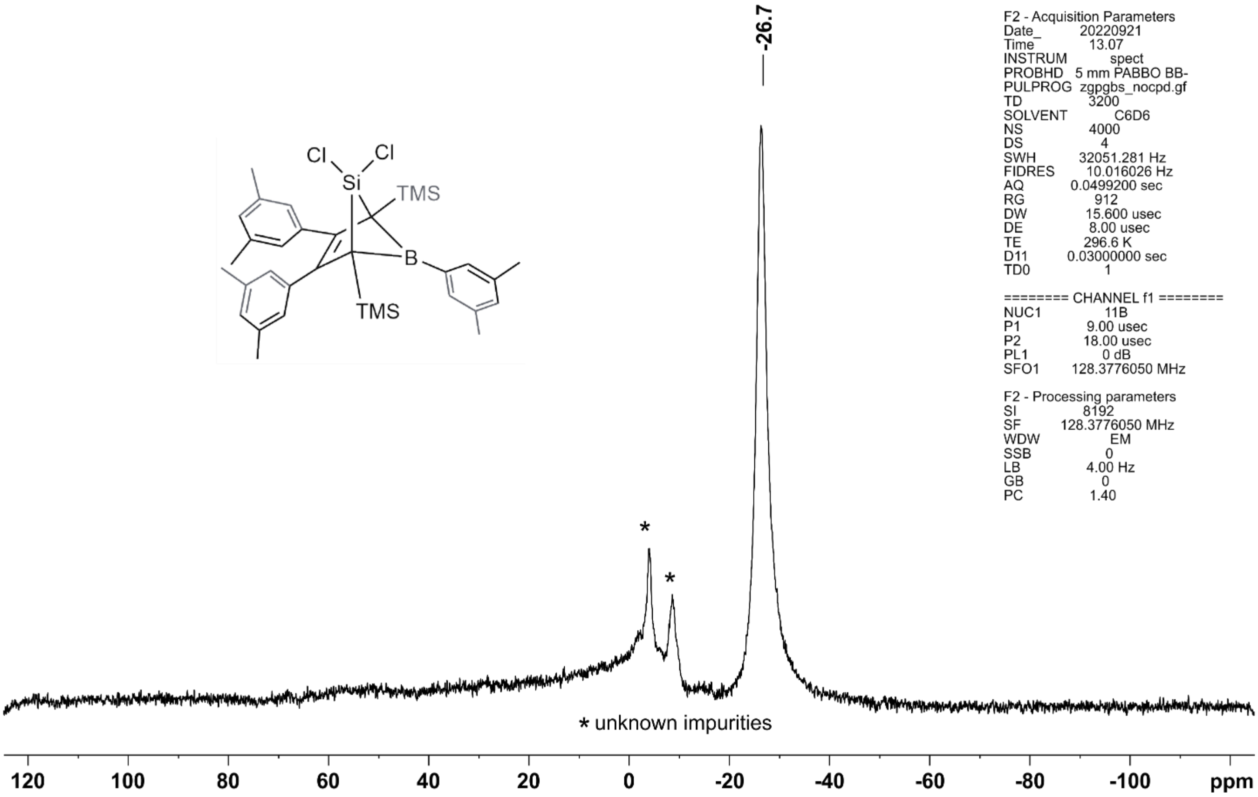

29Si{1H}-NMR spectrum of compound **1a-Xyl** in C6D6

Current Data Parameters
NAME JS272.7
EXPNO 12
PROCNO 1

F2 - Acquisition Parameters
Date_ 20220921
Time 22.30
INSTRUM spect
PROBHD 5 mm PABBO BB-
PULPROG zgig
TD 65536
SOLVENT C6D6
NS 512
DS 0
SWH 32051.281 Hz
FIDRES 0.489064 Hz
AQ 1.0223616 sec
RG 28.5
DW 15.600 usec
DE 6.50 usec
TE 296.4 K
D1 20.00000000 sec
D11 0.03000000 sec
TD0 1

======== CHANNEL f1 ========
NUC1 29Si
P1 6.00 usec
PL1 0 dB
SFO1 79.4866259 MHz

======== CHANNEL f2 ========
CPDPRG[2 waltz16
NUC2 1H
PCPD2 80.00 usec
PL2 -0.25 dB
PL12 17.80 dB
PL2W 13.06416035 W
PL12W 0.20468290 W
SFO2 400.1316005 MHz

F2 - Processing parameters
SI 32768
SF 79.4945750 MHz
WDW EM
SSB 0
LB 1.00 Hz
GB 0
PC 1.40

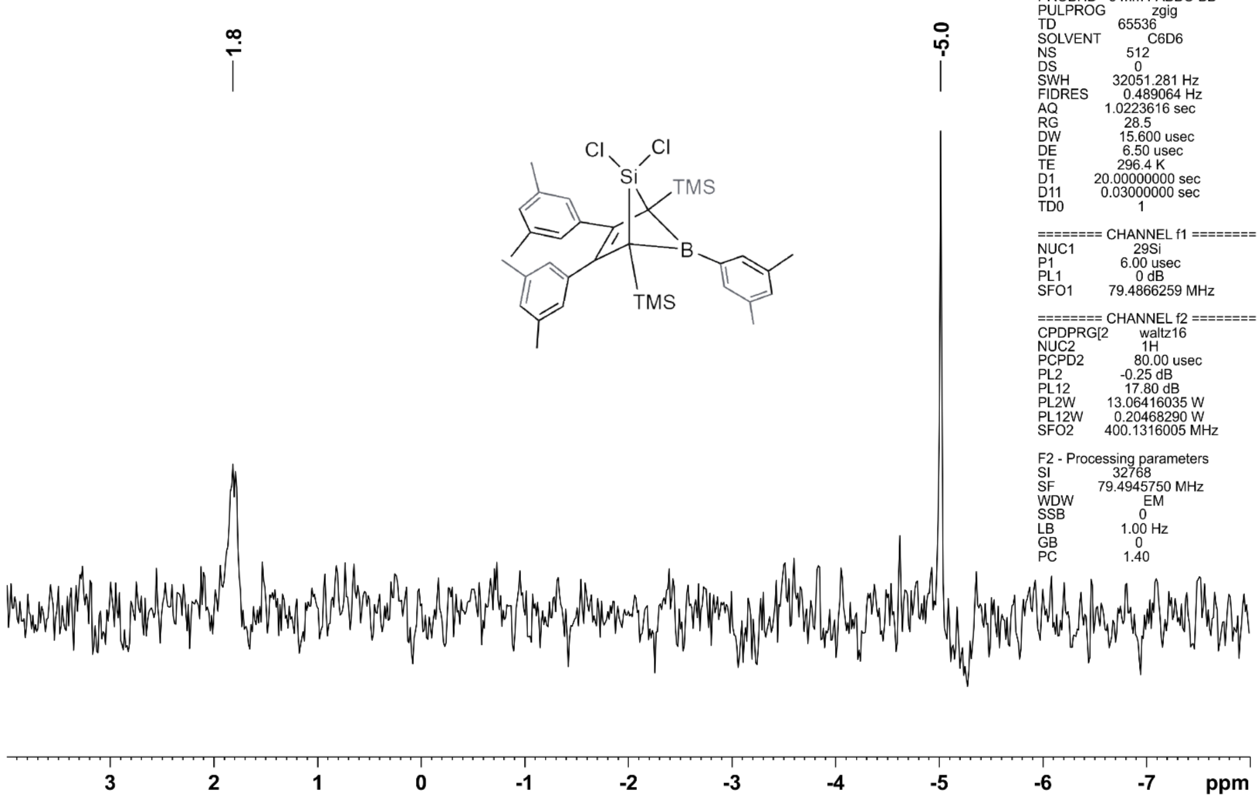

Compound 1a-*p*Xyl

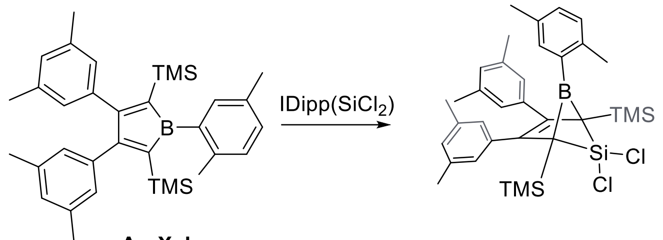

A-*p*Xyl Molecular Weight: 520,71

1a-*p*Xyl Molecular Weight: 619,70

In a glovebox, a solution of borole **A-*p*Xyl** (616 mg, 1.183 mmol, 1 eq.) in toluene (2 mL) was added to a suspension of dichlorosilylene IDipp($SiCl_2$) (675.3 mg, 1.219 mmol, 1.03 eq.) in toluene (12 mL) and the mixture was stirred over night. $ZnCl_2$ (690 mg) was then added to the now red solution and the resulting suspension was then again left to stir over night. The yellow suspension was filtered through a syringe filter equipped with a thin pad of glass fiber and the residue was washed with hexane (1 mL). The solvent of the filtrate was removed under reduced pressure and the resulting solid residue was then extracted three times with hexane (5 mL, 5 mL, 3 mL). The combined extracts were filtered over a glas fiber equpped syringe filter and the solvent was removed from the filtrate under reduced pressure. The residue was washed with hexane (1.5 mL) and dried in vacuo to yield a first crop (198.7 mg). The washing solution was cooled to –30 °C to yield a second crop which was carefully washed with cold hexane (0.1 – 0.2 mL) and dried in vacuo. The product could be obtained as a crystalline, pale yellow to colorless solid (all crops combined: 392.3 mg, 0,6328 mmol, 53 %).

Note: The removal of IDipp with $ZnCl_2$ has was performed as previously reported in the literature[10]

Note: Instead of washing the crude product it can be dissolved in pentane and the solutions are allowed to slowly evaporate. The product crystallizes at the walls of the vial and can be collected.

Analytical Data for Compound 1a-*p*Xyl

NMR:^{1}H (300.13 MHz, 298 K, C_6D_6, C_6D_5H at 7.15 ppm): 7.71 (s, 1H, B-Ar: *o-H*), 7.11 (m, 4H, *o-H*), 7.08 (d, 1H, , ^3J = 7.72 Hz, B-Ar: *m-H*), 6.96 (dd, 1H, , ^3J = 7.72 Hz, ^4J = 1.62 Hz, B-Ar: *p-H*), 6.59 (s, 2H, *p-H*), 2.86 (s, 3H, *o*-C*H*$_3$), 2.20 (s, 3H, B-Ar: , *m*-C*H*$_3$), 1.93 (s, 12H, , *m*-C*H*$_3$), 0.05 (s, 18H, TMS).

^{13}C{^{1}H} (100.64 MHz, 298 K, C_6D_6 solvent signal at 128.0 ppm): 142.0 (B-Ar: *o-C*-CH$_3$), 137.5 (*m-C*-CH$_3$), 135.7 (B-Ar: *o*-*C*H), 135.2 (*ipso-C*), 133.0 (B-Ar: *m-C*-CH$_3$), 130.3 (B-Ar: *m*-*C*H), 130.0 *(p-C)*, 129.3 (B-Ar: *p-C*), 126.8 (C_β), 53.8 (C_α), 23.6 (B-Ar: *o*-*C*H$_3$), 21.0 (*m*-*C*H$_3$), 20.7 (B-Ar: *m*-*C*H$_3$), 0.9 (TMS), not found (B-Ar: *ipso-C*), superimposed by solvent signal (*o-C*).

^{11}B (128.37 MHz, 298 K, C_6D_6): -22.0 ($\omega_{1/2}$ = 170 Hz).

^{29}Si (99.38 MHz, 298 K, C_6D_6): 3.0 ($SiCl_2$), -5.3 (TMS).

Elemental Analysis: ($C_{34}H_{45}BCl_2Si_3$) calcd C 65.90, H 7.32, B 1.74, Cl 11.44, Si 13.6, observed C 65.64, H 7.54.

LIFDI-MS: calcd exact mass: 618.23 m/z, observed m/z: 636.3 $[M+H_2O]^+$

Crystal structure of Compound 1a-*p*Xyl

For further details on the diffraction measurement please see the respective section.

1a-pXyl crystallised from solutions in hexane in a freezer (-40°C).

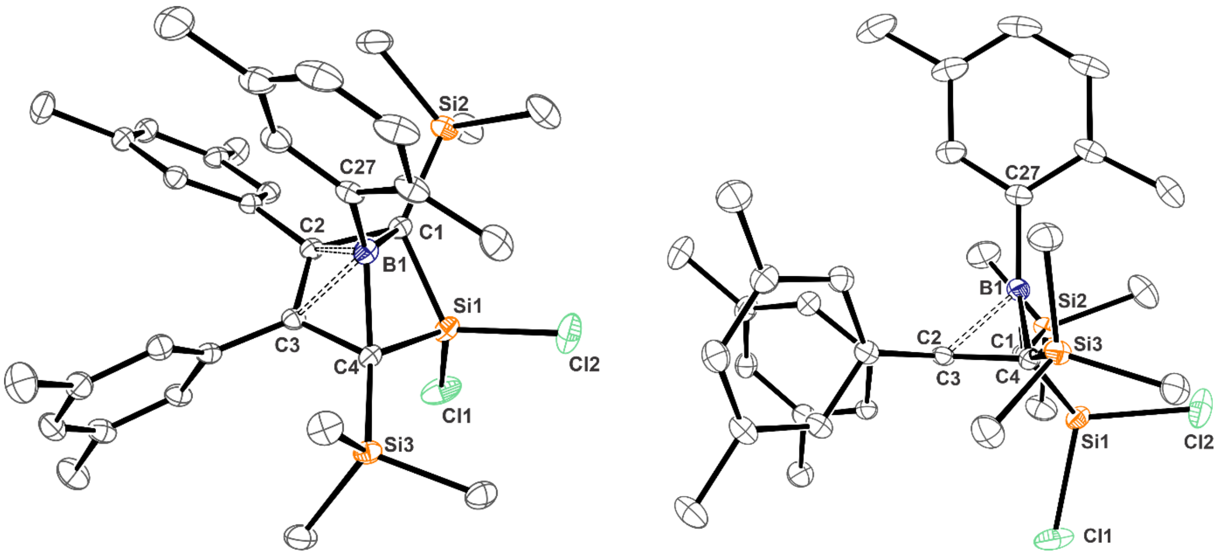

ORTEP plot of the molecular structure of **1a-*p*Xyl**. Atomic displacement parameters are drawn at 50% probability level. A second molecule in the asymmetric unit and hydrogen atoms are omitted for the sake of clarity. Selected bond length in Å: B1-C1 1.693(2), C1-C2 1.494(3), C2-C3 1.398(2), C3-C4 1.502(3), C4-B1 1.674(2), C4-Si1 1.837(2), C1-Si1 1.830(2), C1-Si2 1.874(2), C4-Si3 1.874(2), Si1-Cl1 2.0633(5), Si1-Cl2 2.050(1), B1-Si1 2.358(2). The structure was deposited with the CCSD.

Spectra Plots for Compound 1a-*p*Xyl

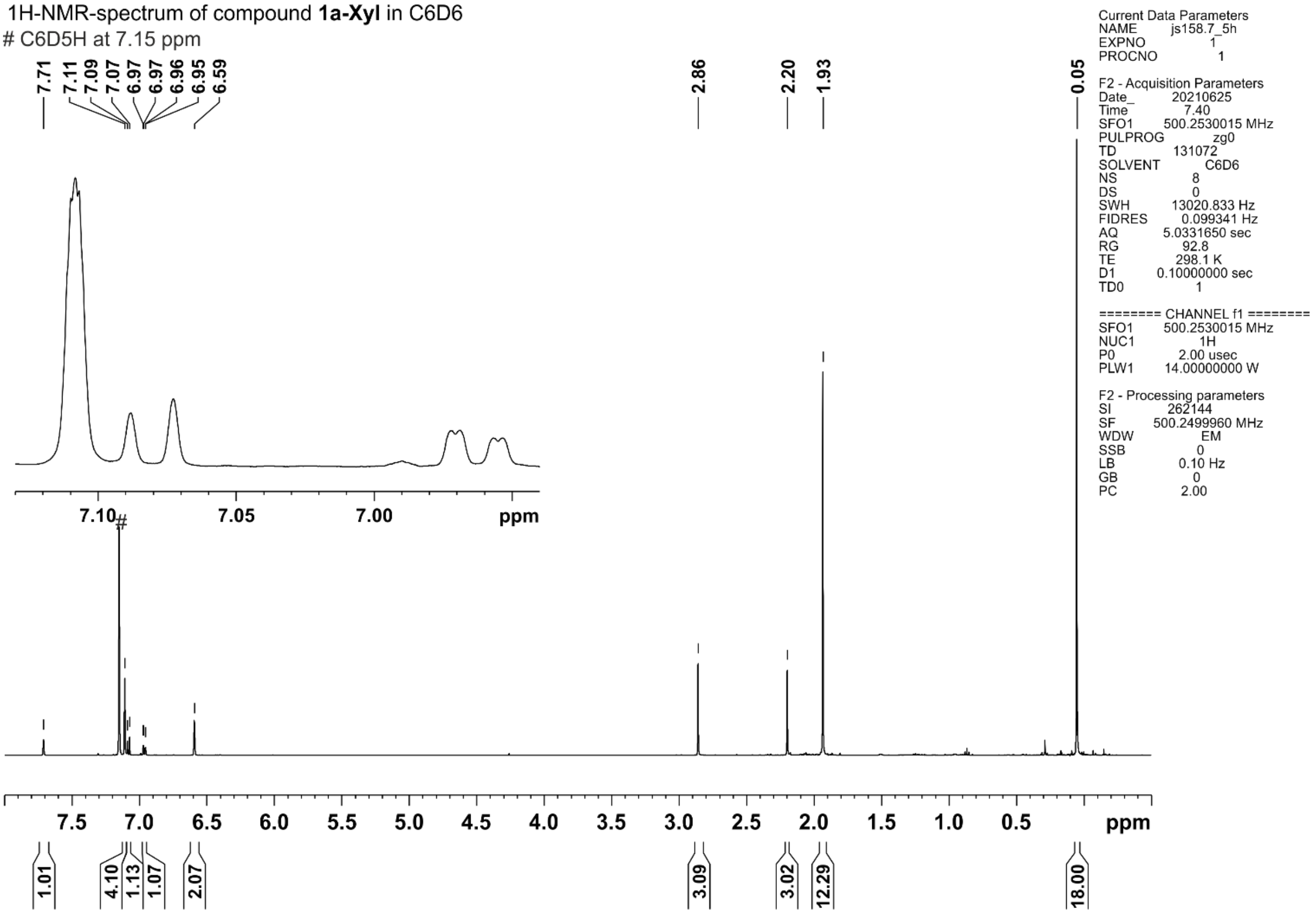

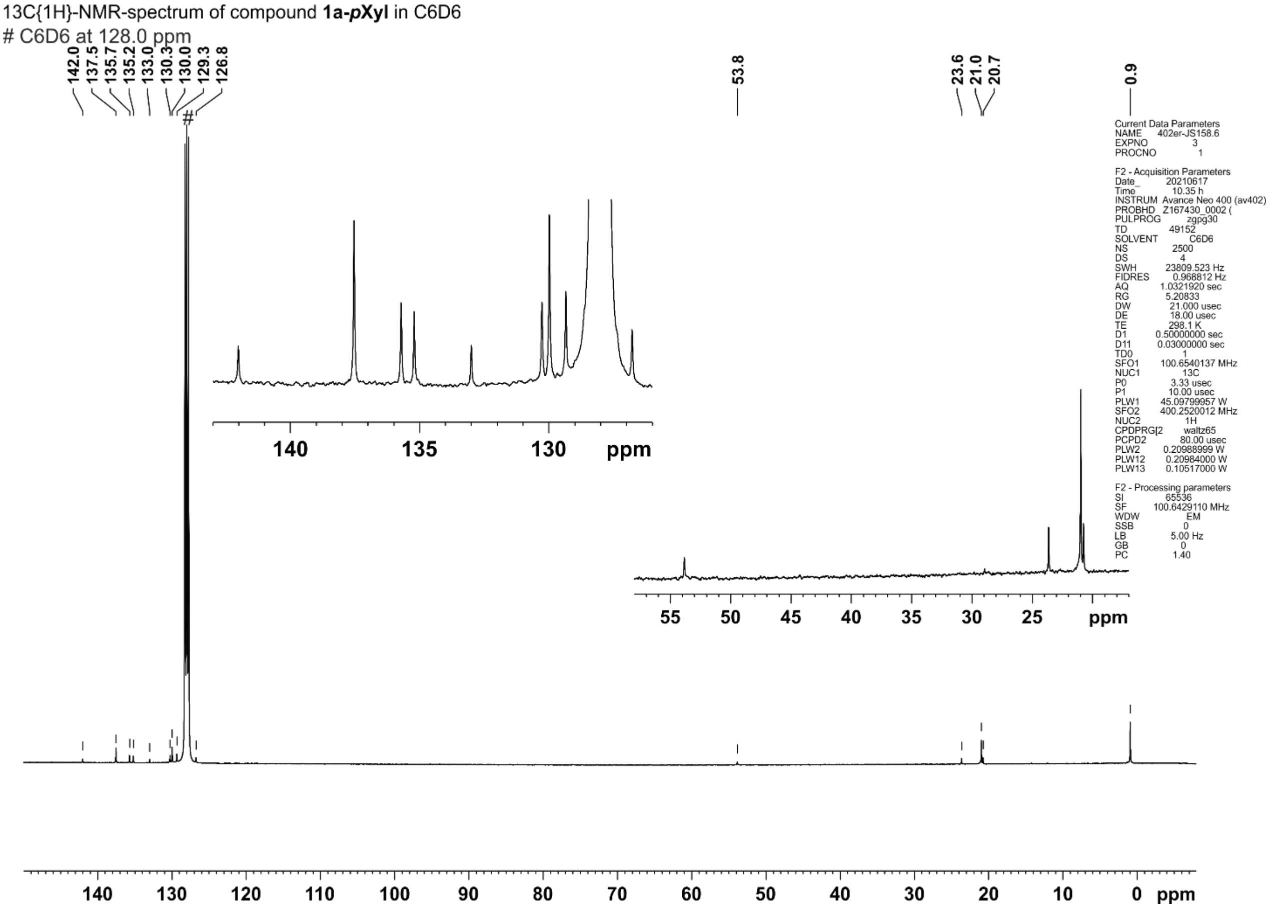

11B-NMR spectrum (background suppressed) of compound **1a-*pXyl*** in C6D6

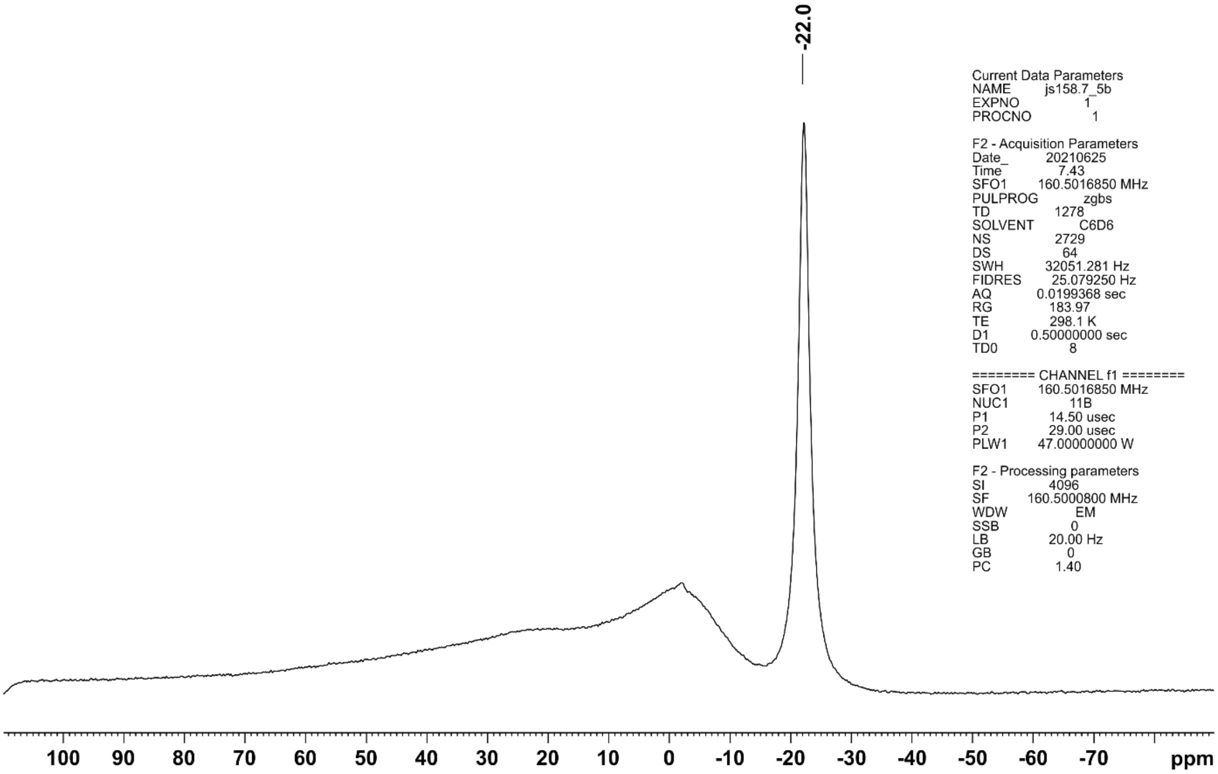

29Si-NMR spectrum of compound **1a-*pXyl*** in C6D6

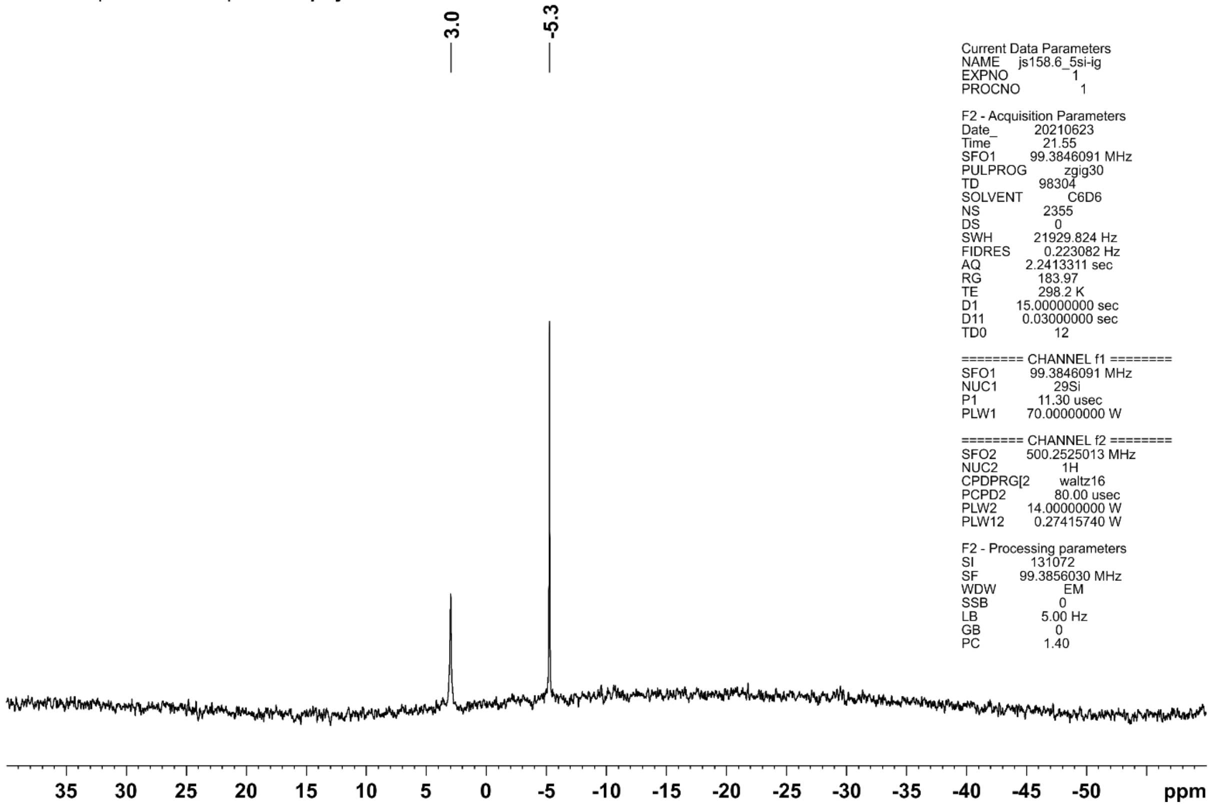

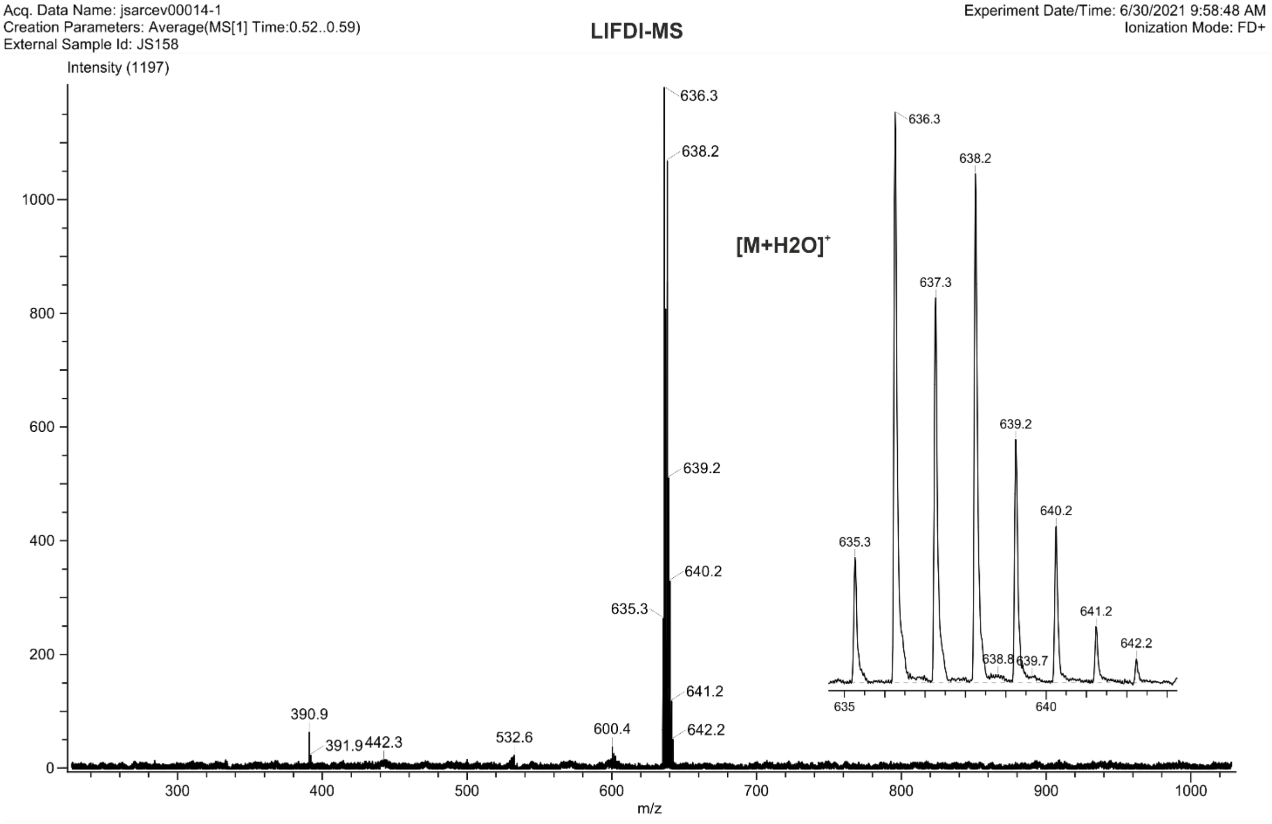

Acq. Data Name: jsarcev00014-1
Creation Parameters: Average(MS[1] Time:0.52..0.59)
External Sample Id: JS158
LIFDI-MS
Experiment Date/Time: 6/30/2021 9:58:48 AM
Ionization Mode: FD+
Intensity (1197)
[M+H2O]+
636.3
638.2
637.3
639.2
640.2
635.3
641.2
642.2
638.8
639.7
390.9
391.9
442.3
532.6
600.4
m/z

Compound 1b-Xyl

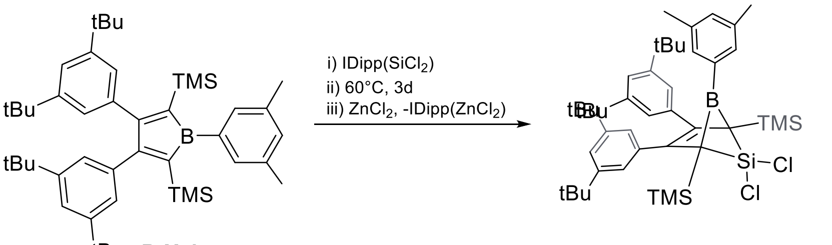

B-Xyl

Molecular Weight: 689,04

1b-Xyl

Molecular Weight: 788,02

In a glovebox, IDipp-$SiCl_2$ (101.8 mg, 0.209 mmol, 1 eq) was placed in a Schlenk flask and a solution of borole **B-Xyl** (143.8 mg, 0.209 mmol, 1 eq) in toluene (5 mL) was added. The resulting dark red solution was stirred at 60 °C for three days, giving a pale red suspension with a beige precipitate. The solvent of the reaction mixture was removed in vacuo and $ZnCl_2$ (31.3 mg, 0.230 mmol, 1.1 eq) and diethyl ether (3 mL) were added. The so obtained pale red suspension was stirred at ambient temperature for two hours. Subsequently, the solvent was removed in vacuo and the solid was extracted into *n*-hexane (3 x 1 mL), filtered through a syringe filter equipped with a pad of glass fiber to yield a pale red extract. The volume of the extract was reduced to approximately 1.5 mL and the solution was stored at –40 °C overnight, leading to the precipitation of a white solid. This solid was isolated, washed with cold *n*-hexane (–40 °C, 3 x 0.2 mL) and dried under reduced pressure to yield compound **1b-Xyl** (105.3 mg, 0.134 mmol, 64 %) as a white solid.

Analytical Data for Compound 1b-Xyl

NMR:

^{1}H (400.13 MHz, 297 K, C_6D_6, CD_5H at 7.15 ppm): 7.55 (m, 2H, *o-H_{Xyl}*), 7.28 (t, $^4J_{HH}$ = 1.8 Hz, 2H, *p-$H_{ar3/4}$*), 7.15 (o-$H_{ar3/4}$, superimposed by the solvent signal), 6.87 (m, 1H, *p-H_{Xyl}*), 2.25 (s, 6H, CH_3), 1.12 (s, 36H, Ar-C(*Me*)$_3$), 0.20 (s, 18H, Si(*Me*)$_3$).

^{13}C{^{1}H} (100.62 MHz, 298 K, C_6D_6, solvent signal at 128.0 ppm): 150.4 (*m-$C_{Ar3/4}$*), 136.9 (*m-C_{Xyl}*), 134.6 (*ipso-$C_{Ar3/4}$*), 134.2 (*o-C_{Xyl}*), 130.2 (*p-C_{Xyl}*), 126.4 (C_β), 124.4 (br s, $\nu_{1/2}$ ≈ 8 Hz, o-$C_{ar3/4}$), 121.8 (*p-$C_{ar3/4}$*), 55.3 (C_α), 34.9 ($Ar_{3/4}$-*C*(CH_3)$_3$), 31.4 ($Ar_{3/4}$-(C(CH_3)$_3$), 21.4 (CH_3), 1.5 (Si(CH_3)$_3$), the signal corresponding to *ipso-C_{Xyl}* could not be found.

^{11}B (128.38 MHz, 297 K, C_6D_6): –26.7 ($\nu_{1/2}$ ≈ 380 Hz).

^{29}Si (inverse gated, 79.49 MHz, 296 K, C_6D_6): 1.0 ($SiCl_2$), –5.0 (TMS).

Elemental Analysis: $C_{46}H_{69}BCl_2Si_3$ calcd C 70.11, H 8.83; observed C 70.32, H 8.93.

Spectra Plots for Compound 1b-Xyl

1H-NMR-spectrum of compound **1b-Xyl** in C6D6
C6D5H at 7.15 ppm

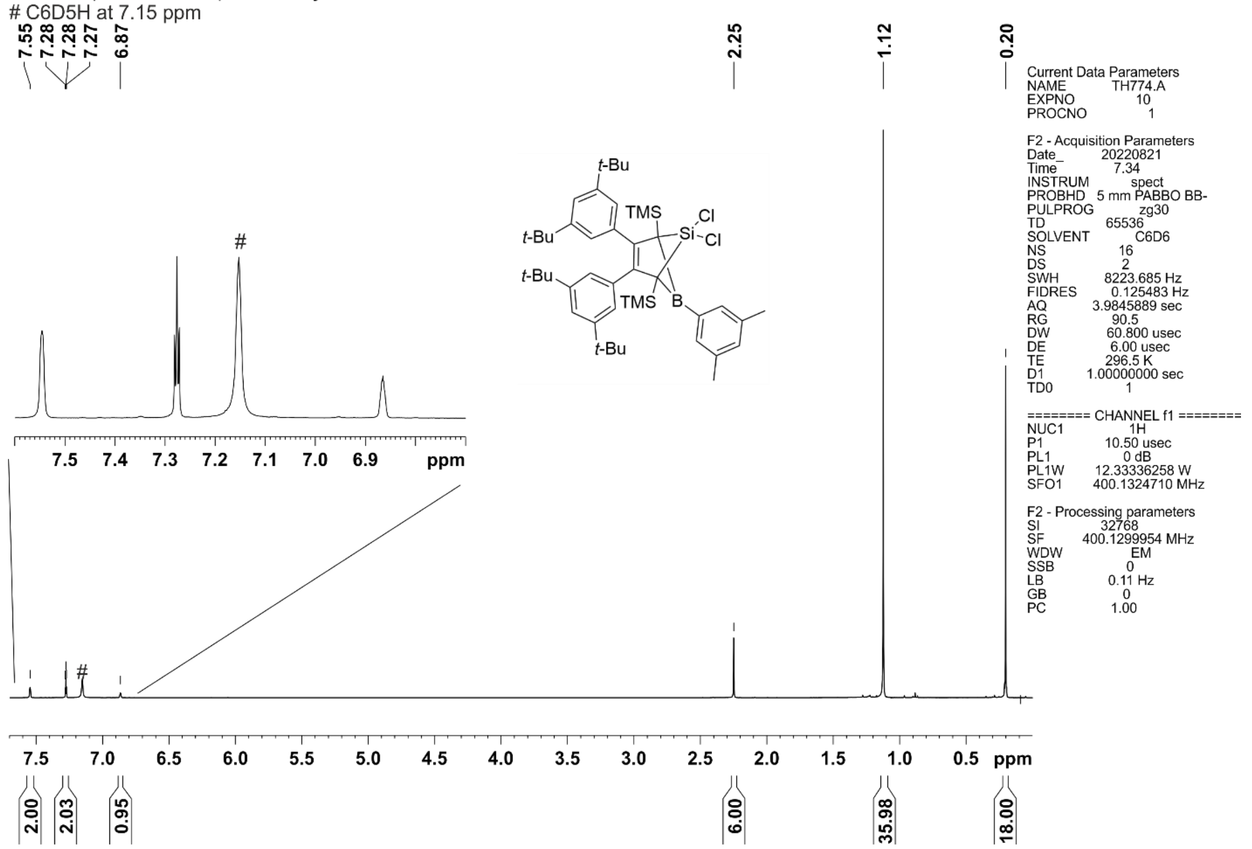

13C{1H}-NMR-spectrum of compound **1b-Xyl** in C6D6
C6D6 at 128.0 ppm

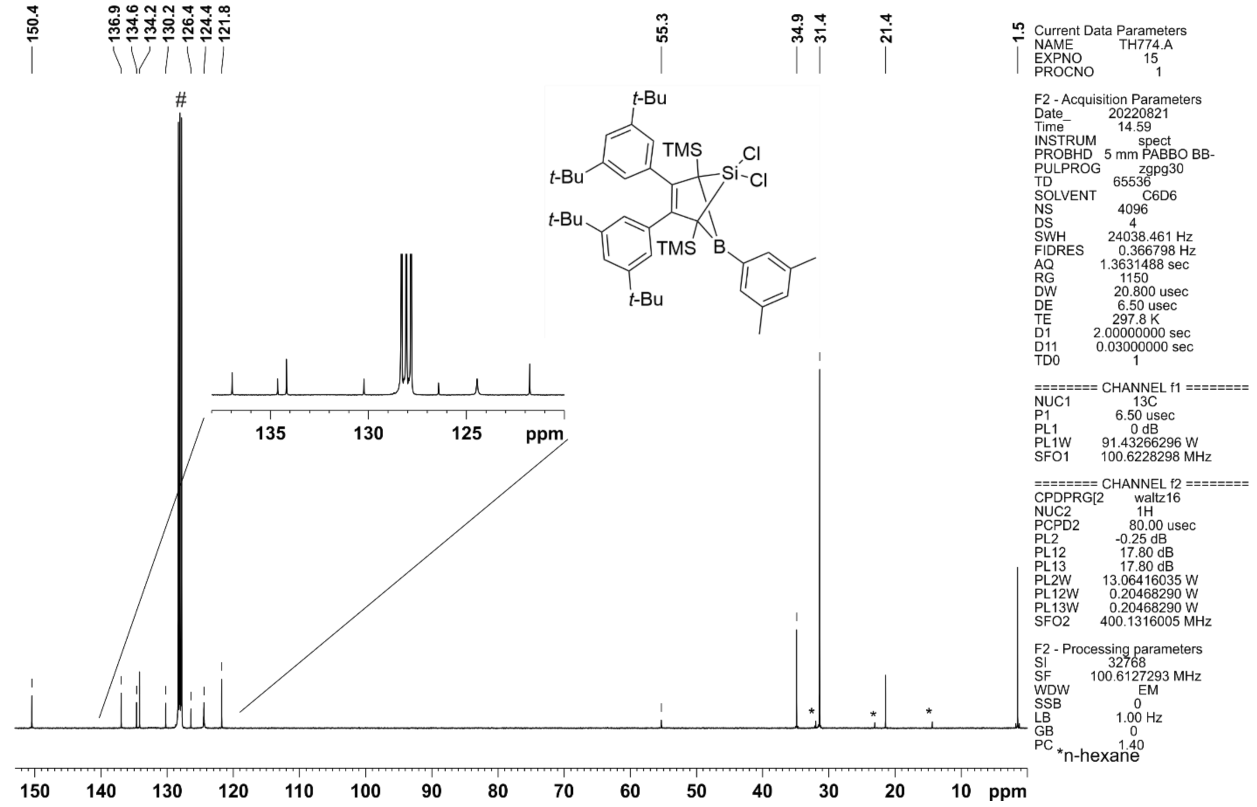

11B-NMR spectrum (background suppressed) of compound **1b-Xyl** in C6D6

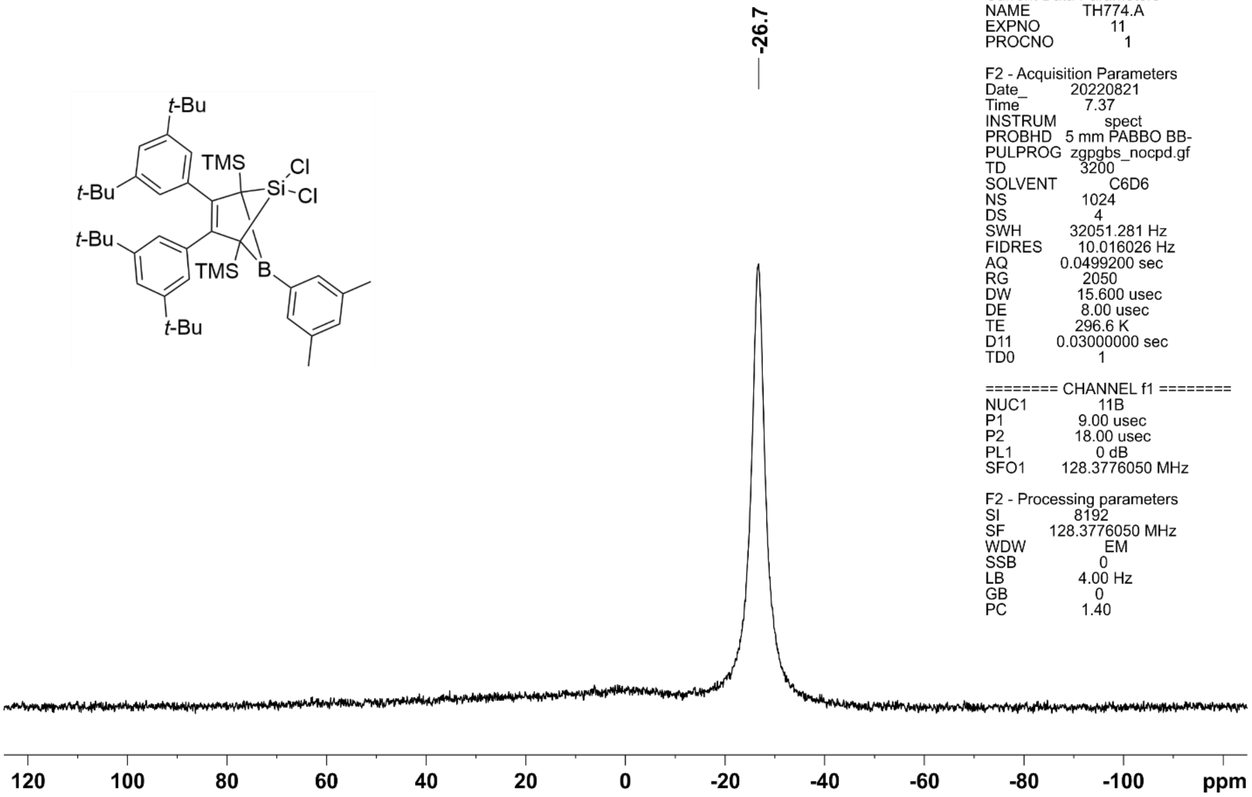

29Si-NMR (inverse gated) spectrum of compound **1b-Xyl** in C6D6

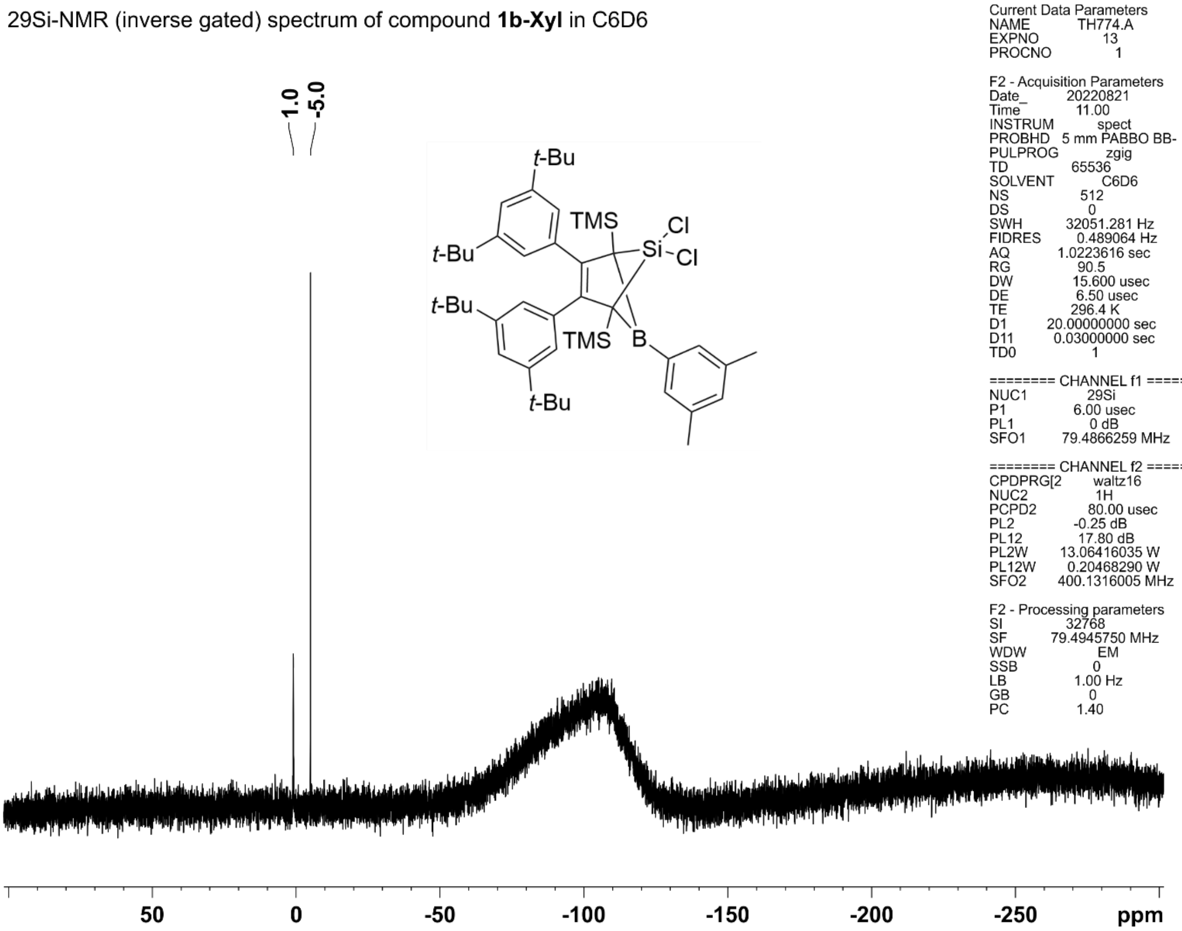

Compound 1b-pXyl

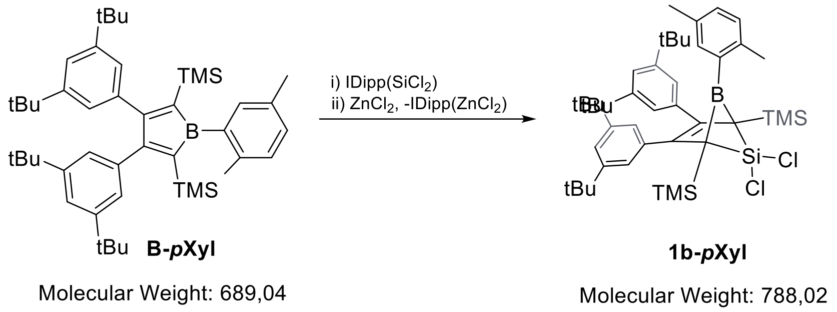

In a glovebox, borole **B-*p*Xyl** (372.2 mg, 0.540 mmol, 1 eq) was dissolved in toluene (10 mL). IDipp-$SiCl_2$ (263.4 mg, 0.540 mmol, 1 eq) was added to the red solution and the reaction was stirred for two hours at ambient temperature. Subsequently, a solution of $ZnCl_2$ (81.0 mg, 0.594 mmol, 1.1 eq) in diethyl ether (2 mL) was added, resulting in a yellowish solution and the precipitation of a beige solid. The suspension was stirred at ambient temperature overnight. The solvent of the reaction mixture was removed under reduced pressure and the solid was extracted into *n*-hexane (4 x 4 mL) and the extracts were filtered through a pad of glass fiber, giving a yellow filtrate. The solvent of the filtrate was removed in vacuo and the resulting solid residue was washed with small amounts of *n*-hexane (3 x 0.5 mL) until a white solid was obtained. This solid was dried under reduced pressure to obtain compound **1b-*p*Xyl** (209.7 mg, 0.266 mmol, 49 %) as a white solid.

Analytical Data for Compound 1b-*p*Xyl

NMR:

^{1}H (400.13 MHz, 297 K, C_6D_6, CD_5H at 7.15 ppm): 7.72 (d, $^{3}J_{HH}$ = 1.5 Hz, 1H, *o-H$_{Xyl}$*), 7.35 (t, $^{4}J_{HH}$ = 1.8 Hz, 2H, *p-H$_{ar3/4}$*), 7.28 (d, $^{4}J_{HH}$ = 1.8 Hz, 4H, *o-H$_{ar3/4}$*), 7.10 (d, $^{3}J_{HH}$ = 7.7 Hz, 1H, *m-H$_{Xyl}$*), 6.99 (dd, $^{3}J_{HH}$ = 7.7 Hz, $^{4}J_{HH}$ = 1.5 Hz, 1H, *p-H$_{Xyl}$*), 2.88 (s, 3H, o-C*H$_3$*), 2.28 (s, 3H, m-C*H$_3$*), 1.16 (s, 36H, Ar-C(*Me*)$_3$), 0.07 (s, 18H, Si(*Me*)$_3$).

^{13}C{^{1}H} (100.65 MHz, 298 K, C_6D_6, solvent signal at 128.0 ppm): 150.7 (*m-C$_{Ar3/4}$*), 142.2 (*o-C$_{Xyl}$*-CH$_3$), 135.4 (*o*-CH$_{Xyl}$), 134.9 (*ipso-C$_{Ar3/4}$*), 133.2 (*m-C$_{Xyl}$*-CH$_3$), 130.4 (*m*-CH$_{Xyl}$), 129.5 (*p*-CH$_{Xyl}$), 127.7 (*C$_\beta$*, partially superimposed by solvent signal, assigned via HMBC), 124.5 (o-*C$_{ar3/4}$*), 121.9 (*p-C$_{ar3/4}$*), 53.3 (*C$_\alpha$*), 35.0 (Ar$_{3/4}$-*C*(CH$_3$)$_3$), 31.5 (Ar$_{3/4}$-(C(*C*H$_3$)$_3$), 23.8 (*o*-CH$_3$), 21.2 (*m*-CH$_3$), 1.0 (Si(CH$_3$)$_3$), the signal corresponding to *ipso-C$_{Xyl}$* could not be found.

^{11}B (128.43 MHz, 297 K, C_6D_6): –22.4 ($\nu_{1/2} \approx$ 400 Hz).

^{29}Si (inverse gated, 79.49 MHz, 297 K, C_6D_6): 3.0 ($SiCl_2$), –5.2 (TMS).

Elemental Analysis: $C_{46}H_{69}BCl_2Si_3$ calcd C 70.11, H 8.83; observed C 69.87, H 8.93.

Spectra Plots for Compound 1b-*p*Xyl

1H-NMR-spectrum of compound **1b-*p*Xyl** in C6D6
C6D5H at 7.15 ppm

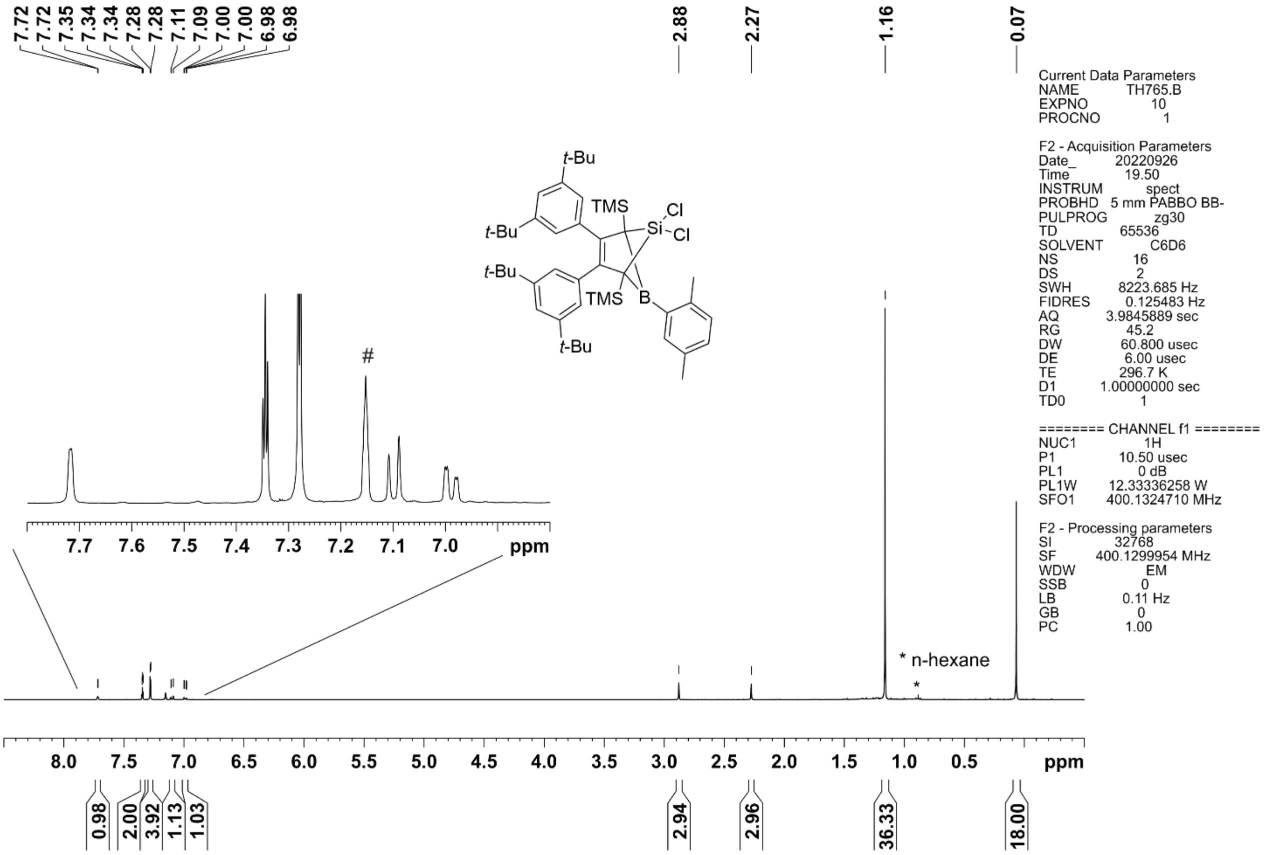

13C{1H}-NMR-spectrum of compound **1b-*p*Xyl** in C6D6
C6D6 at 128.0 ppm

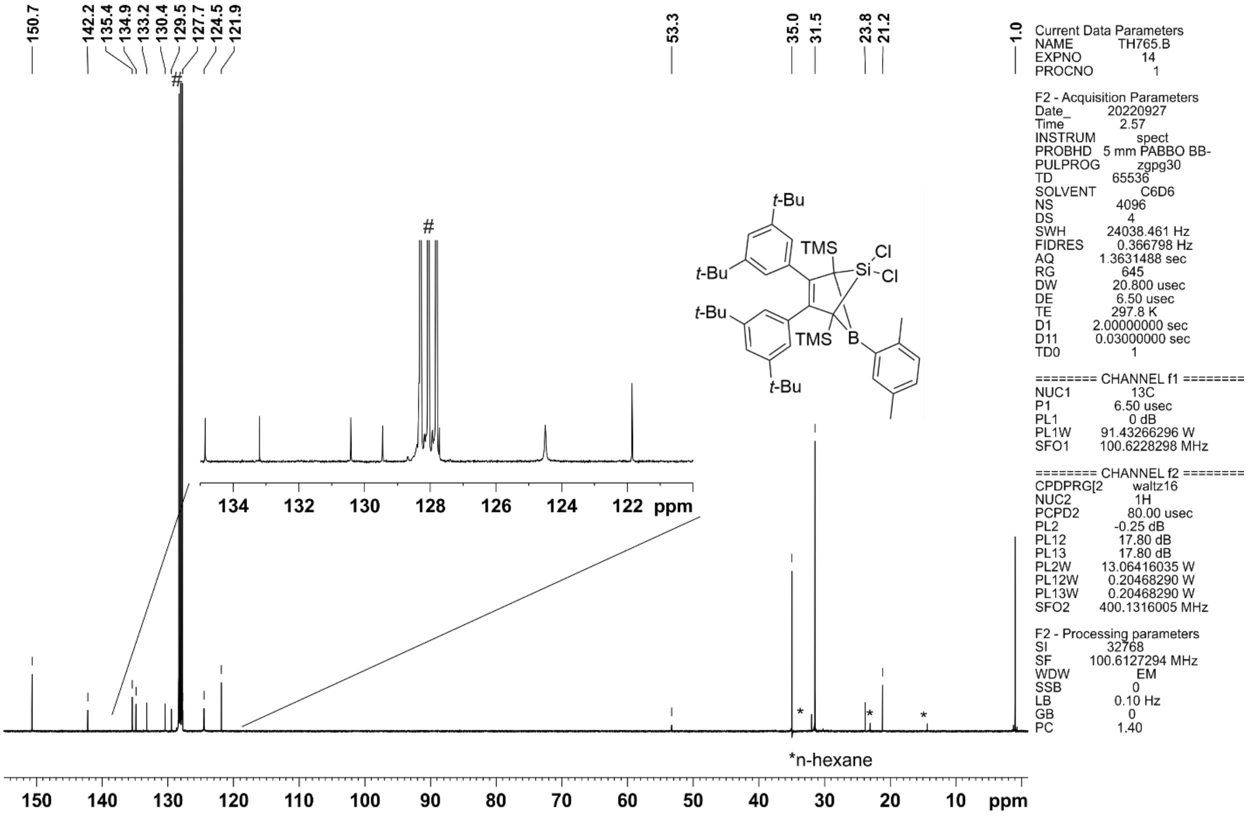

11B-NMR spectrum (background suppressed) of compound **1b-*p*Xyl** in C6D6

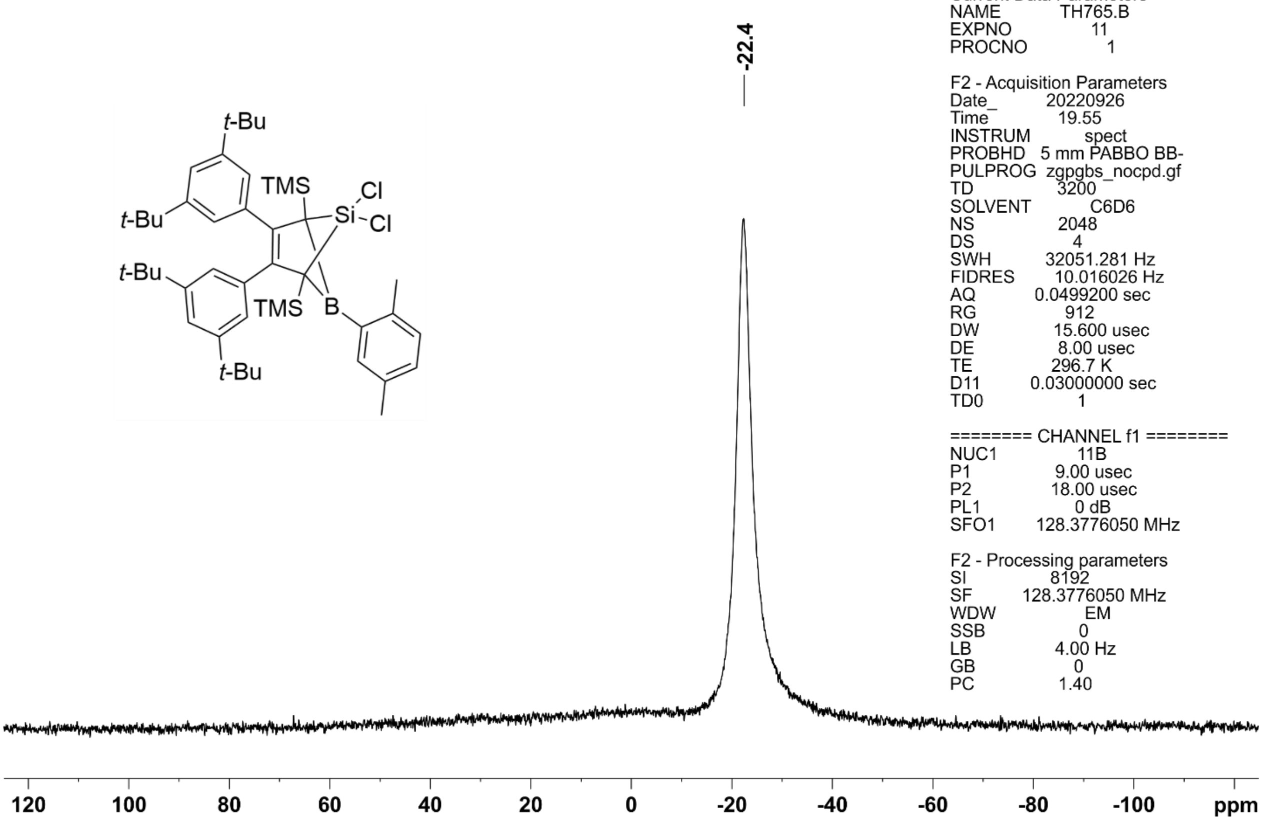

29Si-NMR (inverse gated) spectrum of compound **1b-*p*Xyl** in C6D6

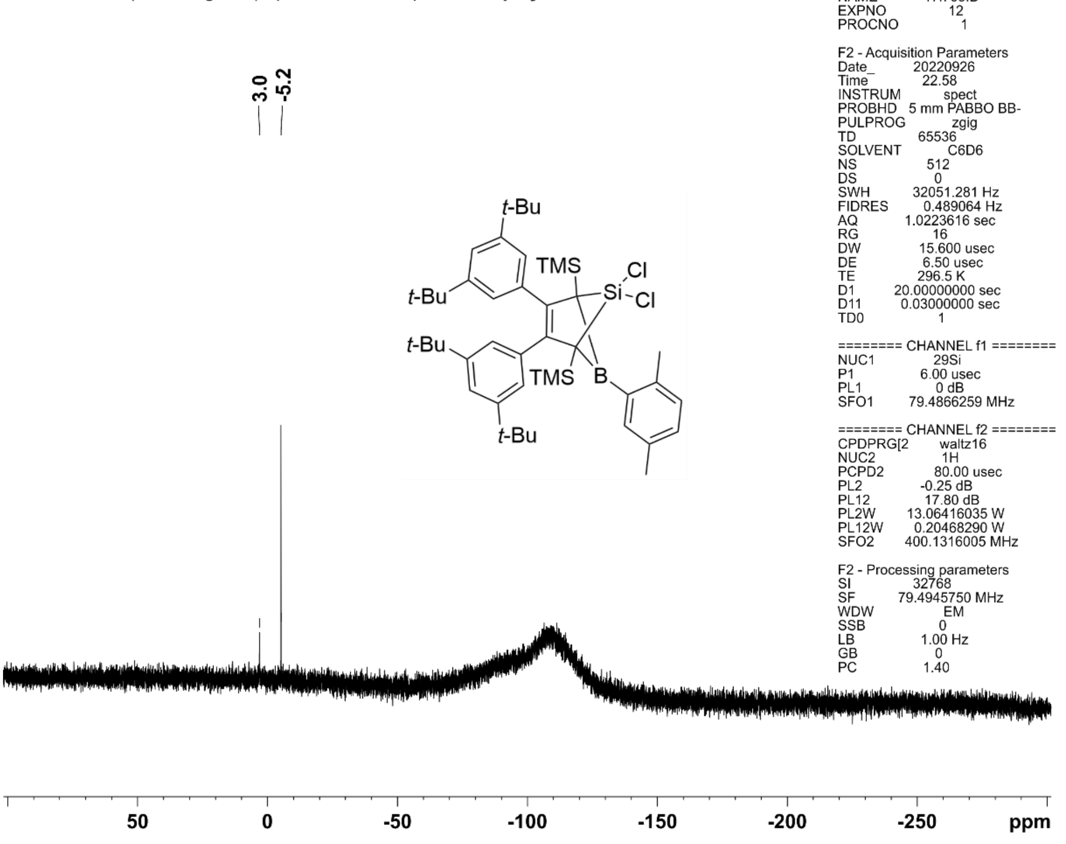

Compounds 1b-Ph* and 2b-Ph*

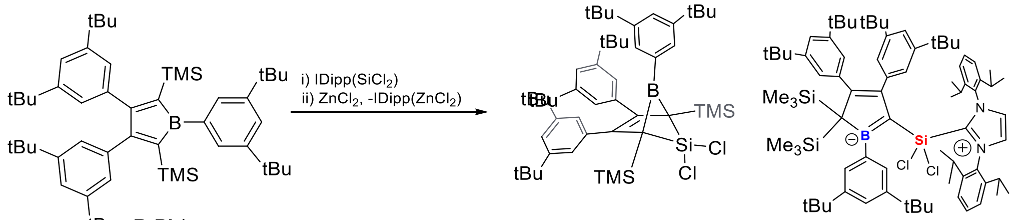

B-Ph* Molecular Weight: 773,20

1b-Ph* Molecular Weight: 872,19

2b-Ph* (only X-ray) Molecular Weight: 1259,78

In a glovebox, IDipp-$SiCl_2$ (121.0 mg, 0.248 mmol, 1 eq) was added to a red solution of borole **B-Ph*** (191.9 mg, 0.248 mmol, 1 eq) in toluene (5mL) and the resulting dark red solution was stirred at ambient temperature. After 30 minutes a solution of $ZnCl_2$ (37.2 mg, 0.273 mmol, 1.1 eq) in diethyl ether (2 mL) was added, whereupon a white solid precipitated and a pale red solution was formed. After stirring the reaction mixture at ambient temperature for three hours, the solvent was removed under reduced pressure. The obtained solid was extracted into *n*-hexane (4 x 1 mL) to give a red extract solution and the extracts were passed through a syringe filter equipped with a pad of glass fiber. The solvent of the filtrate was removed under reduced pressure and the resulting red solid (213.7 mg) was dissolved in a minimal amount of THF (ca. 0.2 mL) and was left to stand at ambient temperature overnight. The so formed red crystals were isolated, washed with cold *n*-hexane (–40 °C, 3 x 0.1 mL) and dried under reduced pressure to presumably yield compound **2b-Ph*** as a red powder (26.6 mg). The mother liquor and washing phase were combined and the solvents were removed under reduced pressure to yield compound **1b-Ph*** (172.7 mg, 0.198 mmol, 80 %) as a off-white to pale green solid in a purity of ca. 90 % (based on ^{1}H-NMR spectroscopy).

Note: Due to solubility the isolation of pure **1b-Ph*** is tedious. In case the first crystallisation attempt from THF did not yield any red crystals, the solution was left to stand at ambient temperature in an open vial until incipient crystallisation is observed. Two drops of THF are added to dissolve the freshly formed crystals and the vial is closed and stored overnight to allow for formation of red crystals. Crystallisation attempts at –40 °C were unsuccessful, because at low temperature a viscous oil is formed from which no crystal formation was observed. Therefore, all crystallisation attempts were performed with saturated solutions at ambient temperature.Further purification of compound **1b-Ph*** can be achieved by crystallisation of the crude product from a saturated *n*-hexane solution (ca. 0.1 mL) at ambient temperature. This way compound **1b-Ph*** can be obtained as a colourless, crystalline solid. However, crystallisation only yields small amounts of pure compound as the crystals have to be thoroughly washed with cold *n*-hexane to remove the viscous mother liquor.

Note: From crude product mixtures **1b-Ph*/2b-Ph*** also single cristals of brick red-orange **2b-Ph*** were isolated and crystallographically investigated. However solution-based analytical examination did not provide meaningful data on the structure as NMR only gave broad signals indicating formation of mixtures and equilibria.

Analytical Data for Compound 1b-Ph*

NMR:

^{1}H (400.30 MHz, 299 K, C_6D_6, CD_5H at 7.15 ppm): 7.72 (d, $^4J_{HH}$ = 1.9 Hz, 2H, *o-H$_{ar1}$*), 7.57 (t, $^4J_{HH}$ = 1.9 Hz, 1H, *p-H$_{ar1}$*), 7.32 (t, $^4J_{HH}$ = 1.8 Hz, 2H, *p-H$_{ar3/4}$*), 7.17 (d, $^4J_{HH}$ = 1.8 Hz, 4H, *o-H$_{ar3/4}$*), 1.40 (s, 18H, Ar_1-C(*Me*)$_3$), 1.12 (s, 36H, $Ar_{3,4}$-C(*Me*)$_3$), 0.22 (s, 18H, Si(*Me*)$_3$).

^{13}C{^{1}H} (100.67 MHz, 300 K, C_6D_6, solvent signal at 128.0 ppm): 150.5 (*m-C$_{Ar3,4}$*), 150.2 (*m-C$_{Ar1}$*), 134.6 (*ipso-C$_{Ar3,4}$*), 130.2 (o-*C$_{ar1}$*), 126.7 (*C$_\beta$*), 124.4 (o-*C$_{ar3,4}$*), 122.8 (*p-C$_{ar1}$*), 122.2 (*p-C$_{ar3,4}$*), 54.8 (*C$_\alpha$*), 35.2 (Ar_1-*C*(CH_3)$_3$), 34.9 ($Ar_{3/4}$-*C*(CH_3)$_3$), 32.0 (Ar_1-(C(*C*H_3)$_3$), 31.5 ($Ar_{3,4}$-(C(*C*H_3)$_3$), 1.5 (Si(*C*H_3)$_3$), the signal corresponding to *ipso-C$_{Ar1}$* was not observed.

^{11}B (128.43 MHz, 299 K, C_6D_6): –25.3 ($\nu_{1/2}$ ≈ 450 Hz).

^{29}**Si** (inverse gated, 79.52 MHz, 299 K, C_6D_6): 1.5 ($SiCl_2$), –5.1 (TMS).

Elemental Analysis: $C_{52}H_{81}BCl_2Si_3$ calcd C 71.61, H 9.36; observed C 71.44, H 9.32.

Crystal structure of Compound 1b-Ph* and 2b-Ph*

For further details on the diffraction measurement please see the respective section.

1b-Ph* crystallised from saturated solutions in hexane. From crude product mixtures **1b-Ph*/2b-Ph*** also single cristals of brick red-orange **2b-Ph*** were isolated and crystallographically investigated.

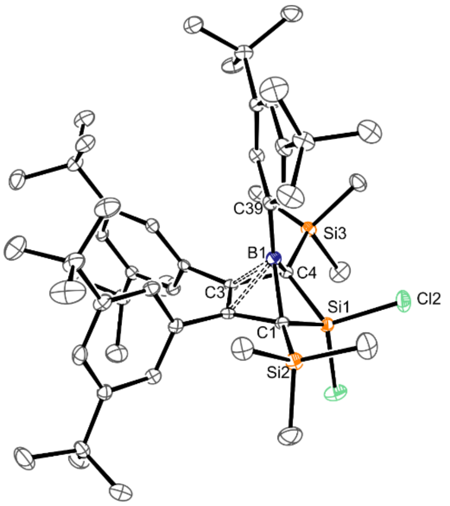

ORTEP plot of the molecular structure of **1b-Ph***. Atomic displacement parameters are drawn at 50% probability level. A second molecule in the asymmetric unit and hydrogen atoms are omitted for the sake of clarity. Selected bond length in Å: B1-C1 1.694(4), C1-C2 1.496(3), C2-C3 1.411(3), C3-C4 1.493(3), C4-B1 1.702(3), C4-Si1 1.836(2), C1-Si1 1.842(2), C1-Si2 1.881(2), C4-Si3 1.873(2), B1-C2 1.783(4), B1-C3 1.803(3), B1-Si1 2.325(2). The structure was deposited with the CCSD.

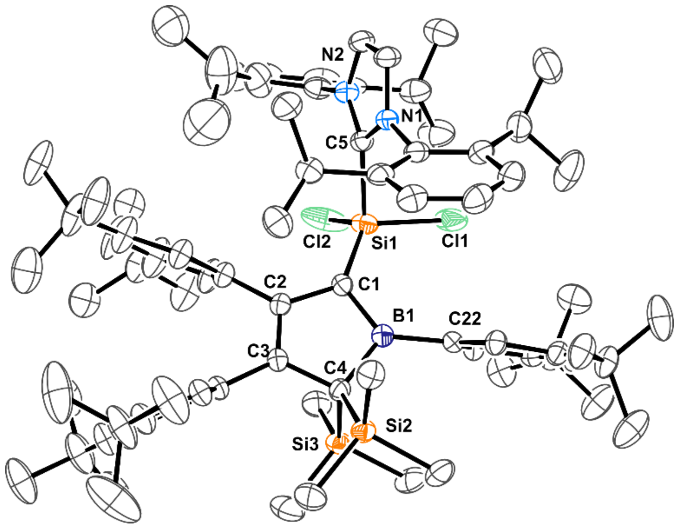

ORTEP plot of the molecular structure of **2b-Ph***. Atomic displacement parameters are drawn at 50% probability level. Hydrogen atoms and disordered t-Bu groups are omitted for the sake of clarity. Selected bond length in Å: B1-C1 1.515(4), C1-C2 1.481(4), C2-C3 1.365(4), C3-C4 1.531(4), C4-B1 1.616(4), C4-Si2 1.903(3), C4-Si3 1.923(3), B1-C22 1.594(4), C1-Si1 1.776(3), Si1-C5 1.963(3), Si1-Cl1 2.055(1), Si1-Cl2 2.072(1). The structure was deposited with the CCSD.

Spectra Plots for Compound 1b-Ph*

1H-NMR-spectrum of compound **1b-Ph*** in C6D6

C6D5H at 7.15 ppm

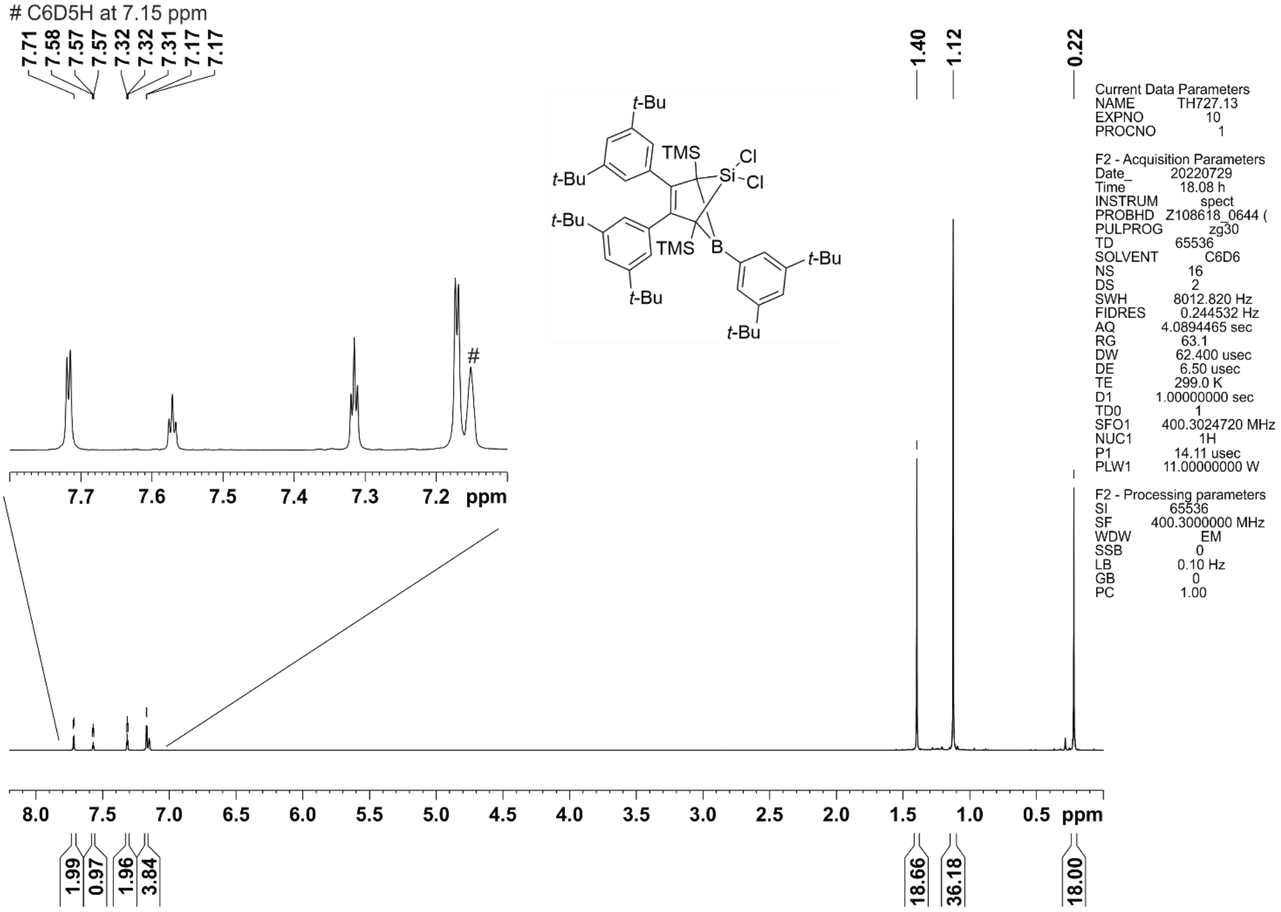

13C{1H}-NMR-spectrum of compound **1b-Ph*** in C6D6

C6D6 at 128.0 ppm

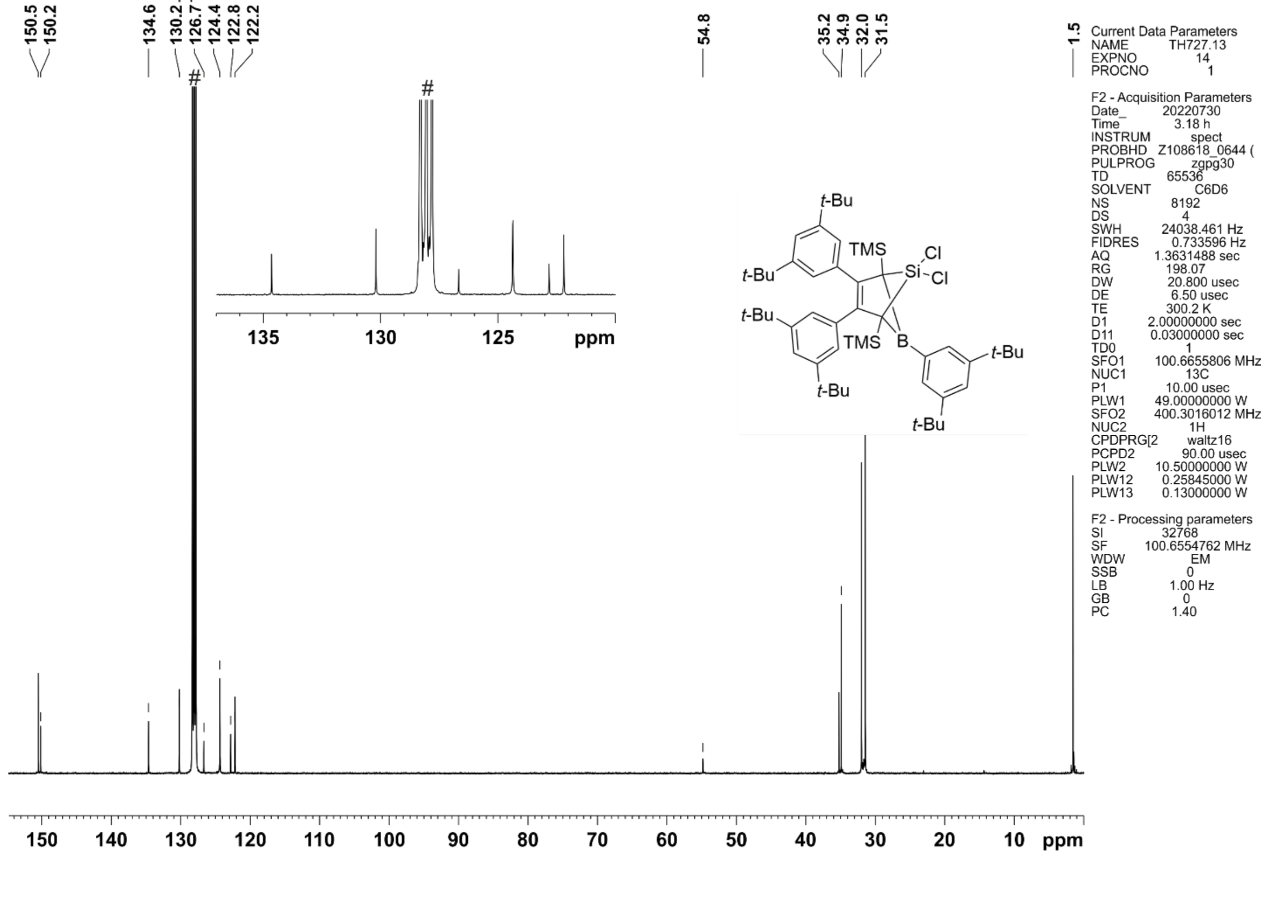

11B-NMR spectrum (background suppressed) of compound **1b-Ph*** in C6D6

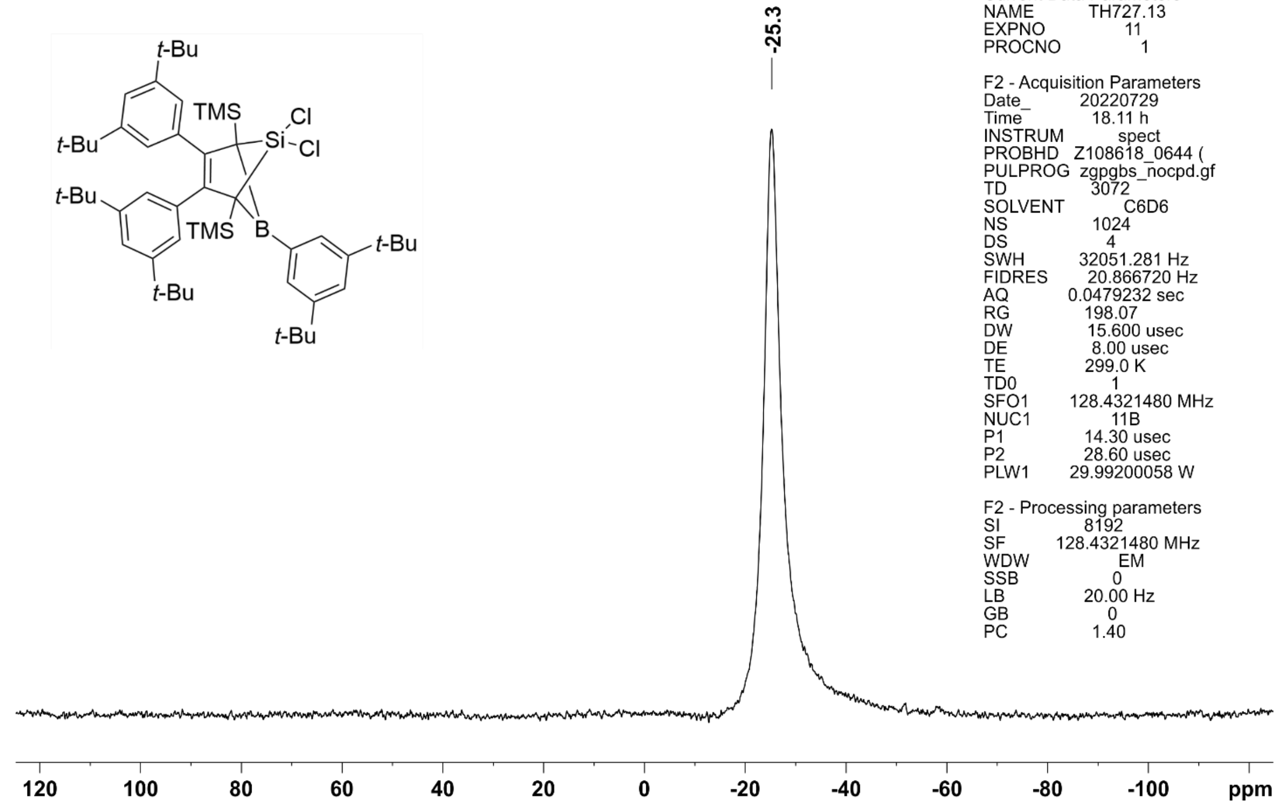

29Si-NMR (inverse gated) spectrum of compound **1b-Ph*** in C6D6

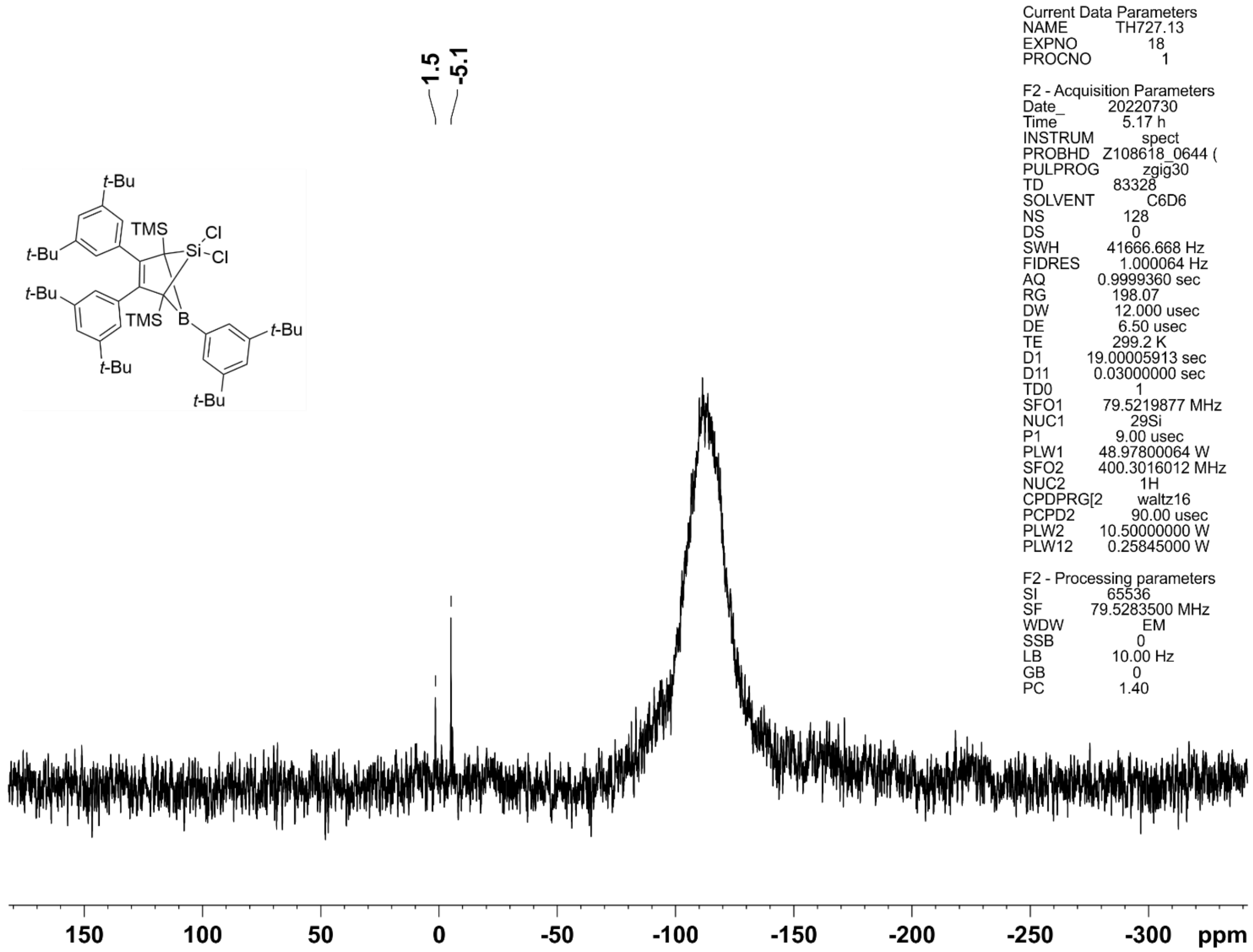

Compound 2a-Me

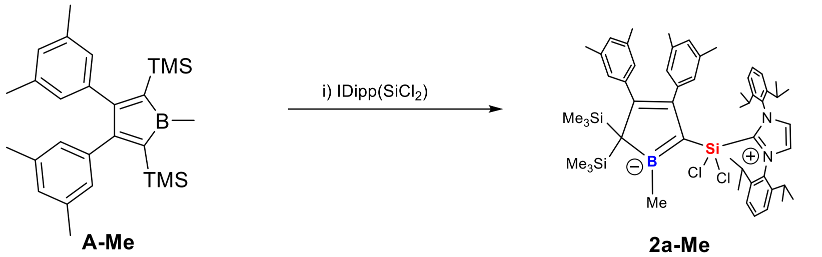

A-Me Molecular Weight: 430,59

2a-Me Molecular Weight: 918,17

A yellow solution of IDipp($SiCl_2$) (145.3 mg, 0.2980 mmol, 1 eq.) in benzene (5 mL, SPS grade) was added to a solution of methylborole **A-Me** (128.7 mg, 0.2975 mmol, 1 eq.) in benzene (2 mL, SPS grade). The dark violet solution was left to stir over night at ambient temperature. The benzene was removed under reduced pressure, resulting in a violet, crystalline solid which was then washed with benzene (1 mL). The product was obtained in form of a dark violet, crystalline material (120.8 mg, 0.132 mmol, 44 %).

Analytical Data for Compound 2a-Me

NMR:

^{1}H (300.13 MHz, 298 K, C_6D_6, C_6D_5H at 7.15 ppm): ca. 7.15-7.13 (superimposed by solvent, NHC: *p-H*), 7.06, 7.04 (2 s, 4H, NHC: *m-H*), 7.01 (s, 2H, NHC-side: *o-H*), 6.93 (s, 2H, TMS-side: *o-H*), 6.61 (s, 1H, TMS-side: *p-H*), 6.56 (s, 1H, NHC-side: *p-H*), 6.39 (s, 2H, *H*C=C*H*), 2.73 (sept, 4H, ^3J = 6.56 Hz, iPr-C*H*), 2.16 (s, 6H, TMS-side: Ar-Me), 2.06 (s, 6H, NHC-side: Ar-Me), 1.31 (d, 12H, ^3J = 6.46 Hz, endo-iPr-CH_3), 0.92 (d, 12H, ^3J = 6.74 Hz, exo-iPr-CH_3), 0.53 (s, 3H, B-CH_3), 0.36 (s, 18H, TMS).

^{13}C{^{1}H} (100.64 MHz, 298 K, C_6D_6 solvent signal at 128.0 ppm): 156.7 (N-*C*-N), 149.4 (NHC-side: C_β), 146.9 (TMS-side: *ipso*-C_{ar}), 146.0 (NHC-side: *ipso*-C_{ar}), 145.5 (NHC: *o*-C_{ar}), 135.4 (TMS-side: *o*-C_{ar}), 134.6 (NHC-side: *o*-C_{ar}), 134.4 (TMS-side: C_β), 134.0 (NHC: *ipso*-C_{ar}), 131.7 (NHC: *p*-C_{ar}), 129.9 (TMS-side: *o*-C_{ar}), 128.9 (NHC-side: *o*-C_{ar}), 126.2 (NHC-side: *p*-C_{ar}), 126.2 (H*C*=*C*H), 125.4 (TMS-side: *p*-C_{ar}), 125.0 (NHC: *m*-C_{ar}), 92.4 (NHC-side: C_α), 50.5 (TMS-side: C_α), 29.4 (iPr-*C*H), 26.5 (exo-iPr-*C*H_3), 22.6 (endo-iPr-*C*H_3), 21.5 (TMS-side: Ar-Me), 21.5 (NHC-side: Ar-Me), 8.8 (B-*C*H_3), 3.6 (TMS).

^{11}B (128.37 MHz, 298 K, C_6D_6): 61.8 ($\omega_{1/2}$ = 1775 Hz).

^{29}Si (99.38 MHz, 298 K, C_6D_6): -4.5 (TMS), -26.2 ($SiCl_2$).

Elemental Analysis: ($C_{54}H_{75}BCl_2N_2Si_3$) calcd C 70.64, H 8.23, B 1.18, Cl 7.72, N 3.05, Si 9.18, observed C 69.63, H 8.38, N 2.98.

LIFDI-MS: calcd exact mass: 917.48 m/z, observed m/z: 391.0 $[NHC-H]^+$, 917.3 $[M]^+$.

UV-*vis*: λ_{max} = 548 nm (toluene, $\varepsilon_{548} \approx 570$ L mol^{-1}cm^{-1}).

Crystal structure of Compound 2a-Me

For further details on the diffraction measurement please see the respective section.

2a-Me crystallised from solutions in toluene in a freezer (-40°C).

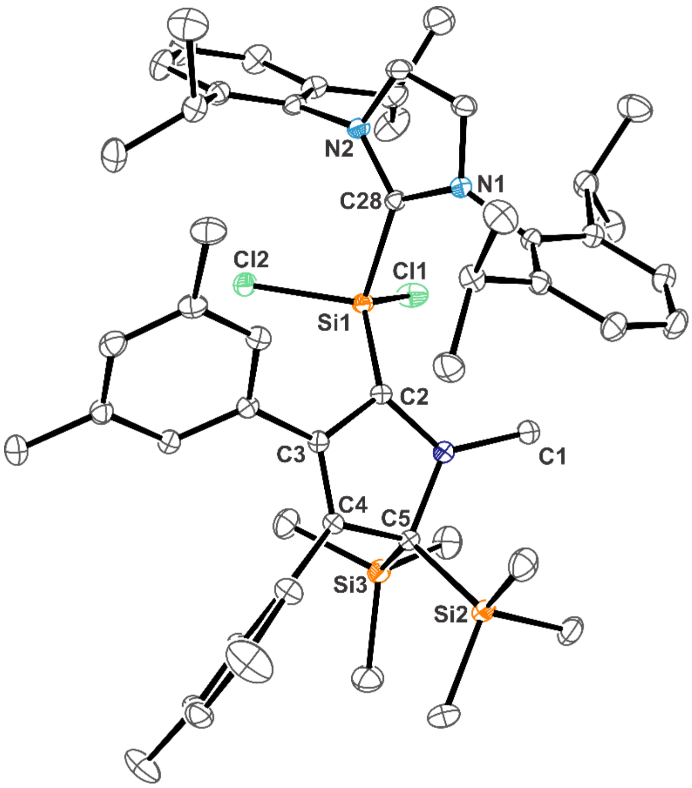

ORTEP plot of the molecular structure of **1a-*p*Xyl**. Atomic displacement parameters are drawn at 50% probability level. A second molecule in the asymmetric unit and hydrogen atoms are omitted for the sake of clarity. Selected bond length in Å: B1-C1 1.588(1), B1-C2 1.513(1), C2-C3 1.477(1), C3-C4 1.371(1), C4-C5 1.530(1), C5-B1 1.610(1), C2-Si1 1.7590(9), Si1-C28 1.947(1). The structure was deposited with the CCSD.

Spectra Plots for Compound 2a-Me

1H-NMR-spectrum of compound **2a-Me** in C6D6
C6D5H at 7.15 ppm

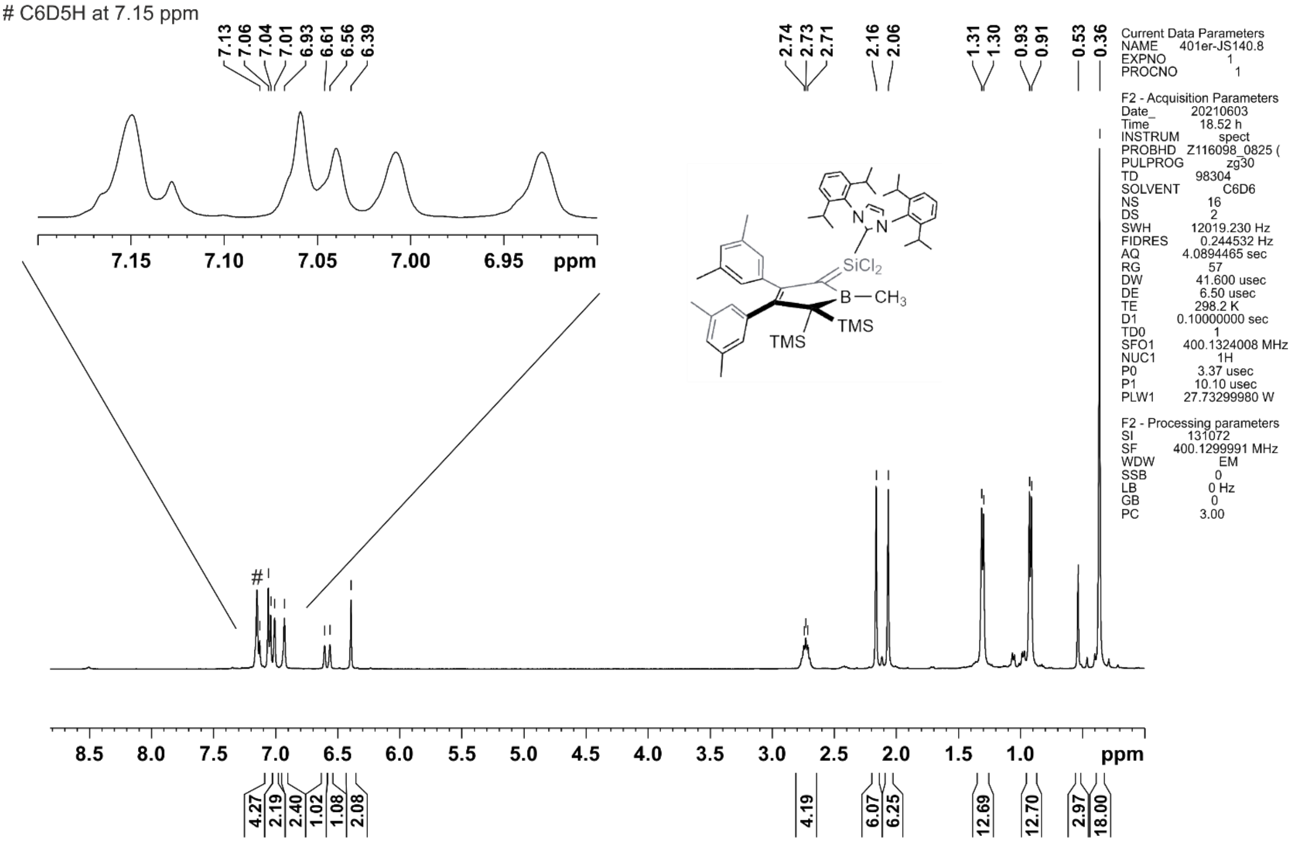

13C{1H}-NMR-spectrum of compound **2a-Me** in C6D6
C6D6 at 128.0 ppm

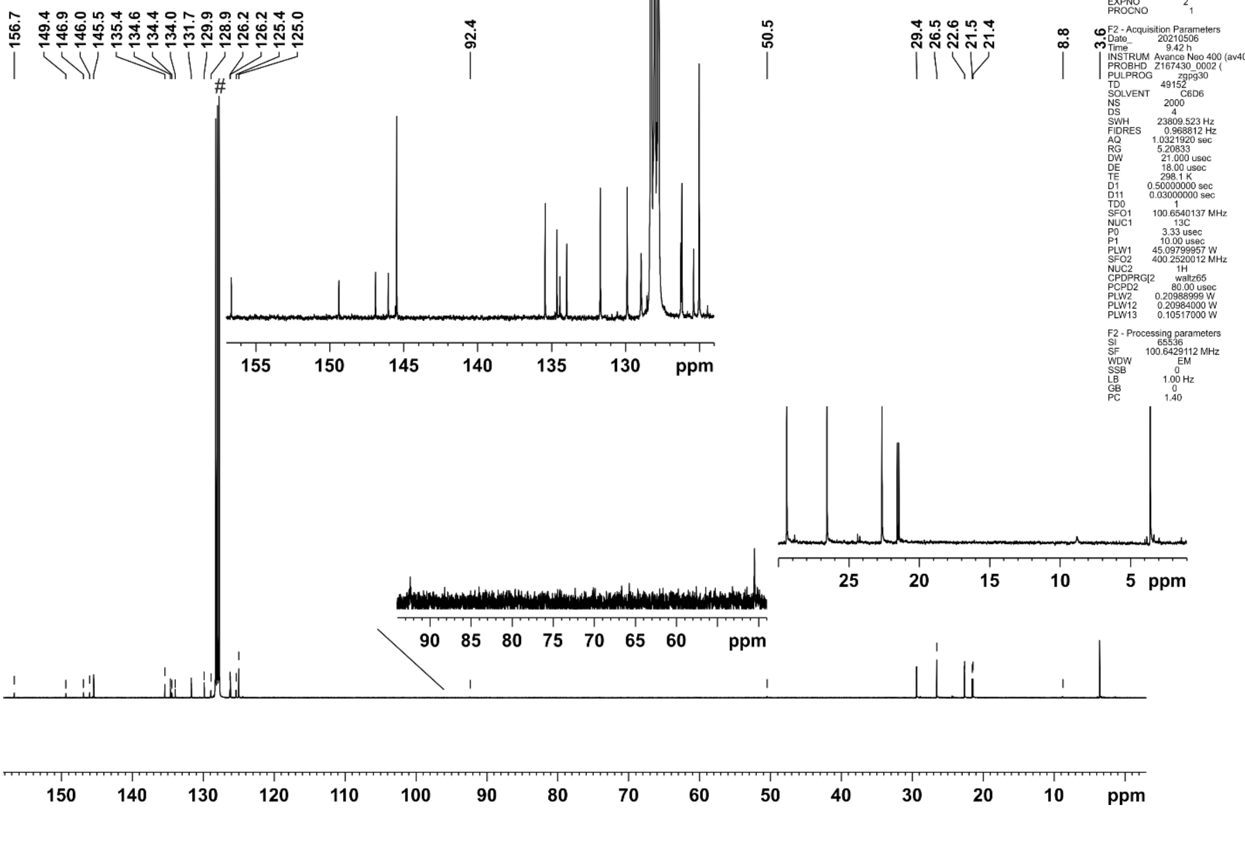

11B-NMR spectrum (background suppressed) of compound **2a-Me** in C6D6

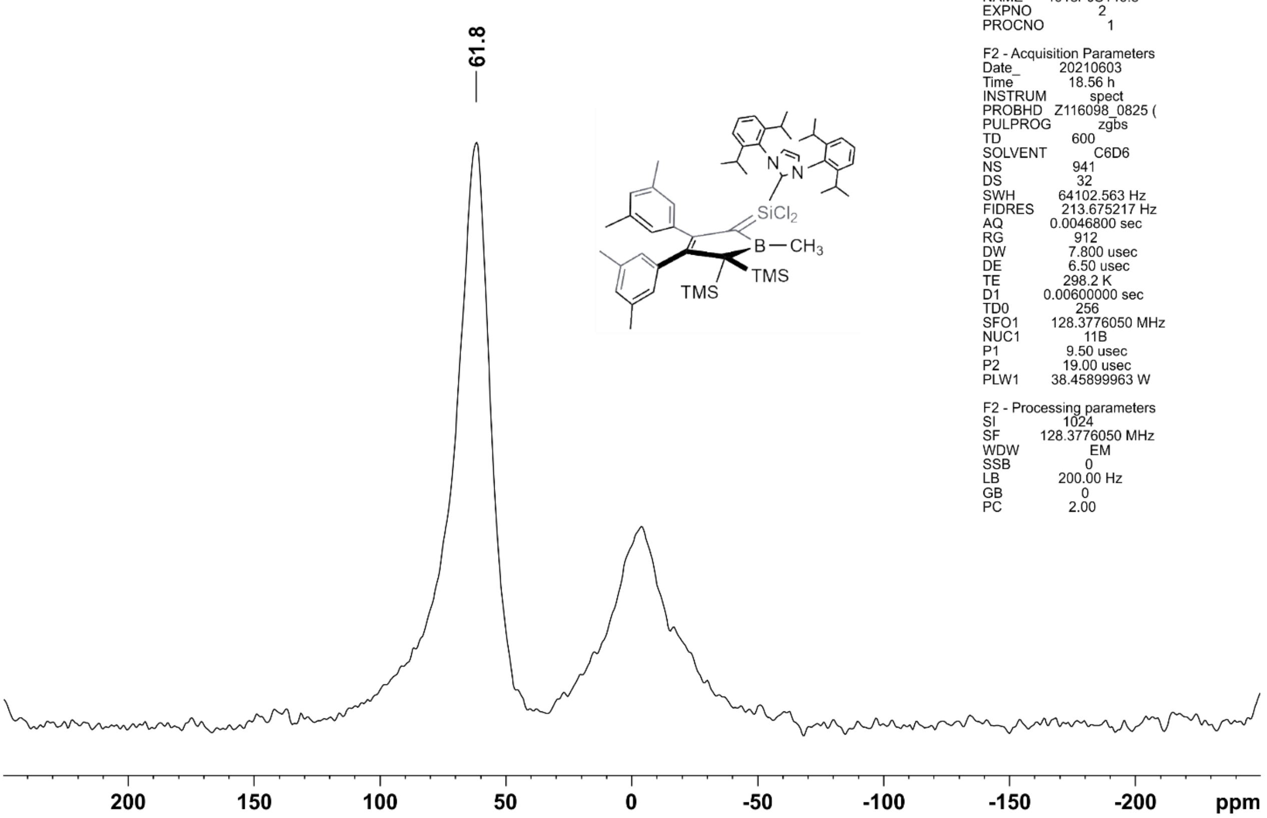

29Si-NMR spectrum of compound **2a-Me** in C6D6

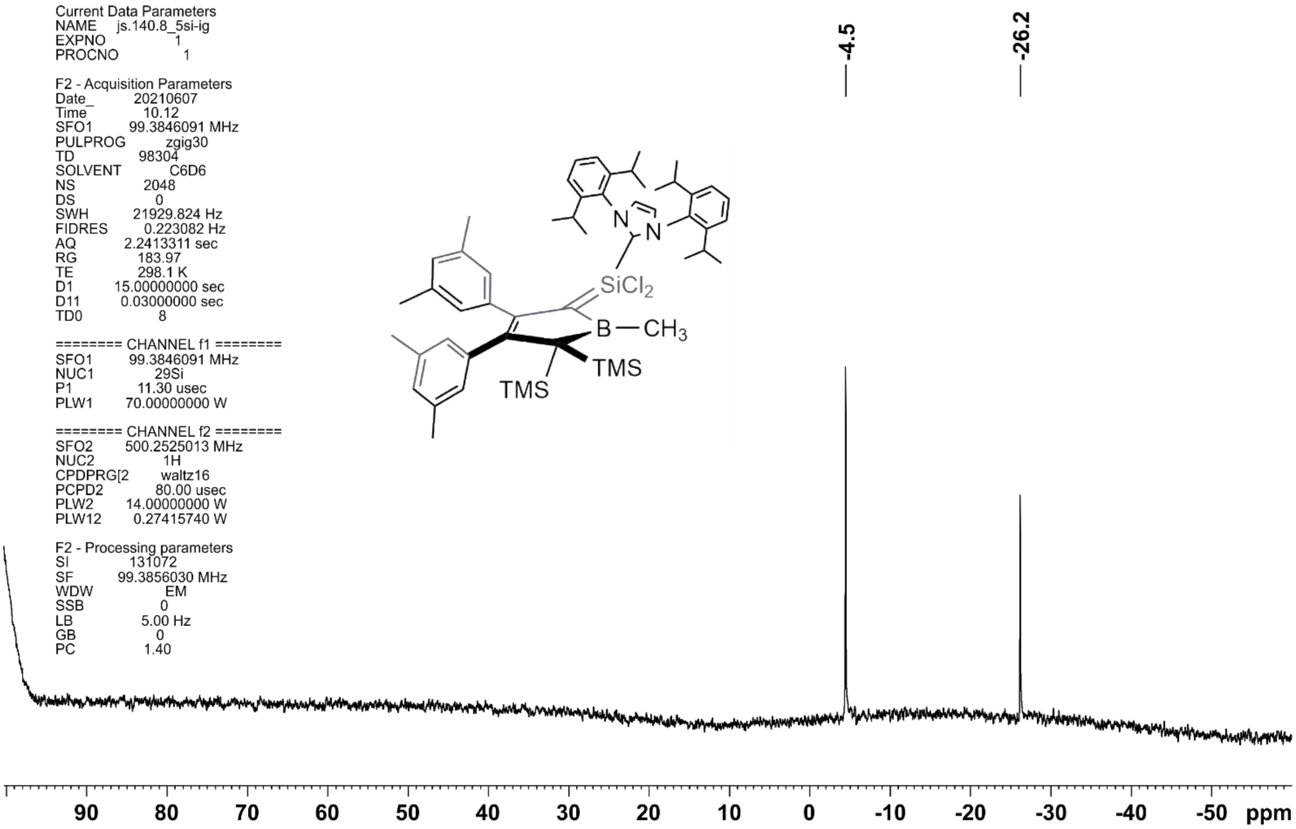

Acq. Data Name: jsarcev00011-1
Creation Parameters: Average(MS[1] Time:0.92..0.92)
External Sample Id: JS 140

LIFDI-MS

Experiment Date/Time: 5/26/2021 11:29:08 AM
Ionization Mode: FD+

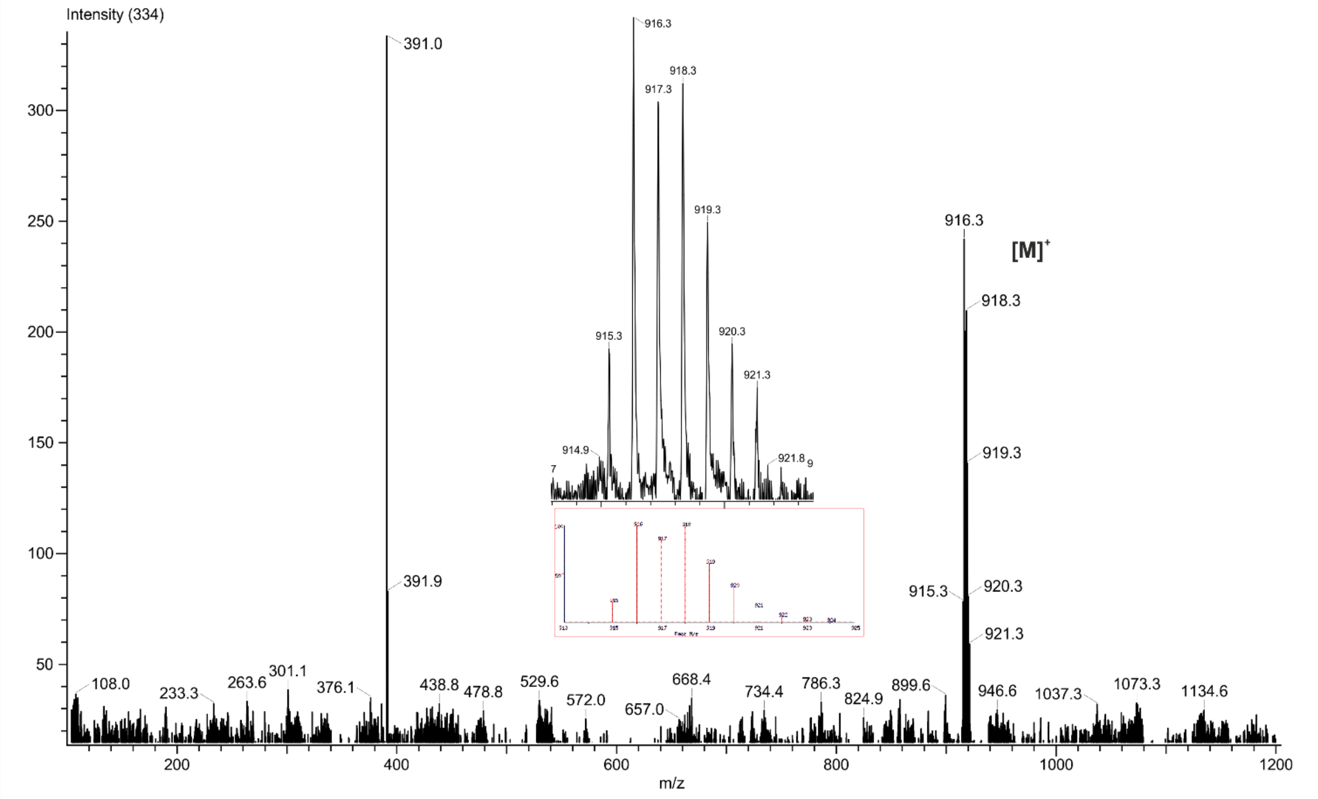

Compound 2a-Cl

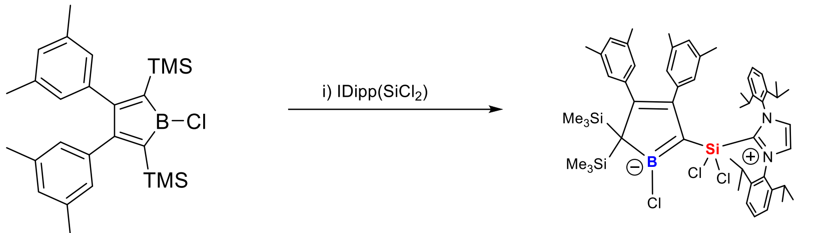

A-Cl Molecular Weight: 451,00

2a-Cl Molecular Weight: 938,59

In a glovebox, a mixture of chloroborole **A-Cl** (189.6 mg, 0.4205 mmol, 1 eq.) and dichlorosilylene IDipp($SiCl_2$) (205.0 mg, 0.4205 mmol, 1 eq.) was solved in benzene (5 mL, SPS grade). The instant dark red solution was stirred at room temperature for 1.5 h after which the benzene was removed under reduced pressure. The dark red solid was then washed with a mixture of toluene and hexane (2 × 3 mL; v/v 1:2). After drying in vacuo, compound **2a-Cl** was obtained as a crystalline red solid (208.9 mg, 0.222 mmol, 53 %).

Analytical Data for Compound 2a-Cl

NMR:

^{1}H (300.13 MHz, 298 K, C_6D_6, C_6D_5H at 7.15 ppm): 7.20 (m, 2H, NHC: *p-H*), 7.09 (s, 2H, NHC: *m-H*), 7.07 (s, 2H, NHC: *m-H*), 6.97 (s, 2H, TMS-side: *o-H*), 6.79 (s, 2H, NHC-side: *o-H*), 6.56 (s, 1H, NHC-side: *p-H*), 6.54 (s, 1H, TMS-side: *p-H*), 6.36 (s, 2H, *H*C=C*H*), 2.75 (sept, 4H, ^{3}J = 6.65 Hz, iPr-C*H*), 2.11 (s, 6H, TMS-side: Ar-Me), 2.08 (s, 6H, NHC-side: Ar-Me), 1.34 (d, 12H, ^{3}J = 6.58 Hz, endo-iPr-CH_3), 0.89 (d, 12H, ^{3}J = 6.80 Hz, exo-iPr-CH_3), 0.44 (s, 18H, TMS)

^{13}C{^{1}H} (100.64 MHz, 298 K, C_6D_6 solvent signal at 128.0 ppm): 154.0 (N-*C*-N), 148.9 (NHC-side: C_β), 145.4 (NHC: *o*-C_{ar}), 145.2 (TMS-side: *ipso*-C_{ar}), 144.9 (NHC-side: *ipso*-C_{ar}), 135.3 (TMS-side: *m*-C_{ar}), 134.7 (NHC-side: *m*-C_{ar}), 133.9 (NHC: *ipso*-C_{ar}), 131.7 (NHC: (*p*-C_{ar}), 131.6 (TMS-side: C_β), 130.5 (TMS-side: *o*-C_{ar}), 129.2 (NHC-side: *o*-C_{ar}), 126.5 (NHC-side: *p*-C_{ar}), 126.5 (H*C*=*C*H), 126.0 (TMS-side: *p*-C_{ar}), 124.9 (NHC: *m*-C_{ar}), 91.2 (NHC-side: C_α), 48.0 (TMS-side: C_α), 29.3 (iPr-*C*H), 26.4 (endo-iPr-*C*H_3), 22.7 (exo-iPr-*C*H_3), 21.5 (NHC-side: Ar-Me), 21.4 (TMS-side: Ar-Me), 3.1 (TMS).

^{11}B (128.37 MHz, 298 K, C_6D_6): 53.8 ($\omega_{1/2}$ = 840 Hz).

^{29}Si (99.38 MHz, 298 K, C_6D_6): -3.4 (TMS), -24.8 ($SiCl_2$).

UV-*vis*: λ_{max} = 500 nm (toluene, $\varepsilon_{500} \approx$ 100 L mol^{-1}cm^{-1}).

Elemental Analysis: ($C_{53}H_{72}BCl_3N_2Si_3$) calcd C 67.82, H 7.73, B 1.15, Cl 11.33, N 2.98, Si 8.98, observed C 67.33, H 7.89, N 2.87.

Crystal structure of Compound 2a-Cl

For further details on the diffraction measurement please see the respective section.

2a-Cl crystallised from solutions in toluene in a freezer (-35°C).

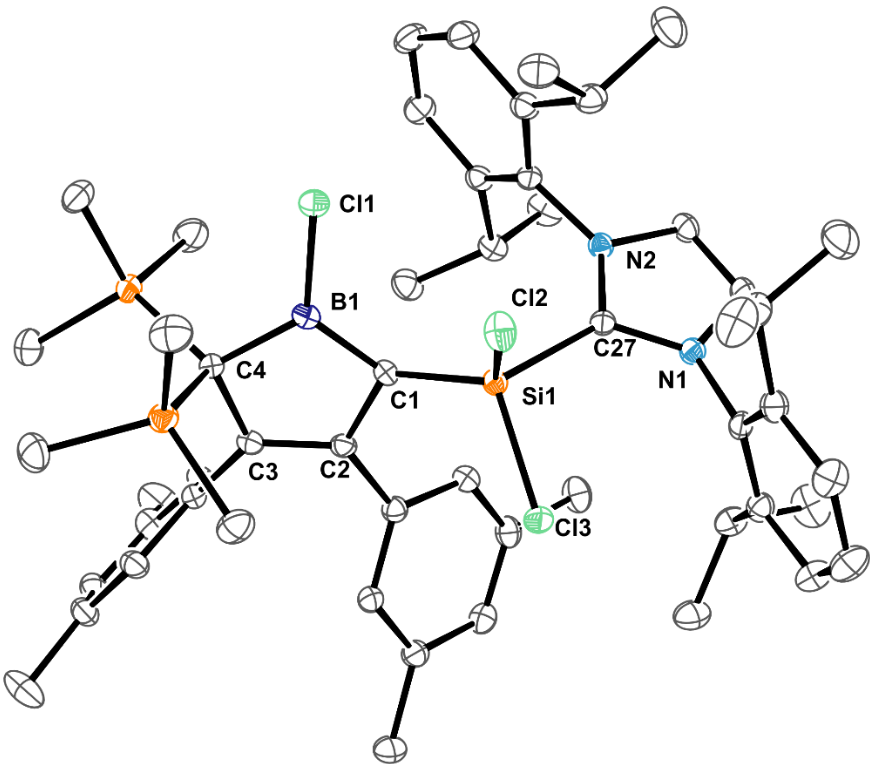

ORTEP plot of the molecular structure of **2a-Cl**. Atomic displacement parameters are drawn at 50% probability level. Hydrogen atoms are omitted for the sake of clarity. Selected bond length in Å: B1-C1 1.491(2), C1-C2 1.483(2), C2-C3 1.369(2), C3-C4 1.536(2), C4-B1 1.591(2), B1-Cl1 1.809(1), C1-Si1 1.765(1), Si1-C27 1.939(1), Si1-Cl2 2.0673(6), Si1-Cl3 2.0667(5). The structure was deposited with the CCSD.

Spectra Plots for Compound 2a-Cl

1H-NMR-spectrum of compound **2a-Cl** in C6D6
C6D5H at 7.15 ppm

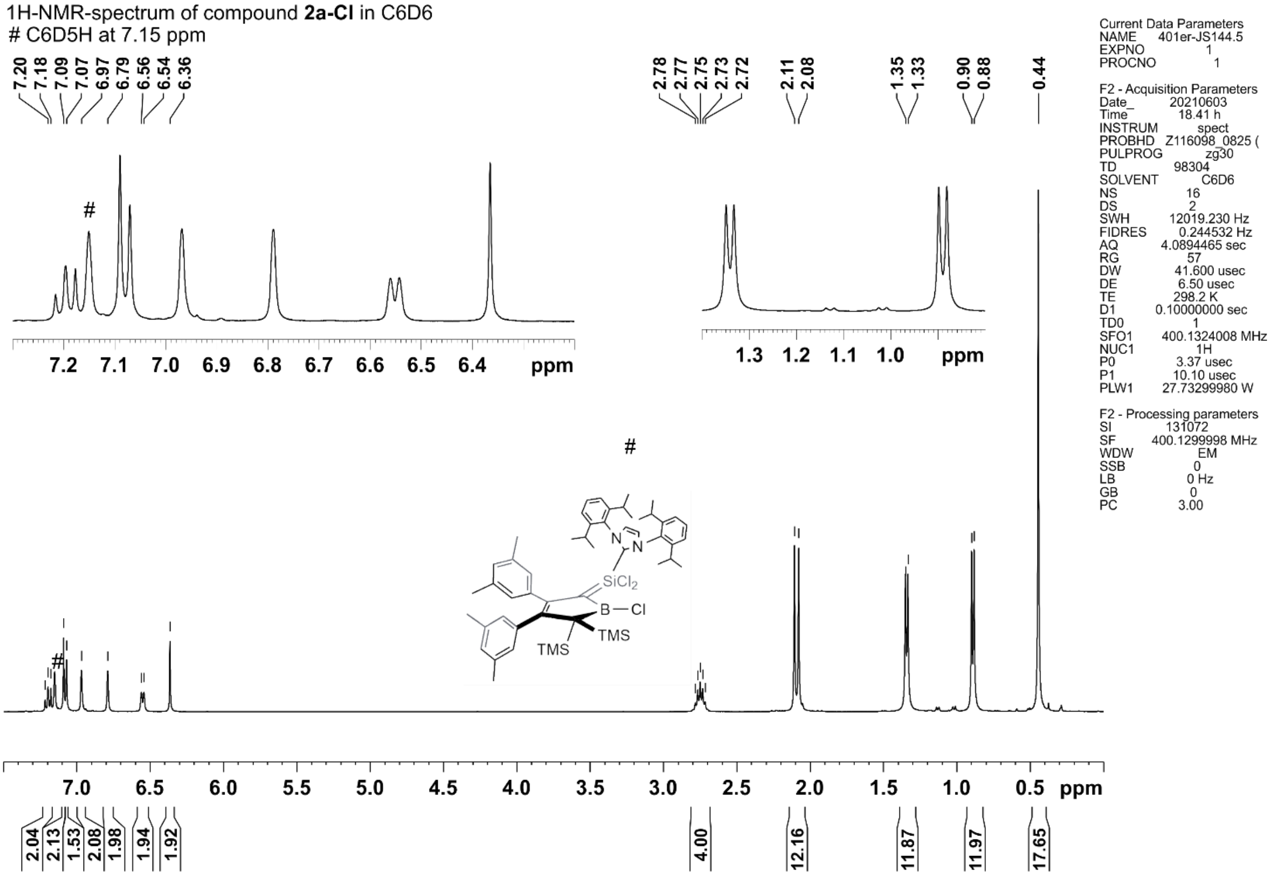

13C{1H}-NMR-spectrum of compound **2a-Cl** in C6D6
C6D6 at 128.0 ppm

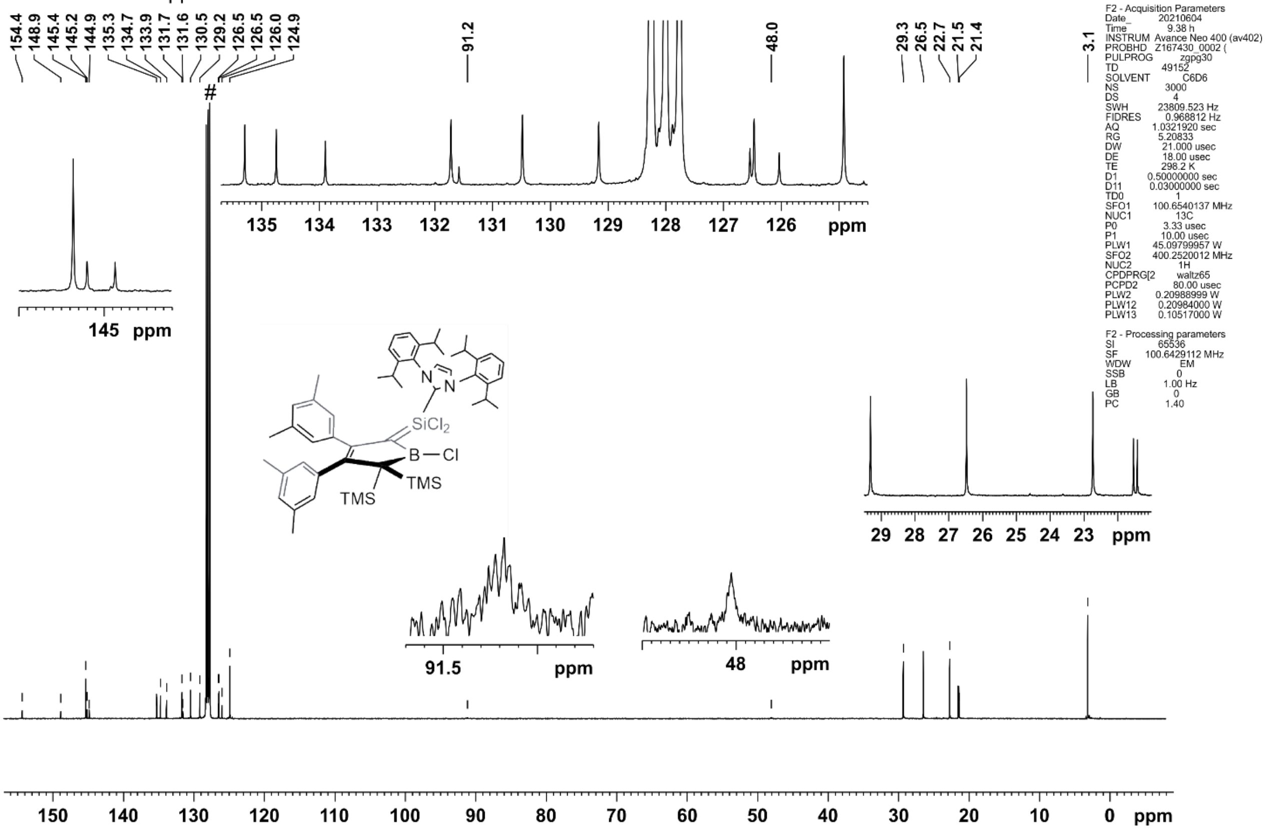

11B-NMR spectrum (background suppressed) of compound **2a-Cl** in C6D6

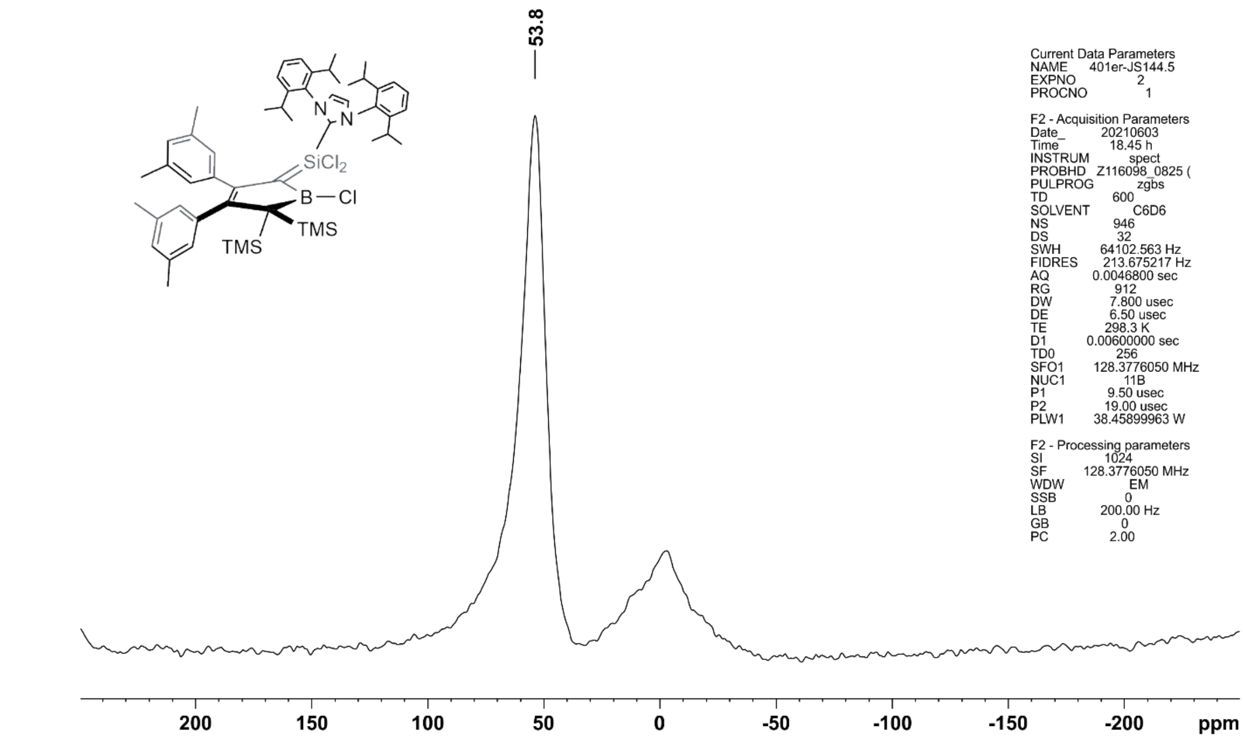

29Si-NMR spectrum of compound **2a-Cl** in C6D6

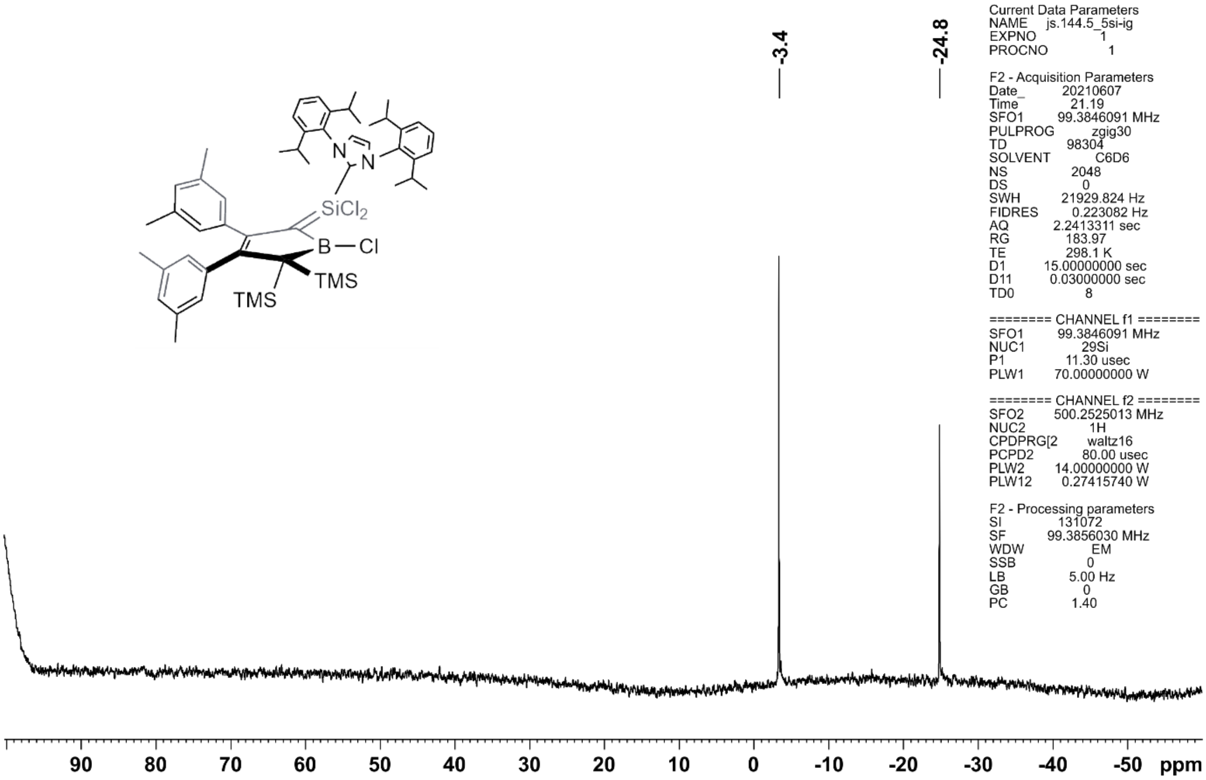

Compound 2a-Xyl

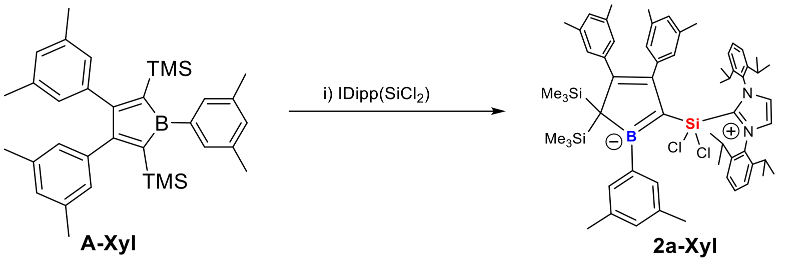

A-Xyl Molecular Weight: 520,71

2a-Xyl Molecular Weight: 1008,30

In a glovebox, to a mixture of borole **A-Xyl** (77.0 mg, 0.159 mmol, 1 eq.) and IDipp-$SiCl_2$ (72.0 mg, 0.149 mmol, 1 eq.) toluene (3 mL) was added. The solution immediately turned dark red and was stirred for 20 min at room temperature. The toluene was removed under reduced pressure to give a dark red, crystalline solid which was then washed with portions of pentane (2 mL, 2 mL, 1 mL). After drying in vacuo, the silene adduct **2a-Xyl** was obtained as a dark red solid (102.5 mg, 0.1016 mmol, 68 %).

Analytical Data for Compound 2a-Xyl

NMR:

^{1}H (400.13 MHz, 348 K, C_6D_6, C_6D_5H at 7.15 ppm): 7.28 (t, 2H, ^3J = 7.64 Hz, NHC: *p-H*), 7.09 (d, 4H, ^3J = 7.64 Hz, NHC: *m-H*), 7.00 (m, 1H, B-Aryl: *p*-H), 6.96 (s, 2H, NHC-side: *o-H*), 6.93 (s, 2H, TMS-side: *o-H*), 6.88 (m, 2H, B-Aryl: *o*-H), 6.78 (s, 1H, NHC-side: *p-H*), 6.57 (s, 1H, TMS-side: *p-H*), 6.44 (m, 2H, *H*C=C*H*), 2.60 (m, 4H, iPr-C*H*), 2.36 (s, 6H, B-Aryl: *m*-C*H*$_3$), 2.16 (s, 6H, TMS-side: Ar-Me), 2.03 (s, 6H, NHC-side: Ar-Me), 1.10 (d, 12H, ^3J = 5.34 Hz, endo-iPr-C*H*$_3$), 0.85 (d, 12H, ^3J = 6.54 Hz, exo-iPr-C*H*$_3$), 0.22 (s, 18H, TMS).

^{13}C{^{1}H} (100.62 MHz, 348 K, C_6D_6 solvent signal at 128.0 ppm): 145.7 (NHC: *o-C$_{ar}$*), 134.5 (TMS-side: *m-C$_{ar}$*), 134.4 (B-Aryl: *o-C$_{ar}$*), 134.3 (NHC-side: *m-C$_{ar}$*), 132.4 (TMS-side: *o-C$_{ar}$*), 132.3 (B-Aryl: *m-C$_{ar}$*), 131.5 (NHC: *o-C$_{ar}$*), 129.2 (NHC-side: *o-C$_{ar}$*), 126.9 (B-Aryl: *o-C$_{ar}$*), 126.5 (H*C*=*C*H), 126.4 (NHC-side: *p-C$_{ar}$*), 126.0 (TMS-side: *p-C$_{ar}$*), 125.2 (NHC: *m-C$_{ar}$*), 29.3 (iPr-*C*H), 26.6 (exo-iPr-*C*H$_3$), 22.6 (endo-iPr-*C*H$_3$), 21.9 (B-Aryl: Ar-Me), 21.2 (TMS-side: Ar-Me), 21.1 (NHC-side: Ar-Me), 4.6 (TMS). Only in 2D spectra: ca. 150.8 (NHC-side: C_β), ca. 133 (TMS-side: C_β), ca. 53 (TMS-side: C_α). Not found: (TMS-side: *ipso-C$_{ar}$*), (NHC-side: *ipso-C$_{ar}$*), (B-Aryl: *ipso-C$_{ar}$*), (NHC-side: C_α).

^{11}B (128.43 MHz, 300 K, C_6D_6): 54.7 ($\omega_{1/2}$ = 557 Hz).

^{29}Si (79.52 MHz, 300 K, C_6D_6): -4.5 (TMS), -23.4 ($SiCl_2$).

Elemental Analysis: ($C_{61}H_{81}BCl_2N_2Si_3$) calcd C 72.66, H 8.10, B 1.07, Cl 7.03, N 2.78, Si 8.36, observed C 72.25, H 7.89, N 2.86.

UV-*vis*: λ_{max} = 514 nm (toluene, $\varepsilon_{514}\approx$190 L mol^{-1}cm^{-1}).

Crystal structure of Compound 2a-Xyl

For further details on the diffraction measurement please see the respective section.

2a-Xyl crystallised from solutions in toluene in a freezer (-35°C).

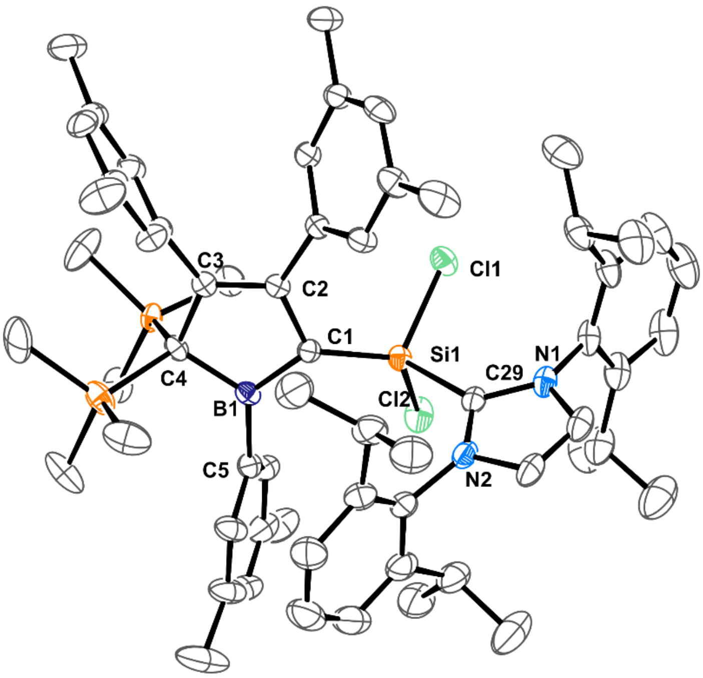

ORTEP plot of the molecular structure of **1a-*p*Xyl**. Atomic displacement parameters are drawn at 50% probability level. A second molecule in the asymmetric unit and hydrogen atoms are omitted for the sake of clarity. Selected bond length in Å: B1-C1 1.513(3), C1-C2 1.476(3), C2-C3 1.368(3), C3-C4 1.525(3), C4-B1 1.619(4), B1-C5 1.591(3), C1-Si1 1.773(2), Si1-C29 1.957(3), Si1-Cl1 2.069(1), Si1-Cl2 2.059(1). The structure was deposited with the CCSD.

Spectra Plots for Compound 2a-Xyl

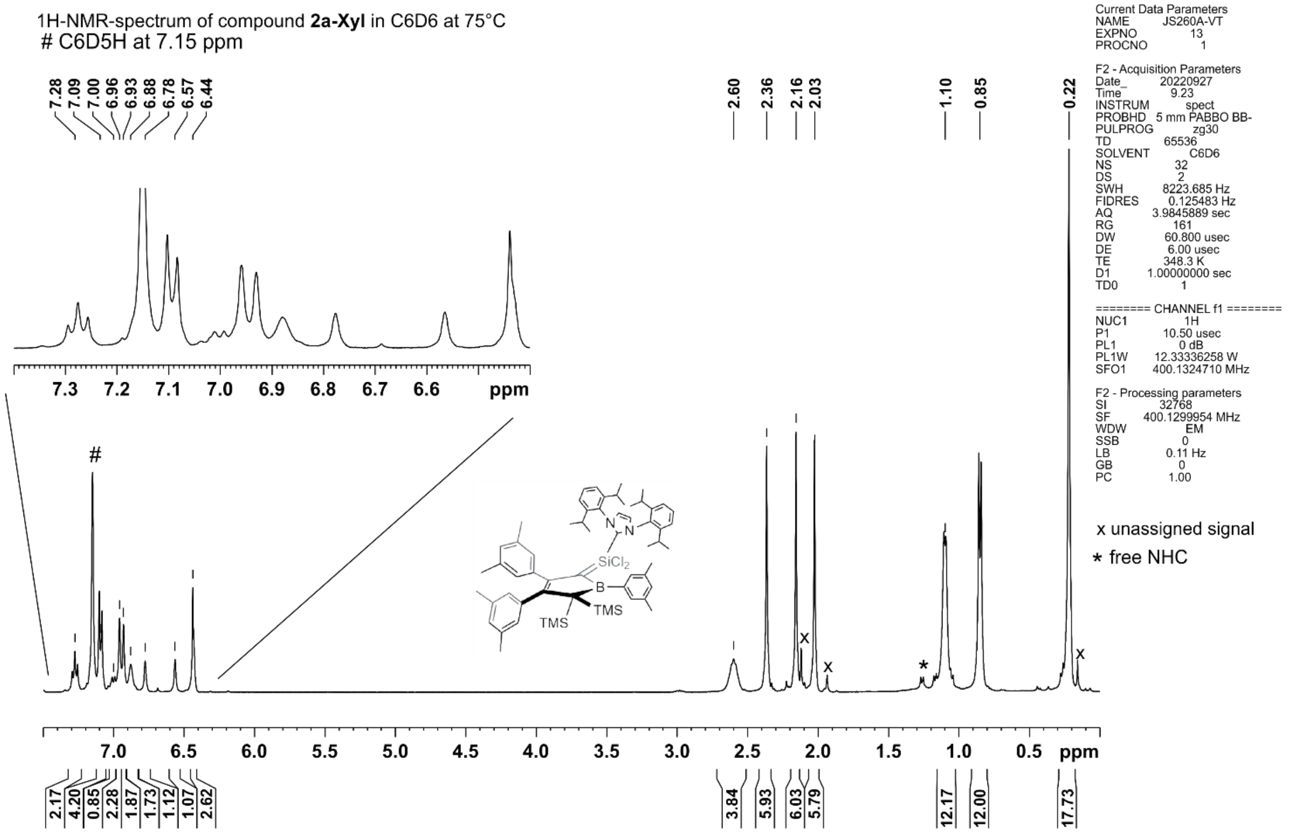

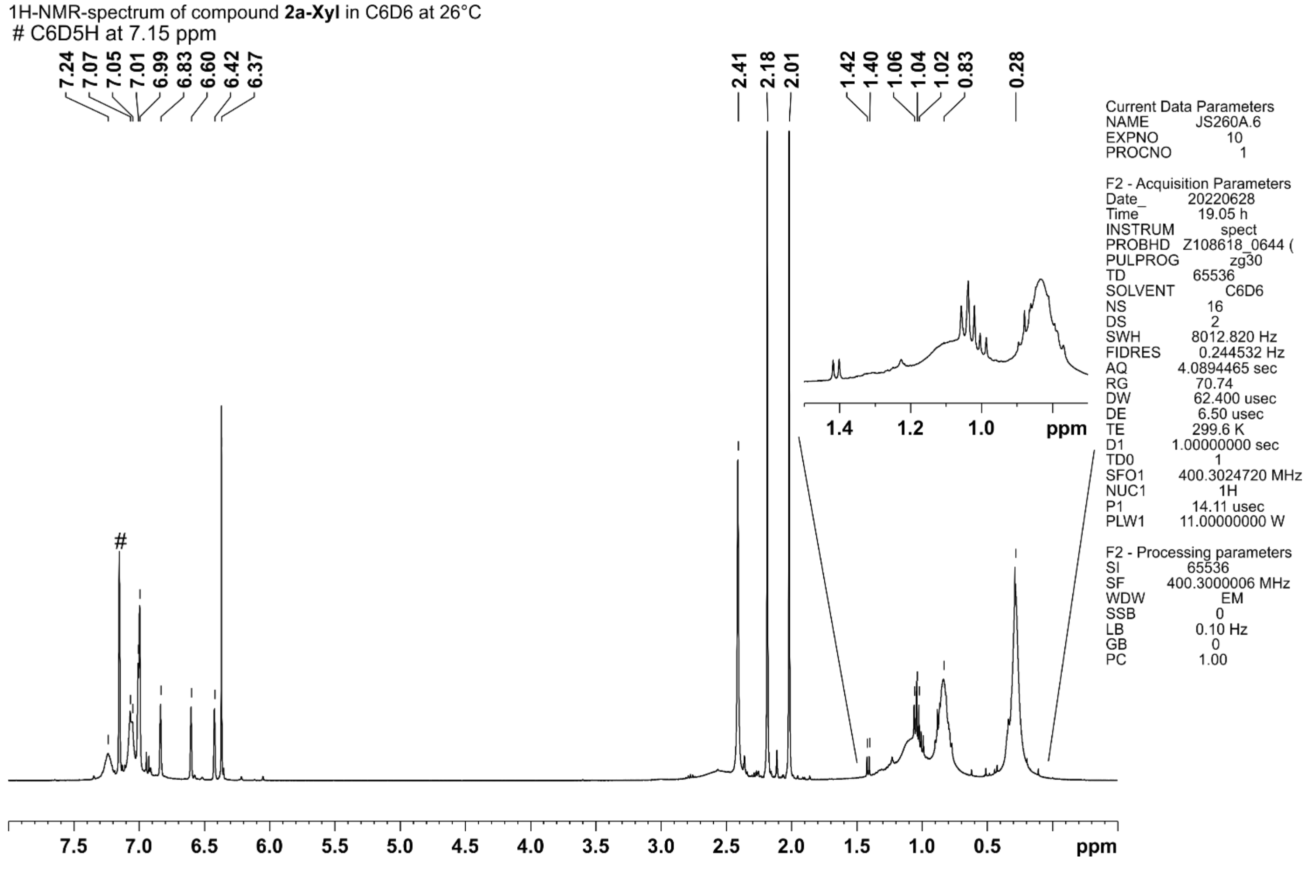

1H-NMR-spectrum of compound **2a-Xyl** in C6D6 at 75°C vs ambient temperature (26°C)

C6D5H at 7.15 ppm

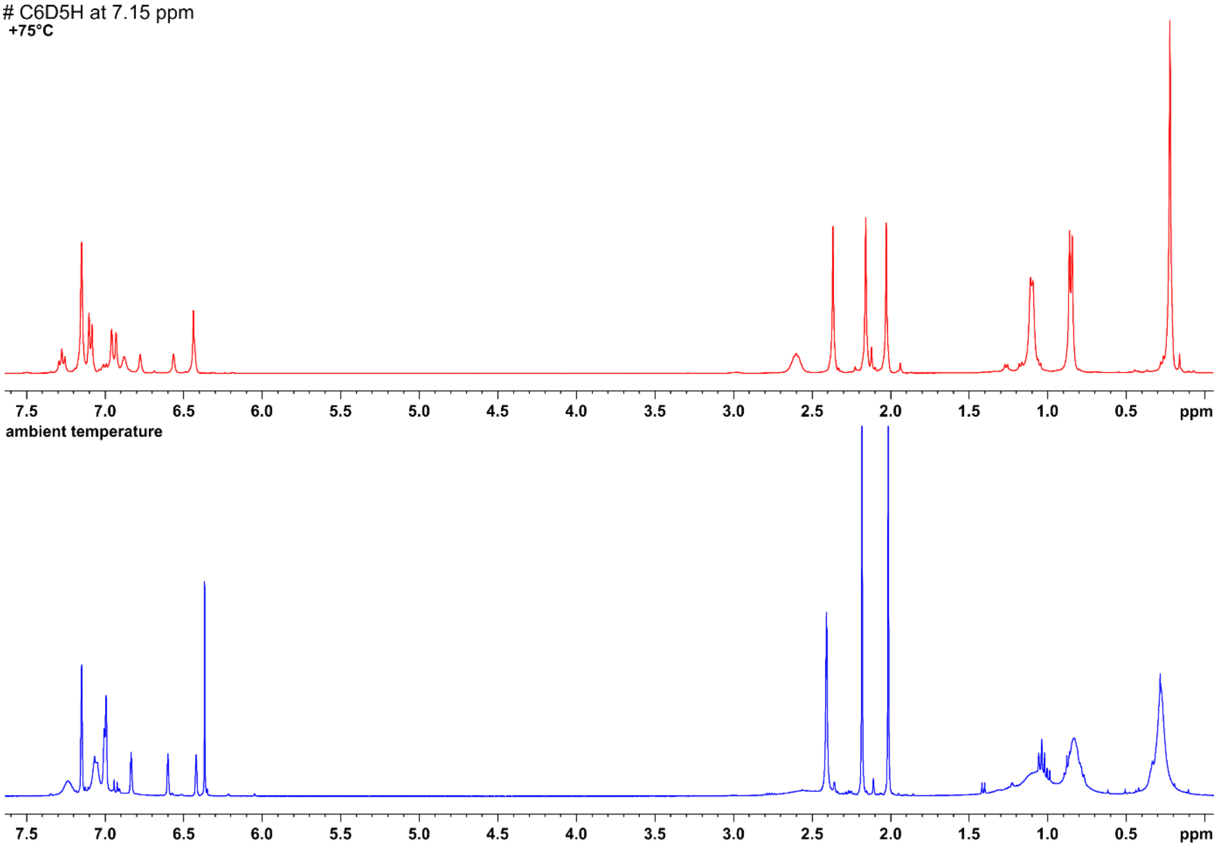

13C{1H}-NMR-spectrum of compound **2a-Xyl** in C6D6 at 75°C

C6D6 at 128.0 ppm

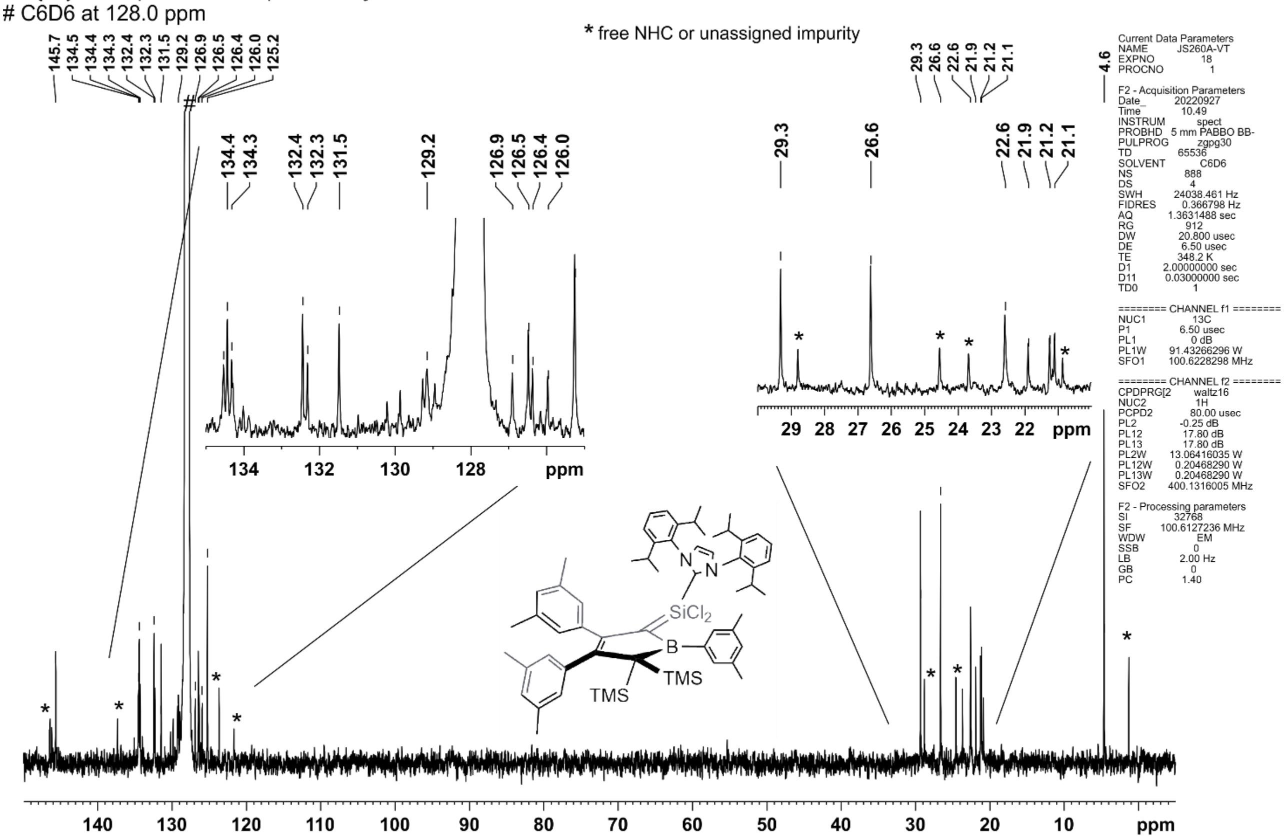

11B-NMR spectrum (background suppressed) of compound **2a-Xyl** in C6D6

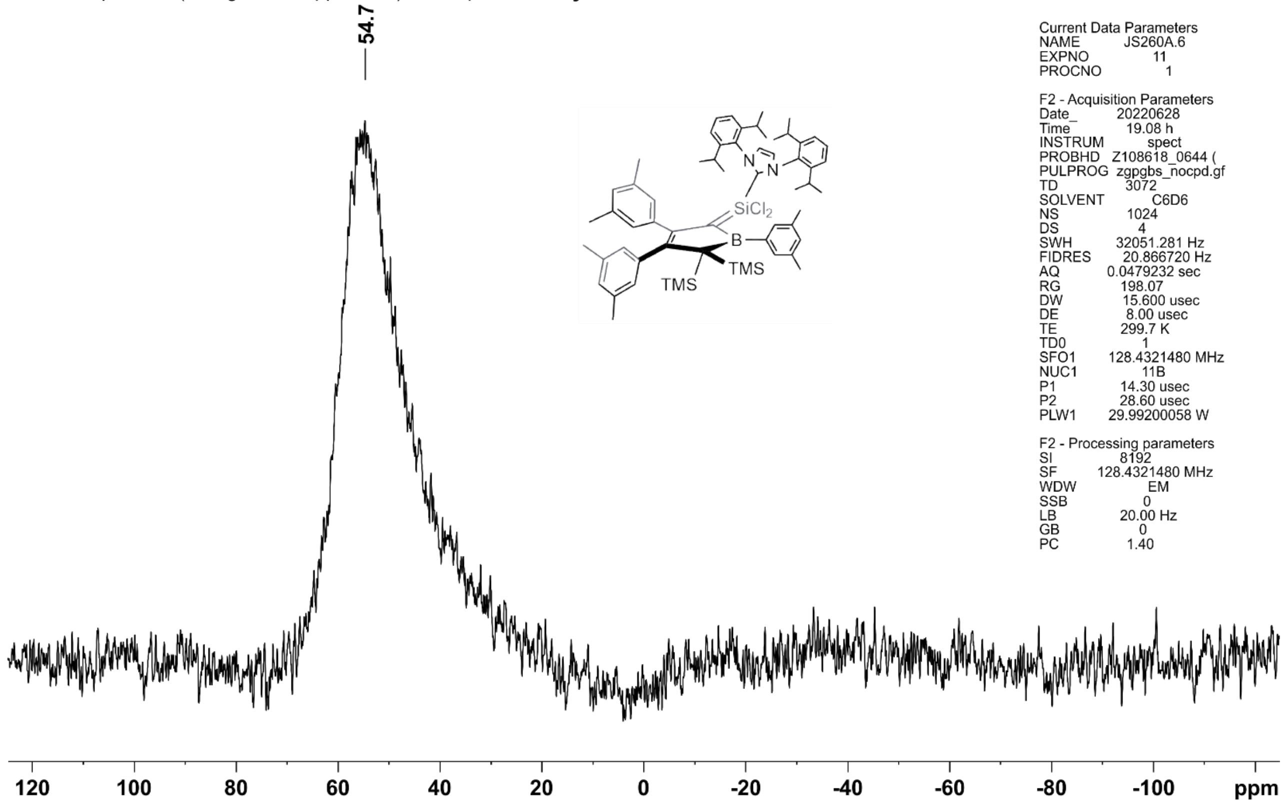

29Si-NMR spectrum of compound **2a-Xyl** in C6D6

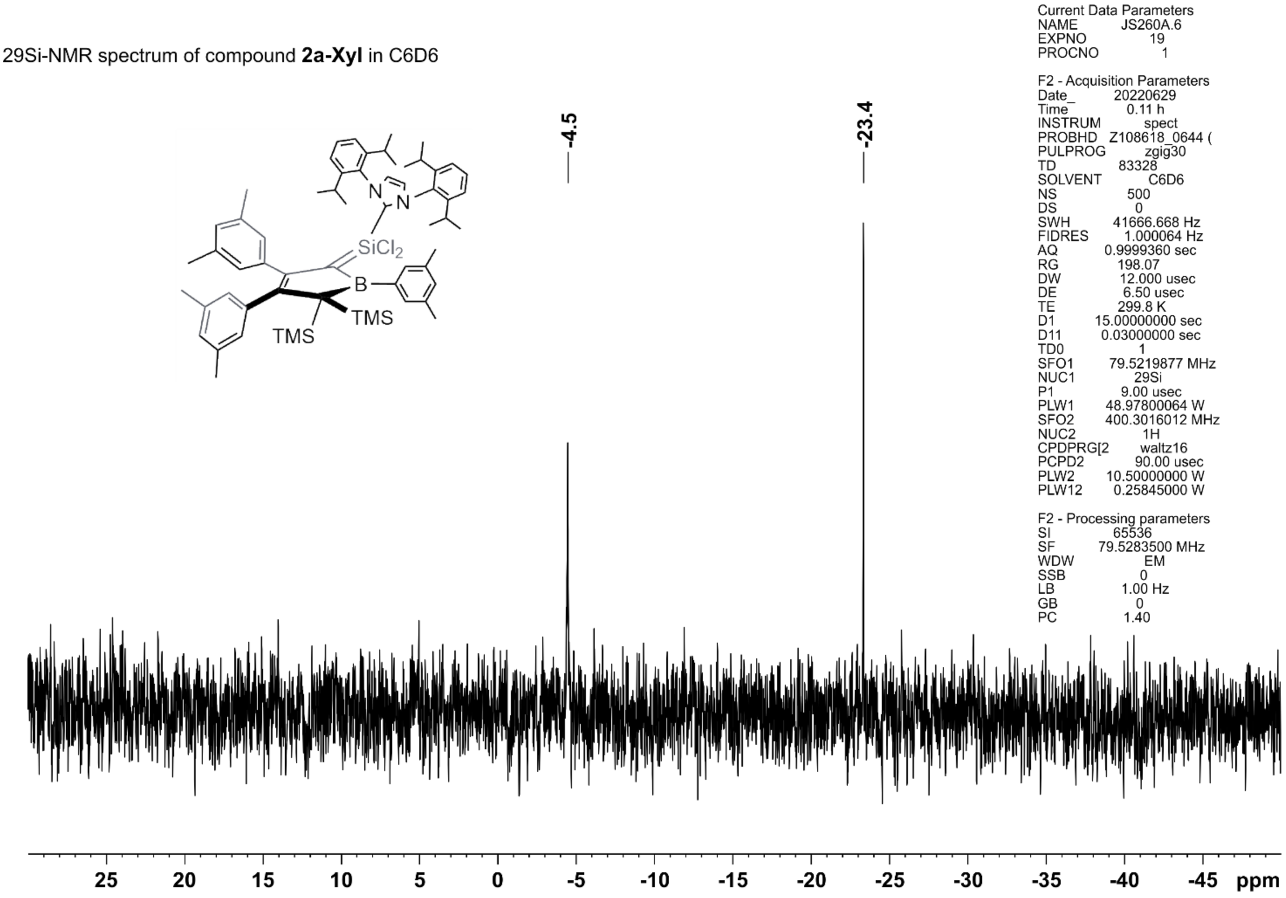

Compound 2b-Me

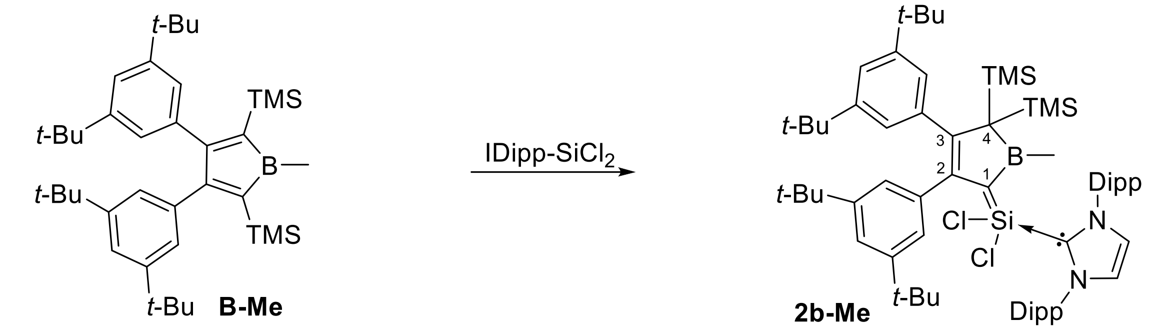

Molecular Weight: 598.9130 Molecular Weight: 1086.50

In a glovebox, methylborole **B-Me** (186.4 mg, 0.311 mmol, 1 eq) and IDipp-$SiCl_2$ (151.8 mg, 0.311 mmol, 1 eq) were dissolved in dry and degassed toluene (10 mL). The resulting deep purple solution was stirred overnight and afterwards the solvent of the reaction mixture was removed under reduced pressure. The deep purple solid was dissolved in hexane (8 mL), filtered and stored at –40 °C overnight to yield a first crop of deep purple crystals. These crystals were washed with cold hexane (–40 °C, 3 × 0.3 mL) and the volume of mother liquor was reduced to approximately 4 mL and stored at –40 °C. After two more crystallization compound **2b-Me** (274.1 mg, 0.252 mmol, 81 %) was obtained as a deep purple solid.

Analytical Data for Compound 2b-Me

NMR:

^{1}H (400.13 MHz, 298 K, C_6D_6, CD_5H at 7.15 ppm): 7.19 (d, $^4J_{HH}$ = 1.8 Hz, 1H, *o-H_{ar2}*), 7.16–7.17 (m, 2H, *p-H_{NHC}*), 7.13 (t, $^4J_{HH}$ = 1.8 Hz, 1H, *p-H_{ar3}*), 7.08 (t, $^4J_{HH}$ = 1.8 Hz, 1H, *p-H_{ar2}*), .04–7.07 (m, 6H, *o-H_{ar3}* + *m-H_{NHC}*), 6.25 (s, 2H, N-C*H_{NHC}*), 2.65 (sept, $^4J_{HH}$ = 6.8 Hz, 4H, C*H*(CH_3)$_2$), 1.35 (s, 18H, Ar_3-C(*Me*)$_3$), 1.29 (s, 18H, Ar_2-C(*Me*)$_3$), 1.29 (d, 12H, CH(C*H_3*)$_2$, partially overlapped by *t*-Bu signal), 0.91 (d, $^4J_{HH}$ = 6.8 Hz, 12H, CH(C*H_3*)$_2$), 0.38 (s, 3H, B-C*H_3*), 0.35 (s, 18H, Si(*Me*)$_3$).

^{13}C{^{1}H} (100.65 MHz, 298 K, C_6D_6, solvent signal at 128.0 ppm): 156.7 (*C_{NHC}*), 150.2 (C_2), 148.3 (*m-C_{Ar3}*), 147.5 (*m-C_{Ar2}*), 146.2 (*ipso-$C_{ar2/3}$*), 146.1 (*ipso-$C_{ar2/3}$*), 145.8 (*o-C_{NHC}*), 136.0 (C_3), 133.8 (*ipso-C_{NHC}*), 131.9 (*p-C_{NHC}*), 126.3 (*o-C_{ar3}*), 126.1 (*o-C_{ar2}*), 126.0 (N-*C*H_{NHC}), 125.0 (*m-C_{NHC}*), 118.1 (*p-C_{ar2}*), 117.4 (*p-C_{ar3}*), 93.1 (C_1), 50.1 (C_4), 34.84 (Ar_3-*C*(CH_3)$_3$), 34.76 (Ar_2-*C*(CH_3)$_3$), 32.05 $Ar_{2/3}$-(C(*C*H_3)$_3$), 32.03 ($Ar_{2/3}$-(C(*C*H_3)$_3$), 29.4 (*C*H(CH_3)$_2$), 26.6 (CH(*C*H_3)$_2$), 22.7 (CH(*C*H_3)$_2$), 8.5 (B-*C*H_3), 3.6 (Si(*C*H_3)$_3$).

^{11}B (128.38 MHz, 298 K, C_6D_6): 62.9 ($\nu_{1/2}$ ≈ 1600 Hz).

^{29}Si (inverse gated, 99.37 MHz, 298 K, C_6D_6): –4.5 (TMS), –28.0 ($SiCl_2$).

Elemental Analysis: $C_{66}H_{99}BCl_2N_2Si_3$ calcd C 72.96, H 9.18, N 2.58; observed C 73.06, H 9.19, N 2.56.

UV/VIS (*n*-hexane): λ_{max} = 560 nm (ε_{560} ≈ 920 L $mol^{-1}cm^{-1}$) .

Crystal structure of Compound 2b-Me

2b-Me crystallised reliably from solutions in pentane or hexane in a freezer (-40°C) and gave homogeneous crops of thin plate-shaped, purple crystals. The crystals were extremely sensitive to ambient atmosphere and immediately lost their purple colour, even when suspended in perfluorinated oil. Handling under a microscope was only possible applying a XTEMP setup supplying a stream of cold nitrogen gas over the sample. However, the crystals obtained (also from all other solvents tested) were consistently of insufficient quality to obtain data sets that allowed more than mere identification of the bonding pattern.

Cell found: monoclinic, $P2_1/c$; a = 23.272(3), b = 15.615(2), c = 24.488(3) Å; α =90°, β = 116.939(3)°, γ = 90°; V = 7933(2) Å^3

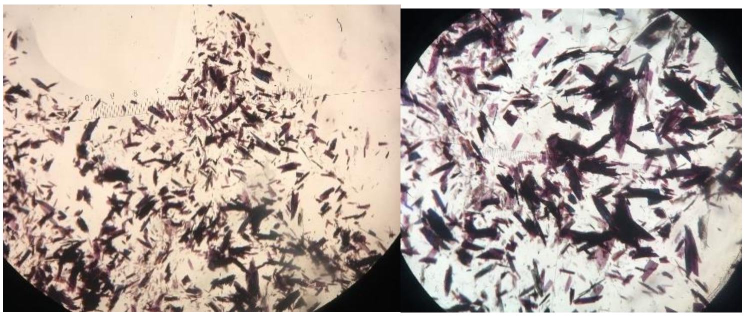

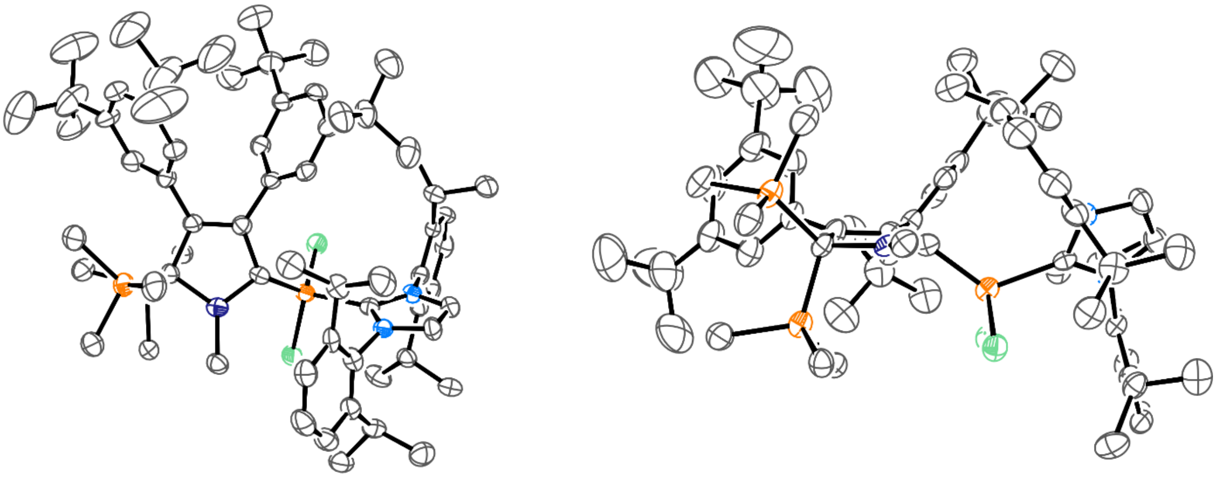

ORTEP plot of two perspectives on the molecular structure of **2b-Me**. Atomic displacement parameters are drawn at 50% probability level. Hydrogen atoms and two (disordered) molecules of pentane are omitted for the sake of clarity. Due to its poor data quality structure was not deposited with the CCSD.

Spectra Plots for Compound 2b-Me

1H-NMR-spectrum of compound **2b-Me** in C6D6
C6D5H at 7.15 ppm

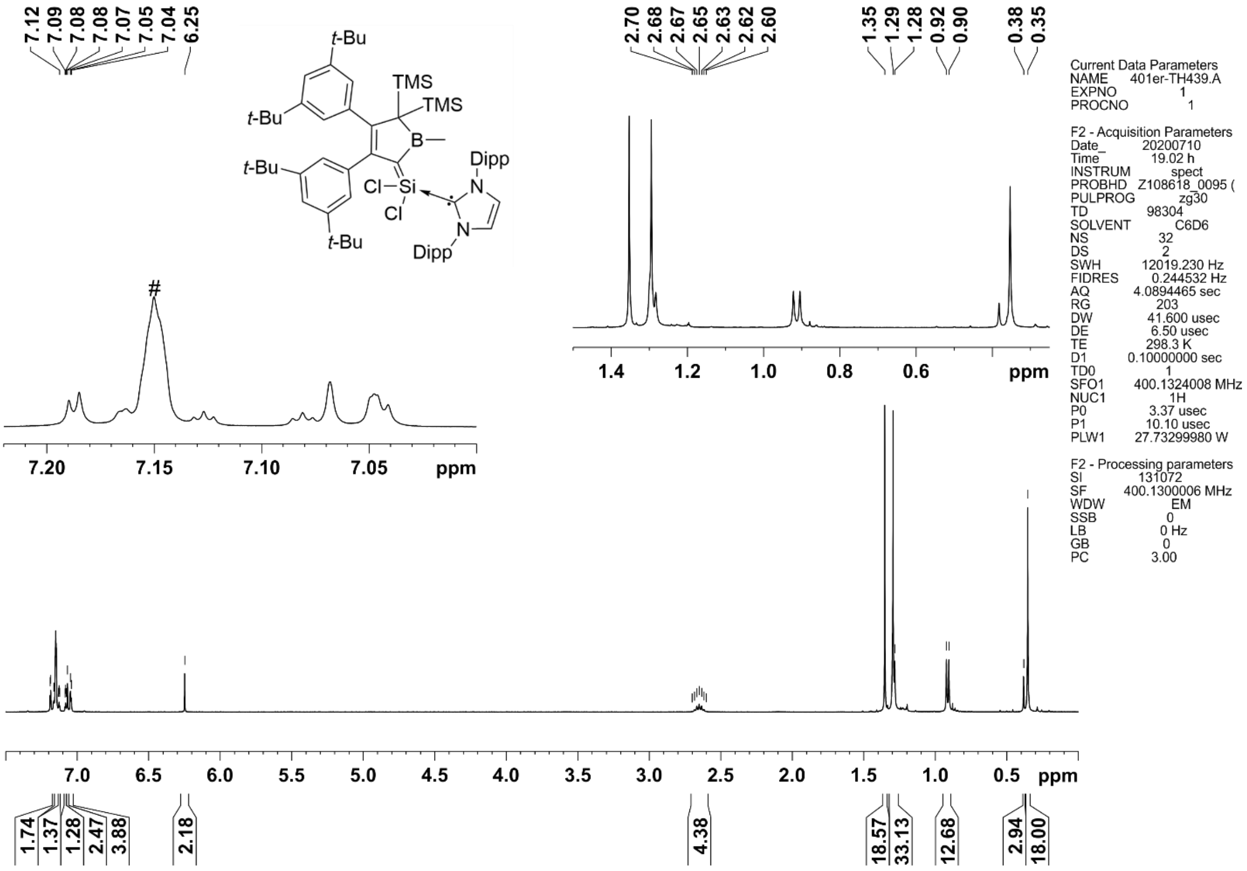

13C{1H}-NMR-spectrum of compound **2b-Me** in C6D6
C6D6 at 128.0 ppm

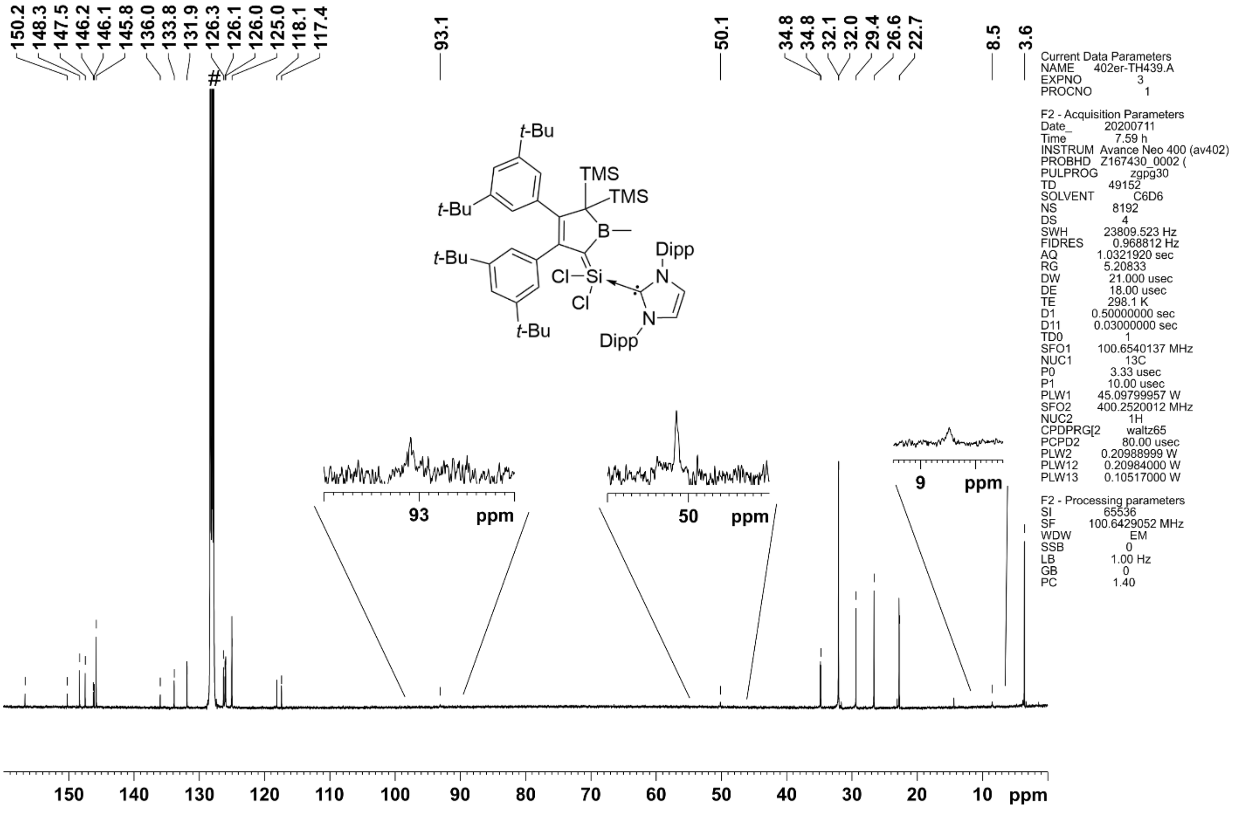

11B-NMR spectrum (background suppressed) of compound **2b-Me** in C6D6

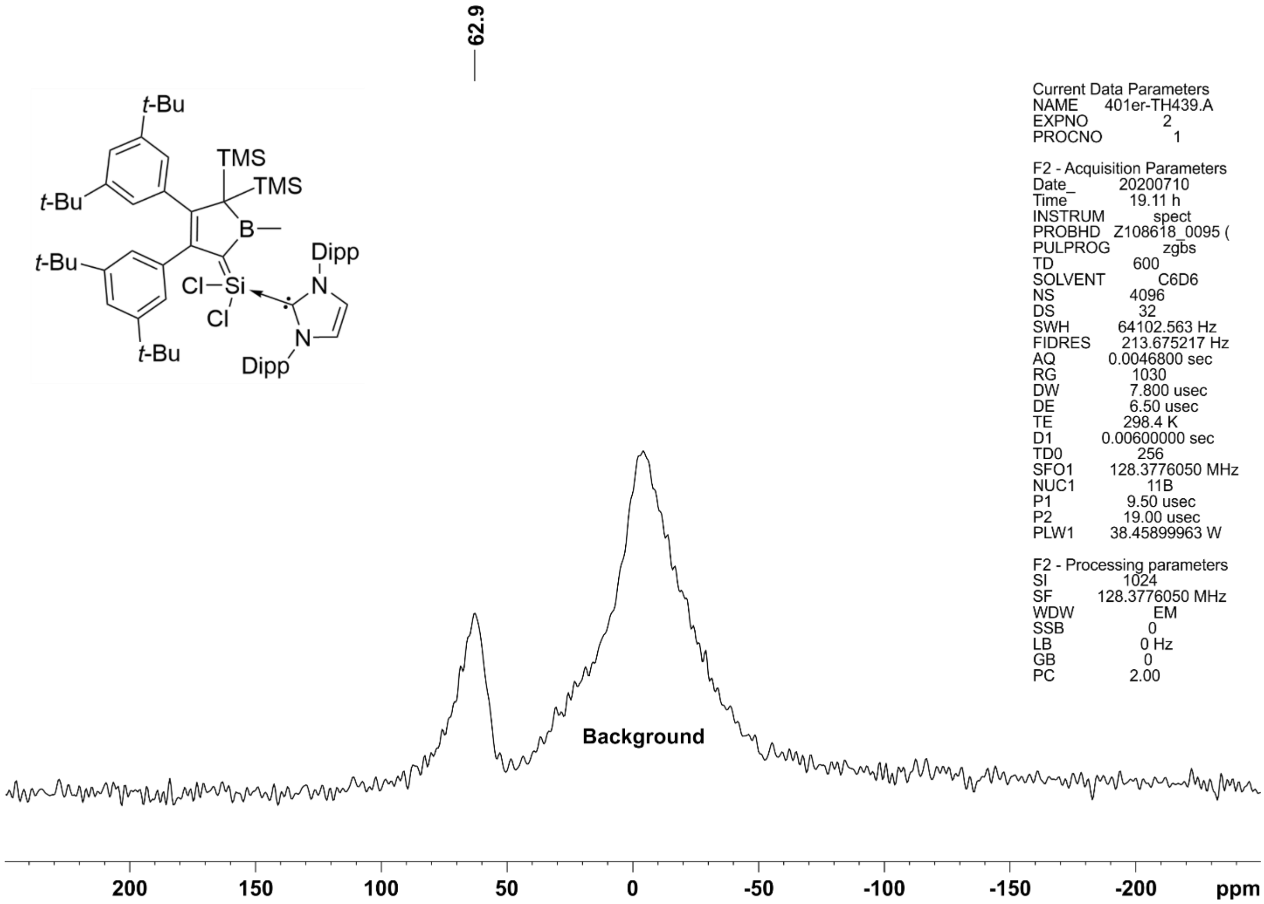

29Si-NMR (inverse gated) spectrum of compound **2b-Me** in C6D6

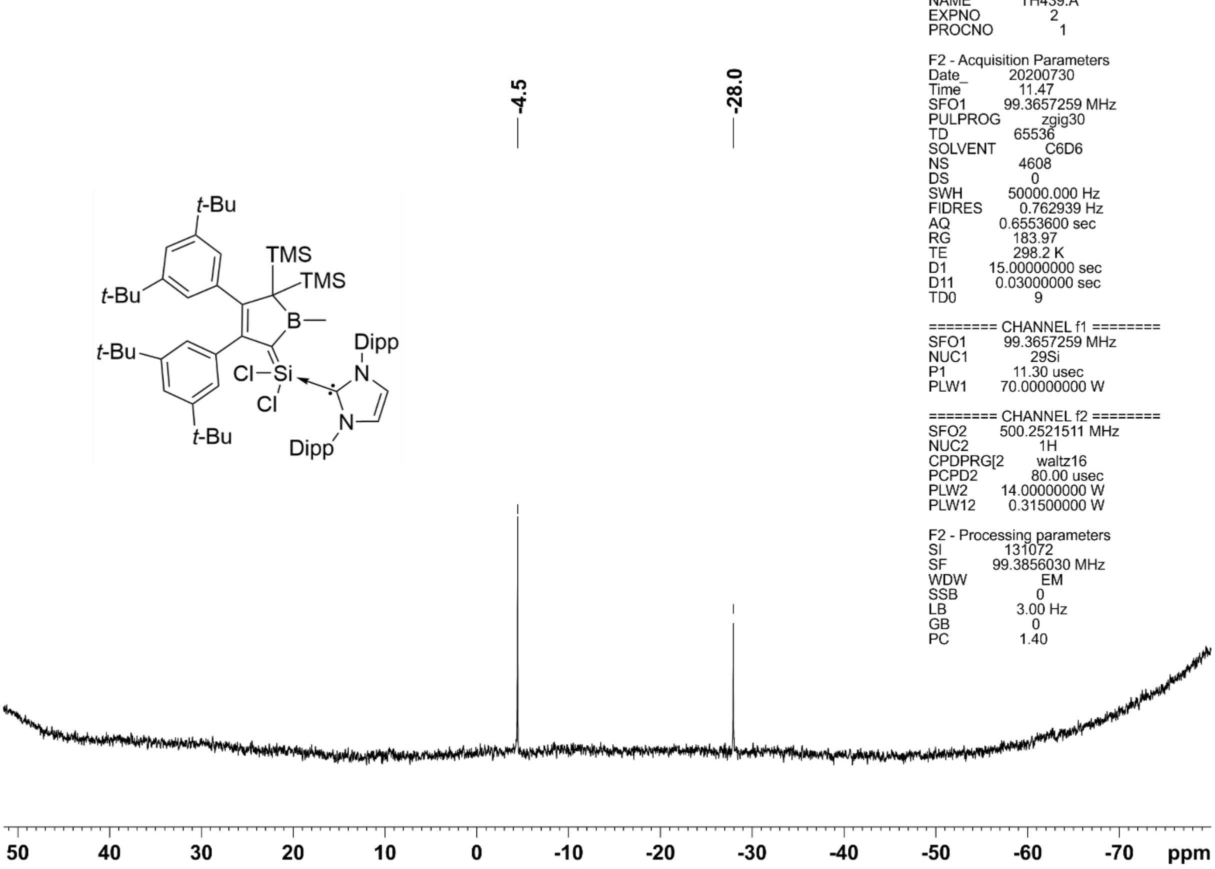

Compound 2b-Cl

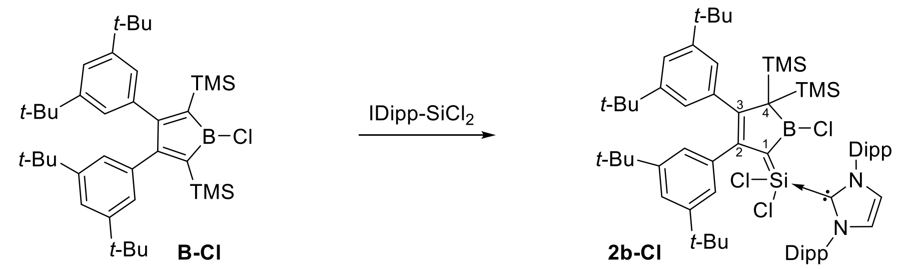

Molecular Weight: 619.3280 Molecular Weight: 1106.9120

In a glovebox, chloroborole **B-Cl** (144.4 mg, 0.233 mmol, 1 eq) and IDipp-$SiCl_2$ (113.7 mg, 0.233 mmol, 1 eq) were dissolved in dry and degassed toluene (8 mL) and the resulting claret solution was stirred overnight at ambient temperature. Subsequently the reaction mixture was filtered through a glass fiber filter (Whatman GF/B) and the solvent of the filtrate was removed under reduced pressure. The obtained solid was dissolved in hexane (16 mL), filtered through a glass fiber filter (Whatman GF/B) and the claret solution was stored for five days at –40 °C. The mother liquor was decanted with a syringe and the newly formed crystals were washed with cold hexane (–40 °C, 3 × 0.5 mL). After drying under reduced pressure, compound **2b-Cl** (188.8 mg, 0.171 mmol, 73 %) was obtained as a claret solid.

Analytical Data for Compound 2b-Cl

NMR:

^{1}H (500.25 MHz, 298 K, C_6D_6, CD_5H at 7.15 ppm): 7.19–7.21 (m, 2H, *p-H*$_{NHC}$), 7.12 (t, $^4J_{HH}$ = 1.8 Hz, 1H, *p-H$_{ar3}$*), 7.11 (t, $^4J_{HH}$ = 1.8 Hz, 1H, *p-H$_{ar2}$*), 7.06–7.09 (m, 6H, *o-H$_{ar3}$ + m-H$_{NHC}$*), 7.05 (d, $^4J_{HH}$ = 1.8 Hz, 2H, *o-H$_{ar2}$*), 6.30 (s, 2H, N-C*H*$_{NHC}$), 2.75 (sept, $^4J_{HH}$ = 6.7 Hz, 4H, C*H*$(CH_3)_2$), 1.34 (d, $^4J_{HH}$ = 6.7 Hz, 12H, CH(C*H$_3$*)$_2$), 1.31 (s, 36H, Ar$_{2/3}$-C(*Me*)$_3$), 0.87 (d, $^4J_{HH}$ = 6.7 Hz, 12H, CH(C*H$_3$*)$_2$), 0.40 (s, 18H, Si(*Me*)$_3$).

^{13}C{^{1}H} (100.65 MHz, 298 K, C_6D_6, solvent signal at 128.0 ppm): 154.9 (*C*$_{carbene}$), 150.4 (C_2), 148.0 (*m-C$_{Ar3}$*), 147.4 (*m-C$_{Ar2}$*), 145.5 (*o-C$_{NHC}$*), 144.4 (*ipso-C$_{ar2/3}$*), 143.4 (*ipso-C$_{ar2/3}$*), 134.1 (*ipso-C$_{NHC}$*), 131.9 (C_3), 131.8 (*p-C$_{NHC}$*), 127.4 (*o-C$_{ar3}$*), 126.6 (N-*C*H$_{NHC}$), 126.5 (*o-C$_{ar2}$*), 125.0 (*m-C$_{NHC}$*), 118.4 (*p-C$_{ar2}$*), 117.9 (*p-C$_{ar3}$*), 91.3 (C_1), 47.2 (C_4), 34.84 (Ar$_{2/3}$-*C*$(CH_3)_3$), 34.75 (Ar$_{2/3}$-*C*$(CH_3)_3$), 32.0 (Ar$_{2/3}$-(C(*C*H$_3$)$_3$), 31.9 (Ar$_{2/3}$-(C(*C*H$_3$)$_3$), 29.3 (*C*H$(CH_3)_2$), 26.5 (CH(*C*H$_3$)$_2$), 23.0 (CH(*C*H$_3$)$_2$), 3.0 (Si(*C*H$_3$)$_3$).

^{11}B (128.38 MHz, 298 K, C_6D_6): 52.4 ($\nu_{1/2}$ ≈ 1600 Hz).

^{29}Si (inverse gated, 99.37 MHz, 298 K, C_6D_6): –3.8 (TMS), –24.1 ($SiCl_2$).

Elemental Analysis: $C_{65}H_{96}BCl_3N_2Si_3$ calcd C 70.53, H 8.74, N 2.53; observed C 70.72, H 8.81, N 2.50.

UV/VIS (*n*-hexane)**:** λ_{max} = 500 nm (ε_{500} ≈ 470 L mol^{-1}cm^{-1})

Crystal structure of Compound 2b-Cl

2b-Cl crystallised reliably from solutions in pentane or hexane in a freezer (-40°C) and gave homogeneous crops of thin plate-shaped, red crystals. The crystals were extremely sensitive to ambient atmosphere and immediately started to loose their colour, even when suspended in perfluorinated oil within seconds (see pictures). Handling under a microscope was only possible applying a XTEMP setup supplying a stream of cold nitrogen gas over the sample. However, the crystals obtained (also from all other solvents tested) were consistently of insufficient quality to obtain data sets that allowed more than mere identification of the bonding pattern.

Cell found: triclinic, P-1; a = 14.9566(8), b = 18.4452(12), c = 29.6594(16) Å; α = 86.942(4)°, β = 88.311(3)°, γ = 76.467(3)°; V = 7942.6(8) Å^3

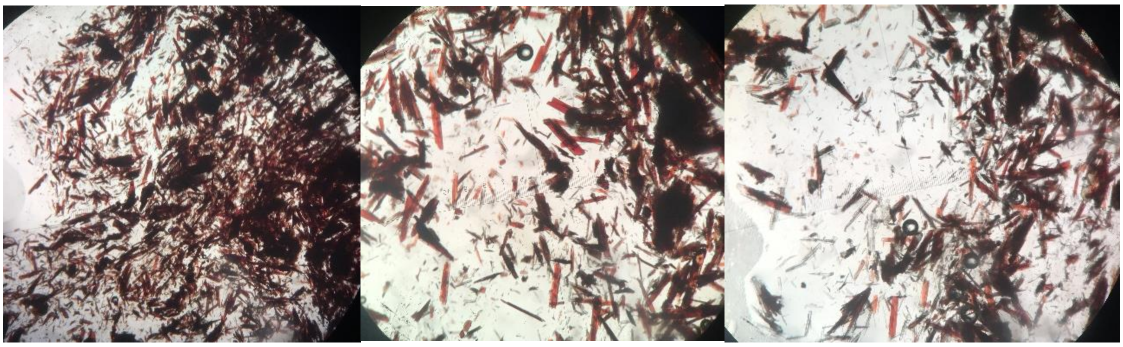

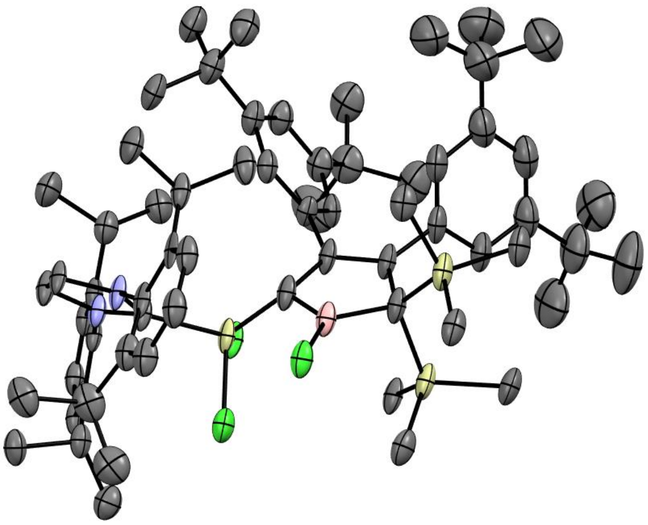

Thermal ellipsoid plot of the molecular structure of **2b-Cl**. Atomic displacement parameters are drawn at 50% probability level. Hydrogen atoms, (disordered) molecules of pentane and a second molecule in the asymmetric unit are omitted for the sake of clarity. Due to its poor data quality structure was not deposited with the CCSD.

Spectra Plots for Compound 2b-Cl

1H-NMR-spectrum of compound **2b-Cl** in C6D6
C6D5H at 7.15 ppm

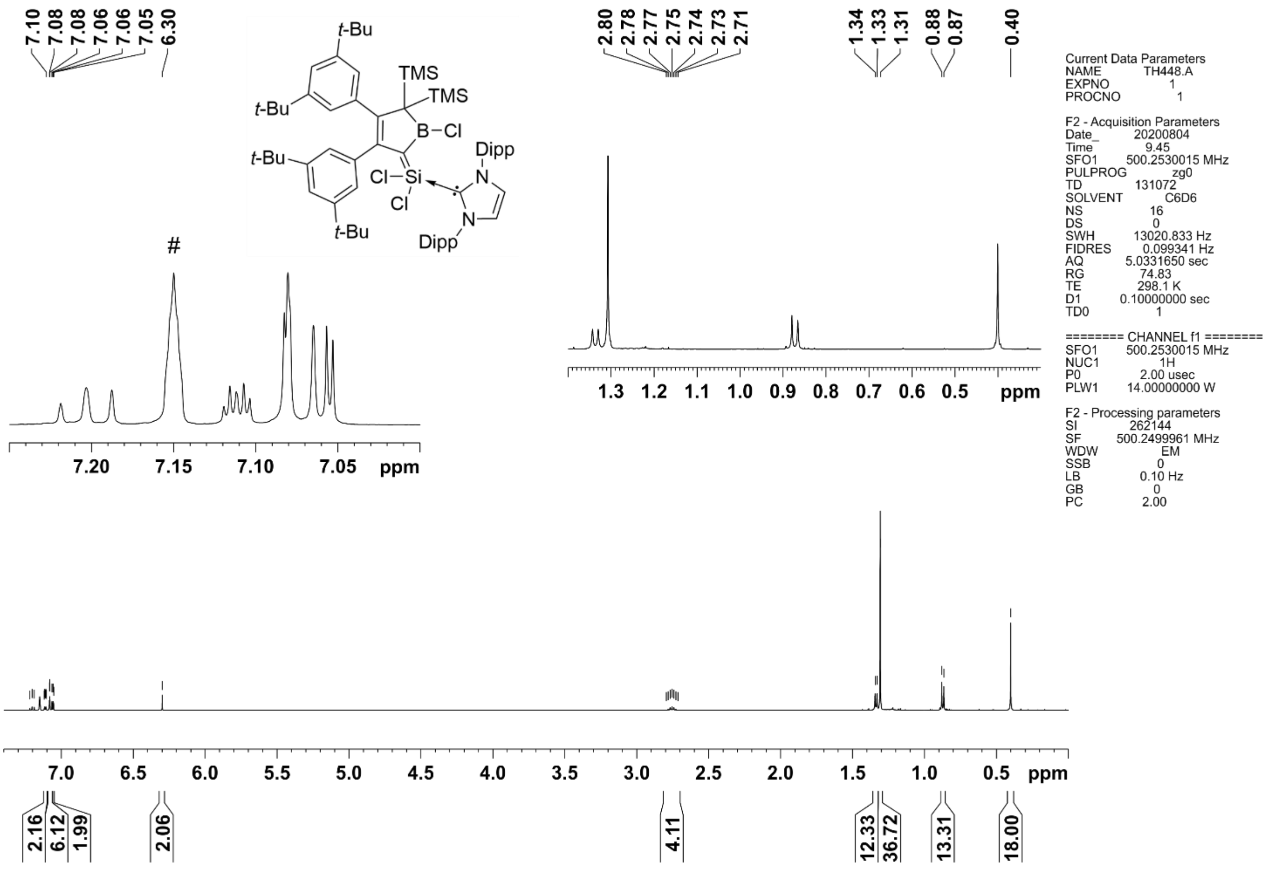

13C{1H}-NMR-spectrum of compound **2b-Cl** in C6D6
C6D6 at 128.0 ppm

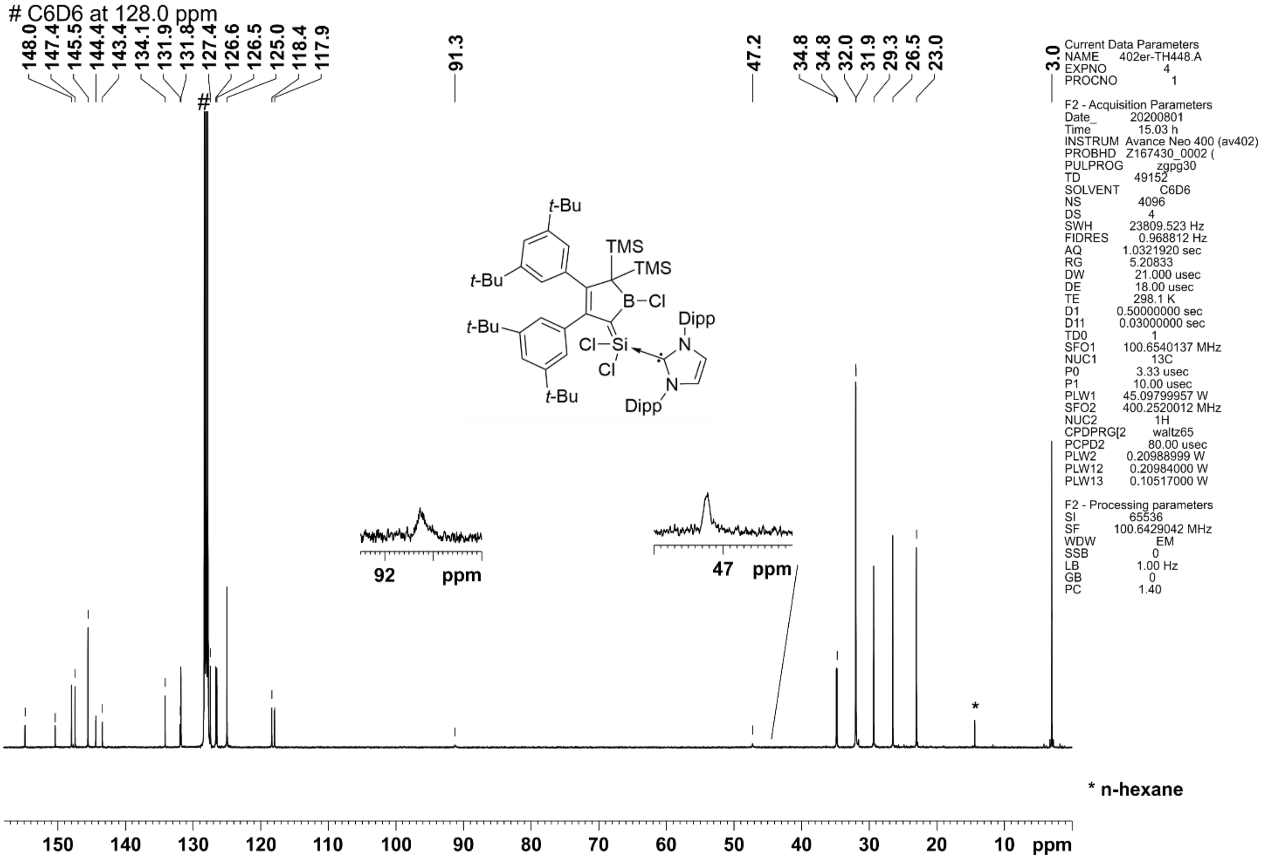

11B-NMR spectrum (background suppressed) of compound **2b-Cl** in C6D6

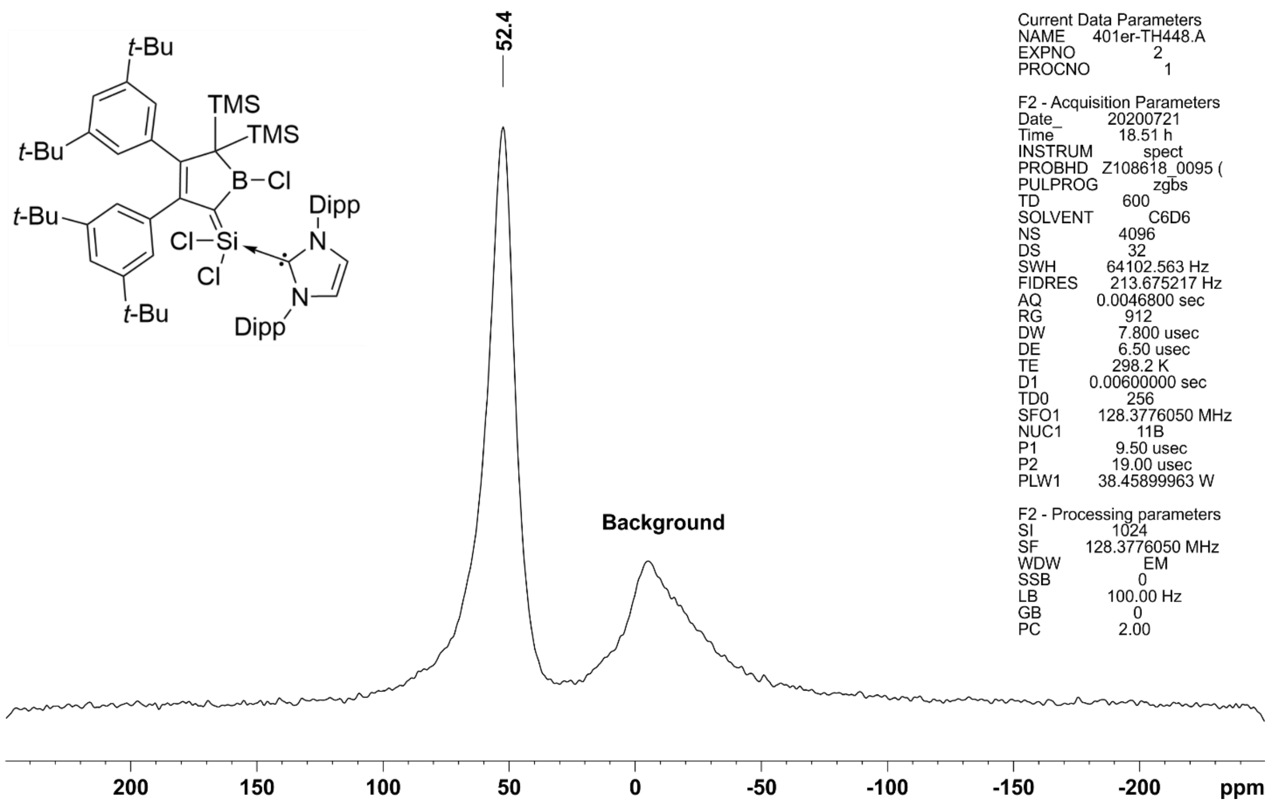

29Si-NMR (inverse gated) spectrum of compound **2b-Cl** in C6D6

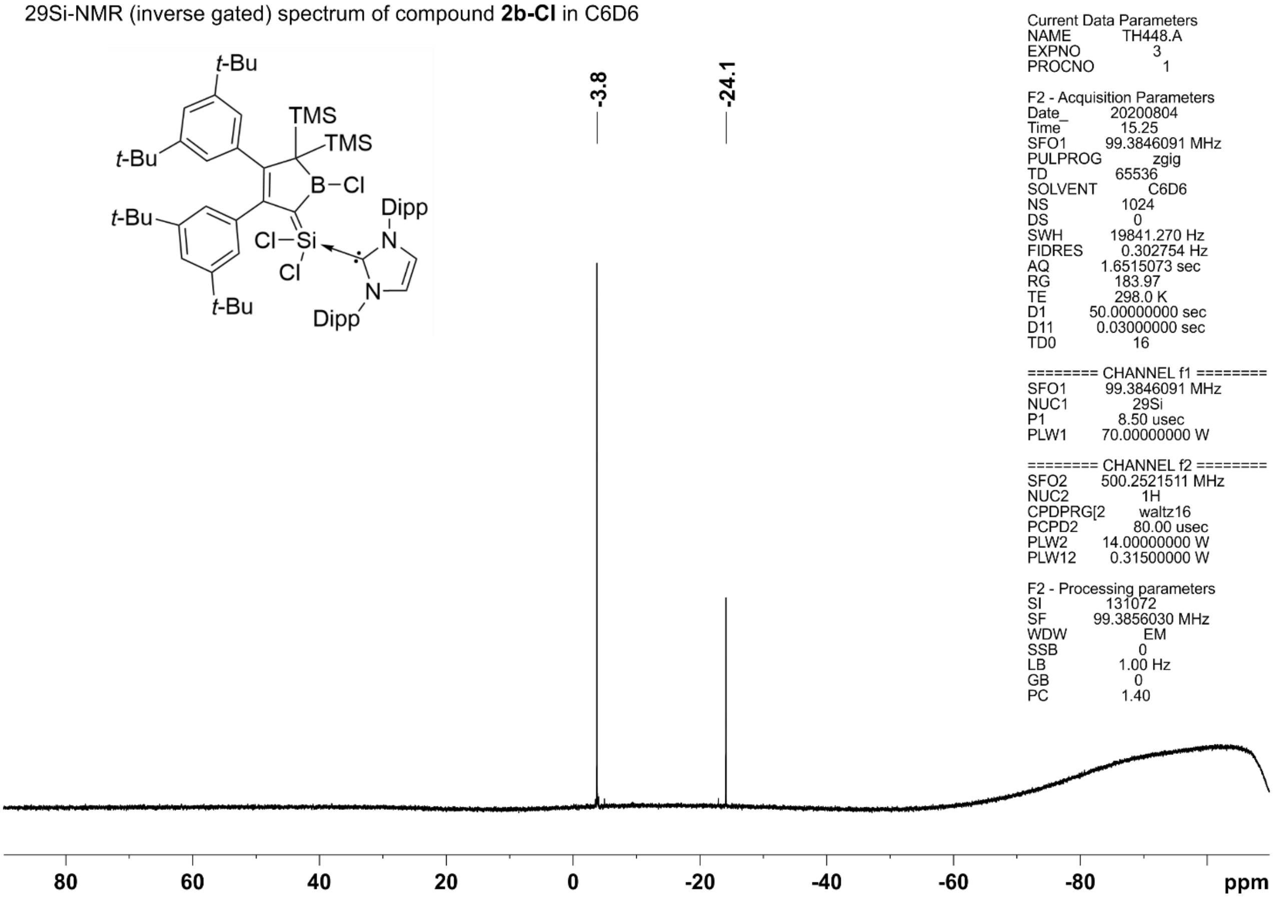

Compound 2b-Xyl

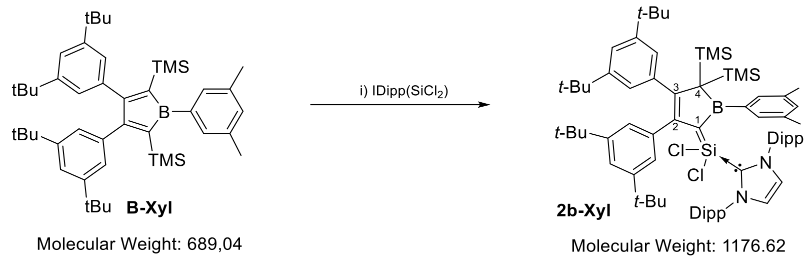

IDipp-$SiCl_2$ (111.3 mg, 0.228 mmol, 1 eq) was suspended in toluene (6 mL) and the yellow suspension was cooled to –78 °C. At this temperature, a –78 °C cold solution of **B-Xyl** (157.3 mg, 0.228 mmol, 1 eq) in toluene (3 mL) was added, giving a dark red reaction mixture. The reaction was allowed to warm to –20 °C over the course of two hours and the solvent was afterwards removed under reduced pressure. In a glovebox, the resulting red solid was washed with pentane (3 x 0.5 mL) and the remaining solid was dissolved in THF (1.2 mL) and stored at –40 °C for two days. The so formed red crystals were isolated, washed with cold pentane (–40 °C, 3 x 0.2 mL) and dried under reduced pressure, giving compound **2b-Xyl** (96.5 mg, 0.082 mmol, 36 %) as a red solid.

Note: Compound **2b-Xyl** is not stable in solution and slowly rearranges at ambient temperature to compound **1b-Xyl** over the course of several weeks. In the solid state, however, compound **2b-Xyl** was found to be stable for at least two weeks. As compound **2b-Xyl** represents the kinetically favored product of the reaction between **B-Xyl** and IDipp-SiCl2, the product ratio between compound **2b-Xyl** and compound **1b-Xyl** can be controlled by the reaction temperature (see table 1-SI).

Table 1-SI: Observed product ratios of the reaction of compound **B-Xyl** with compound **IDipp($SiCl_2$)** at different temperatures. Product ratios were calculated based on the ^{1}H-NMR spectrum of the crude reaction mixtures.

Temperature	Compound **2b-Xyl** [%]	Compound **1b-Xyl** [%]
Ambient temperature	45	55
–40 °C	85	15
–78 °C	95	5

Analytical Data for Compound 2b-Xyl

NMR:

^{1}H (400.13 MHz, 297 K, C_6D_6, CD_5H at 7.15 ppm): 7.51–7.16 (m, 5H), 7.15 (1H, *p-H*$_{ar2/3}$, partially overlapped by the solvent signal), 7.12–6.90 (m, 6H), 7.07 (t, $^4J_{HH}$ = 1.7 Hz, 1H, *p-H*$_{ar2/3}$), 6.79 (s, 1H, *p-H*$_{Xyl}$), 6.04 (s, 2H, N-C*H*$_{NHC}$), 2.34 (br s, 10H, C*H*$_3$ + C*H*(CH_3)$_2$), 1.52–1.15 (br, 48H, Ar-C(*Me*)$_3$ + CH(C*H*$_3$)$_2$), 0.76 (d, $^3J_{HH}$ = 6.8 Hz, 12H, CH(C*H*$_3$)$_2$), 0.28 (s, 18H, Si(*Me*)$_3$). Signals corresponding to *o-H*$_{NHC}$ + *p-H*$_{NHC}$ + *p-H*$_{Xyl}$ + *o-H*$_{ar2/3}$ could not be assigned unequivocally.

^{13}C{^{1}H} (100.62 MHz, 298 K, C_6D_6, solvent signal at 128.0 ppm): 155.5 (*C*$_{NHC}$), 149.7, 148.4, 146.8, 144.2, 144.1, 134.7, 133.7, 131.3 (*o-C*$_{ar2/3}$), 128.7, 126.43 (*p-C*$_{Xyl}$), 126.35 (N-*C*H$_{NHC}$), 125.2 (*o-C*$_{ar2/3}$), 118.9 (*p-C*$_{ar2/3}$), 118.4 (*p-C*$_{ar2/3}$), 93.5 (C_1), 52.3 (C_4), 35.0 (Ar$_{2/3}$-*C*(CH_3)$_3$), 34.9 (Ar$_{2/3}$-*C*(CH_3)$_3$), 32.1 (Ar$_{2/3}$-(C(*C*H$_3$)$_3$), 29.3 (*C*H(CH_3)$_2$), 25.6 (CH(*C*H$_3$)$_2$), 22.9 (CH(*C*H$_3$)$_2$), 22.2 (*C*H$_3$), 5.2 (Si(*C*H$_3$)$_3$).Signals corresponding to C_2 + C_3 + *ipso-C*$_{ar2/3}$ + *m-C*$_{ar2/3}$ + *ipso-C*$_{Xyl}$ + *o-C*$_{Xyl}$ + *m-C*$_{Xyl}$ + *ipso-C*$_{NHC}$ + *o-C*$_{NHC}$ + *m-C*$_{NHC}$ + *p-C*$_{NHC}$ could not be assigned unequivocally.

^{11}B (128.38 MHz, 296 K, C_6D_6): 57.4 ($\nu_{1/2}$ ≈ 1700 Hz).

^{29}Si (inverse gated, 79.49 MHz, 297 K, C_6D_6): –4.4 (TMS), –25.2 (*Si*Cl_2).

Elemental Analysis: $C_{73}H_{105}BN_2Si_3Cl_2$ calcd C 74.52, H 9.00, N 2.38; observed C 75.12, H 9.66, N 2.41.

UV/VIS (toluene): λ_{max} = 487 nm (ε_{487} ≈ 730 L mol^{-1}cm^{-1})

Spectra Plots for Compound 2b-Xyl (RT) and mixtures of 2b-Xyl and 1b-Xyl (70°C)

1H-NMR-spectrum of compound **2b-Xyl** in C6D6 (ambient temperature)

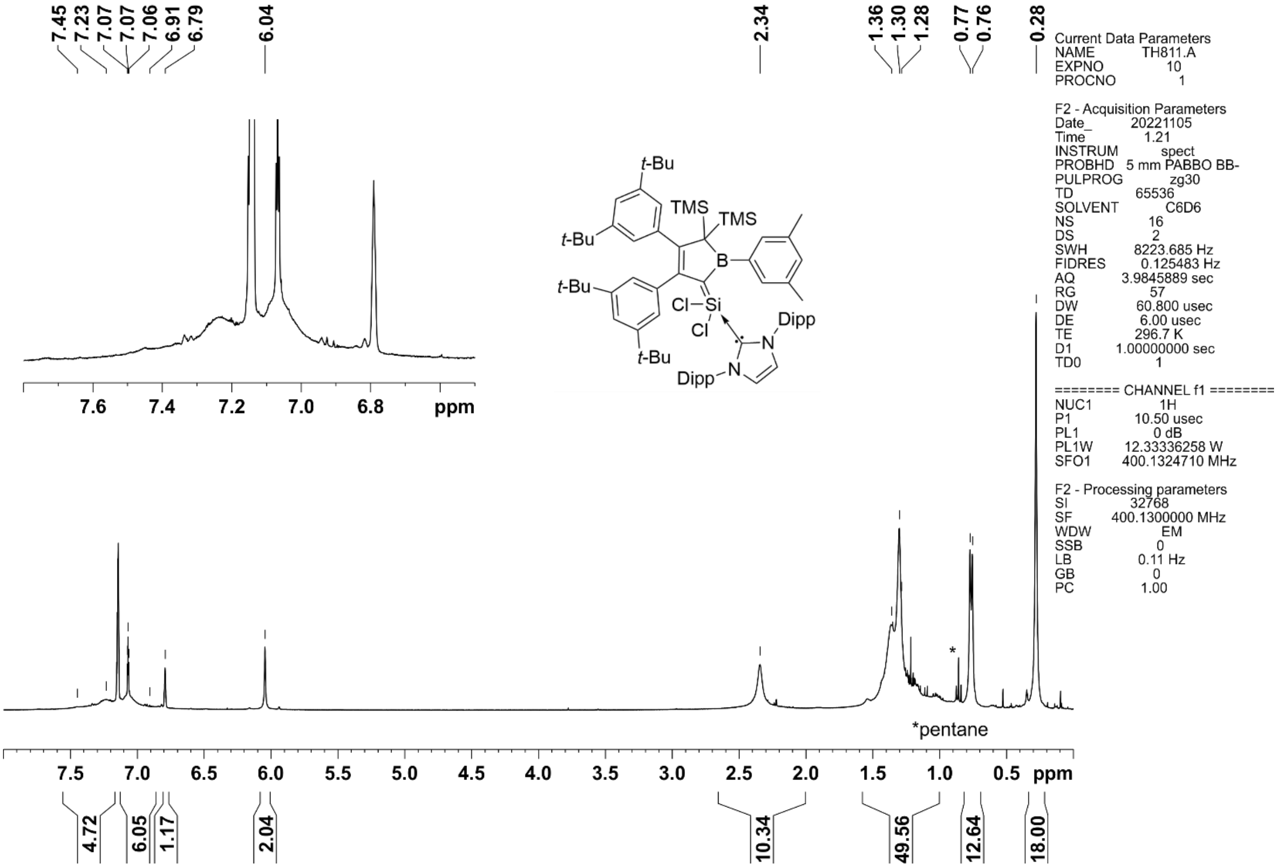

1H-NMR-spectrum of compound **2b-Xyl** in C6D6 at 343 K (70°C)

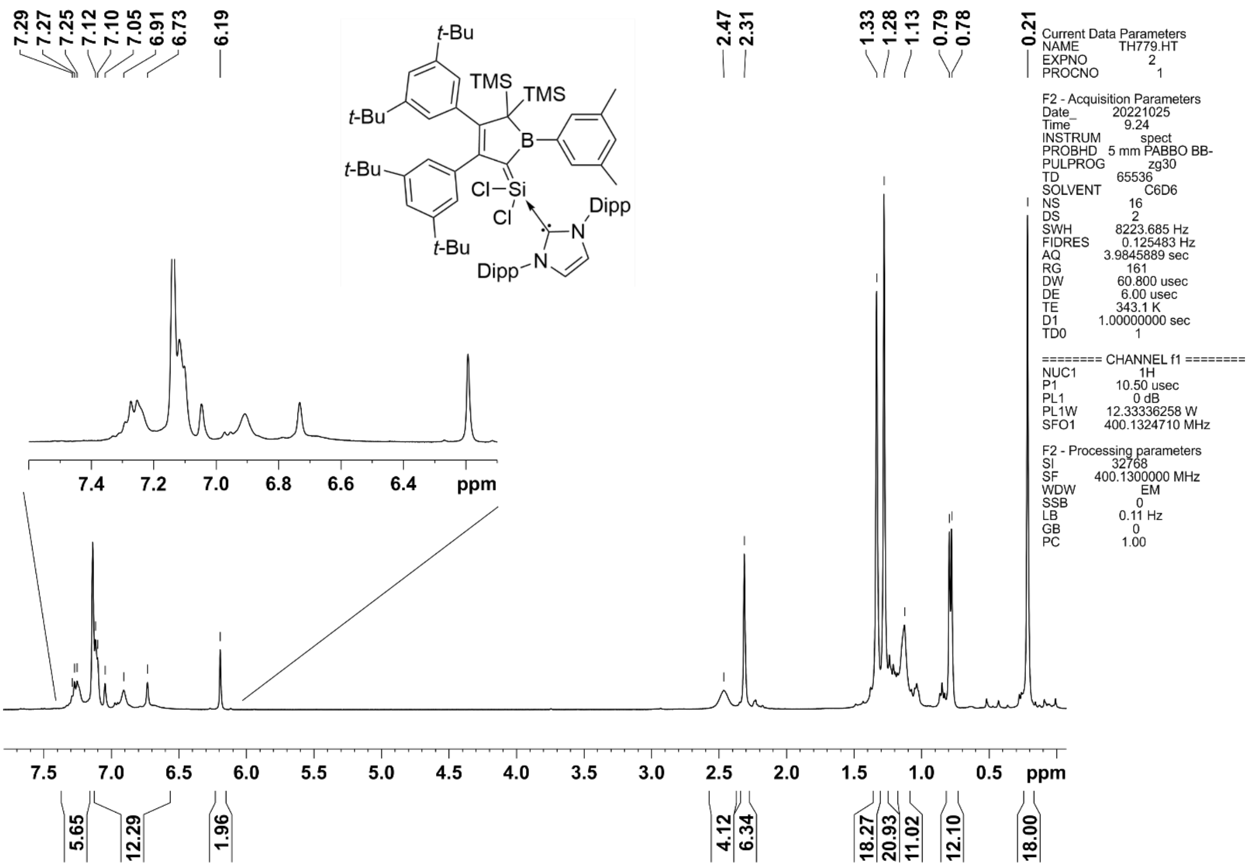

13C{1H}-NMR-spectrum of compound **2b-Xyl** in C6D6 at ambient temperature

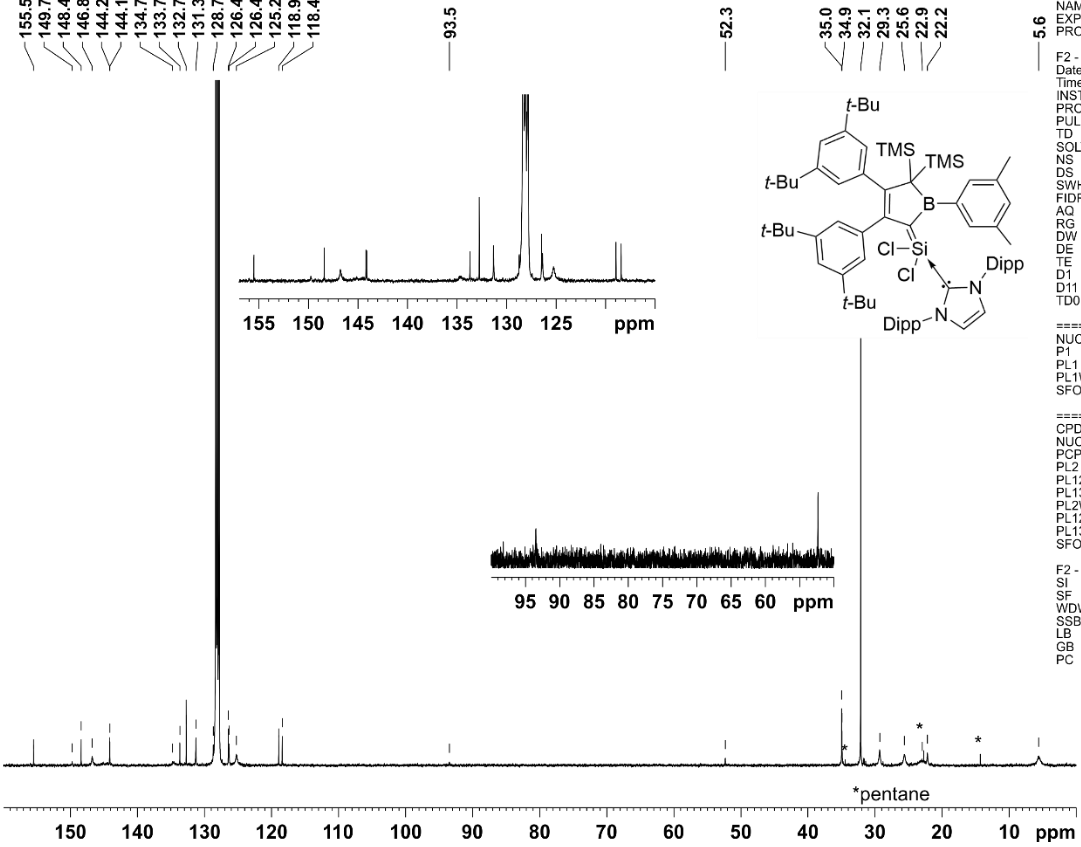

Current Data Parameters
NAME TH811.A
EXPNO 15
PROCNO 1

F2 - Acquisition Parameters
Date_ 20221105
Time 13.03
INSTRUM spect
PROBHD 5 mm PABBO BB-
PULPROG zgpg30
TD 65536
SOLVENT C6D6
NS 8192
DS 4
SWH 24038.461 Hz
FIDRES 0.366798 Hz
AQ 1.3631488 sec
RG 512
DW 20.800 usec
DE 6.50 usec
TE 298.1 K
D1 2.00000000 sec
D11 0.03000000 sec
TD0 1

======== CHANNEL f1 ========
NUC1 13C
P1 6.50 usec
PL1 0 dB
PL1W 91.43266296 W
SFO1 100.6228298 MHz

======== CHANNEL f2 ========
CPDPRG[2 waltz16
NUC2 1H
PCPD2 80.00 usec
PL2 -0.25 dB
PL12 17.80 dB
PL13 17.80 dB
PL2W 13.06416035 W
PL12W 0.20468290 W
PL13W 0.20468290 W
SFO2 400.1316005 MHz

F2 - Processing parameters
SI 32768
SF 100.6127299 MHz
WDW EM
SSB 0
LB 1.00 Hz
GB 0
PC 1.40

13C{1H}-NMR-spectrum of compound **2b-Xyl** in C6D6 at 343 K (70°C)

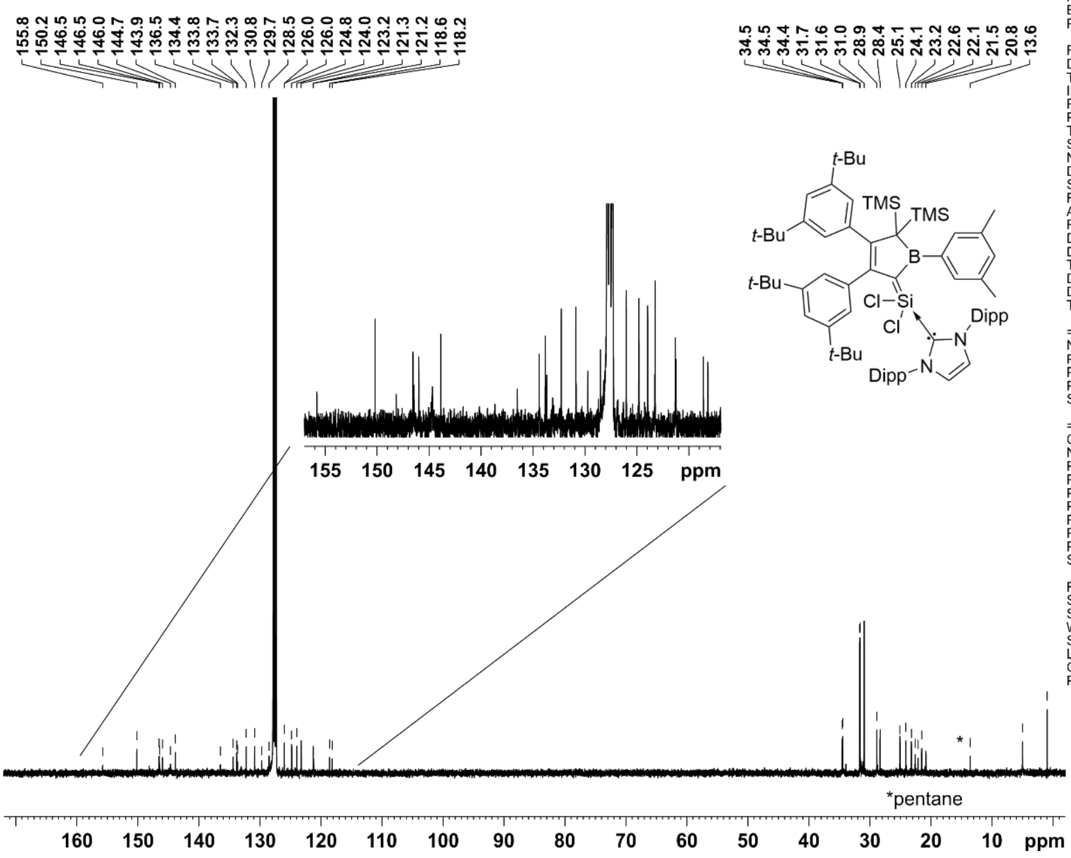

Current Data Parameters
NAME TH779.HT
EXPNO 5
PROCNO 1

F2 - Acquisition Parameters
Date_ 20221025
Time 10.01
INSTRUM spect
PROBHD 5 mm PABBO BB-
PULPROG zgpg30
TD 65536
SOLVENT C6D6
NS 512
DS 4
SWH 24038.461 Hz
FIDRES 0.366798 Hz
AQ 1.3631488 sec
RG 912
DW 20.800 usec
DE 6.50 usec
TE 343.3 K
D1 2.00000000 sec
D11 0.03000000 sec
TD0 1

======== CHANNEL f1 ========
NUC1 13C
P1 6.50 usec
PL1 0 dB
PL1W 91.43266296 W
SFO1 100.6228298 MHz

======== CHANNEL f2 ========
CPDPRG[2 waltz16
NUC2 1H
PCPD2 80.00 usec
PL2 -0.25 dB
PL12 17.80 dB
PL13 17.80 dB
PL2W 13.06416035 W
PL12W 0.20468290 W
PL13W 0.20468290 W
SFO2 400.1316005 MHz

F2 - Processing parameters
SI 262144
SF 100.6127690 MHz
WDW EM
SSB 0
LB 0.60 Hz
GB 0
PC 1.40

11B-NMR spectrum (background suppressed) of compound **2b-Xyl** in C6D6 at ambient temperature

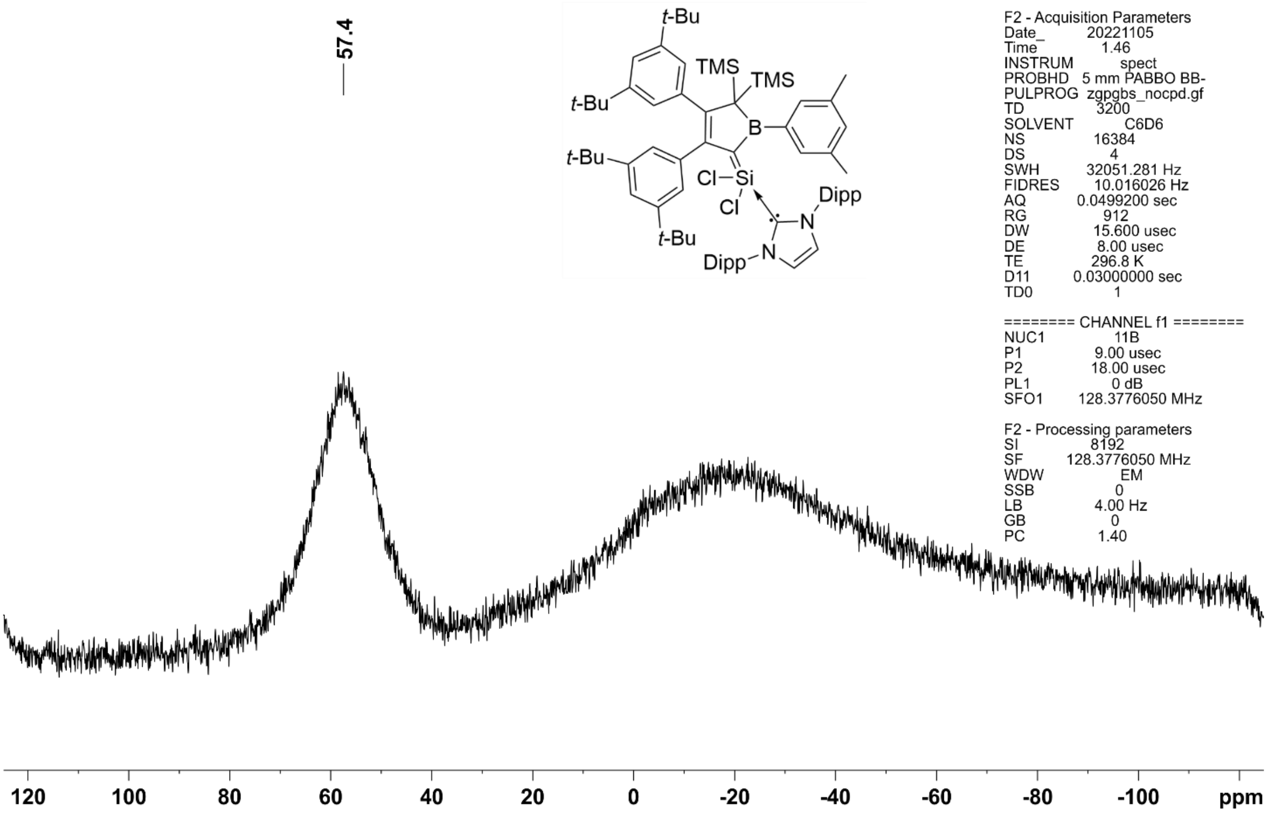

11B-NMR spectrum (background suppressed) of compound **2b-Xyl** in C6D6 at 343 K (70°C)

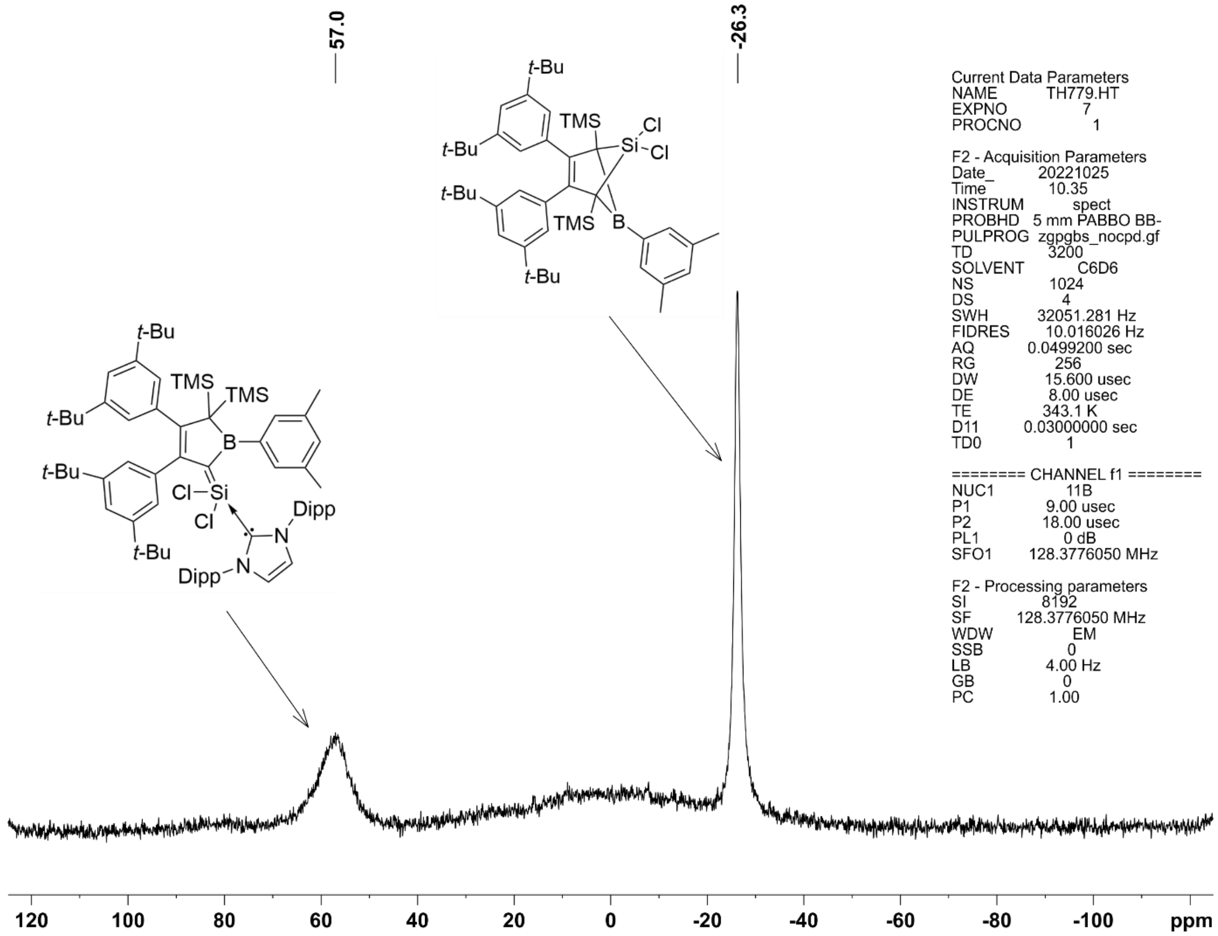

29Si-NMR (inverse gated) spectrum of compound **2b-Xyl** in C6D6 at ambient temperature

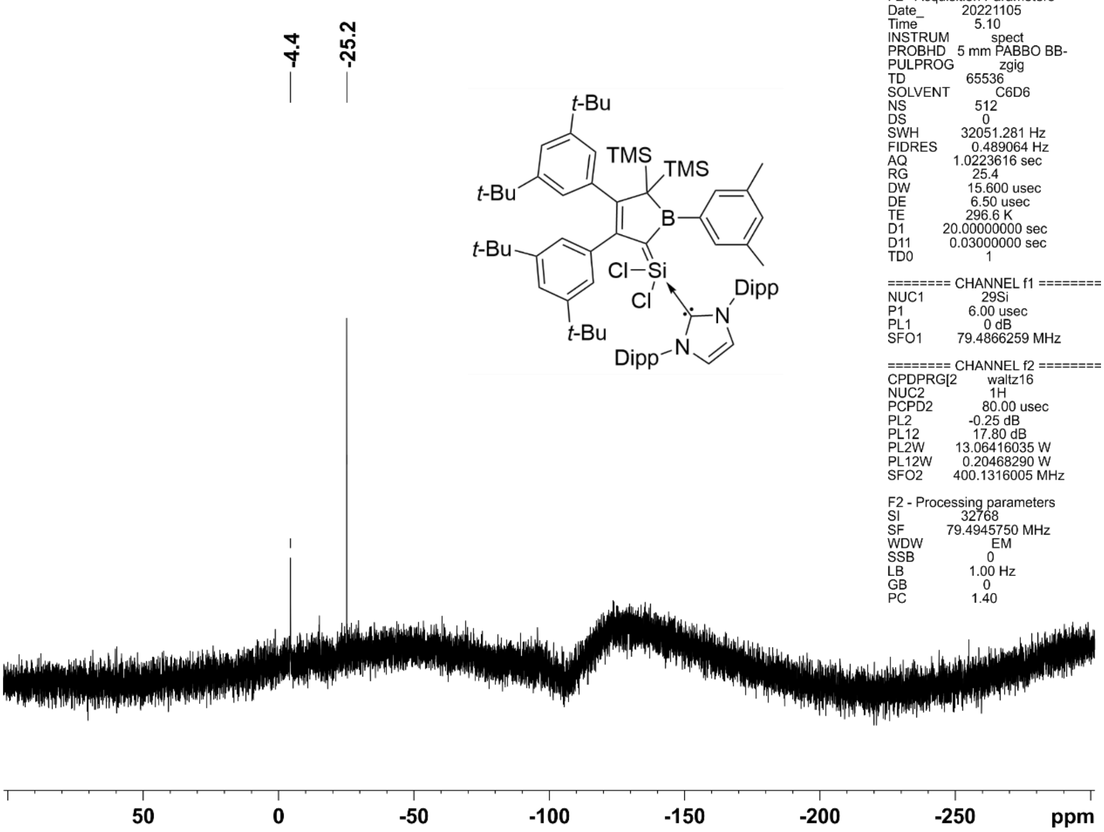

Current Data Parameters
NAME TH811.A
EXPNO 13
PROCNO 1

F2 - Acquisition Parameters
Date_ 20221105
Time 5.10
INSTRUM spect
PROBHD 5 mm PABBO BB-
PULPROG zgig
TD 65536
SOLVENT C6D6
NS 512
DS 0
SWH 32051.281 Hz
FIDRES 0.489064 Hz
AQ 1.0223616 sec
RG 25.4
DW 15.600 usec
DE 6.50 usec
TE 296.6 K
D1 20.00000000 sec
D11 0.03000000 sec
TD0 1

======== CHANNEL f1 ========
NUC1 29Si
P1 6.00 usec
PL1 0 dB
SFO1 79.4866259 MHz

======== CHANNEL f2 ========
CPDPRG[2 waltz16
NUC2 1H
PCPD2 80.00 usec
PL2 -0.25 dB
PL12 17.80 dB
PL2W 13.06416035 W
PL12W 0.20468290 W
SFO2 400.1316005 MHz

F2 - Processing parameters
SI 32768
SF 79.4945750 MHz
WDW EM
SSB 0
LB 1.00 Hz
GB 0
PC 1.40

Si29-NMR (DEPT20) spectrum of compound **2b-Xyl** in C6D6 at 343 K (70°C)

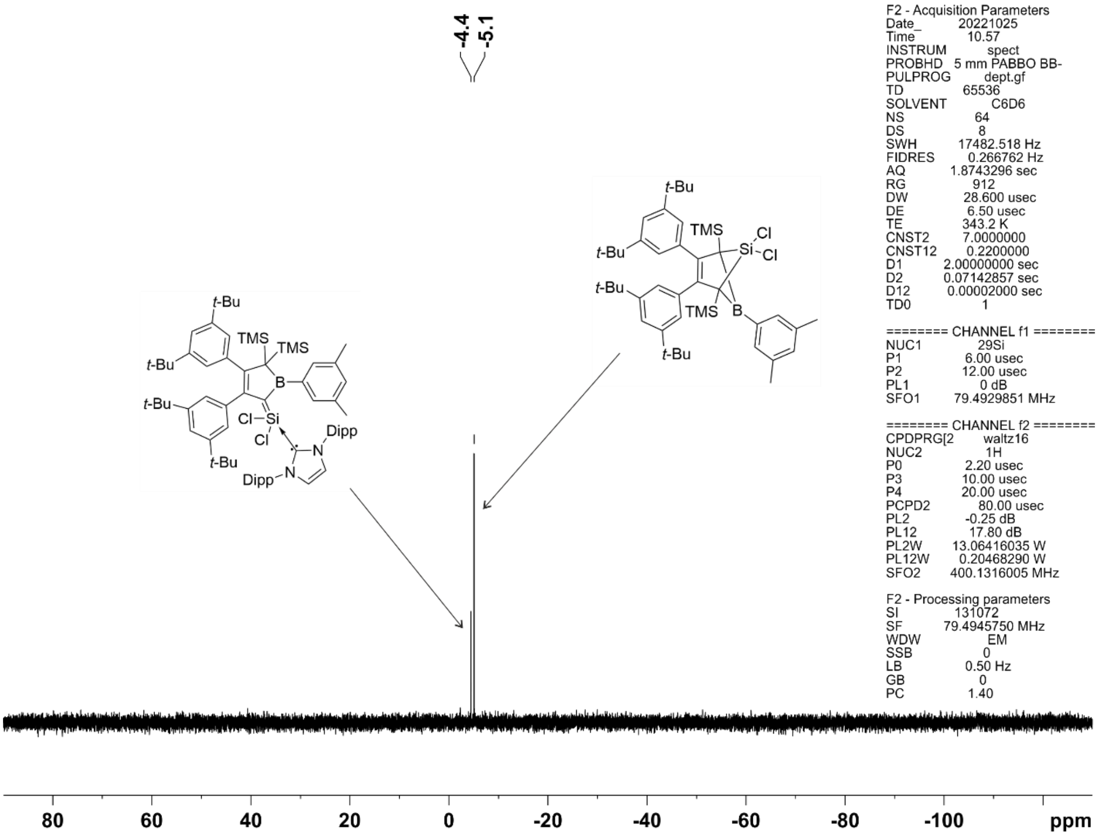

Current Data Parameters
NAME TH779.HT
EXPNO 10
PROCNO 1

F2 - Acquisition Parameters
Date_ 20221025
Time 10.57
INSTRUM spect
PROBHD 5 mm PABBO BB-
PULPROG dept.gf
TD 65536
SOLVENT C6D6
NS 64
DS 8
SWH 17482.518 Hz
FIDRES 0.266762 Hz
AQ 1.8743296 sec
RG 912
DW 28.600 usec
DE 6.50 usec
TE 343.2 K
CNST2 7.0000000
CNST12 0.2200000
D1 2.00000000 sec
D2 0.07142857 sec
D12 0.00002000 sec
TD0 1

======== CHANNEL f1 ========
NUC1 29Si
P1 6.00 usec
P2 12.00 usec
PL1 0 dB
SFO1 79.4929851 MHz

======== CHANNEL f2 ========
CPDPRG[2 waltz16
NUC2 1H
P0 2.20 usec
P3 10.00 usec
P4 20.00 usec
PCPD2 80.00 usec
PL2 -0.25 dB
PL12 17.80 dB
PL2W 13.06416035 W
PL12W 0.20468290 W
SFO2 400.1316005 MHz

F2 - Processing parameters
SI 131072
SF 79.4945750 MHz
WDW EM
SSB 0
LB 0.50 Hz
GB 0
PC 1.40

Compound 3a-*p*Xyl

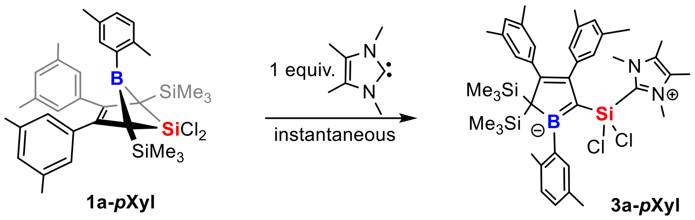

Molecular Weight: 619,70 Molecular Weight: 743,89

A solution of 1,3,4,5-tetramethylimidazol-2-ylidene (21.0 mg, 0.1691 mmol, 1 eq.) in toluene (1 mL) was added to a stirred solution of the bicyclic compound **1a-*p*Xyl** (105.3 mg, 0.1699 mmol, 1 eq.) in toluene (2 mL). The toluene was removed in vacuo from the immediately red solution to give a red sticky oil. Pentane was repeatedly added in small portions (1-2 mL) and subsequently removed under reduced pressure until a lightly orange powder was obtained. The powder was then washed with a mixture of pentane (4 mL) and a few drops dichloromethane. Pentane was then again repeatedly added in small portions and subsequently removed to obtain a yellow powder (33.9 mg). The solvent was removed from the washing solution and the residue was dissolved in pentane (2 mL) and a few drops of dichloromethane. The solution was then stored at -40 °C for a few hours to give a yellow precipitate. The supernatant was decanted off and the precipitate was dried in vacuum to give a second crop of **3a-*p*Xyl** as yellow powder (17.3 mg). (51.2 mg combined yields, 0.0688 mmol, 41 %)

Analytical Data for Compound 3a-*p*Xyl

NMR:

^{1}H (400.13 MHz, 297 K, C_6D_6, C_6D_5H at 7.15 ppm): 7.27 (s, 2H, TMS-side: *o-H*), 7.21 (d, 1H, ^4J = 1.24 Hz, B-Aryl: *o-H*), 7.10 (m, 2H, NHC-side: *o-H*), 6.95 (d, 1H, , ^3J = 7.63 Hz, B-Aryl: *m-H*), 6.71 (dd, 1H, ^3J = 7.63 Hz , ^4J = 1.55 Hz, B-Aryl: *p-H*), 6.62 (s, 1H, TMS-side: *p-H*), 6.40 (s, 1H, NHC-side: *p-H*), 3.04 (s, 6H, N- C*H*$_3$), 2.81 (s, 3H, B-Aryl: *o-* C*H*$_3$), 2.19 (s, 6H, TMS-side: *m*-C*H*$_3$), 2.10 (s, 3H, B-Aryl: *m*-C*H*$_3$), 2.02 (s, 6H, NHC-side: *m*-C*H*$_3$), 0.92 (s, 6H, *H*$_3$C-RC=CR-C*H*$_3$), 0.49 (s, 18H, TMS).

^{13}C{^{1}H} (100.62 MHz, 298 K, C_6D_6 solvent signal at 128.0 ppm): 150.6 (B-Aryl: : *ipso-C$_{ar}$*), 147.1 (N-*C*-N), 145.7; 144.5 (*ipso-C$_{ar}$*), 137.4 (B-Aryl: *o-C*-CH$_3$), 135.6 (NHC-side: *m-C$_{ar}$*), 135.0 (NHC-side: *C$_β$*; uncertain assignmet), 134.8 (TMS-side: *m-C$_{ar}$*), 133.9 (B-Aryl: *o*-CH), 132.1 (TMS-side: *o-C$_{ar}$*), 131.0 (TMS-side: *C$_β$*), 130.6 (B-Aryl: *m-C*- CH$_3$), 128.9 (B-Aryl: *m-C*H), 128.6 (NHC-side: *o-C$_{ar}$*), 126.8 (TMS-side: *p-C$_{ar}$*), 126.6 (N-*C*=*C*-N), 126.1 (NHC-side: *p-C$_{ar}$*), 125.3 (B-Aryl: : *p-C$_{ar}$*), 101.9 (NHC-side: *C$_α$*), 51.8 (TMS-side: *C$_α$*), 34.3 (N- *C*H$_3$), 25.1 (B-Aryl: o-*C*H$_3$), 21.5 (B-Aryl: *m-C*H$_3$), 21.4 (TMS-side: Ar-Me), 21.4 (NHC-side: Ar-Me), 7.6 (H$_3$*C*-RC=CR-*C*H$_3$), 4.6 (TMS).

^{11}B (128.37 MHz, 297 K, C_6D_6): 54.8 ($\omega_{1/2}$ = 468 Hz).

^{29}Si (79.49 MHz, 297 K, C_6D_6): -4.6; -5.0 (TMS), -19.9 ($SiCl_2$).

Elemental Analysis: ($C_{53}H_{73}BCl_3N_2Si_3$) calcd C 66.20, H 7.72, B 1.45, Cl 9.53, N 3.77, Si 11.31, observed C 64.75, H 7.64, N 4.29.

UV-*vis*: (in toluene) λ_{max} = 423 nm (shoulder) (ε_{423} = 700 L mol^{-1}cm^{-1}).

Spectra Plots for Compound 3a-*p*Xyl

1H-NMR-spectrum of compound **3a-*p*Xyl** in C6D6
C6D5H at 7.15 ppm

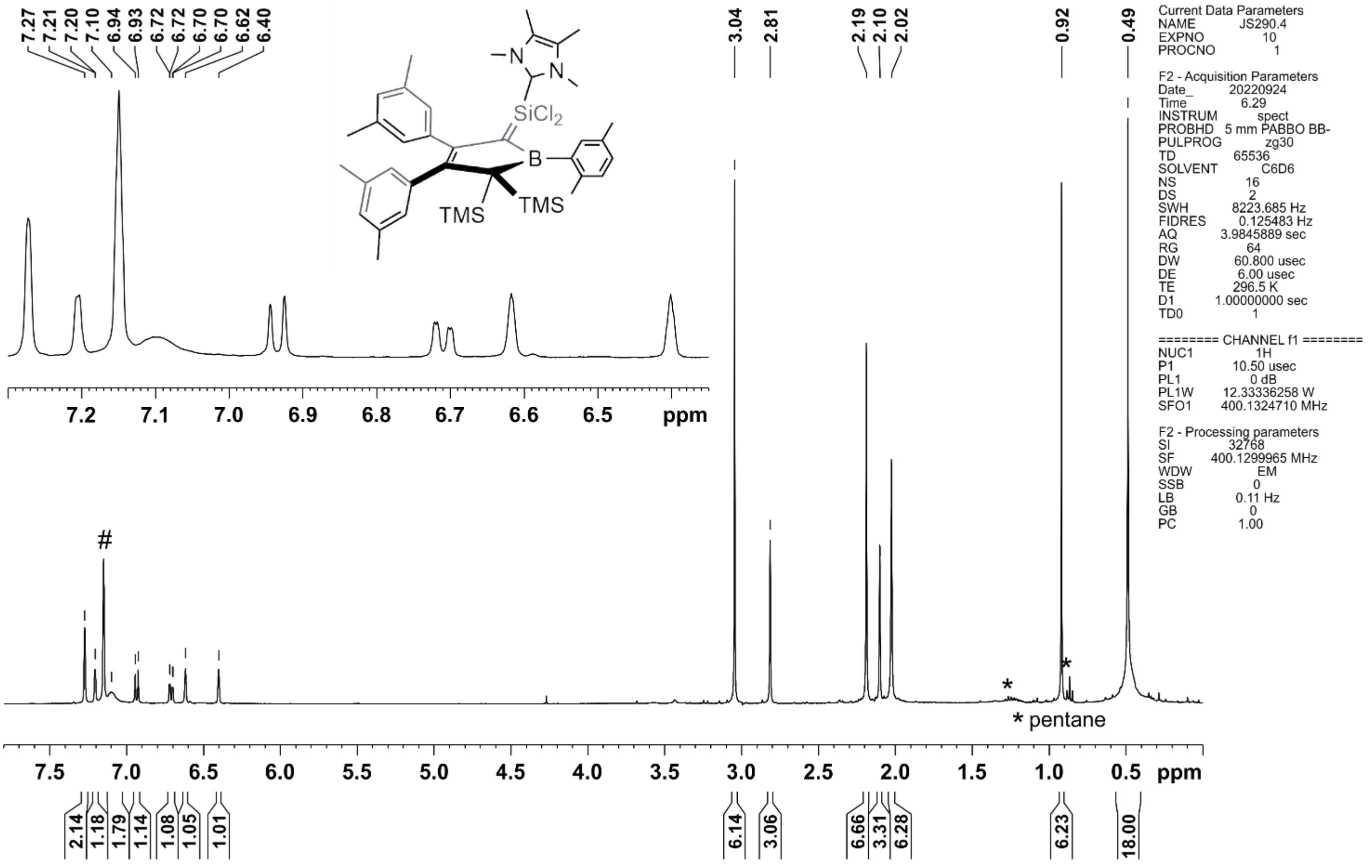

13C{1H}-NMR-spectrum of compound **3a-*p*Xyl** in C6D6
C6D6 at 128.0 ppm

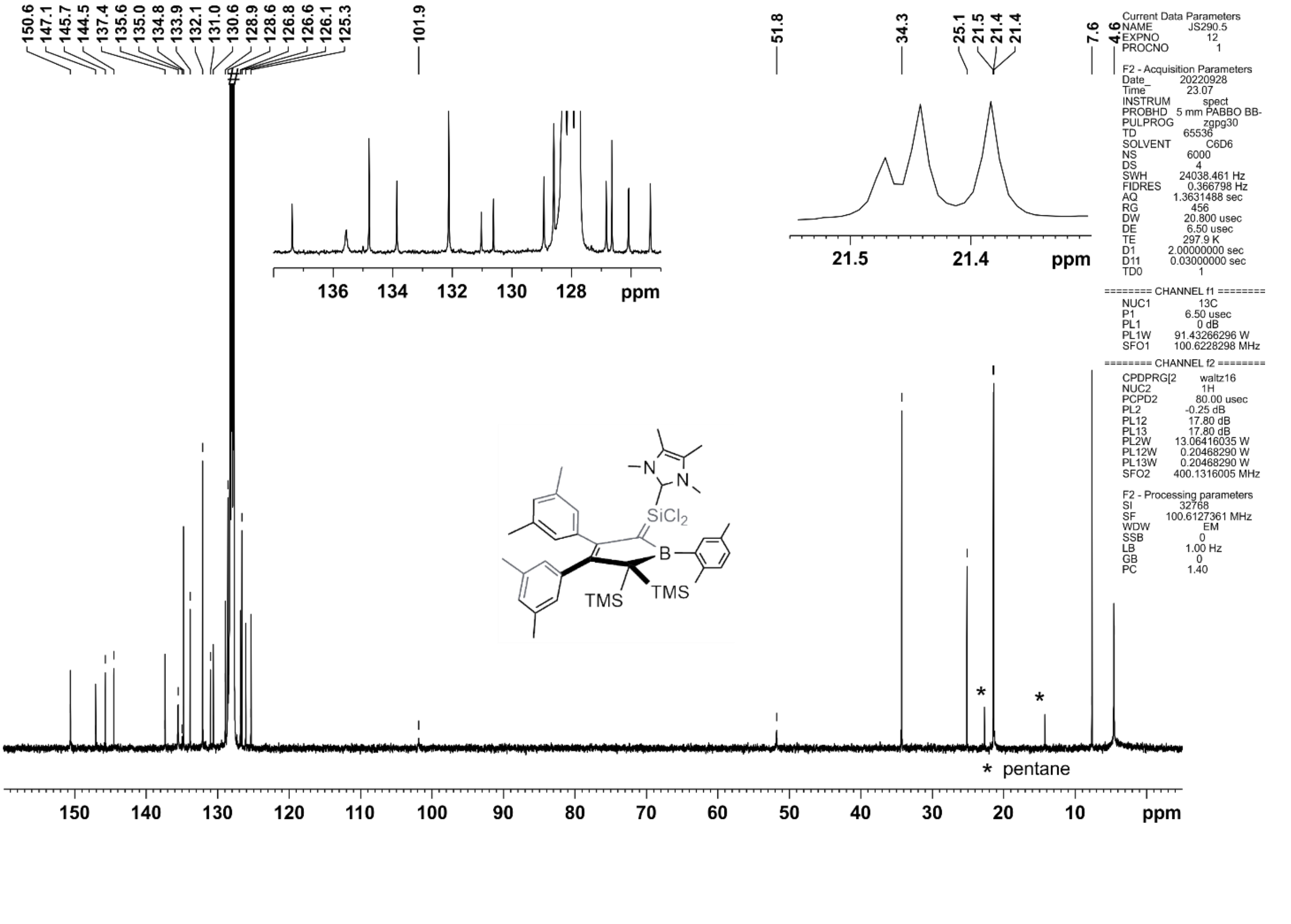

11B-NMR spectrum (background suppressed) of compound **3a-*p*Xyl** in C6D6

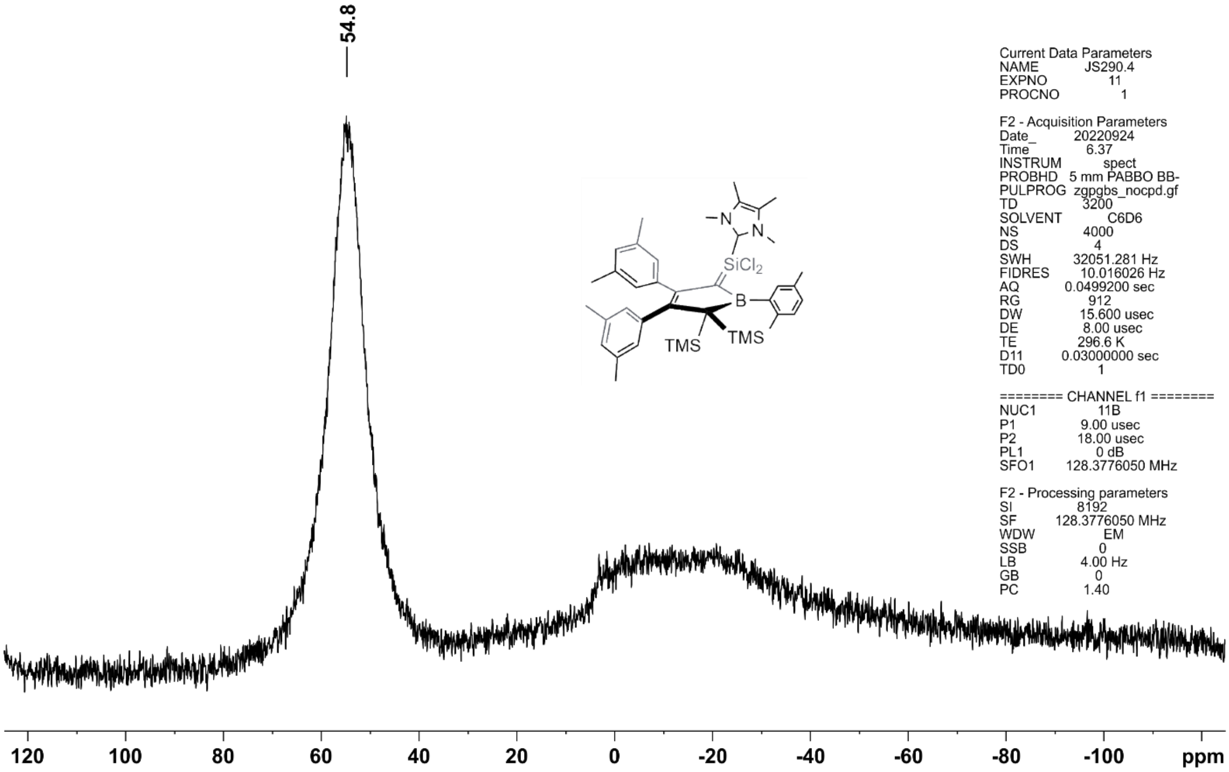

29Si-NMR spectrum of compound **3a-*p*Xyl** in C6D6

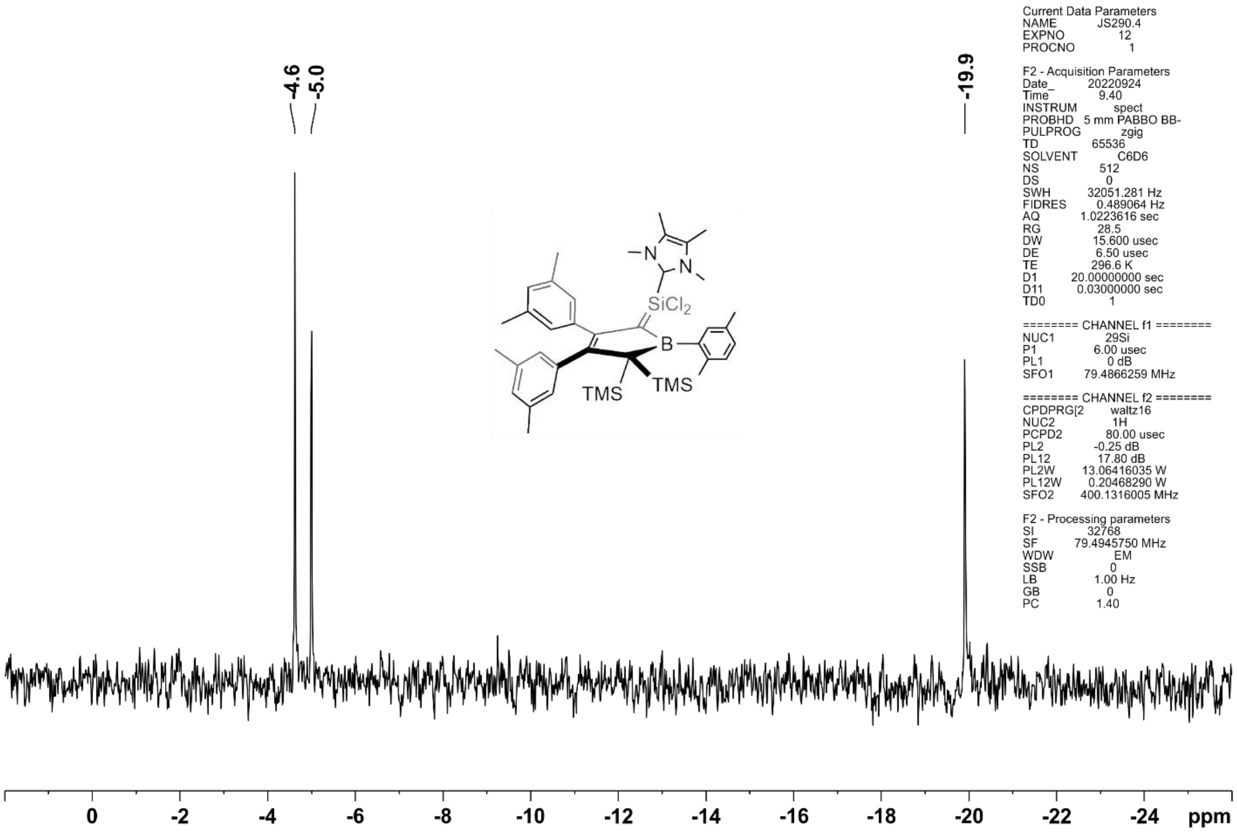

Compound 3b-*p*Xyl

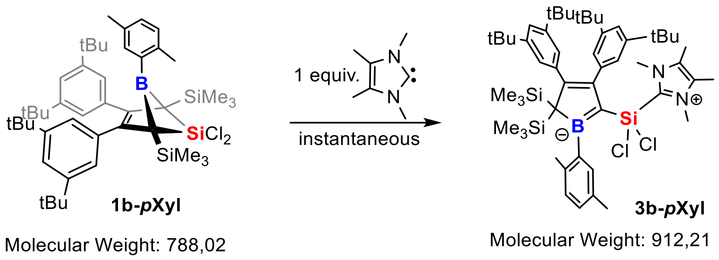

In a glovebox, **1b-*p*Xyl** (102.2 mg, 0.130 mmol, 1 eq) was dissolved in toluene (3 mL), giving a colourless solution. A solution of Me_4NHC (16.1 mg, 0.130 mmol, 1 eq) in toluene (1 mL) was added, whereupon the colour of the reaction mixture changed to yellow. After stirring the reaction mixture for 30 min at ambient temperature, the solvent was removed under reduced pressure to yield a yellow solid. The solid was washed with pentane (4 x 1 mL) and the pale-yellow pentane solution was discarded. The remaining solid was dried under reduced pressure to give compound **3b-*p*Xyl** (98.2 mg, 0.108 mmol, 83 %) as a yellow solid.

Analytical Data for Compound 3b-*p*Xyl

NMR:

^{1}H (400.13 MHz, 296 K, C_6D_6, CD_5H at 7.15 ppm): 7.73–7.55 (br s, 1H, *o-H*$_{ar2/3}$), 7.39 (br s, 2H, *o-H*$_{ar2/3,}$), 7.260 (t, $^{4}J_{HH}$ = 1.8 Hz, 1H, *p-H*$_{ar2/3}$), 7.256 (t, $^{4}J_{HH}$ = 1.8 Hz, 1H, *p-H*$_{ar2/3}$), ca. 7.18 (br s, 1H, *o-H*$_{ar2/3}$, partially superimposed by the solvent signal), 6.86 (d, $^{4}J_{HH}$ = 1.6 Hz, 1H, *o-H*$_{Xyl}$), 6.70 (d, $^{3}J_{HH}$ = 7.7 Hz, 1H, *m-H*$_{Xyl}$), 6.47 (dd, $^{3}J_{HH}$ = 7.7 Hz, $^{4}J_{HH}$ = 1.6 Hz, 1H, *p-H*$_{Xyl}$), 3.02 (s, 6H, N-C*H*$_3$), 2.64 (s, 3H, o-C*H*$_3$), 1.85 (s, 3H, m-C*H*$_3$), 1.57–1.40 (br s, 9H, Ar-C(*Me*)$_3$), 1.40 (s, 18H, Ar-C(*Me*)$_3$), 1.40–1.26 (br s, 9H, Ar-C(*Me*)$_3$), 0.91 (s, 6H, C$_{NHC}$-C*H*$_3$), 0.53 (s, 9H, Si(*Me*)$_3$),0.21–0.58 (br s, 9H, Si(*Me*)$_3$).

^{13}C{^{1}H} (100.62 MHz, 298 K, C_6D_6, solvent signal at 128.0 ppm): 152.5 (C_2)*, 150.3 (*ipso-C*$_{Xyl}$), 148.7 (br, *m-C*$_{Ar2/3}$)*, 147.7 (*m-C*$_{Ar2/3}$), 147.1 (*C*$_{carbene}$), 144.6 (*ipso-C*$_{Ar2/3}$)*, 144.2 (*ipso-C*$_{Ar2/3}$)*, 137.1 (o-*C*$_{Xyl}$-CH$_3$), 133.9 (*o*-*C*H$_{Xyl}$), 132.2 (C_3)*, 130.4 (*m-C*$_{Xyl}$-CH$_3$), 128.9 (o-*C*$_{ar2/3}$), 128.8 (*m*-*C*H$_{Xyl}$), 126.7 (C=*C*$_{NHC}$), 125.2 (br, o-*C*$_{ar2/3}$), 124.7 (*p*-*C*H$_{Xyl}$), 118.3 (*p-C*$_{ar2/3}$), 118.2 (*p-C*$_{ar2/3}$), 101.4 (C_1)*, 51.8 (C_4), 34.9 (Ar-*C*(CH$_3$)$_3$), 34.5 (N$_{NHC}$-*C*H$_3$), 32.1 (Ar-(C(*C*H$_3$)$_3$), 32.0 (Ar-(C(*C*H$_3$)$_3$), 25.1 (*o*-*C*H$_3$), 21.3 (*m*-*C*H$_3$), 7.7 (C$_{NHC}$-*C*H$_3$), 5.1 (Si(*C*H$_3$)$_3$).

*tentative assignments based on similar compounds **2b-Me** and **2b-Cl**

^{11}B (128.38 MHz, 297 K, C_6D_6): 79.0 ($\nu_{1/2}$ ≈ 1100 Hz).

^{29}Si (DEPT-20, 79.49 MHz, 299 K, C_6D_6): –4.5 (TMS), –5.0 (TMS), –20.0 ($SiCl_2$).

Elemental Analysis: $C_{53}H_{81}BN_2Si_3Cl_2$ calcd C 69.78, H 8.95, N 3.07; observed C 69.79, H 9.36, N 3.07.

UV/VIS (toluene): λ_{max} ≈ 420 nm (shoulder)(ε_{420} ≈ 370 L mol^{-1} cm^{-1})

Crystal structure of Compound 3b-*p*Xyl

For further details on the diffraction measurement please see the respective section.

3b-*p*Xyl crystallised from solutions in toluene in a freezer (-40°C).

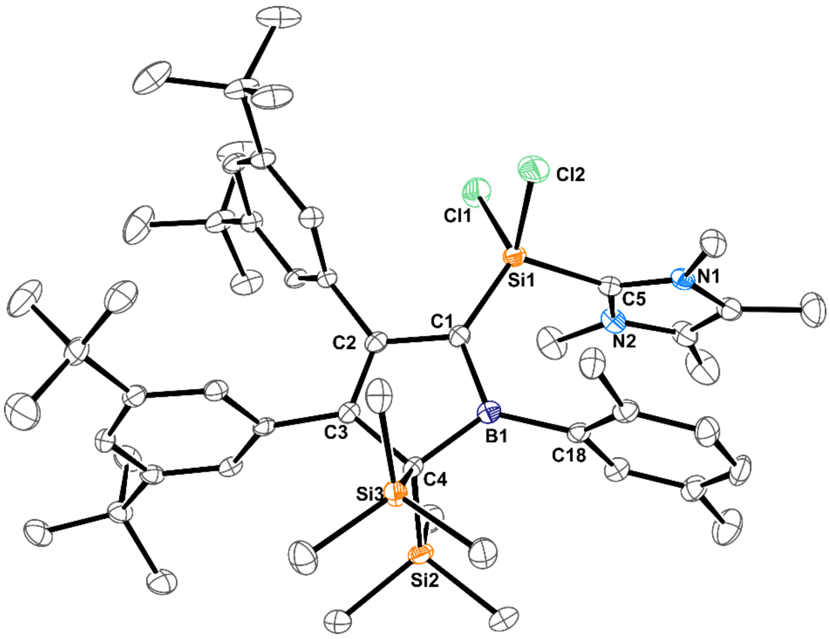

ORTEP plot of the molecular structure of **3b-*p*Xyl**. Atomic displacement parameters are drawn at 50% probability level. Hydrogen atoms are omitted for the sake of clarity. Selected bond length in Å: B1-C1 1.499(3), C1-C2 1.466(3), C2-C3 1.355(3), C3-C4 1.538(3), C4-B1 1.633(3), B1-C18 1.600(3), C1-Si1 1.765(2), Si1-C5 1.908(2), Si1-Cl1 2.0772(8), Si1-Cl2 2.0851(9). The structure was deposited with the CCSD.

Spectra Plots for Compound 3b-*p*Xyl

1H-NMR-spectrum of compound **3b-*p*Xyl** in C6D6

\# C6D5H at 7.15 ppm

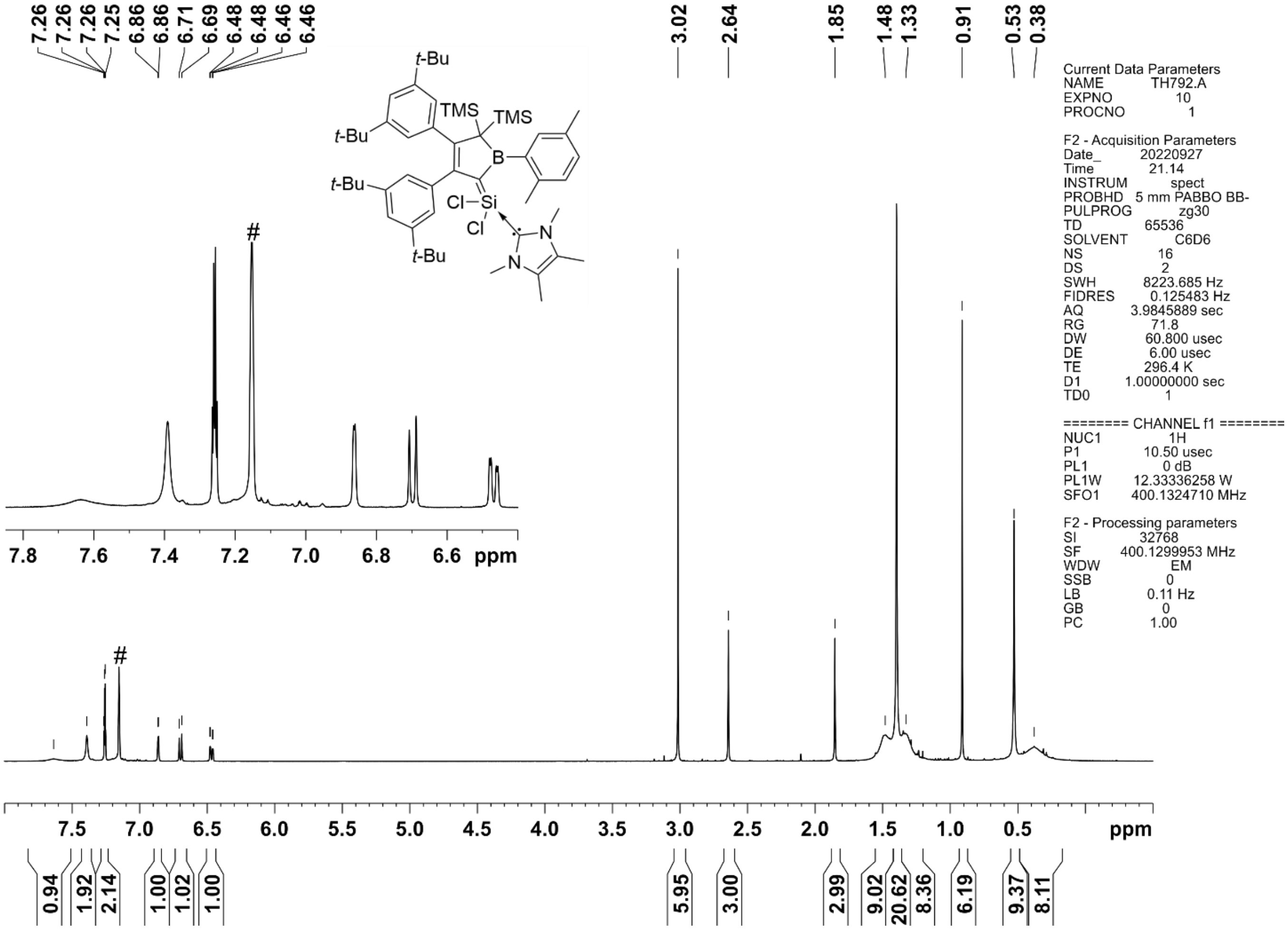

13C{1H}-NMR-spectrum of compound **3b-*p*Xyl** in C6D6

C6D6 at 128.0 ppm

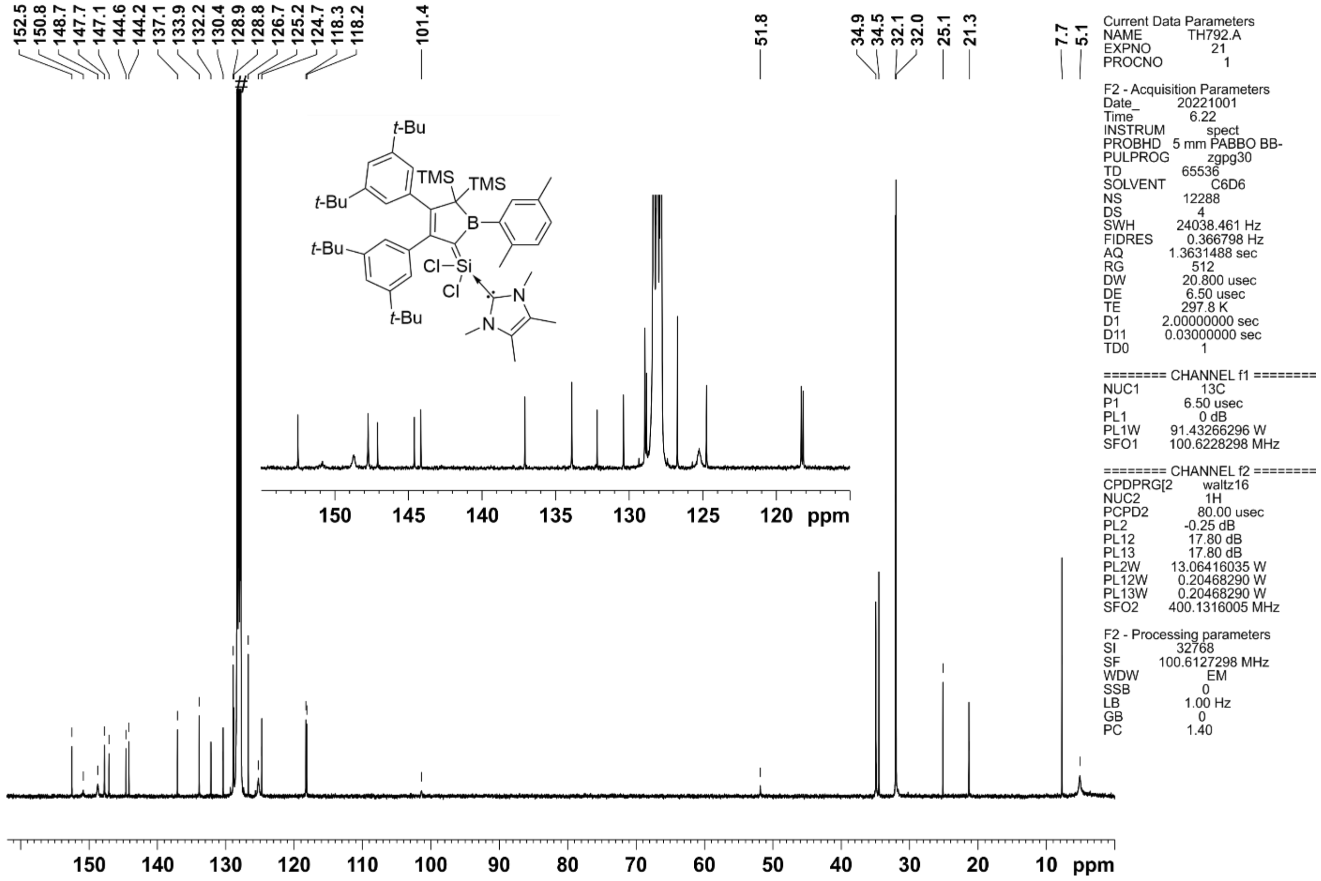

11B-NMR spectrum (background suppressed) of compound **3b-*p*Xyl** in C6D6

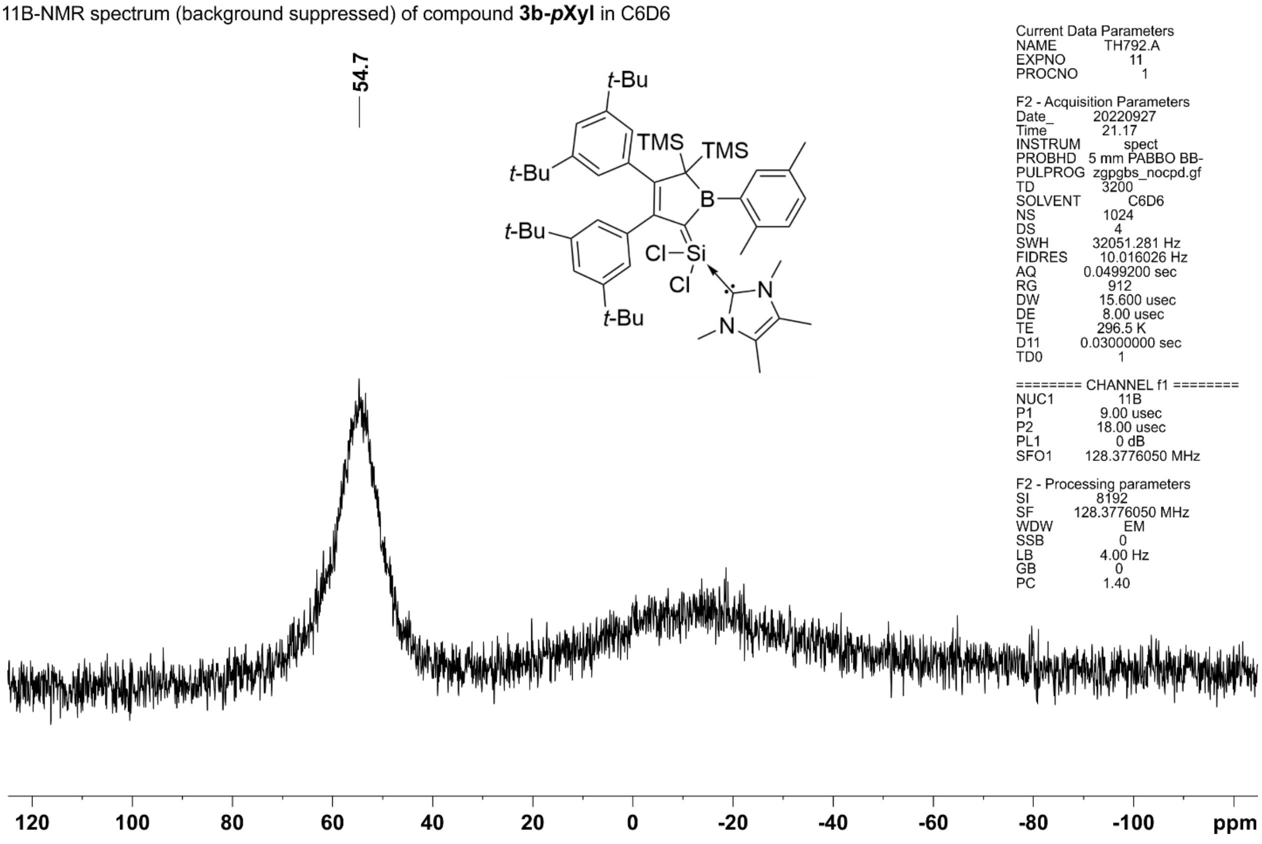

29Si-NMR (inverse gated) spectrum of compound **3b-*p*Xyl** in C6D6

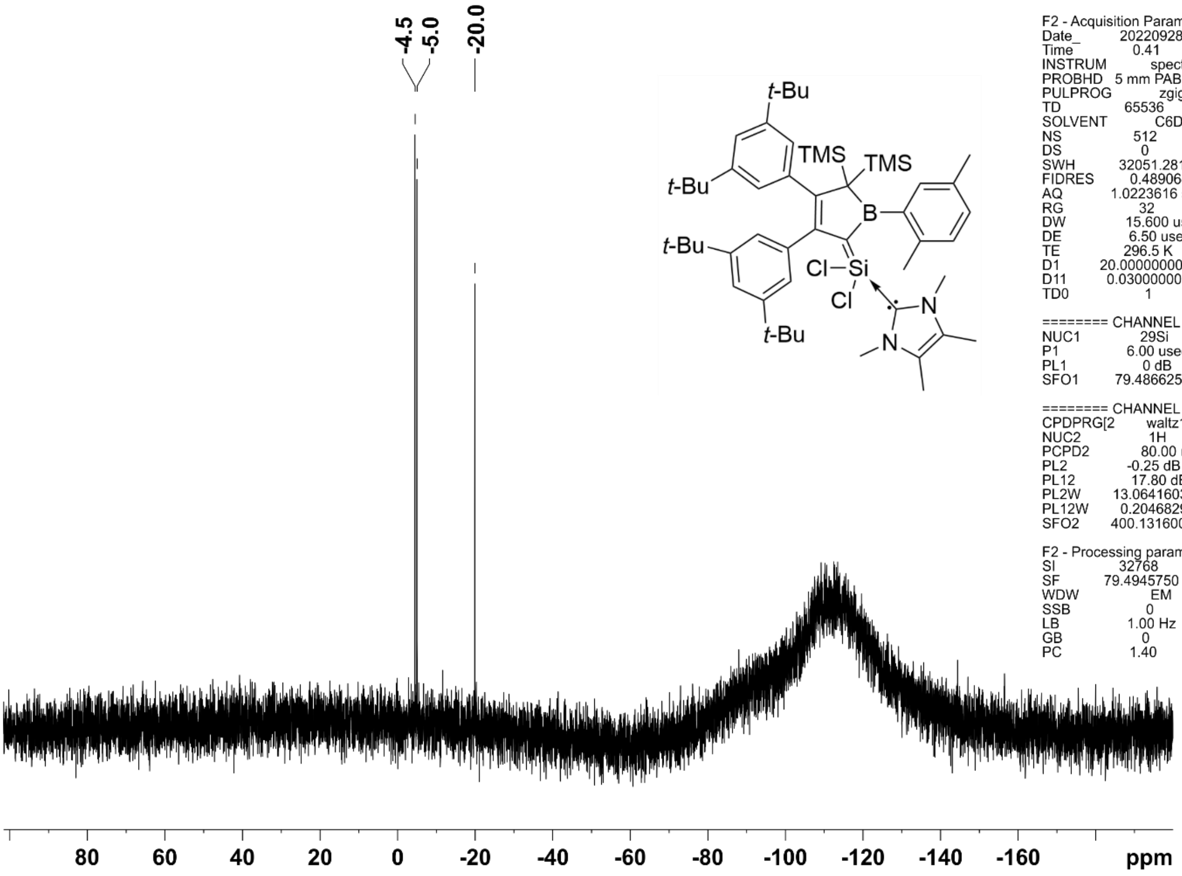

Current Data Parameters
NAME TH792.A
EXPNO 13
PROCNO 1

F2 - Acquisition Parameters
Date_ 20220928
Time 0.41
INSTRUM spect
PROBHD 5 mm PABBO BB-
PULPROG zgig
TD 65536
SOLVENT C6D6
NS 512
DS 0
SWH 32051.281 Hz
FIDRES 0.489064 Hz
AQ 1.0223616 sec
RG 32
DW 15.600 usec
DE 6.50 usec
TE 296.5 K
D1 20.00000000 sec
D11 0.03000000 sec
TD0 1

======== CHANNEL f1 ========
NUC1 29Si
P1 6.00 usec
PL1 0 dB
SFO1 79.4866259 MHz

======== CHANNEL f2 ========
CPDPRG[2 waltz16
NUC2 1H
PCPD2 80.00 usec
PL2 -0.25 dB
PL12 17.80 dB
PL2W 13.06416035 W
PL12W 0.20468290 W
SFO2 400.1316005 MHz

F2 - Processing parameters
SI 32768
SF 79.4945750 MHz
WDW EM
SSB 0
LB 1.00 Hz
GB 0
PC 1.40

Compound 4a-*p*Xyl

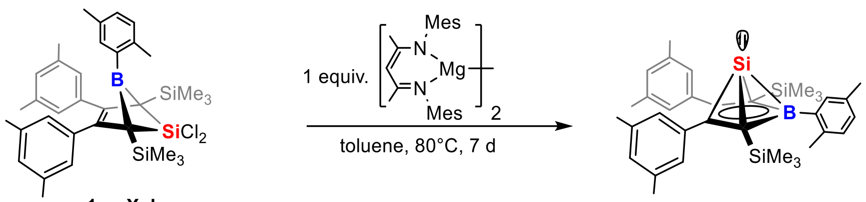

1a-*p*Xyl Molecular Weight: 619,70 → **4a-*p*Xyl** Molecular Weight: 548,80

In a glove box, a Schlenk-tube was charged with the bicyclic compound **1a-*p*Xyl** (303.4 mg, 0.489 mmol, 1 eq.) and the Mg(I) reducing agent (723.1 mg, 1.011 mmol, 1 eq.). Toluene (7 mL) was added and the resulting suspension was left to stir at 80 °C for 7 days. The toluene was removed from the dark red brown suspension to give a red brown residue. Hexane (5 mL) was added and subsequently removed in vacuo to ensure residual toluene to evaporate. The residue was suspended in hexane (6 mL) and filtered through a syringe filter equipped with a pad of glass fiber. The solvent was removed from the filtrate and the residue was thoroughly dried under reduced pressure to give a foamy off-white to brown solid (389.4 mg). This crude product was then crystalized out of pentane which resulted in nearly colorless crystals under a dark brownish oil. The supernatant was removed and the crystals were carefully washed with cold hexane, which resulted in nearly colorless crystals with about 5 % leftover reducing agent (55.7 mg, 0.101 mmol, 21 %).

Note: In an attempt to remove residual reducing agent the crystals were recrystallized from toluene to give colorless needles. The needles were carefully washed with cold pentane and dried then in vacuum (22.0 mg, 0.0399 mmol, 8 %). However this only reduced the residual reducing agent to 3 %.

The product was obtained as a mixture of the two possible conformers in a ratio of ca 85:15.

Analytical Data for Compound 4a-*p*Xyl

NMR:

Note: Two conformation isomers present. Two ppm values separated by a semicolon refer to the same atom in the molecule.

^{1}H (400.13 MHz, 296 K, C_6D_6, C_6D_5H at 7.15 ppm): 7.60; 7.54 (d, 1H, ^{4}J = 1.41 Hz, B-Ar: *o-H*), 7.17 (d, 1H, , ^{3}J = 7.76 Hz, B-Ar: *m-H*), 7.07 (dd, 1H, , ^{3}J = 7.62 Hz, ^{4}J = 1.73 Hz, B-Ar: *p-H*), 7.05; 7.03 (s, 4H, *o-H*), 6.54; 6.52 (s, 2H, *p-H*), 2.71; 2.64 (s, 3H, *o-*C*H*$_3$), 2.35; 2.31 (s, 3H, B-Ar: *m-*C*H*$_3$), 1.96 (s, 12H, , *m-*C*H*$_3$), -0.06 (s, 18H, TMS).

^{13}C{^{1}H} (100.62 MHz, 298 K, C_6D_6 solvent signal at 128.0 ppm): 140.0 (*ipso-C*), 138.3 (C_β), 138.0 (B-Ar: *o-C*-CH$_3$), 137.2; 137.1 (*m-C*-CH$_3$), 136.1; 135.9 (B-Ar: *o-C*H), 133.1 (B-Ar: *m-C*-CH$_3$), 129.8 (*o-C*), 129.6 (*p-C*), 129.1 (B-Ar: *m-C*H), 109.9 (C_α), 26.4; 23.4 (B-Ar: *o-C*H$_3$), 21.3 (B-Ar: *m-C*H$_3$), 21.0 (*m-C*H$_3$), 1.6; 1.4 (TMS), superimposed by solvent signal (B-Ar: *p-C*), only visible in 2D: ca. 142.5 (B-Ar: *ipso-C*).

^{11}B (128.37 MHz, 297 K, C_6D_6): 30.6 ($\omega_{1/2}$ = 316 Hz).

^{29}Si (79.47 MHz, 296 K, C_6D_6): -354.8 (apical Si – minor conformer), -355.6 (^{10}B isotopologue), -355.7 (apical Si-major conformer).

^{29}Si-DEPT20 (79.49 MHz, 297 K, C_6D_6): -6.7 (TMS).

Elemental Analysis: ($C_{34}H_{45}BSi_3$) calcd C 74.41, H 8.27, B 1.97, Si 15.35, observed C 73.56, H 8.40.

Crystal structure of Compound 4a-*p*Xyl

For further details on the diffraction measurement please see the respective section.

4a-*p*Xyl crystallised from solutions in toluene in a freezer (-35°C).

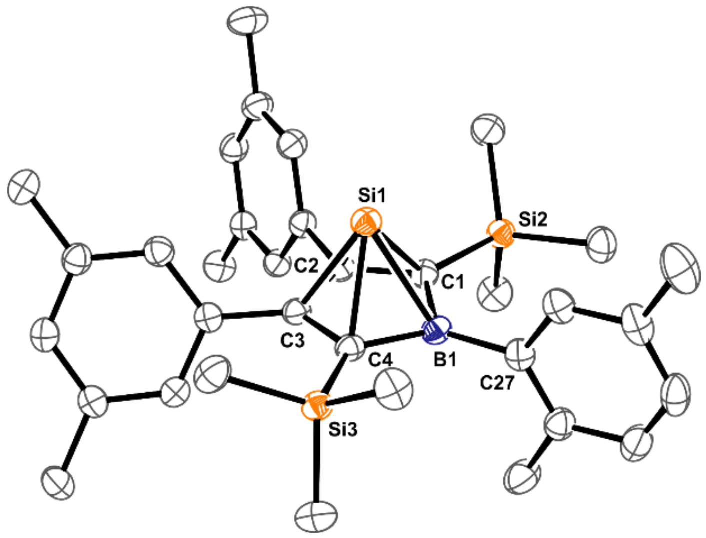

ORTEP of the solid state molecular structure of Si(II) borole halfsandwich compound **4a-*p*Xyl**. H-atoms are omitted for the sake of clarity. Anisotropic displacement parameters are drawn at 50% probability. Selected interatomic distances [Å]: B1–C1 1.549(3), C1–C2 1.462(3), C2–C3 1.429(3), C3–C4 1.460(3), C4–B1 1.547(3), B1–Si1 2.190(2), C1–Si1 2.100(2), C2–Si2 2.094(2), C3–Si1 2.109(2), C4–Si1 2.102(2). $(BC_4\text{-borole})_{centr}$–Si1. 1.700. The structure was deposited with the CCSD.

Spectra Plots for Compound 4a-*p*Xyl

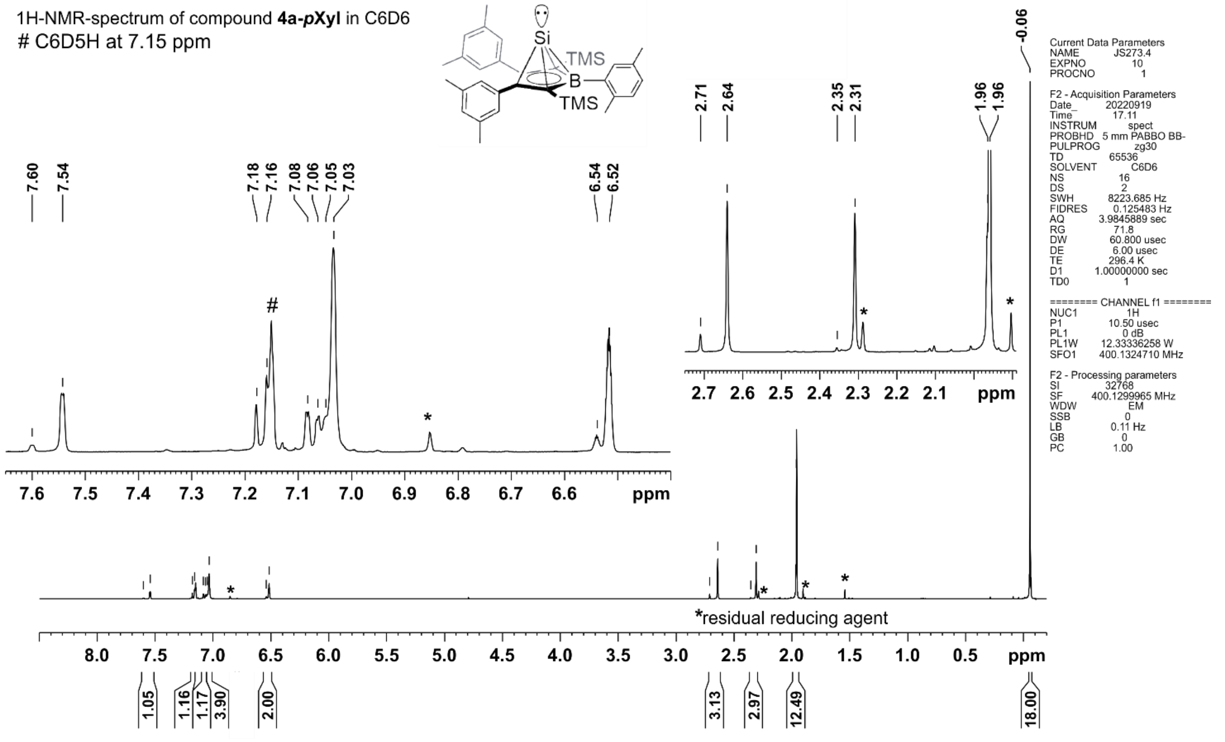

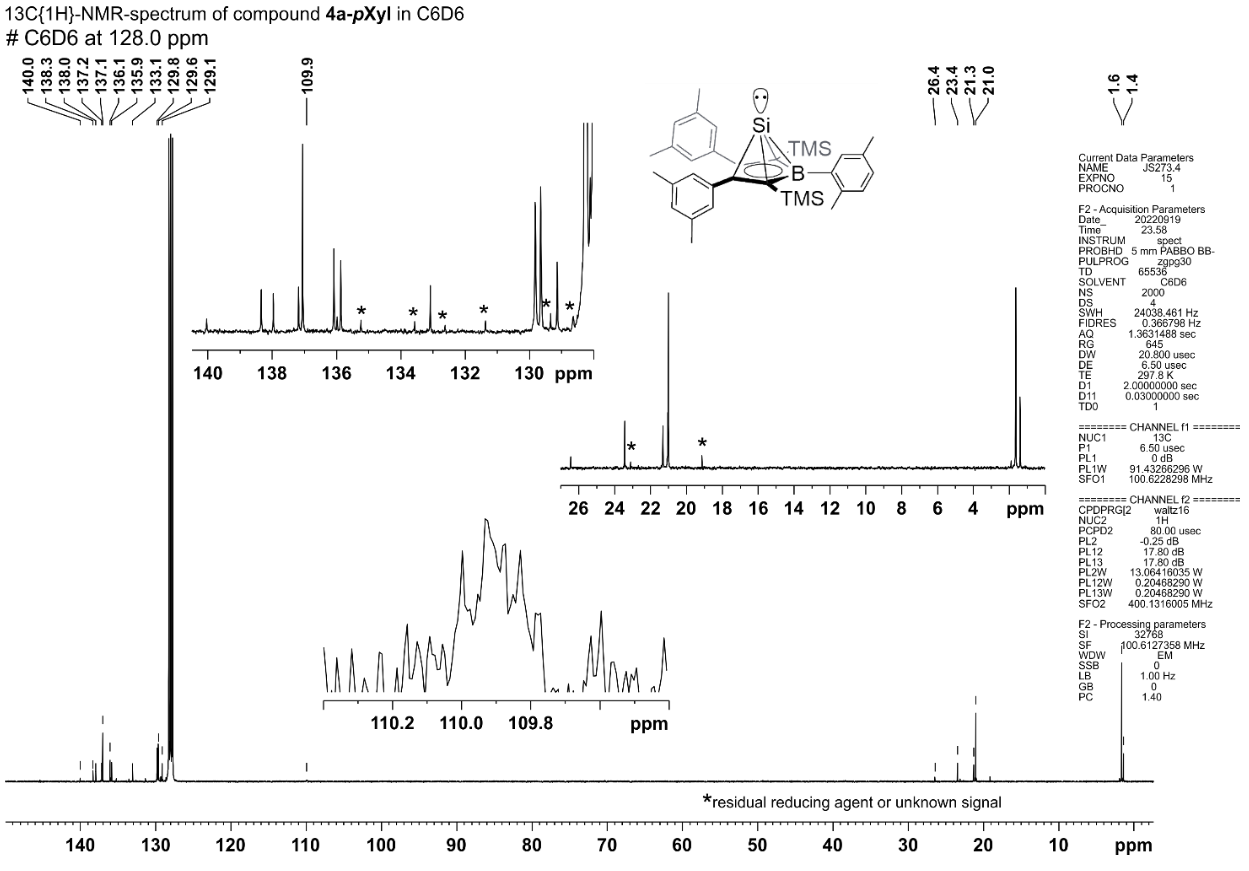

11B-NMR spectrum (background suppressed) of compound **4a-*p*Xyl** in C6D6

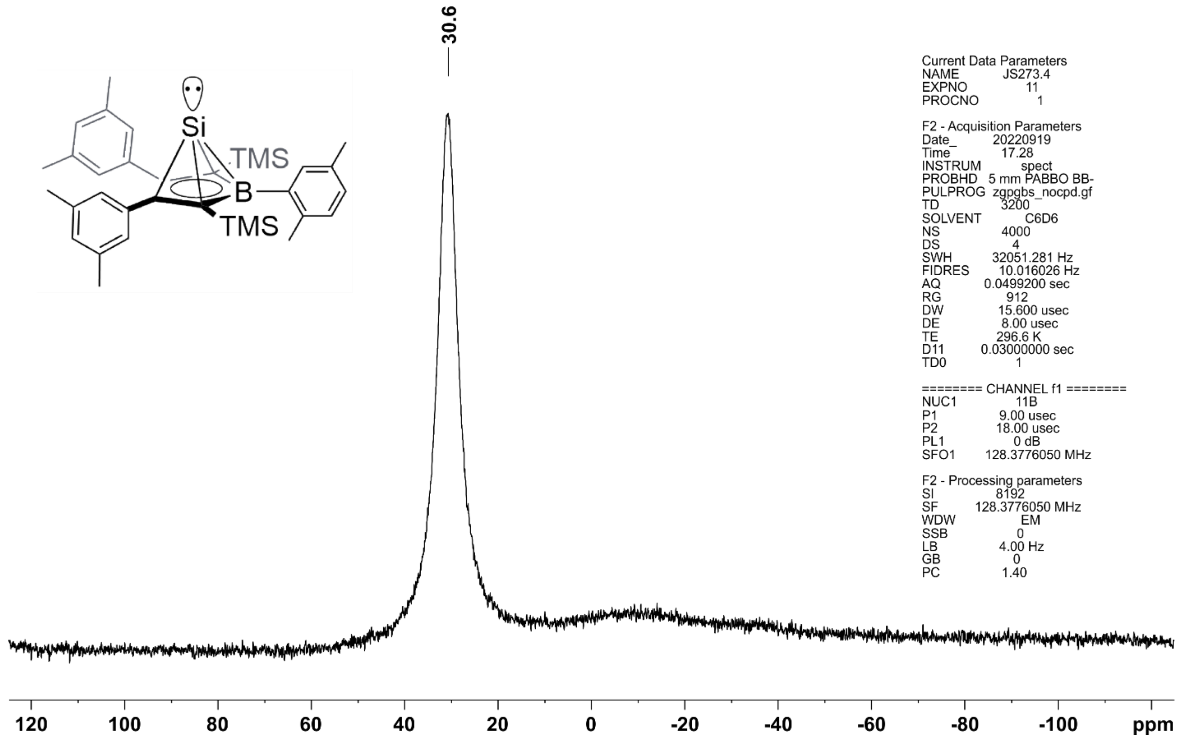

29Si-NMR spectrum of compound **4a-*p*Xyl** in C6D6

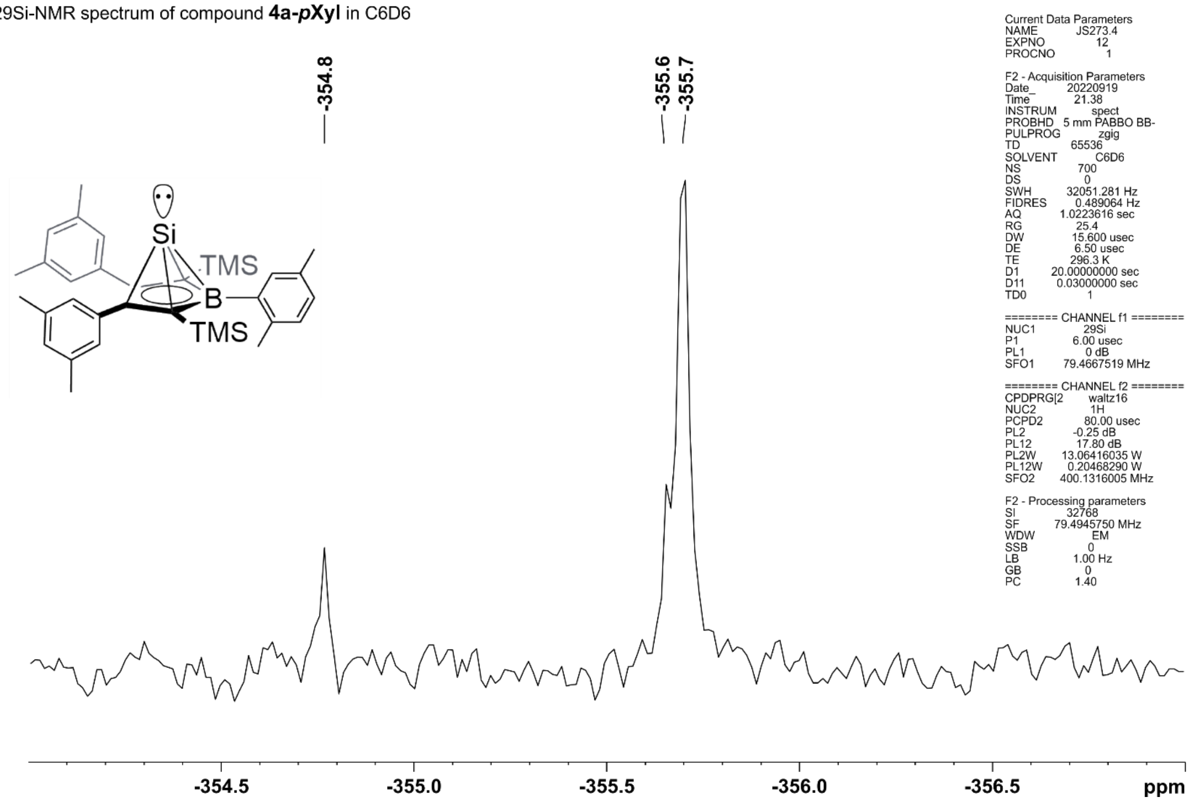

29Si-DEPT20-NMR spectrum of compound **4a-*p*Xyl** in C6D6

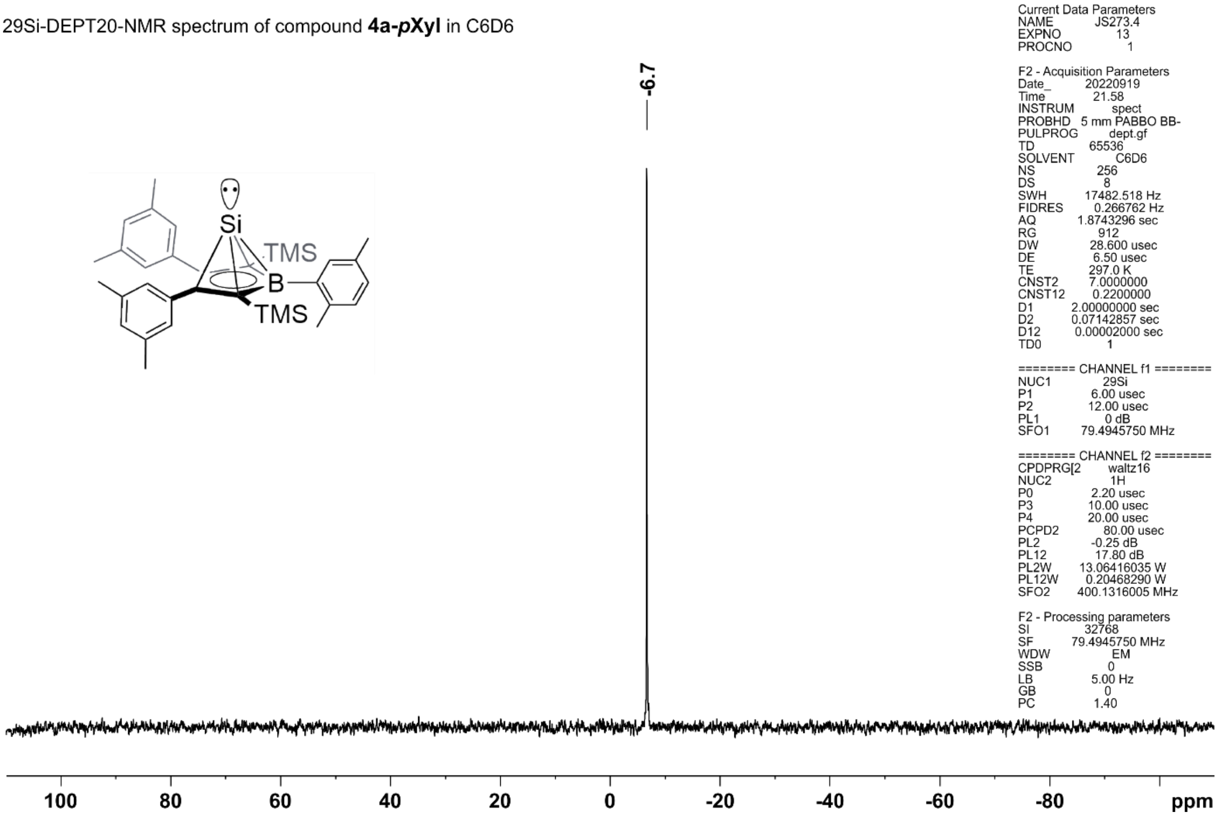

Compound 5b-Me and 4b-Me

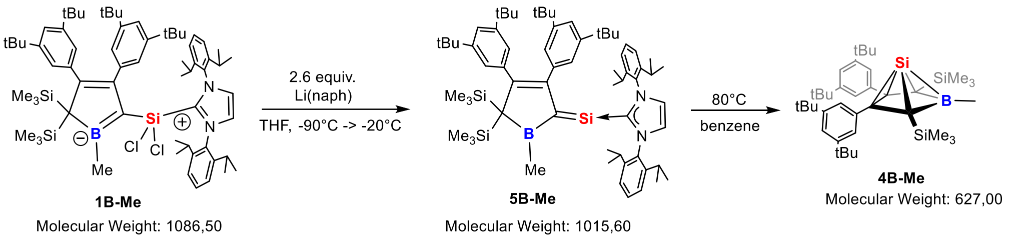

Lithium (2.6 mg, 0.378 mmol, 2.6 eq) and naphthalene (48.5 mg, 0.378 mmol, 2.6 eq) were placed in a Schlenk-flask, suspended in dry THF (2 mL) and stirred overnight to yield a dark green solution. Compound **1b-Me** (158.0 mg, 0.145 mmol, 1 eq) was dissolved in dry THF (4 mL) to give a dark purple solution. Both solutions were afterwards cooled to –90 °C (acetone/liquid nitrogen bath) and at this temperature the solution of lithium naphthalene was carefully added to the solution of compound NHC-supported silylium ylide **1b-Me**. The resulting brownish solution was left to warm to room temperature in the cooling bath. At ca. –60 °C the brownish reaction mixture turned dark red. As the temperature of the cooling bath reached -20 °C, the reaction mixture was taken out of the bath and the solvent was removed under reduced pressure. The obtained red solid was transferred to a glovebox and extracted with pentane (7 x 1 mL). The solvent of the red extract was removed under reduced pressure and the obtained solid was dissolved in diethyl ether (2.3 mL) and stored at –40 °C over three days. Since no crystal formation was observed after this time, the solution was left to stand openly in the glovebox. This way, red crystals of compound NHC-supported silavinylidene **5b-Me** were formed, which were isolated by decanting off the mother liquor and washing the crystals with pentane (3 x 0.2 mL). Afterwards the crystals were dried under reduced pressure to give compound **5b-Me** (69.2 mg, 0.068 mmol, 47 %) as a dark red solid.

Note: If the reaction is performed with higher excess of lithium naphthalenide (3.2 equivalents) under otherwise identical conditions, no formation of compound **5b-Me** is observed. Instead, NMR spectroscopic analysis of the crude reaction mixture indicated the formation of compound **4b-Me** (ca. 65 %) as well as the corresponding lithium-boroledidide Li_2[**B-Me**] (ca. 35 %).
Note: If solutions of isolated **5b-Me** in benzene are kept at 80°C over the course of a few days the clean conversion to **4b-Me** and elimination of free IDipp can be monitored by NMR-spectroscopy.

Analytical Data for Silavinylidene 5b-Me

NMR:

^{1}H (400.13 MHz, 296 K, C_6D_6, CD_5H at 7.15 ppm): 7.16–7.21 (m, 4H, *p*-H_{NHC} + *o*-H_{ar3}), 7.13 (t, $^4J_{HH}$ = 1.8 Hz, 1H, *p*-H_{ar3}), 7.03–7.11 (m, 7H, *p*-H_{ar2} + *o*-H_{ar2} + *m*-H_{NHC}), 6.58 (s, 2H, N-CH_{NHC}), 2.72–3.18 (br, 4H, C*H*(CH_3)$_2$), 1.20–1.45 (br, 12H, CH(CH_3)$_2$, partially overlapped by *t*-Bu signals), 1.28 (s, 18H, Ar_3-C(*Me*)$_3$), 1.22 (s, 18H, Ar_2-C(*Me*)$_3$), 1.07 (s, 3H, B-CH_3), 0.89–1.07 (br, 12H, CH(CH_3)$_2$), 0.24 (s, 18H, Si(*Me*)$_3$).

^{13}C{^{1}H} (100.62 MHz, 298 K, C_6D_6, solvent signal at 128.0 ppm): 181.1 (C_{NHC}), 180.8 (C_1), 157.3 (C_2), 148.5 (*m*-C_{Ar3}), 148.1 (*m*-C_{Ar2}), 145.8 (br s, *o*-C_{NHC}), 144.7 (*ipso*-$C_{ar2/3}$), 143.4 (*ipso*-$C_{ar2/3}$), 137.1 (C_3), 134.2 (*ipso*-C_{NHC}), 131.0 (*p*-C_{NHC}), 127.0 (*o*-C_{ar2}), 126.8 (*o*-C_{ar3}), 125.0 (br s, *m*-C_{NHC}), 124.5 (N-CH_{NHC}), 117.84 (*p*-$C_{ar2/3}$), 117.81 (*p*-$C_{ar2/3}$), 48.2 (C_4), 34.9 (Ar_3-*C*(CH_3)$_3$), 34.6 (Ar_2-*C*(CH_3)$_3$), 31.94 (Ar_2-(C(*C*H_3)$_3$), 31.88 (Ar_3-(C(*C*H_3)$_3$), 29.3 (br s, *C*H(CH_3)$_2$), 26.0 (br s, CH(*C*H_3)$_2$), 22.8 (br s, CH(*C*H_3)$_2$), 10.3 (B-*C*H_3), 3.0 (Si(*C*H_3)$_3$).

^{11}B (128.38 MHz, 297 K, C_6D_6): 61.3 ($\nu_{1/2}$ ≈ 1700 Hz).

^{29}Si (inverse gated, 79.49 MHz, 296 K, C_6D_6): 226.3 (Si), –4.8 (TMS).

Elemental Analysis: $C_{66}H_{99}BN_2Si_3$ calcd C 78.06, H 9.83, N 2.76; observed C 77.45, H 9.83, N 2.76.

UV/VIS (toluene): λ_{max} = 496 nm (ε_{496} ≈ 7200 L mol^{-1} cm $^{-1}$).

Analytical Data for half-sandwich cluster 4b-Me

NMR:

^{1}H (400.13 MHz, 297 K, C_6D_6, CD_5H at 7.15 ppm): 7.24 (t, $^{4}J_{HH}$ = 1.8 Hz, 2H, *p-H$_{ar}$*), 7.12–7.16 (br, partially overlapped by the solvent signal, 4H, o-*H$_{ar}$*), 1.17 (s, 36H, Ar-C(*Me*)$_3$), 1.08 (s, 3H, B-C*H$_3$*), 0.16 (s, 18H, Si(*Me*)$_3$).

^{13}C{^{1}H} (100.62 MHz, 298 K, C_6D_6, solvent signal at 128.0 ppm): 150.0 (*m-C$_{Ar}$*), 140.5 (borole-*C$_{3,4}$*), 135.8 (*ipso-C$_{ar}$*), 126.3 (*o-C$_{ar}$*), 120.9 (*p-C$_{ar}$*), 107.0 (borole-*C$_{2,5}$*), 34.7 (Ar-(*C*(CH$_3$)$_3$), 31.5 (Ar-(C(*C*H$_3$)$_3$), 2.1 (Si(*C*H$_3$)$_3$), –1.1 (B-*C*H$_3$).

^{11}B (128.38 MHz, 297 K, C_6D_6): 31.4 ($\nu_{1/2}$ ≈ 700 Hz).

^{29}Si (inverse gated, 79.48 MHz, 297 K, C_6D_6): –7.1 (TMS), –347.63 (borole-*Si*, additional signal at -347.59 for ^{10}B isotopologue).

Crystal structure of Compound 5b-Me

For further details on the diffraction measurement please see the respective section.

5b-Me crystallised from solutions in ether upon slow evaporation.

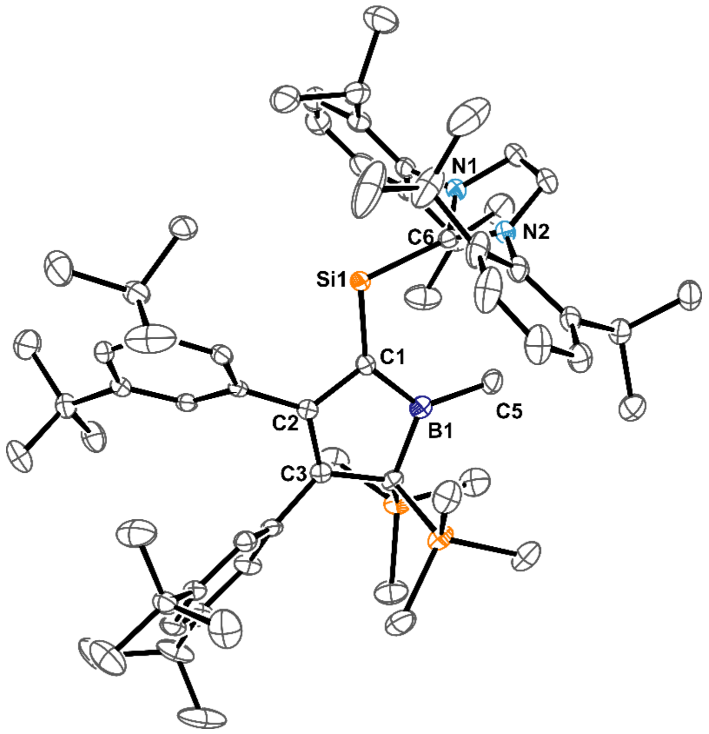

ORTEP of the solid state molecular structure of NHC-supported silavinylidene **5b-Me**. H-atoms and a partially occupied ether molecule are omitted for the sake of clarity. Anisotropic displacement parameters are drawn at 50% probability. Selcted bond length in Å: B1–C1 1.536(3), C1–C2 1.477(3), C2–C3 1.361(3), C3–C4 1.539(3), C4–B1 1.603(3), B1–C5 1.580(4), C1–Si1 1.783(2), Si1–C6 1.973(3). The structure was deposited with the CCSD.

Spectra Plots for Compound 5b-Me

1H-NMR-spectrum of compound **5b-Me** in C6D6
C6D5H at 7.15 ppm

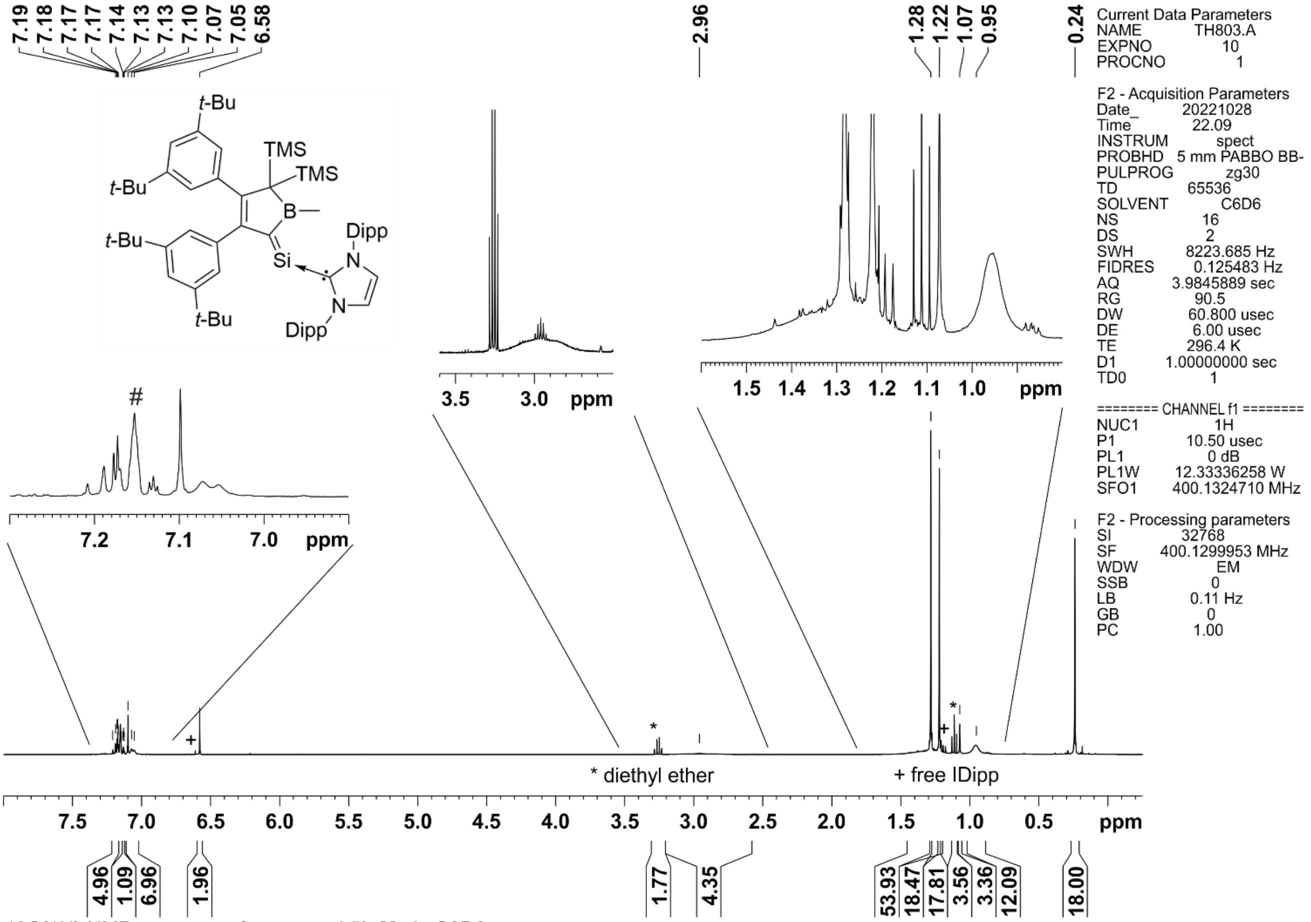

13C{1H}-NMR-spectrum of compound **5b-Me** in C6D6
C6D6 at 128.0 ppm

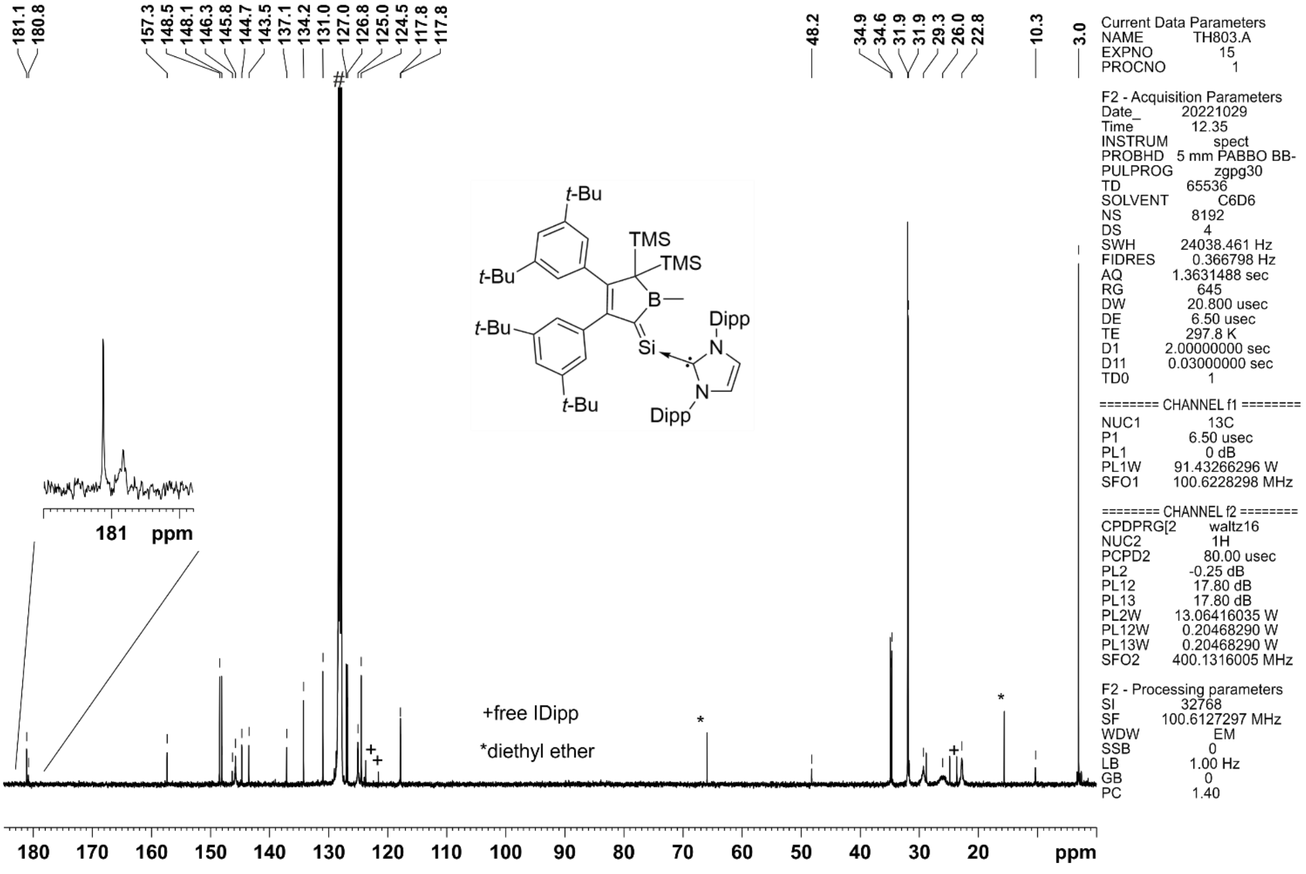

11B-NMR spectrum (background suppressed) of compound **5b-Me** in C6D6

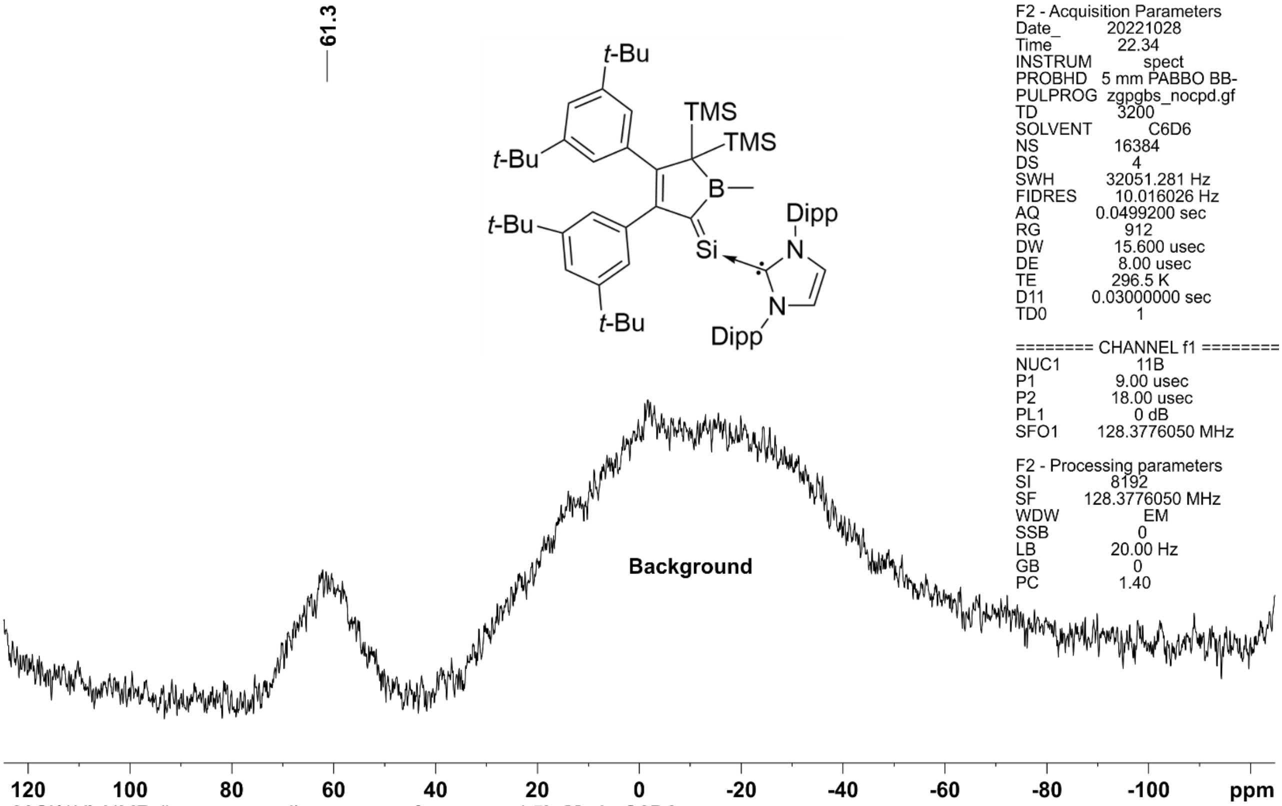

Current Data Parameters
NAME TH803.A
EXPNO 11
PROCNO 1

F2 - Acquisition Parameters
Date_ 20221028
Time 22.34
INSTRUM spect
PROBHD 5 mm PABBO BB-
PULPROG zgpgbs_nocpd.gf
TD 3200
SOLVENT C6D6
NS 16384
DS 4
SWH 32051.281 Hz
FIDRES 10.016026 Hz
AQ 0.0499200 sec
RG 912
DW 15.600 usec
DE 8.00 usec
TE 296.5 K
D11 0.03000000 sec
TD0 1

======== CHANNEL f1 ========
NUC1 11B
P1 9.00 usec
P2 18.00 usec
PL1 0 dB
SFO1 128.3776050 MHz

F2 - Processing parameters
SI 8192
SF 128.3776050 MHz
WDW EM
SSB 0
LB 20.00 Hz
GB 0
PC 1.40

29Si{1H}-NMR (inverse gated) spectrum of compound **5b-Me** in C6D6

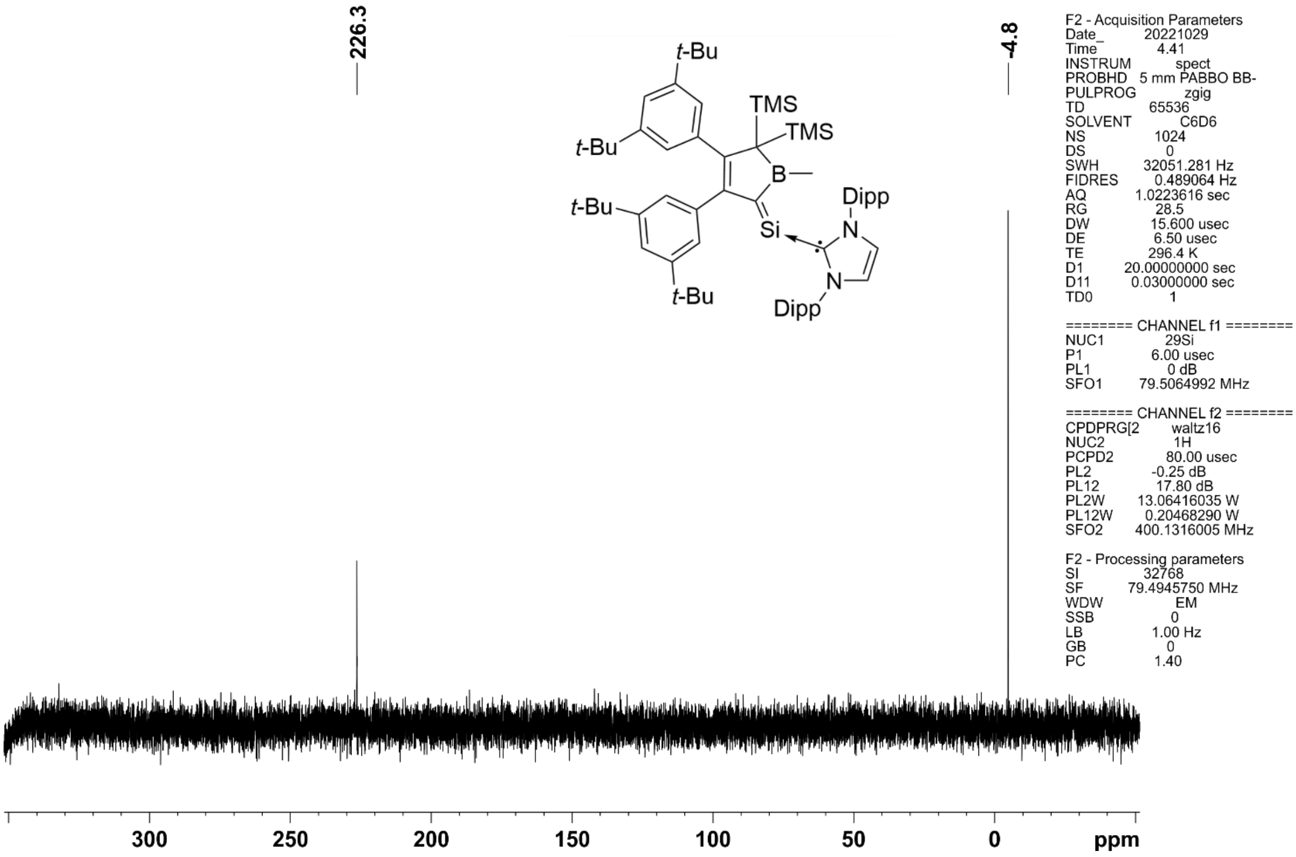

Current Data Parameters
NAME TH803.A
EXPNO 13
PROCNO 1

F2 - Acquisition Parameters
Date_ 20221029
Time 4.41
INSTRUM spect
PROBHD 5 mm PABBO BB-
PULPROG zgig
TD 65536
SOLVENT C6D6
NS 1024
DS 0
SWH 32051.281 Hz
FIDRES 0.489064 Hz
AQ 1.0223616 sec
RG 28.5
DW 15.600 usec
DE 6.50 usec
TE 296.4 K
D1 20.00000000 sec
D11 0.03000000 sec
TD0 1

======== CHANNEL f1 ========
NUC1 29Si
P1 6.00 usec
PL1 0 dB
SFO1 79.5064992 MHz

======== CHANNEL f2 ========
CPDPRG[2 waltz16
NUC2 1H
PCPD2 80.00 usec
PL2 -0.25 dB
PL12 17.80 dB
PL2W 13.06416035 W
PL12W 0.20468290 W
SFO2 400.1316005 MHz

F2 - Processing parameters
SI 32768
SF 79.4945750 MHz
WDW EM
SSB 0
LB 1.00 Hz
GB 0
PC 1.40

Spectra Plots for Compound 4b-Me

1H-NMR-spectrum of compound 4b-Me in C6D6
C6D5H at 7.15 ppm

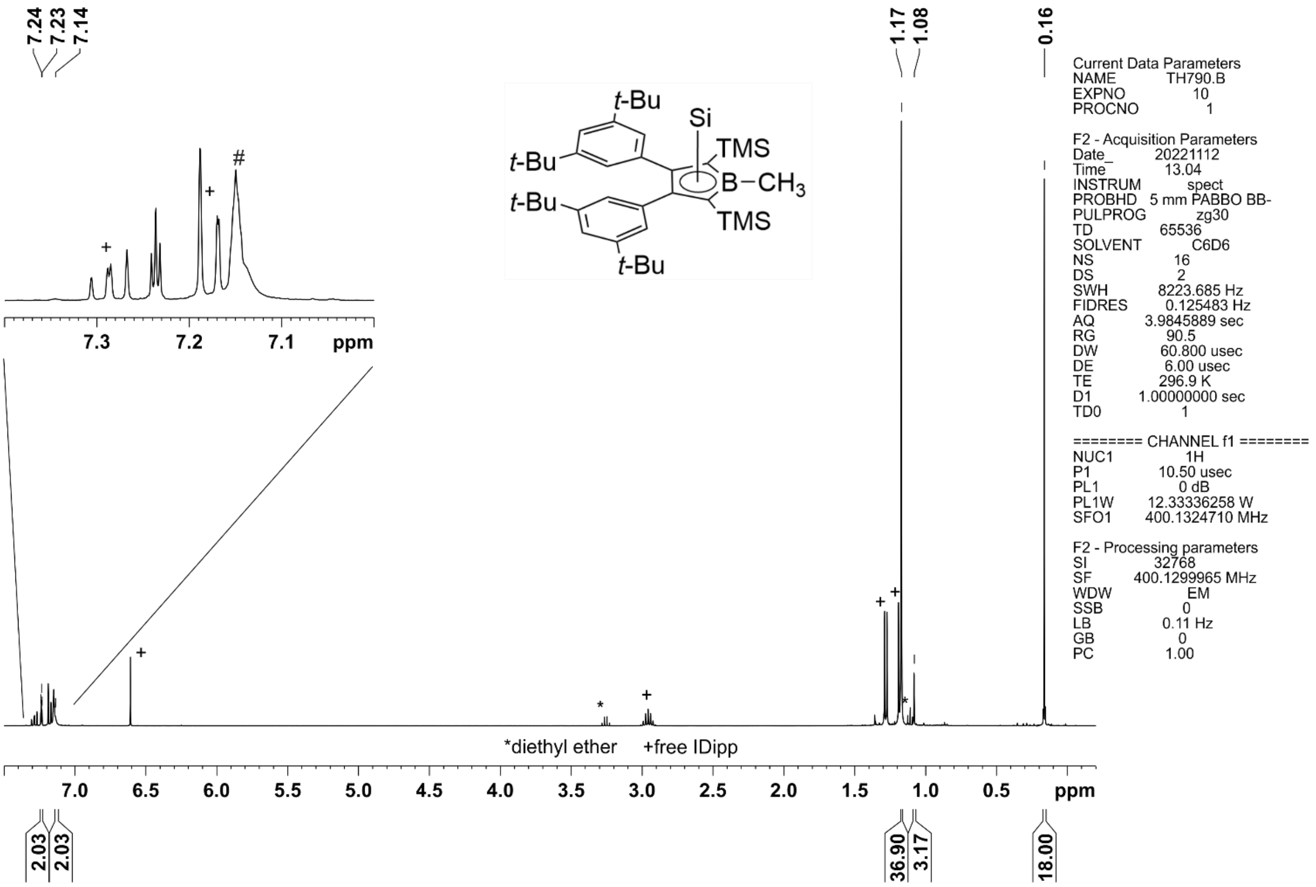

13C{1H}-NMR-spectrum of compound 4b-Me (containing some free IDipp) in C6D6
C6D6 at 128.0 ppm

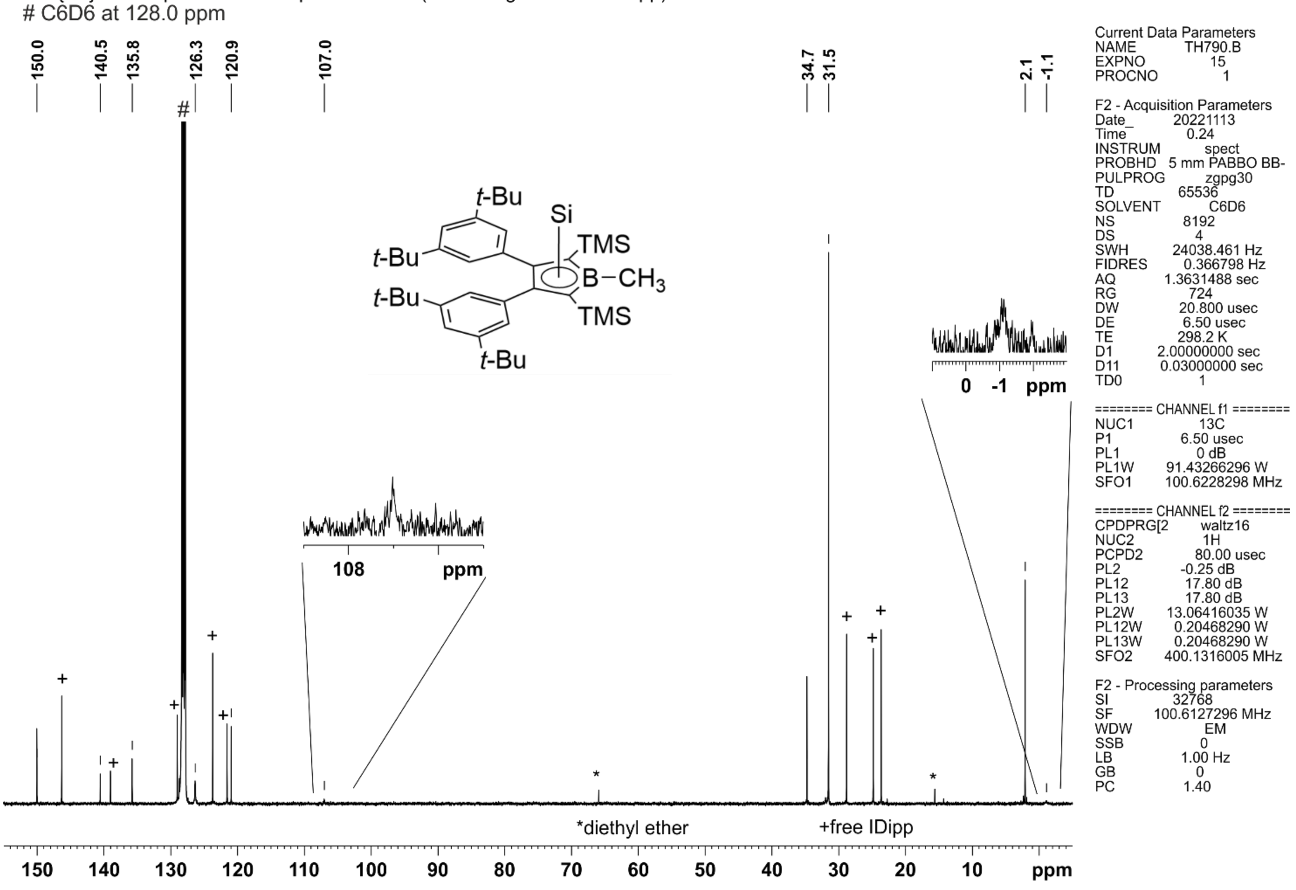

11B-NMR spectrum (background suppressed) of compound **4b-Me** in C6D6

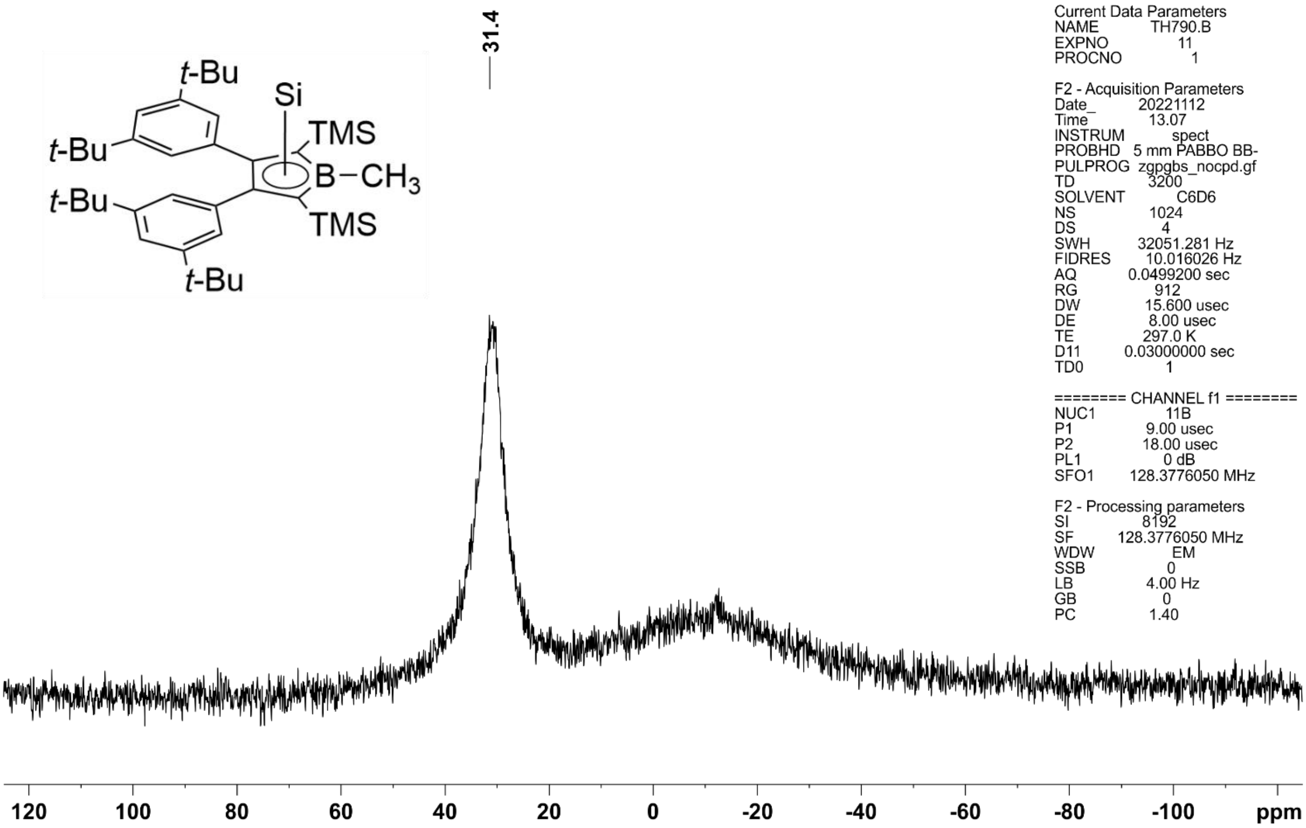

29Si{1H}-NMR (inverse gated) spectrum of compound **4b-Me** in C6D6

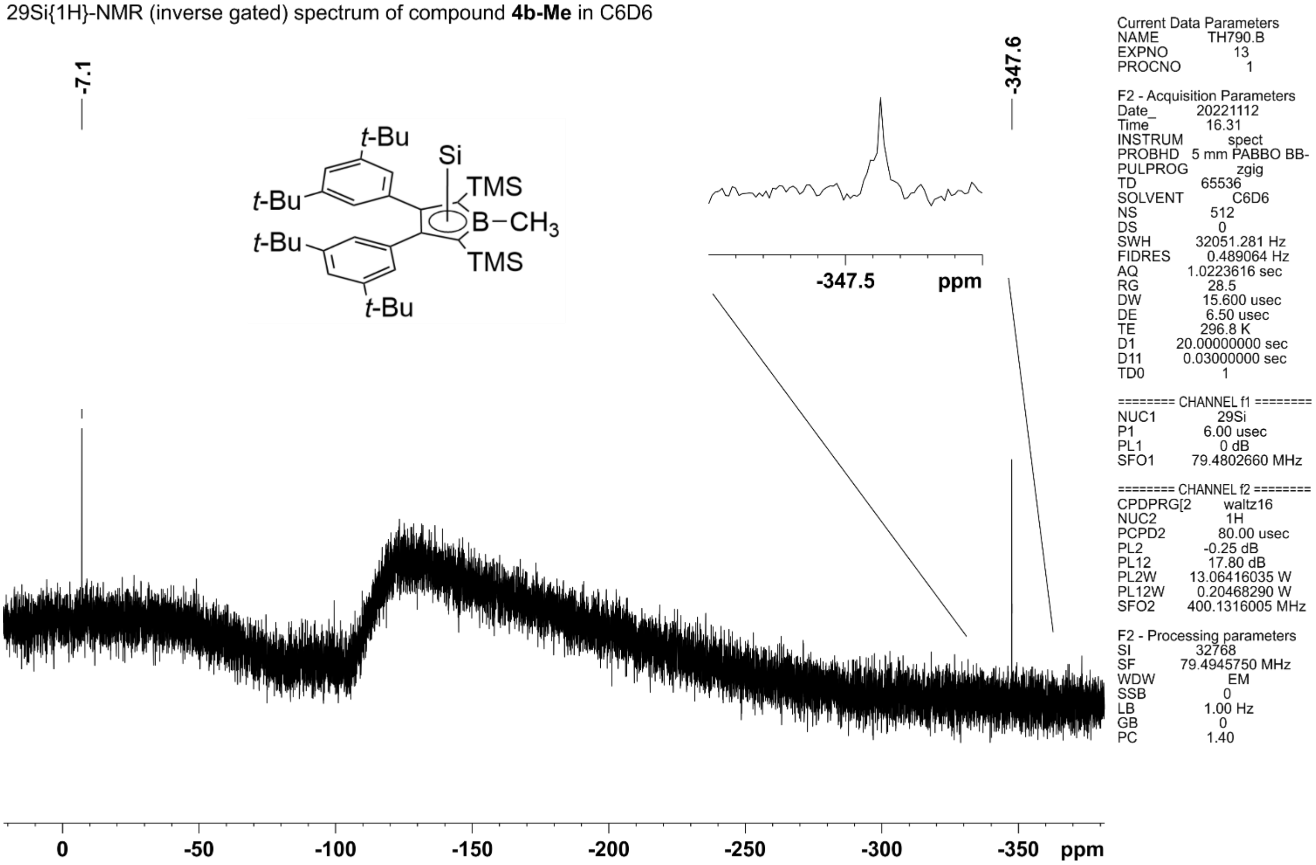

Compound 4b-Ph*

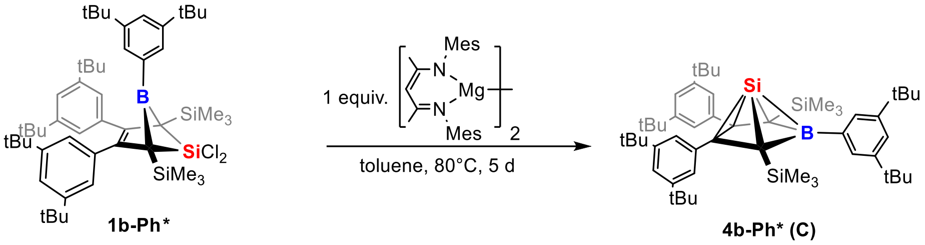

1b-Ph*
Molecular Weight: 872,19

4b-Ph* (C)
Molecular Weight: 801,29

Compound **1b-Ph*** (purity ca. 90 %, 138.2 mg, 0.158 mmol, 1 eq) was dissolved in toluene (5 mL) and Mg(I)-dimer (113.4 mg, 0.158 mmol, 1 eq) was added. The resulting pale yellow suspension was heated to 80 °C in a sealed Schlenk flask. After five days, the solvent was removed under reduced pressure and the Schlenk flask was transferred to a glove box. The yellowish solid was extracted with *n*-hexane (3 x 1 mL) the solvent of the red-yellow extract was removed under reduced pressure. The obtained solid (168 mg) was once again dissolved in *n*-hexane (0.5 mL) and filtered through a syringe filter equipped with a thin plug of glass fiber. The filter was washed with *n*-hexane (2 x 0.1 mL) and the volume of the filtrate solution was reduced to approximately 0.3 mL and stored at –40 °C overnight. The resulting colourless crystals were isolated, washed with cold *n*-hexane (–40 °C, 3 x 0.1 mL) and dried under reduced pressure. After a second crystallisation step, compound **4b-Ph*** (20.8 mg, 0.026 mmol, 16 %) was isolated as a colourless solid.

The spectroscopic data match those of the previously reported compound **4b-Ph***, which was synthesized via a different synthetic route. (T. Heitkemper, J. Sarcevic, C. P. Sindlinger, *J. Am. Chem. Soc.* **2020**, *142*, 21304-21309)

Note: In general, the reaction proceeds selectively to give compound **4b-Ph*** without the formation of any observable side products. However, as the starting material only showed a purity of ca. 90 %, the formed crystalline solid had to be thoroughly washed with cold *n*-hexane and therefore the yield of the isolated product is very low.

UV*vis* Spectra Plots

Free Boroles A-Me, A-Xyl, A-*p*Xyl, B-Xyl, B-*p*Xyl

Normalized for the respective λ_{max} for comparison. In hexane solution.

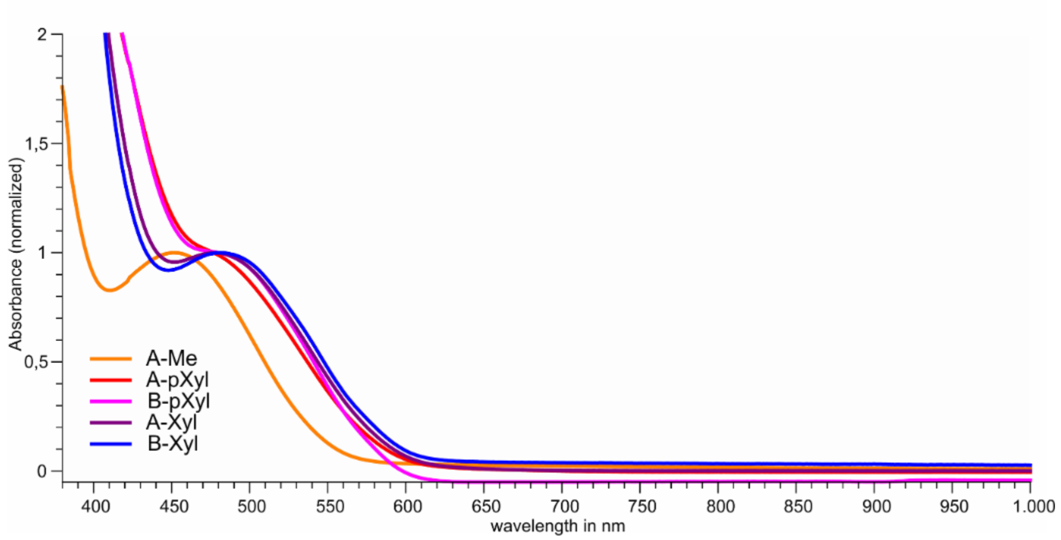

NHC-supported Silylium Ylides Compounds 2

Normalized for the respective λ_{max} for comparison. In hexane or toluene solution.

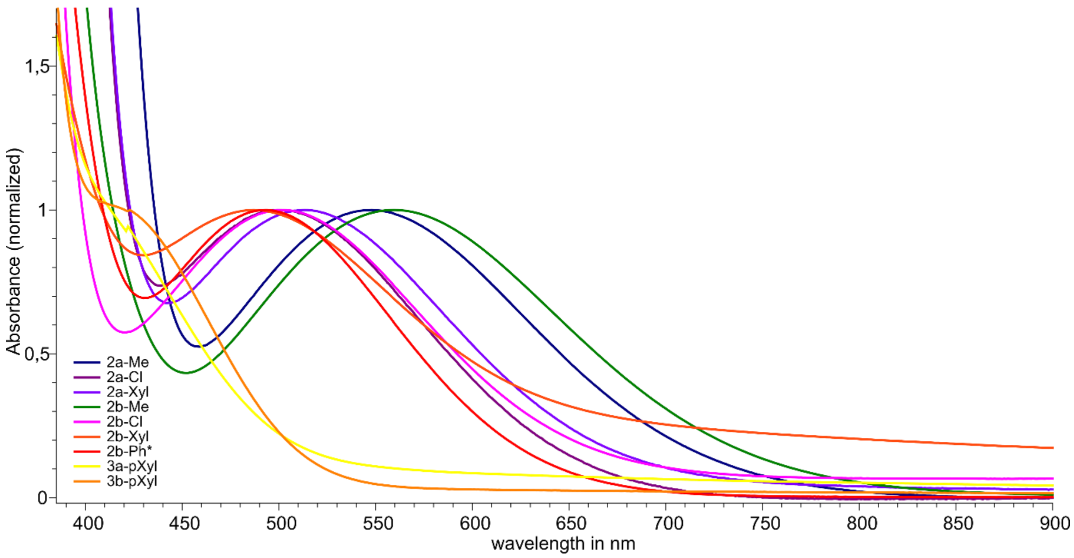

NHC-supported Silavinylidene 5b-Me

Normalized for the respective λ_{max} for comparison. In toluene solution.

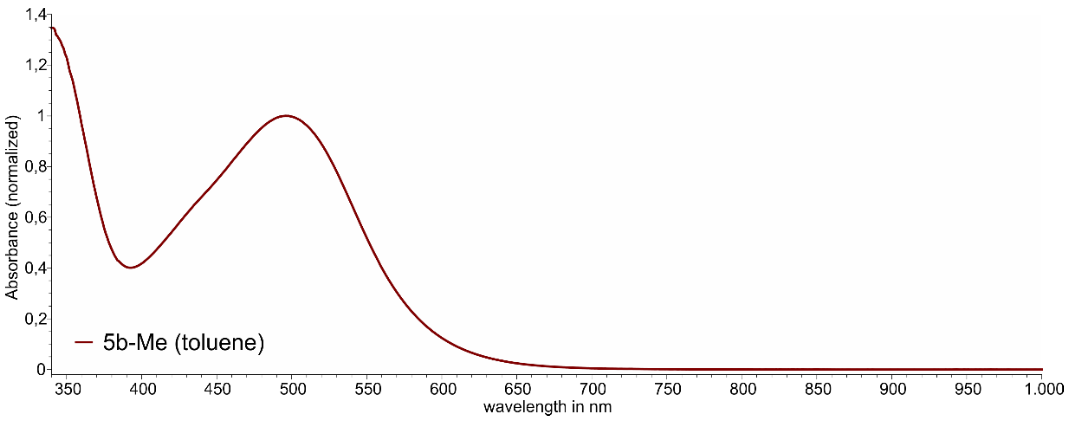

Crystallographic Details

General Data Acquisition and Processing

X-ray data were collected on Bruker APEX II CCD diffractometers or a Bruker APEX CCD diffractometer with Mo Kα radiation. The data were integrated using SAINT implemented in Bruker's APEX3 programme suite.[11] SADABS was used for multi-scan absorption correction.[12] Structure solution was performed with SHELXT[13] and refined using SHELXL[14] along the graphical user interphase of ShelXle by full matrix least squares calculations of F^2.[15] In some cases DSR has been applied to treat disordered solvent molecules.[16] All hydrogen atoms were placed using a riding model with regards to the respective carbon atom. Further details on the individual data sets are tabulated in the analytical section of each compound. CIF-reports were generated using Kratzert's FinalCif programme.

Tabulated Crystallographic Details

	**A-Me**	**A-Xyl**	**B-Xyl**	**1A-*p*Xyl**	**1B-Ph***	**2A-Me**	**2A-Cl**	**2A-Xyl**	**2B-Ph***	**3B-*p*Xyl**	**4A-*p*Xyl**	**5B-Me**
CCDC number	2233795	2233796	2233797	2233798	2233799	2233800	2233801	2233802	2233803	2233804	2233805	2239382
Empirical formula	$C_{27}H_{39}BSi_2$	$C_{34}H_{45}BSi_2$	$C_{104}H_{166}B_2Si_4$	$C_{34}H_{45}BCl_2Si_3$	$C_{52}H_{81}BCl_2Si_3$	$C_{54}H_{75}BCl_2N_2Si_3$	$C_{53}H_{72}BCl_3N_2Si_3$	$C_{61}H_{81}BCl_2N_2Si_3$	$C_{79}H_{117}BCl_2N_2Si_3$	$C_{53}H_{81}BCl_2N_2Si_3$	$C_{34}H_{45}BSi_3$	$C_{66}H_{99}BN_2Si_3\times0.25(OC_4H_{10})$
Formula weight	430.57	520.69	1550.34	619.68	872.14	918.14	938.55	1008.25	1260.72	912.17	548.78	1034.08
Temperature [K]	100(2)	100(2)	100(2)	100(2)	100(2)	100(2)	100(2)	100(2)	100(2)	100(2)	100(2)	100(2)
Crystal system	orthorhombic	triclinic	triclinic	monoclinic	triclinic	monoclinic	monoclinic	orthorhombic	monoclinic	monoclinic	monoclinic	monoclinic
Space group (number)	$Pbca$ (61)	$P\overline{1}$ (2)	$P\overline{1}$ (2)	$P2_1/c$ (14)	$P\overline{1}$ (2)	$P2_1/n$ (14)	$P2_1/n$ (14)	$P2_12_12_1$ (19)	$P2_1/c$ (14)	$P2_1/n$ (14)	$P2_1/c$ (14)	$P2_1/n$ (14)
a [Å]	20.340(5)	8.7268(4)	11.975(2)	23.592(3)	12.085(2)	17.1027(9)	17.1220(14)	15.9046(13)	10.726(3)	9.8784(9)	11.9105(7)	14.8214(11)
b [Å]	11.791(3)	11.9752(5)	14.700(3)	15.0926(17)	21.926(4)	15.8004(8)	15.7803(13)	16.4022(14)	30.190(9)	21.109(2)	20.2221(9)	26.3082(18)
c [Å]	22.744(6)	16.8842(8)	14.916(3)	22.907(3)	22.008(4)	20.5088(11)	20.5144(17)	23.053(2)	24.137(8)	26.089(2)	14.3115(7)	17.7522(13)
α [°]	90	72.238(2)	90.820(3)	90	76.903(3)	90	90	90	90	90	90	90
β [°]	90	79.568(2)	103.725(3)	118.969(2)	74.670(3)	91.840(3)	91.6690(10)	90	98.286(5)	93.790(2)	103.638(4)	110.1790(10)
γ [°]	90	73.411(2)	90.717(3)	90	83.086(3)	90	90	90	90	90	90	90
Volume [Å^3]	5455(2)	1601.88(13)	2550.1(9)	7135.6(14)	5466.3(17)	5539.2(5)	5540.4(8)	6013.9(9)	7735(4)	5428.2(9)	3349.8(3)	6497.1(8)
Z	8	2	1	8	4	4	4	4	4	4	4	4
ρ_{calc} [gcm^{-3}]	1.049	1.080	1.010	1.154	1.060	1.101	1.125	1.114	1.083	1.116	1.088	1.057
μ [mm^{-1}]	0.141	0.131	0.100	0.304	0.215	0.217	0.265	0.205	0.171	0.220	0.162	0.112
F(000)	1872	564	856	2640	1896	1976	2008	2168	2744	1976	1184	2266
Crystal size [mm^3]	0.67×0.31×0.20	0.22×0.17×0.16	0.42×0.37×0.09	0.49×0.26×0.22	0.30×0.26×0.25	0.35×0.23×0.18	0.33×0.30×0.26	0.16×0.14×0.13	0.66×0.24×0.13	0.30×0.30×0.30	0.18×0.15×0.10	0.30×0.30×0.30
Crystal colour	orange	red	orange	colourless	colourless	purple	dark red	red	red	yellow	colourless	red
Crystal shape	block	block	block	block	prism	block	block	block	block	block	block	block
Radiation	MoK_α (λ=0.71073 Å)	MoK_α (λ=0.71073 Å)	MoK_α (λ=0.71073 Å)	MoK_α (λ=0.71073 Å)	MoK_α (λ=0.71073 Å)	MoK_α (λ=0.71073 Å)	MoK_α (λ=0.71073 Å)	MoK_α (λ=0.71073 Å)	MoK_α (λ=0.71073 Å)	MoK_α (λ=0.71073 Å)	MoK_α (λ=0.71073 Å)	MoK_α (λ=0.71073 Å)
2θ range [°]	3.58 to 58.53 (0.73 Å)	2.55 to 56.56 (0.75 Å)	2.77 to 59.41 (0.72 Å)	2.03 to 59.24 (0.72 Å)	1.96 to 52.84 (0.80 Å)	3.05 to 63.06 (0.68 Å)	3.05 to 57.42 (0.74 Å)	3.05 to 53.48 (0.79 Å)	3.19 to 50.70 (0.83 Å)	3.13 to 53.54 (0.79 Å)	4.49 to 54.20 (0.78 Å)	3.10 to 52.75 (0.80 Å)
Index ranges	−27 ≤ h ≤ 27<br>−16 ≤ k ≤ 16<br>−31 ≤ l ≤ 31	−11 ≤ h ≤ 11<br>−15 ≤ k ≤ 15<br>−22 ≤ l ≤ 22	−16 ≤ h ≤ 16<br>−20 ≤ k ≤ 20<br>−20 ≤ l ≤ 20	−32 ≤ h ≤ 32<br>−20 ≤ k ≤ 20<br>−31 ≤ l ≤ 31	−15 ≤ h ≤ 15<br>−27 ≤ k ≤ 27<br>−27 ≤ l ≤ 27	−25 ≤ h ≤ 24<br>−23 ≤ k ≤ 23<br>−30 ≤ l ≤ 30	−23 ≤ h ≤ 23<br>−21 ≤ k ≤ 21<br>−27 ≤ l ≤ 27	−20 ≤ h ≤ 20<br>−20 ≤ k ≤ 20<br>−29 ≤ l ≤ 29	−12 ≤ h ≤ 12<br>−36 ≤ k ≤ 36<br>−28 ≤ l ≤ 28	−12 ≤ h ≤ 12<br>−26 ≤ k ≤ 26<br>−33 ≤ l ≤ 33	−15 ≤ h ≤ 15<br>−25 ≤ k ≤ 25<br>−18 ≤ l ≤ 11	−18 ≤ h ≤ 18<br>−32 ≤ k ≤ 32<br>−22 ≤ l ≤ 22
Reflections collected	152308	130940	83217	151999	98760	138413	100382	85184	57307	118344	87866	157989
Independent reflections	7412<br>R_{int} = 0.0517<br>R_{sigma} = 0.0173	7951<br>R_{int} = 0.0301<br>R_{sigma} = 0.0129	14387<br>R_{int} = 0.0371<br>R_{sigma} = 0.0260	19995<br>R_{int} = 0.0561<br>R_{sigma} = 0.0342	22404<br>R_{int} = 0.0863<br>R_{sigma} = 0.0697	18434<br>R_{int} = 0.0431<br>R_{sigma} = 0.0263	14326<br>R_{int} = 0.0485<br>R_{sigma} = 0.0283	12770<br>R_{int} = 0.0498<br>R_{sigma} = 0.0319	14109<br>R_{int} = 0.0635<br>R_{sigma} = 0.0624	11570<br>R_{int} = 0.1105<br>R_{sigma} = 0.0572	7377<br>R_{int} = 0.0677<br>R_{sigma} = 0.0386	13292<br>R_{int} = 0.1290<br>R_{sigma} = 0.0537
Complete to θ = 25.24°	100.0 %	99.9 %	100.0 %	100.0 %	100.0 %	100.0 %	100.0 %	100.0 %	99.7 %	100.0 %	99.9 %	100.0 %
Data / Restraints / Parameters	7412/0/282	7951/0/346	14387/1786/816	19995/0/746	22404/0/1093	18434/0/578	14326/0/577	12770/0/642	14109/712/949	11570/0/574	7377/0/356	13292/216/754
Goodness-of-fit on F^2	1.073	1.056	1.084	1.031	1.001	1.021	1.026	1.045	1.026	1.028	1.092	1.039
Final *R* indexes [$I \geq 2\sigma(I)$]	R_1 = 0.0382<br>wR_2 = 0.1064	R_1 = 0.0419<br>wR_2 = 0.1174	R_1 = 0.0576<br>wR_2 = 0.1520	R_1 = 0.0372<br>wR_2 = 0.0939	R_1 = 0.0478<br>wR_2 = 0.1028	R_1 = 0.0341<br>wR_2 = 0.0881	R_1 = 0.0348<br>wR_2 = 0.0891	R_1 = 0.0346<br>wR_2 = 0.0802	R_1 = 0.0643<br>wR_2 = 0.1678	R_1 = 0.0456<br>wR_2 = 0.0980	R_1 = 0.0484<br>wR_2 = 0.1270	R_1 = 0.0503<br>wR_2 = 0.1099
Final *R* indexes [all data]	R_1 = 0.0468<br>wR_2 = 0.1140	R_1 = 0.0437<br>wR_2 = 0.1191	R_1 = 0.0691<br>wR_2 = 0.1608	R_1 = 0.0425<br>wR_2 = 0.0979	R_1 = 0.0743<br>wR_2 = 0.1143	R_1 = 0.0460<br>wR_2 = 0.0964	R_1 = 0.0450<br>wR_2 = 0.0968	R_1 = 0.0422<br>wR_2 = 0.0843	R_1 = 0.1046<br>wR_2 = 0.1888	R_1 = 0.0698<br>wR_2 = 0.1095	R_1 = 0.0657<br>wR_2 = 0.1341	R_1 = 0.0821<br>wR_2 = 0.1269
peak/hole [eÅ^{-3}]	0.45/-0.28	0.55/-0.33	0.71/-0.29	0.91/-0.32	0.50/-0.43	0.47/-0.28	0.61/-0.23	0.27/-0.19	0.95/-0.79	0.37/-0.31	0.23/-0.51	0.36/-0.27
Flack X parameter								0.013(16)				

Computational Details

Structure Optimisation, Frequency Calculation and Thermochemical Approximations

Computational examination was performed using ORCA (version 4.2.1.).[17, 18] Structures were optimised starting from (where available) experimental X-Ray structures or modified input structures on the basis of experimental structures on RI-BP86-D3BJ[19] def2SVP/J model chemistry[20-24] in the gas phase followed by a frequency calculation on the same level of theory and when necessary, thermochemical corrections were taken from these frequency calculations. For numerical accuracy, grid6 and finalgrid7 were applied. No imaginary frequencies were observed confirming minima. Only for **A-Xyl (I3)**, **A-Me (I3)** and spurious imaginary frequencies (> i20 cm^{-1}) where found potentially due to numeric noise. Transition states were pre-screened by NEB (nudge elastic band) potential energy surface screens prior to transition state optimisations. Transition states have been found to possess one imaginary frequency along a reasonably expectable reaction coordinate. All structures were then reoptimized using BP86-D3BJ-def2TZVP/J model chemistry and all considered SCF energies, property calculations as well as NBO analyses[25, 26] are based on these gas phase structures. Graphical depictions were created using ChemCraft or IBOview.[27-29] For species considered in mechanistic discussions single point calculations using RI-BP86-(D3BJ)/def2TZVPP and CPCM solvation model for benzene where performed.

Thermochemical data for all compounds computationally probed are listed in the Table (see next page). The respective structures are documented as a combined .xyz-Structure-File separately provided.

NBO Analyses

NBO, NLMO and NRT analyses were conducted using NBO7.0.[25, 26] Wavefunctions to be analysed by NBO were obtained on the structures optimised on BP86-D3BJ-def2TZVP/J level of theory. For large molecules a BP86 single point calculation with def2-SVP basis sets on the organic substituents and def2-TZVP basis set for the elements of the central heterocyclic [C_4BSi]-fragment were chosen.

Selected NBO Data for 2A-Me

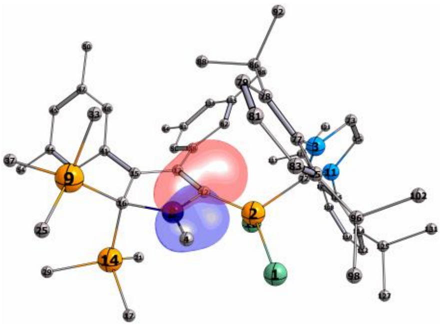

NBO Data for B8-C12 single and double bond:

101. (1.93335) BD (1) B 8- C 12 (28.18%) 0.5309* B 8 s(32.25%)p 2.10(67.61%)d 0.00(0.13%)f 0.00(0.02%)
(82.21%) 0.9067* C 12 s(0.20%)p 99.99(99.67%)d 0.37(0.08%) f 0.24(0.05%)

102. (1.62009) BD (2) B 8- C 12 (17.79%) 0.4217* B 8 s(0.13%)p 99.99(99.60%)d 1.85(0.24%) f 0.20(0.03%)
(82.21%) 0.9067* C 12 s(0.20%)p99.99(99.67%)d 0.37(0.08%) f 0.24(0.05%)

Depicition of NBO102 (C=B-Bond) (isolevel at 0.05 a.u.)

Excerpt of the SECOND ORDER PERTURBATION THEORY ANALYSIS OF FOCK MATRIX IN NBO BASIS for NBO 102

Threshold for printing: 0.50 kcal/mol

Donor (L) NBO	Acceptor (NL) NBO	E(2) kcal/mol	E(NL)-E(L) a.u.	F(L,NL) a.u.
..102. BD (2) B 8- C 12	248. BD*(1)Cl 1-Si 2	1.88	0.15	0.015
102. BD (2) B 8- C 12	249. BD*(1)Si 2-Cl 10	2.37	0.16	0.017
102. BD (2) B 8- C 12	250. BD*(1)Si 2- C 12	0.85	0.30	0.014
102. BD (2) B 8- C 12	251. BD*(1)Si 2- C 72	3.06	0.24	0.024
102. BD (2) B 8- C 12	255. BD*(1) C 4- H 5	0.64	0.47	0.015
102. BD (2) B 8- C 12	256. BD*(1) C 4- H 6	1.08	0.46	0.020
102. BD (2) B 8- C 12	260. BD*(2) B 8- C 12	12.21	0.26	0.050
102. BD (2) B 8- C 12	262. BD*(1)Si 9- C 16	0.96	0.32	0.016
102. BD (2) B 8- C 12	272. BD*(2) C 13- C 15	27.91	0.10	0.048

TD-DFT

TD-DFT calculations were performed in ORCA 4.2.1 using RIJCOSX-approximation and CAM-B3LYP functional[30] with def2-SVP/J basis set on all atoms. Single-point calculations were performed on the structures obtained from BP86 optimisation with def2-TZVP basis set as detailed above. Listed are the four absorptions of lowest wavenumbers and a depiction of the difference density plots (green: positive; magenta: negative) for the transition of lowest wavenumber. Exemplarily for compound **2a-Me**.

TD-DFT Compound 2a-Me

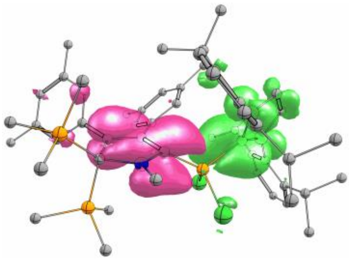

Difference density plot (isolevel at 0.002 a.u.) for transition 1

	energy (cm^{-1})	wavelength (nm)	f_{osc}	Relevant Transitions HOMO #246
1	18683.1	535.2	0.02124	245a -> 247a : 0.025526 (c= -0.15976872)<br>246a -> 247a : 0.943519 (c= 0.97134887)
2	28976.5	345.1	0.09077	246a -> 248a : 0.350637 (c= -0.59214599)<br>246a -> 249a : 0.034247 (c= -0.18505816)<br>246a -> 250a : 0.441711 (c= -0.66461365)<br>246a -> 251a : 0.062946 (c= 0.25088960)<br>246a -> 253a : 0.066755 (c= -0.25836976)
3	29482.7	339.2	0.17450	246a -> 248a : 0.140982 (c= 0.37547512)<br>246a -> 249a : 0.056159 (c= 0.23697830)<br>246a -> 251a : 0.059251 (c= 0.24341485)<br>246a -> 252a : 0.196907 (c= 0.44374223)<br>246a -> 253a : 0.500075 (c= -0.70715975)
4	30202.7	331.1	0.03181	246a -> 248a : 0.058263 (c= -0.24137662)<br>246a -> 249a : 0.209110 (c= -0.45728550)<br>246a -> 250a : 0.075921 (c= 0.27553704)<br>246a -> 251a : 0.416863 (c= -0.64564910)<br>246a -> 253a : 0.204767 (c= -0.45251205)

TD-DFT Compound 5b-Me

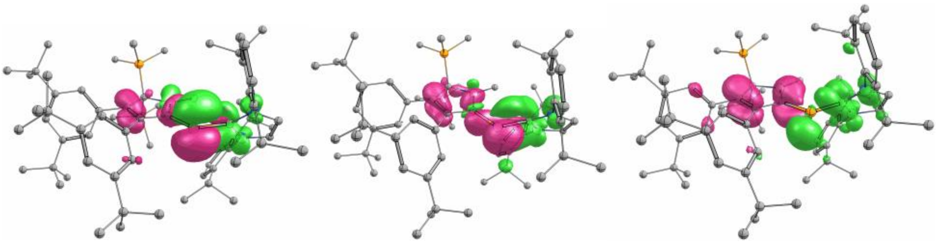

Difference density plots (isolevel at 0.002 a.u.) for transitions 1-3 (left to right)

	energy (cm^{-1})	wavelength (nm)	f_{osc}	Relevant Transitions HOMO #277
1	21943.0	455.7	0.23451	276a -> 278a : 0.289826 (c= 0.53835536)<br>277a -> 278a : 0.630658 (c= 0.79413966)
2	24599.6	406.5	0.09858	276a -> 278a : 0.598815 (c= -0.77383142)<br>276a -> 279a : 0.012628 (c= -0.11237369)<br>276a -> 282a : 0.011246 (c= 0.10604557)<br>277a -> 278a : 0.302462 (c= 0.54996556)<br>277a -> 283a : 0.011681 (c= -0.10807876)
3	29355.7	340.6	0.06149	275a -> 278a : 0.013876 (c= 0.11779472)<br>276a -> 279a : 0.011720 (c= -0.10825953)<br>277a -> 279a : 0.516667 (c= 0.71879521)<br>277a -> 280a : 0.139783 (c= -0.37387558)<br>277a -> 282a : 0.019190 (c= -0.13852833)<br>277a -> 283a : 0.186204 (c= 0.43151366)<br>277a -> 284a : 0.024156 (c= 0.15542337)
4	32484.6	307.8	0.29605	275a -> 278a : 0.012284 (c= -0.11083387)<br>276a -> 278a : 0.023048 (c= -0.15181709)<br>276a -> 279a : 0.264116 (c= 0.51392229)<br>276a -> 280a : 0.118301 (c= -0.34394922)<br>276a -> 282a : 0.018213 (c= -0.13495424)<br>276a -> 283a : 0.156073 (c= 0.39506125)<br>277a -> 279a : 0.094578 (c= 0.30753543)<br>277a -> 280a : 0.033312 (c= 0.18251559)<br>277a -> 283a : 0.033044 (c= -0.18178050)<br>277a -> 284a : 0.174195 (c= 0.41736690)

Tabulated Thermochemical Data for all Optimised Structures

			BP86-D3BJ\def2TZVP (gasphase structures); Thermal corrrections from def2-SVP calcs							BP86-D3BJ/ def2TZVPP (C6D6)
#	ENTRY	Description	E(SCF)	E(ZPV)corr	Hcorr	Gcorr	E(ZPV)	H	G	E(SCF)
1	Me4NHC	Me4NHC	-383,6052	0,1766	0,1878	0,1420	-383,4286	-383,4173	-383,4632	-383,6169
2	IDipp	IDipp	-1160,5608	0,5530	0,5846	0,4977	-1160,0078	-1159,9762	-1160,0631	-1160,5817
3	IDipp-SiCl2	IDipp-SiCl2	-2370,7668	0,5583	0,5952	0,4972	-2370,2085	-2370,1716	-2370,2696	-2370,7912
4	Me2NHC	Me2NHC Model Carbene	-304,9323	0,1226	0,1307	0,0918	-304,8097	-304,8016	-304,8405	-304,9424
5	Me2NHC-SiCl2	Me2NHC-SiCl2 Model Carbene Dichlorosilylene	-1515,1260	0,1287	0,1417	0,0906	-1514,9973	-1514,9844	-1515,0354	-1515,1401
6	B-Me	borole B-Me	-2128,8355	0,8909	0,9468	0,8138	-2127,9446	-2127,8887	-2128,0217	-2128,8648
7	B-Cl	borole B-Cl	-2549,2124	0,8571	0,9123	0,7802	-2548,3553	-2548,3001	-2548,4322	
8	A-Cl	borole A-Cl	-2077,2408	0,5280	0,5680	0,4623	-2076,7127	-2076,6727	-2076,7784	
9	A-Me	borole A-Me	-1656,8637	0,5618	0,6026	0,4960	-1656,3018	-1656,2611	-1656,3677	-1656,8838
10	A-pXyl	borole A-pXyl	-1927,3593	0,6678	0,7153	0,5956	-1926,6915	-1926,6440	-1926,7637	
11	A-Xyl	borole A-Xyl	-1927,3588	0,6677	0,7145	0,5963	-1926,6911	-1926,6443	-1926,7625	-1927,3826
12	M-Me	model borole M-Me	-1115,8709	0,3524	0,3790	0,3013	-1115,5185	-1115,4919	-1115,5696	-1115,8853
13	1b-Me	Bicyclic product 1b-Me	-3339,0632	0,8976	0,9575	0,8171	-3338,1656	-3338,1057	-3338,2461	-3339,0934
14	1b-pXyl	Bicyclic product 1b-pXyl	-3609,5611	1,0040	1,0704	0,9176	-3608,5571	-3608,4907	-3608,6434	-3609,5940
15	1b-Ph*	Bicyclic product 1b-Ph*	-3845,5474	1,1683	1,2424	1,0765	-3844,3791	-3844,3050	-3844,4709	
16	2b-Me	Silene-adduct 2b-Me	-4499,6954	1,4552	1,5459	1,3497	-4498,2402	-4498,1495	-4498,3457	-4499,7454
17	2b-Ph*	Silene-adduct 2b-Ph*	-5006,1726	1,7256	1,8310	1,6082	-5004,4470	-5004,3416	-5004,5645	
18	B-Me (I1)	NHC(SiCl2)-Borole B-Me Adduct @B	-4499,6722	1,4542	1,5454	1,3479	-4498,2180	-4498,1268	-4498,3243	-4499,7229
19	B-Me (I2)	NHC(SiCl2)-Borole B-Me Adduct @Calpha	-4499,6440	1,4542	1,5454	1,3479	-4498,1899	-4498,0987	-4498,2961	-4499,6954
20	B-Me (I3)	SiCl2-Silylene-Borole B-Me Adduct	-3339,0420	0,8974	0,9575	0,8165	-3338,1446	-3338,0845	-3338,2255	-3339,0713
21	B-Me (I4)	Inverse Bicycle 1b-Me	-3339,0417	0,8968	0,9571	0,8158	-3338,1449	-3338,0846	-3338,2258	-3339,0712
22	2b-Me-(SILENE)	Silene (NHC dissociation from 2b-Me)	-3339,0273	0,8973	0,9575	0,8161	-3338,1300	-3338,0698	-3338,2113	-3339,0572
23	3b-pXyl	Silene-adduct 3b-pXyl	-3993,2368	1,1843	1,2619	1,0881	-3992,0524	-3991,9749	-3992,1486	-3993,2796
24	4b-Me	Si(II) Half sandwich 4b-Me	-2418,4125	0,8935	0,9505	0,8160	-2417,5189	-2417,4620	-2417,5964	-2418,4418
25	5b-Me	IDipp-supported silavinylidene 5b-Me	-3579,0126	1,4492	1,5376	1,3450	-3577,5634	-3577,4750	-3577,6676	-3579,0618
26	1a-Me	Bicyclic product 1a-Me	-2867,0901	0,5684	0,6133	0,4987	-2866,5217	-2866,4768	-2866,5914	-2867,1113
27	1a-Cl	Bicyclic product 1a-Cl	-3287,4549	0,5338	0,5781	0,4643	-3286,9211	-3286,8768	-3286,9907	
28	1a-pXyl	Bicyclic product 1a-pXyl	-3137,5874	0,6744	0,7260	0,5983	-3136,9130	-3136,8614	-3136,9892	-3137,6114
29	1a-Xyl	Bicyclic product 1a-Xyl	-3137,5869	0,6740	0,7258	0,5968	-3136,9129	-3136,8610	-3136,9901	-3137,6112
30	2a-Me	Silene-adduct 2a-Me	-4027,7220	1,1260	1,2015	1,0313	-4026,5960	-4026,5205	-4026,6907	-4027,7635
31	2a-Cl	Silene-adduct 2a-Cl	-4448,1003	1,0913	1,1665	0,9964	-4447,0090	-4446,9338	-4447,1039	
32	2a-Xyl	Silene-adduct 2a-Xyl	-4298,2165	1,2323	1,3145	1,1317	-4296,9843	-4296,9020	-4297,0849	-4298,2606
33	A-Me (I1)	NHC(SiCl2)-Borole A-Me Adduct @B	-4027,6963	1,1251	1,2012	1,0294	-4026,5712	-4026,4951	-4026,6669	-4027,7387
34	A-Xyl (I1)	NHC(SiCl2)-Borole A-Xyl Adduct @B	-4298,1934	1,2323	1,3144	1,1321	-4296,9611	-4296,8790	-4297,0613	-4298,2376
35	A-Me (I2)	NHC(SiCl2)-Borole A-Me Adduct @Calpha	-4027,6705	1,1258	1,2011	1,0306	-4026,5448	-4026,4695	-4026,6399	-4027,7148
36	A-Me (I3)	SiCl2-Silylene-Borole A-Me Adduct	-2867,0699	0,5681	0,6123	0,4992	-2866,5018	-2866,4576	-2866,5706	-2867,0901
37	A-Xyl (I3)	SiCl2-Silylene-Borole A-Xyl Adduct	-3137,5661	0,6731	0,7245	0,5966	-3136,8930	-3136,8415	-3136,9695	-3137,5897
38	A-Me (I4)	Inverse Bicycle 1a-Me	-2867,0693	0,5676	0,6129	0,4975	-2866,5017	-2866,4564	-2866,5718	-2867,0897
39	A-Xyl (I4)	Inverse Bicycle 1a-Xyl	-3137,5615	0,6733	0,7255	0,5955	-3136,8882	-3136,8360	-3136,9661	-3137,5854
40	2a-Me-(SILENE)	Silene (NHC dissociation from 2a-Me)	-2867,0544	0,5682	0,6133	0,4982	-2866,4862	-2866,4411	-2866,5562	-2867,0752
41	2a-Xyl-(SILENE)	Silene (NHC dissociation from 2a-Xyl)	-3137,5470	0,6733	0,7257	0,5950	-3136,8737	-3136,8213	-3136,9520	-3137,5711
42	4a-pXyl	Si(II) Half sandwich 4a-pXyl (major conformer)	-2216,9382	0,6699	0,7187	0,5960	-2216,2683	-2216,2195	-2216,3422	
43	4a-pXyl-CONF	Si(II) Half sandwich 4a-pXyl (minor conformer)	-2216,9379	0,6701	0,7187	0,5969	-2216,2678	-2216,2192	-2216,3410	
44	M-Me (I1)	NHC(SiCl2)-Borole Adduct @B	-2631,0756	0,4856	0,5239	0,4240	-2630,5900	-2630,5516	-2630,6516	-2631,1003
45	M-Me (I3)	SiCl2-Silylene-Borole-Adduct	-2326,0738	0,3587	0,3895	0,3036	-2325,7151	-2325,6843	-2325,7702	-2326,0878
46	M-Me (TS1)	Transition State I3 --> I4	-2326,0727	0,3591	0,3893	0,3044	-2325,7136	-2325,6834	-2325,7682	-2326,0866
47	M-Me (I4)	Inverse Bicycle	-2326,0793	0,3585	0,3893	0,3033	-2325,7208	-2325,6900	-2325,7760	-2326,0933
48	M-Me (TS2)	Transition State I4 --> P1	-2326,0773	0,3574	0,3877	0,3027	-2325,7200	-2325,6896	-2325,7746	-2326,0915
49	P1	Bicyclic Product	-2326,0975	0,3596	0,3900	0,3046	-2325,7380	-2325,7075	-2325,7929	-2326,1127
50	M-Me (I2)	NHC(SiCl2)-Borole Adduct @Calpha	-2631,0834	0,4863	0,5242	0,4250	-2630,5971	-2630,5592	-2630,6584	-2631,1059
51	M-Me (TS-3)	Transition State I2 ---> I5	-2631,0519	0,4845	0,5224	0,4231	-2630,5673	-2630,5295	-2630,6287	-2631,0787
52	M-Me (I5)	TMS-Borate Intermediate	-2631,0579	0,4852	0,5236	0,4228	-2630,5727	-2630,5343	-2630,6351	-2631,0822
53	M-Me (TS-4)	Transition State I5 ---> P2	-2631,0558	0,4845	0,5225	0,4224	-2630,5713	-2630,5332	-2630,6334	-2631,0798
54	P2	Sileneadduct Product	-2631,0800	0,4852	0,5238	0,4228	-2630,5947	-2630,5562	-2630,6571	-2631,1051
55	P2-(SILENE)	Silene (NHC dissociation from P2)	-2326,0615	0,3591	0,3898	0,3039	-2325,7024	-2325,6717	-2325,7576	-2326,0754

Comment on the Computationally Assessed Mechanistic Proposal

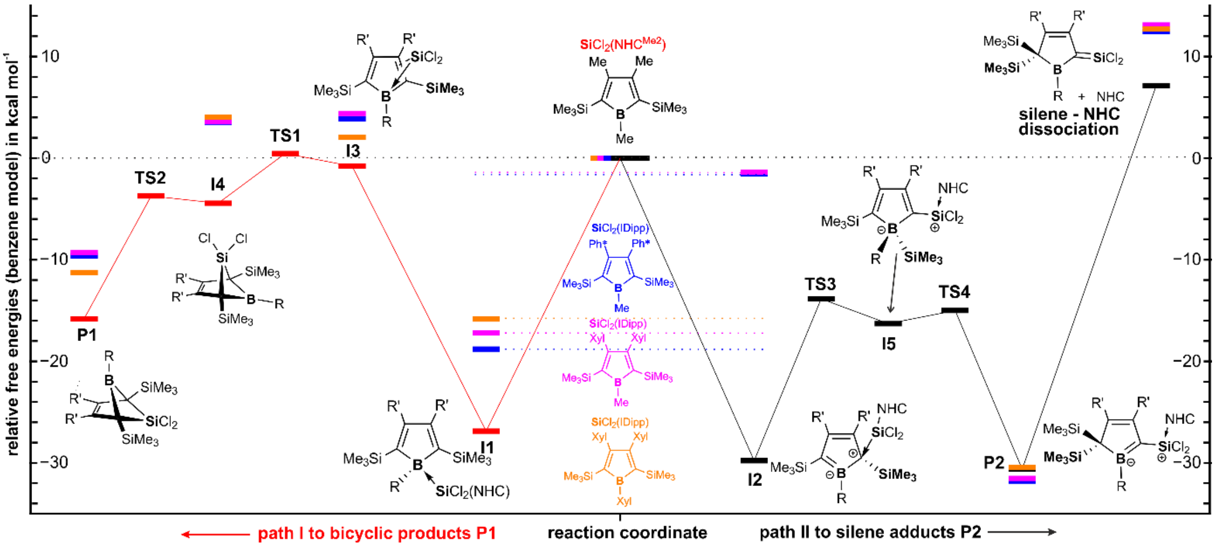

Graphical Summary of computationally probed species involved in the putative processes proposed to be involved. Red and black lines and data points refer to a model system of strongly reduced steric bulk both in the NHC($SiCl_2$) and the borole. Solid lines not connecting to a transition states should just be considered an aide to the eye. Orange, pink and blue data points refer to the respective actually experimentally probed systems depicted.

Note: The data points for the model system likely do not adequately reflect the energetic profile of the experimentally probed species as sterics and ligand repulsion likely play an important role. Therefore, barriers calculated only for the model system may serve as an argument for likely small barriers associated with similar intramolecular migrations, but whenever intermolecular processes or associations/dissociations are involved, the model system likely does not provide useful energies for comparison with experimental observations.

To approach the experimental observation of two competing product formation pathways from the reaction of boroles with $SiCl_2$(IDipp) we propose individual steps toward each product **1** or **2** (Scheme 8). Intuitively, the anticipated first step would be the Lewis-adduct formation (**I1**) of the Lewis-base $SiCl_2$(IDipp) adding to the Lewis-acidic boron atom. So reported an example of a silylene-donor adduct to pentaphenyl borole.[31] An alternative target for a nucleophilic attack at boroles is the C_α atom which reveals a vinylogous connection to the boron-site to result in a zwitter-ionic boratabutadiene-type adduct **I2**.[32] Braunschweig previously reported on a reversible B-/ C_α-atom adduct formation of 2,6-lutidin to boroles.[33, 34] None of these putative intermediates **I1** and **I2** have been experimentally observed in the course of the reaction but we consider the decisive forking of the pathway to occur at this stage. The relative stabilities of each of these isomeric adducts are strongly depending on the substitution pattern. Computational assessment (BP86/def2-TZVPP and benzene solvation model) of a model borole of drastically reduced steric bulk (1,3,4-trimethyl-2,5-$(TMS)_2$ borole) and model carbene (1,3-dimethylimidazol-2-ylidene suggests strongly exergonic (ca. –29 kcal mol^{-1}) formations of both adducts **I1** and **I2** from the free borole and $SiCl_2$(NHC) with **I2** being even slightly more stable. However, when actual experimentally probed substitution patterns are assessed an inverse relative stability is observed and the respective C_α adducts **I2** are by far less exergonic (ca. –2 kcal mol^{-1}) than the B-adducts **I1** (ca. –17 kcal mol^{-1}).

Pathway I assumes an energetically uphill dissociation of the NHC. Computational NHC removal from both adducts **I1** and **I2** affords the identical structure **I3** after optimization. **I3** can be described as an adduct of an ambiphilic dichlorosilylene to the equally ambiphilic borole. The dissociation free energy of the NHC from **I1** or **I2** is prohibitively high (ca. 22-27 kcal mol^{-1}) for the small carbene in our model but is reasonably accessible for the more bulky IDipp derivatives. **I3** is the intermediate before a low barrier (ca. 1 kcal mol^{-1} – identified for the reduced model system) 2-electron redox transfer, which is essentially a [1+4]-cycloaddtion reaction, from the silylene into the π-system of the borole to form **I4.** **I4** is an isomer of 5-sila-6-borabicyclo[2.1.1]hex-2-ene in which the borane moiety remains in plane without interactions to the olefinic π-system in its backbone. A second low-barrier (ca. 1 kcal mol^{-1}) rearrangement transforms **I4** in the final bicyclic product **P1**. The process **I3→I4→P1** is continuously energetically downhill with very small barriers.

Pathway II originates from the C_α-adduct **I2** from which a 1,2-$SiMe_3$ migration from C_α to the B-atom occurs forming the silyl borate intermediate **I5**. **I5** was only identified as a local minimum for the model system of reduced steric bulk and forms from **I2** via a reasonably accessible energy barrier (ca. 15 kcal mol^{-1}). Experimentally, no evidence for the involvement of a species of the **I5**-type were observed. This is in line with a very small computationally predicted barrier (ca 1 kcal mol^{-1}) for the subsequent 1,2-$SiMe_3$-migration to give the final silene-adduct product **P2**. The involvement of a species like **I5** (i.e. silyl migration *via* boron) is further supported by the complete suppression of $SiMe_3$-migration (and thus **P2** formation) when the boron-bound aryl groups are bearing one *ortho*-methyl group, effectively blocking the B-atom migration route. A case for comparable sigmatropic 1,2- and 1,3-shifts of hydrogen in borole-NHC adducts have been described by Braunschweig and coworkers.[35] For all substitution variants probed, despite considerable steric bulk and thus repulsive interactions involved, the silene adducts **P2** were the global energy minimum of all species investigated and thermodynamically much more favoured than the bicyclic species **P1**. The energetic profile of the model system suggests reasonable barriers for the key rearrangements but does not entirely rationalize all experimental observations for the real systems which likely goes back to vastly differing steric profiles.

A key experimental finding is that mixing the starting materials in the cold favors the formation of the silene adduct suggesting that a decisive energy barrier at an early stage after bifurcation must be lower on the path towards **P2**. Given that the critical NHC-dissociation (**I1/I2→I3**) on the path to the bicyclic products **P1** is predicted to be energetically more demanding than the barrier (**I2→TS3→I5**) we suggest this to be the putative critical step. Notably, for derivatives with suitably balanced steric profile to allow for low-temperature controlled selective formation of the silene adducts (i.e. sufficient suppression of bicyclus formation) such as **2a-Xyl** or **2b-Xyl**, a fairly clean and irreversible conversion into the respective bicyclic products **1a-Xyl** or **1b-Xyl** and free NHC is observed at elevated temperatures of 40-80°C. We suggest that this reverse rearrangement returns to **I2** via the initial formation pathway rather than alternative routes involving the dissociation of the NHC from the adducts **P2** to putative unsupported silenes. This dissociation and silene generation would, in all cases, be prohibitively unfavoured (ca. 37-43 kcal mol^{-1}).

The model compound potential energy surface suggests similarly high energy barriers for the reverse reaction from the bicyclic products **P1→TS2→I4** as from **P2→TS4→I5** which would also indicate a potential reversibility of the formation of **P1** to ultimately yield the silene adduct product class **P2**. This reaction should in principle be thermodynamically favoured as **P2** is predicted to be more stable than **P1**. However, the formation of the **P2** from treating bicylic products **P1** with IDipp has not been experimentally observed.

Literature

1. R. K. Harris, E. D. Becker, S. M. Cabral de Menezes, R. Goodfellow and P. Granger, *Pure Appl. Chem.*, 2001, **73**, 1795-1818.
2. T. Heitkemper, L. Naß and C. P. Sindlinger, *Dalton Trans.*, 2020, **49**, 2706 - 2714.
3. J. Sarcevic, T. Heitkemper and C. P. Sindlinger, *Chem. Commun.*, 2022, **58**, 246-249.
4. T. Heitkemper, L. Naß and C. P. Sindlinger, *Angew. Chem. Int. Ed.*, 2021, **60**, 20055-20060.
5. T. Heitkemper, J. Sarcevic and C. P. Sindlinger, *J. Am. Chem. Soc.*, 2020, **142**, 21304-21309.
6. L. Hintermann, *Beilstein J. Org. Chem.*, 2007, **3**, 22.
7. R. S. Ghadwal, H. W. Roesky, S. Merkel, J. Henn and D. Stalke, *Angew. Chem. Int. Ed.*, 2009, **48**, 5683-5686.
8. N. Kuhn and T. Kratz, *Synthesis*, 1993, 561-562.
9. S. J. Bonyhady, C. Jones, S. Nembenna, A. Stasch, A. J. Edwards and G. J. McIntyre, *Chem. Eur. J.*, 2010, **16**, 938-955.
10. N. Weyer, M. Heinz, J. I. Schweizer, C. Bruhn, M. C. Holthausen and U. Siemeling, *Angew. Chem.*, 2021, **133**, 2656-2660.
11. SAINTv8.30C, Bruker AXS, WI, USA, Madison, **2013**.
12. G. M. Sheldrick, SADABS, **2008** University of Göttingen, Göttingen, Germany.
13. G. M. Sheldrick, *Acta Cryst.*, 2015, **A71**, 3.
14. G. M. Sheldrick, *Acta Cryst.*, 2015, **C71**, 3.
15. C. B. Hübschle, G. M. Sheldrick and B. Dittrich, *J. Appl. Crystallogr.*, 2011, **44**, 1281-1284.
16. D. Kratzert and I. Krossing, *J. Appl. Crystallogr.*, 2018, **51**, 928-934.
17. F. Neese, *Wiley Interdiscip. Rev. Comput. Mol. Sci.*, 2012, **2**, 73-78.
18. F. Neese, *Wiley Interdiscip. Rev. Comput. Mol. Sci.*, 2018, **8**, e1327.
19. S. Grimme, S. Ehrlich and L. Goerigk, *J. Comput. Chem.*, 2011, **32**, 1456-1465.
20. A. D. Becke, *Phys. Rev. A*, 1988, **38**, 3098-3100.
21. J. P. Perdew and W. Yue, *Phys. Rev. B*, 1986, **33**, 8800-8802.
22. A. Schäfer, C. Huber and R. Ahlrichs, *J. Chem. Phys.*, 1994, **100**, 5829-5835.
23. F. Weigend and R. Ahlrichs, *Phys. Chem. Chem. Phys.*, 2005, **7**, 3297-3305.
24. K. Eichkorn, F. Weigend, O. Treutler and R. Ahlrichs, *Theor. Chem. Acc.*, 1997, **97**, 119-124.
25. E. D. Glendening, C. R. Landis and F. Weinhold, *Wiley Interdiscip. Rev. Comput. Mol. Sci.*, 2012, **2**, 1-42.
26. E. D. Glendening, J. K. Badenhoop, A. E. Reed, J. E. Carpenter, J. A. Bohmann, C. M. Morales, P. Karafiloglou, C. R. Landis and F. Weinhold, NBO7.0., **2018**.
27. G. A. Zhurko, Chemcraft - graphical software for visualization of quantum chemistry computations. https://www.chemcraftprog.com; Version 1.8 (build610b), **2014**
28. G. Knizia and J. E. M. N. Klein, *Angew. Chem. Int. Ed.*, 2015, **54**, 5518-5522.
29. G. Knizia, IboView, **2015.**
30. T. Yanai, D. P. Tew and N. C. Handy, *Chem. Phys. Lett.*, 2004, **393**, 51-57.
31. Y. Li, R. K. Siwatch, T. Mondal, Y. Li, R. Ganguly, D. Koley and C.-W. So, *Inorg. Chem.*, 2017, **56**, 4112-4120.
32. F. Ge, G. Kehr, C. G. Daniliuc and G. Erker, *Organometallics*, 2015, **34**, 229-235.
33. H. Braunschweig, C.-W. Chiu, D. Gamon, K. Gruß, C. Hörl, T. Kupfer, K. Radacki and J. Wahler, *Eur. J. Inorg. Chem.*, 2013, **2013**, 1525-1530.
34. K. Ansorg, H. Braunschweig, C. W. Chiu, B. Engels, D. Gamon, M. Hügel, T. Kupfer and K. Radacki, *Angew. Chem. Int. Ed.*, 2011, **50**, 2833-2836.
35. H. Braunschweig, C.-W. Chiu, T. Kupfer and K. Radacki, *Inorg. Chem.*, 2011, **50**, 4247-4249.
